# The Gut-Brain Axis in Healthy Females: Lack of Significant Association between Microbial Composition and Diversity with Psychiatric Measures

**DOI:** 10.1371/journal.pone.0170208

**Published:** 2017-01-19

**Authors:** Susan C. Kleiman, Emily C. Bulik-Sullivan, Elaine M. Glenny, Stephanie C. Zerwas, Eun Young Huh, Matthew C. B. Tsilimigras, Anthony A. Fodor, Cynthia M. Bulik, Ian M. Carroll

**Affiliations:** 1 Department of Nutrition, The University of North Carolina at Chapel Hill, Chapel Hill, North Carolina, United States of America; 2 Department of Medicine, The University of North Carolina at Chapel Hill, Chapel Hill, North Carolina, United States of America; 3 Department of Psychiatry, The University of North Carolina at Chapel Hill, Chapel Hill, North Carolina, United States of America; 4 Department of Bioinformatics and Genomics, The University of North Carolina at Charlotte, Charlotte, North Carolina, United States of America; 5 Department of Medical Epidemiology and Biostatistics, Karolinska Institutet, Stockholm, Sweden; 6 Center for Gastrointestinal Biology and Disease, The University of North Carolina at Chapel Hill, Chapel Hill, North Carolina, United States of America; University of Illinois at Urbana-Champaign, UNITED STATES

## Abstract

**Objective:**

This study examined associations between the composition and diversity of the intestinal microbiota and measures of depression, anxiety, eating disorder psychopathology, stress, and personality in a group of healthy adult females.

**Methods:**

Female participants (n = 91) ages 19–50 years with BMI 18.5–25 kg/m^2^ were recruited from central North Carolina between July 2014 and March 2015. Participants provided a single fecal sample and completed an online psychiatric questionnaire that included five measures: (i) Beck Anxiety Inventory; (ii) Beck Depression Inventory-II; (iii) Eating Disorder Examination-Questionnaire; (iv) Perceived Stress Scale; and (v) Mini International Personality Item Pool. Bacterial composition and diversity were characterized by Illumina sequencing of the 16S rRNA gene, and associations were examined using Kendall’s tau-b correlation coefficient, in conjunction with Benjamini and Hochberg’s False Discovery Rate procedure.

**Results:**

We found no significant associations between microbial markers of gut composition and diversity and scores on psychiatric measures of anxiety, depression, eating-related thoughts and behaviors, stress, or personality in a large cohort of healthy adult females.

**Discussion:**

This study was the first specifically to examine associations between the intestinal microbiota and psychiatric measures in healthy females, and based on 16S rRNA taxonomic abundances and diversity measures, our results do not suggest a strong role for the enteric microbe-gut-brain axis in normal variation on responses to psychiatric measures in this population. However, the role of the intestinal microbiota in the pathophysiology of psychiatric illness may be limited to more severe psychopathology.

## Introduction

Investigations conducted over the last decade have generated consensus among researchers that the intestinal microbiota plays a vital role in a range of physiologic processes, especially those related to immunologic and metabolic function. Emerging evidence also suggests that a healthy intestinal microbiota is important for normal brain development [1]. The enteric microbe-gut-brain axis has garnered increasing attention as a key, bidirectional communication pathway that influences mood, cognition, and behavior [2–4]. In addition to a direct connection via the vagus nerve, it may be possible that gut bacteria interact with the brain through production of neurotransmitters, hormones, and other metabolites [5].

Whether a dysbiosis in the intestinal microbiota, an unhealthy change in the normal microbial ecology of the gut, plays a direct role in the pathophysiology of psychiatric disorders remains to be determined; however, both preclinical animal studies and clinical human studies are actively investigating this question. Numerous studies in animal models have documented behavioral changes following manipulation of the intestinal microbiota, including effects on behavior associated with stress [6], anxiety [1, 7–9], and depression [5, 10]. Validating animal models of the enteric microbe-gut-brain axis in human populations has reported modest associations and has been limited by small sample sizes and lack of consistency in assessment of psychiatric and microbial outcome measures. Although underpowered, these studies in human cohorts suggest a potential role for the intestinal microbiota in anxiety, depression, stress, cognitive reactivity, and eating disorders [11–15].

The majority of the emerging data reporting a relationship between the intestinal microbiota and psychological/behavioral traits has been demonstrated in either rodents or in humans who suffer from threshold psychiatric disorders [16]. It is unknown whether these associations hold across the entire spectrum of severity of psychopathology, or if they are confined to the more pathological extremes. Our goal, therefore, was to determine whether the associations between the intestinal microbiota and psychological measures hold across the spectrum of severity of our target psychological dimensions in healthy individuals. We examined associations between the composition and diversity of the intestinal microbiota and measures of depression, anxiety, eating disorder psychopathology, stress, and personality in a group of healthy adult females.

## Materials and Methods

The study was approved by the Biomedical Institutional Review Board at the University of North Carolina at Chapel Hill. All participants provided written consent before study participation.

### Study Population

To limit the natural variability in the composition of the intestinal microbiota between healthy individuals, we recruited healthy adult females (n = 100) ages 19–50 years with BMI 18.5–25.0 kg/m^2^ from central North Carolina via listserv announcements, targeted emails, and social media to serve as controls for ongoing research (clinicaltrials.gov—NCT01916538). Participants were recruited between July 2014 and March 2015. Due to possible impact on the intestinal microbiota, potential participants were excluded for the following reasons: (i) history of gastrointestinal tract surgery (other than appendectomy or cholecystectomy); (ii) history of inflammatory bowel diseases, irritable bowel syndrome, or celiac disease; (iii) history of eating disorders (anorexia nervosa, bulimia nervosa, binge-eating disorder); (iv) treatment in the last two months with antibiotics or steroids; (v) intentional use of probiotics during the last two months (via food or supplement); and/or (vi) abuse of laxatives within the last month.

### Body Composition and Assessments

Participants self-reported current height and weight during the screening process. Participants completed an online psychiatric questionnaire that included five widely used and validated measures: (i) Beck Anxiety Inventory (BAI) [17, 18]; (ii) Beck Depression Inventory-II (BDI) [19, 20]; (iii) Eating Disorder Examination-Questionnaire (EDE-Q) [21, 22]; (iv) Perceived Stress Scale-10 (PSS) [23, 24]; and (v) Mini International Personality Item Pool (Mini IPIP) [25].

### Sample Collection, Processing, and Storage

During the consent process, participants were provided with an at-home stool collection kit and trained in sample collection procedures. Each kit included: Styrofoam container, disposable collection hat, stool collection tube, biohazard bag, pair of non-latex gloves, two ice packs, and stool collection record sheet. Participants were instructed to return the sample (in the biohazard bag, with ice packs, in the Styrofoam box) to the research office within 24 hours of collection and to keep the sample refrigerated during any interim period. Samples were then immediately transferred to the laboratory, where they were mechanically homogenized with a sterile spatula, aliquoted into sterile 2 ml cryotubes, and stored in a −80°C freezer for future DNA isolation and molecular microbiological analysis.

### DNA Isolation

Bacterial DNA was isolated from collected samples using a phenol/chloroform extraction method combined with physical disruption of bacterial cells and a DNA clean-up kit (QIAmp DNA Stool Mini Kit [Qiagen, Valencia, CA]), as previously described [26, 27].

### Sequencing of 16S rRNA Genes

Bacterial community composition in isolated DNA samples was characterized by amplification of the V4 variable region of the 16S rRNA gene by polymerase chain reaction (PCR) (forward primer 515, 5'-GA GTG CCA GCM GCC GCG GTA A-3'; reverse primer 806, 5'-ACG GAC TAC HVG GGT WTC TAA T-3'). Forward and reverse primers incorporated single-nucleotide phase shifts of six different lengths each, to improve the quality of the sequence data generated. Generation of 16S rRNA sequences consisted of two separate amplifications: (1) 95°C for three minutes, then 10 cycles of 95°C for 30 seconds, 50°C for 30 seconds, and 72°C for 30 seconds, followed by one cycle of 72°C for five minutes using 120 ng of fecal DNA as template, mixes of the 6 forward and 6 reverse 16S V4 primers at a final concentration of 10 μM, and the KAPA2G Robust PCR kit (Kapa Biosystems, Wilmington, MA); and (2) 95°C for three minutes, then 22 cycles of 95°C for 30 seconds, 50°C for 30 seconds, and 72°C for 30 seconds, followed by one cycle of 72°C for five minutes using 5 μL of purified PCR product from the first amplification as template, 10 μM of forward and reverse primers that contain Illumina MiSeq adapter sequences with a 12-base error-correcting Golay barcode incorporated in the reverse primer, and the KAPA HiFi HotStart ReadyMix PCR kit [28]. Purification of PCR products was carried out after each amplification using the HighPrep PCR clean-up kit (MagBio, Lausanne, Switzerland) with a DynaMag-96 side magnet (Life Technologies, Carlsbad, CA). Equimolar 16S rRNA PCR products were then quantified and pooled for sequencing. Sequencing was performed on an Illumina MiSeq desktop sequencer (Illumina, San Diego, CA) by the High-Throughput Sequencing Facility in the Carolina Center for Genome Sciences at the UNC School of Medicine. Demultiplexed MiSeq data for each of the 91 participants can be accessed on the MG-RAST online metagenomics server [29]: http://metagenomics.anl.gov/linkin.cgi?project=mgp20265 (Project ID: mgp20265).

### Analysis of 16S rRNA Sequences

16S rRNA sequencing data were processed by the Quantitative Insights Into Microbial Ecology (QIIME) pipeline [30], with quality filtering as previously described [27]. Forward sequence reads (250 bp) were clustered into Operational Taxonomic Units (OTUs) based on their sequence similarity at a 97% threshold using BLAST and assigned taxonomy using the Greengenes database [31]. Principal coordinates were generated using unweighted and weighted UniFrac distances [32–34].

Results were validated using an alternate pipeline, in which forward reads from the 16S rRNA sequencing data were classified with version 2.10.1 of the RDP classifier with a threshold of a 50% RDP score [35].

Statistical significance was determined using Kendall’s tau-b correlation coefficient in R [36]. R scripts are available at: https://github.com/mcbtBINF/healthyCohort/.

The diversity of the intestinal microbiota was characterized by the Shannon diversity index [37, 38].

### Statistical Analysis

Associations between psychiatric and microbial measures were examined using Kendall’s tau-b correlation coefficient, in conjunction with Benjamini and Hochberg’s False Discovery Rate (FDR) procedure to correct for multiple comparisons [39]. Psychiatric measures included: BAI (anxiety), BDI (depression), EDE-Q (total + subscales for dietary restraint, eating concern, shape concern, and weight concern), PSS (stress), and Mini-IPIP (scales for extraversion, agreeableness, conscientiousness, neuroticism, and imagination). Microbial measures included: alpha diversity (Shannon diversity index) and taxa abundance of bacterial groups at the phylum, class, order, family, and genus levels. Linear models were additionally constructed wherein the first two principal coordinates were regressed against each of the psychiatric measures and other participant metadata. The FDR procedure was applied to the number of comparisons per outcome and per taxonomic rank. The α level used was 0.05, but for FDR correction, a more lenient criterion of 0.1 was used [40]. The minimal effect sizes for >80% and >95% power, respectively, are 0.08 and 0.14 at the genus level based on Kendall’s tau-b using simulated data with various degrees of correlation at 10% FDR. All analyses were conducted in R [36]. R scripts are available at: https://github.com/mcbtBINF/healthyCohort/.

## Results

Of 100 participants who consented to participate in the study, 94 completed the psychiatric questionnaires *and* submitted a fecal sample, of which sequencing results from 91 samples met minimal sequencing depth standards for analysis. Demographic and clinical characteristics of the final participant sample (n = 91) are shown in Table 1. In brief, the participants had a mean (SD) age of 29.0 (7.9) years and were within the normal or healthy weight range for adults [41]. On average, their scores indicate normal or minimal levels of anxiety (BAI), depression (BDI), and stress (PSS) and are in line with, or lower than, those of similar non-clinical samples [18, 42–44]. Total scores on the EDE-Q and its four subscales (dietary restraint and eating, weight, and shape concerns) are lower than norms for U.S. college students and young adult females in Sweden and Australia [45–47], which is likely a reflection of the participant recruitment and screening process, which eliminated individuals with a lifetime eating disorder history.

**Table 1:** Demographic and clinical characteristics of participants in this study (n = 91) as compared to clinical and normative values.

Metric	Our cohort—mean (SD)	Our cohort—range	Possible range	Clinical/severity thresholds or “case” values—mean (SD)	Normative values in other healthy populations—mean (SD)
Age (years)	29.0 (7.9)	19–50	-----	-----	-----
BMI (kg/m^2^)	21.7 (1.9)	18.5–25.0	-----	18.5–24.9 = normal or healthy weight [41]	In 2015, 37.4% of NC women fell in this range [48]
BAI	5.0 (4.8)	0–19	0–63 [49]	Scores of <9 = “normal or no anxiety” [49]	6.6 (8.1) [50]
BDI-II	5.2 (5.9)	0–35	0–63 [42]	Scores of <13 = below threshold for depression [42]	8.32 (7.74) [51]
EDE-Q Total	0.6 (0.5)	0–2.7	0–6^a^	3.09 (0.83) [22]	1.52 (1.25) [46]
• Dietary restraint	0.4 (0.6)	0–2.8	0–6 [52]	2.65 (1.48) [22]	1.30 (1.40) [46]
• Eating concern	0.2 (0.2)	0–1.4	0–6 [52]	2.02 (0.95) [22]	0.76 (1.06) [46]
• Shape concern	1.1 (0.8)	0–4.6	0–6 [52]	4.01 (0.98) [22]	2.23 (1.65) [46]
• Weight concern	0.7 (0.8)	0–4.5	0–6 [52]	3.68 (1.08) [22]	1.79 (1.51) [46]
PSS (10-item)	12.4 (6.3)	0–30	0–40	-----^b^ [53]	23.2 [54]
Mini-IPIP:					
• Extraversion	12.6 (4.1)	4–20	4–20 [25]	-----	12.99 (3.83) [55]
• Neuroticism	9.9 (3.4)	4–17	4–20 [25]	-----	11.81 (3.72) [55]
• Agreeableness	16.5 (2.6)	11–20	4–20 [25]	-----	16.57 (2.85) [55]
• Conscientiousness	15.2 (2.9)	8–20	4–20 [25]	-----	13.22 (3.53) [55]
• Intellect/imagination	14.5 (3.1)	7–20	4–20 [25]	-----	15.81 (3.11) [55]

BMI, body mass index; BAI, Beck Anxiety Inventory; BDI, Beck Depression Inventory-II; EDE-Q, Eating Disorder Examination-Questionnaire; PSS, Perceived Stress Scale-10; Mini-IPIP, Mini-International Personality Item Pool.

^a^Average of subcategory values.

^b^The PSS is not used to index diagnostic thresholds. Higher scores reflect higher perceived stress.

-----No suitable clinical data available, e.g. there is no clinical threshold for Agreeableness.

Following sequencing of 16S rRNA genes, we had 91 samples with complete data, after excluding those samples with insufficient depth of sequence reads for our downstream analysis.

The total number of 16S rRNA sequence reads was 15,391,194, and the mean number of reads was 169,134 per sample (range: 47,492–317,380 sequence reads).

**Fig 1:**
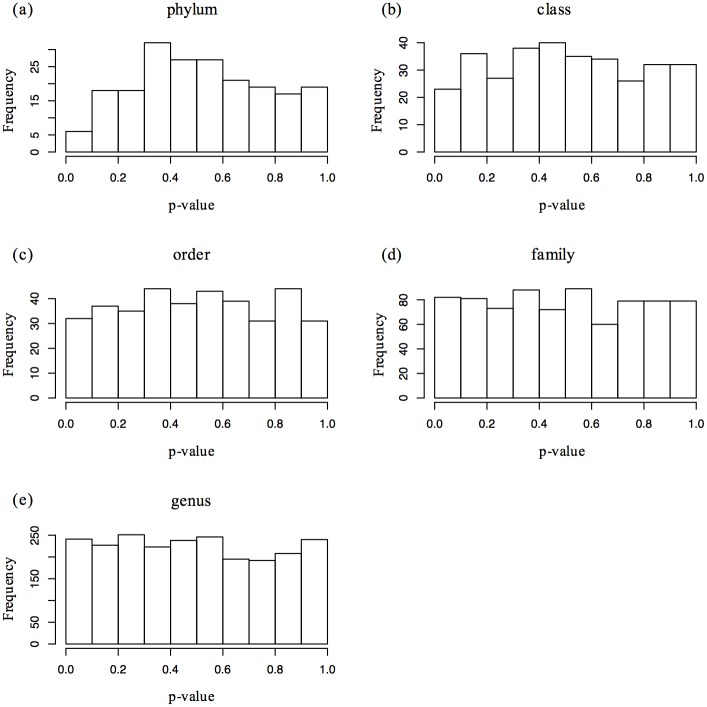
Histograms of p-values for associations with psychiatric measures by taxonomic level. Associations between psychiatric and microbial measures were examined using Kendall’s tau-b correlation coefficient, in conjunction with Benjamini and Hochberg’s False Discovery Rate procedure, using data generated by the RDP classifier. Psychiatric measures included: Beck Anxiety Inventory, Beck Depression Inventory-II, Eating Disorder Examination-Questionnaire, Perceived Stress Scale, and Mini-International Personality Item Pool. P-value frequencies were examined at each taxonomic level: (a) phylum; (b) class; (c) order; (d) family; and (e) genus.

When examining associations between psychiatric measures and the composition and diversity of the intestinal microbiota, there were no associations that met established significance thresholds. We considered 17 different measures from our human participants—the 15 measures in Table 1 plus participant height and weight. The RDP classifier reported 232 non-rare taxa (12 phyla, 19 classes, 22 orders, 46 families, and 133 genera) that were present in at least 25% of our samples. At each taxonomic level, we also calculated the Shannon diversity index. We therefore evaluated 3,944 hypotheses [17 measures * (232 taxa + 5 Shannon diversity metrics)] using the non-parametric Kendall’s tau-b test for association. Histograms of generated p-values across all possible associations (Fig 1) are largely uniform, suggesting that the null hypothesis of no association is generally supported across all taxonomic levels. Using FDR correction for all 3,944 hypotheses, there were no significant hits even if the threshold were set to 93% FDR (S1 Table).

We also used a less conservative correction, in which associations between each of the 17 human measurements and each taxonomic level were corrected independently (for example, the comparisons of BDI and the 12 phyla were corrected only for the 12 phyla independent of all the other tests that we ran). Even using this much less stringent threshold, where we might expect some spurious correlations, there were no significant hits at a 5% FDR. We conclude that there is a striking lack of correlation between microbial community composition and the measurements we have gathered from this human cohort.

**Fig 2:**
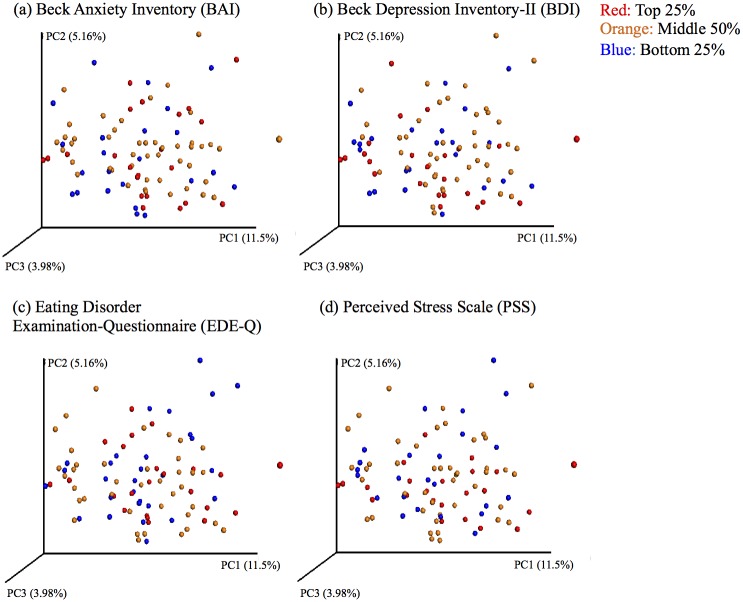
Principal coordinate plots of psychiatric measures by quartile. Principal coordinates were generated using unweighted UniFrac distances from the QIIME pipeline and allocated to quartiles (red: top quartile; orange: middle two quartiles; blue: bottom quartile) based on scores from the (a) Beck Anxiety Inventory; (b) Beck Depression Inventory-II; (c) Eating Disorder Examination-Questionnaire; and (d) Perceived Stress Scale. Plots are based on the first three principal coordinates, which explain 11.5% (PC1), 5.16% (PC2), and 3.98% (PC3) of the variance in microbial composition, and do not cluster by quartile—supporting a lack of association between microbial markers and these psychiatric measures in healthy individuals.

To further visualize the associations in our data set, we generated principal coordinate plots using unweighted UniFrac distances and colored these plots by quartiles of the main psychiatric measures of interest (BAI, BDI, EDE-Q total, PSS) (Fig 2). These plots are based on the first three principal coordinates, which explain 11.5% (PC1), 5.16% (PC2), and 3.98% (PC3) of the variance in microbial composition. The plots do not show evidence of clustering or segregation based on extreme values on psychiatric measures, which further supports a lack of microbial markers for these psychiatric outcomes in this population. The regression of PC1 and PC2 against each of the psychiatric measures and other participant variables did not indicate any significant linear relationships after FDR correction.

## Discussion

Our results provide evidence for a lack of association in physically and psychologically healthy adult females between microbial markers of gut composition and diversity and a collection of psychiatric measures, including anxiety, depression, eating-related thoughts and behaviors, stress, and personality. No associations between these measures met established significance thresholds in our analysis. Consistent with our results, recent work in large (>1000) Dutch and Flemish cohorts suggests that effect sizes for a wide variety of clinical and lifestyle variables associated with the microbiome in healthy individuals are on the order of 0.01 or smaller, likely below what would be detectable with our sample size of ~100 [56, 57].

Animal models suggest a role for the intestinal microbiota in anxiety, depression, and stress, and many animal studies have documented behavioral changes following manipulation of the intestinal microbiota using prebiotics, probiotics, antibiotics, infection with pathogenic bacteria, or microbial transfer to germ-free (GF) mice (i.e., mice raised in a sterile environment and lacking an intestinal microbiota). Seminal work by Sudo *et al*. (2004) on hypothalamic-pituitary-adrenal (HPA) axis activity showed that GF mice have exaggerated stress response when compared to conventionally raised mice [6]. GF mice also have reduced anxiety-like behavior compared to conventional mice [1, 7–9], which can be reversed via early-life colonization with intestinal bacteria [1, 7]. Anxiety-like behavior can also be increased in mice with pathogenic infection [58–60] or transferred between mice with a characteristic anxiety phenotype and non-anxious GF mice using microbial transfer [61]. Probiotic formulations, such as *Lactobacillus rhamnosus* and *Bifidobacterium infantis*, have been shown in animal models to reduce depressive and anxiety-like behavior at effect sizes similar to antidepressant treatment [5, 10], and prebiotic human milk oligosaccharides may reduce stress-induced anxiety-like behavior and stimulate changes in microbial diversity [62]. Furthermore, using GF mice, it has recently been discovered that the intestinal microbiota is necessary for appropriate and dynamic regulation of myelin-related genes [63]. Altogether, these findings, together with ours, suggest that changes within the intestinal microbiota may be of central importance to the development or maintenance of depression and anxiety—but that the effects are only observed in more extreme expressions of the traits.

The majority of studies investigating the enteric microbe-gut-brain axis has been in animal models with few attempts to translate these findings to a psychologically healthy human population [16]. Significant results generally lack replication. Mixed evidence has emerged from investigations comparing the intestinal microbiotas of individuals with major depressive disorder to healthy controls, with one study failing to find significant between-group differences in microbial diversity or taxonomic composition [64], while the other found increased diversity and significant taxonomic differences at the phylum, family, and genus levels [11]. In patients with acute anorexia nervosa, which is frequently comorbid with depression, work from our laboratory has shown that microbial diversity was both associated with depression and significantly lower than in healthy controls [12]. Composition and diversity of the intestinal microbiota may also be associated with temperament in young children, but how such links may evolve during the development of adult personality is unclear [65].

Prebiotic and probiotic supplementation has emerged in human clinical studies as potential means for altering mood, but connecting post-intervention changes in mood to differences in microbial composition or diversity is lacking. Studies have reported improvement in measures of depression, anxiety, cognitive reactivity, and stress levels in healthy volunteers after placebo-controlled supplementation trials of prebiotic or probiotic formulas [13–15], but these supplements may not be associated with observable compositional changes to the intestinal microbiota. As probiotic supplementation has been found to have little effect on the composition of the intestinal microbiota, changes in mood or behavior may be mediated by the metatranscriptome (i.e., functional activity of enteric microbes) rather than the intestinal microbiota community composition [66, 67].

Nevertheless, clinical trials of novel treatments for depression based on manipulation of the intestinal microbiota are underway. Seminal work that used *Lactobacillus rhamnosus* to demonstrate the central importance of the enteric microbe-gut-brain axis [5] is currently being tested in human trials, which are investigating the effects of probiotic supplementation in healthy volunteers and as an augmentation to antidepressant treatment for individuals with treatment-resistant depression [3]. Another ongoing randomized controlled trial is examining the potential benefit of minocycline (a tetracycline antibiotic) treatment as an adjunctive treatment for individuals with moderate to severe depression [68]. Yet because these intervention studies use microbial strains that are not permanent members of the enteric microbiota, it is possible that any evidence of behavioral change is not due to changes to the intestinal microbiota but rather more direct mechanisms of action.

The majority of the emerging data relating to associations between mood and the composition of the intestinal microbiota has been demonstrated in either rodents or in humans with diagnosable psychiatric disorders such as major depressive disorder [16]. Our goal was to see if this phenomenon translates to subthreshold psychological variation in healthy individuals. This would inform us as to whether the link between enteric microbial communities and behavior is found in all individuals—across the full spectrum of a trait (e.g., depression/anxiety)—or is only detectable in individuals at the extreme end of the distribution such as those suffer from a psychiatric illness. To this end, we designed this study explicitly to investigate the relationship between the composition of the intestinal microbiota and continuous measures of psychological traits in healthy individuals. Our findings are in line with an emerging trend in microbiome research: little taxonomic association with healthy variation. Associations between microbial composition and diversity and a wide range of variables reflecting anthropometry, lifestyle, diet, disease, and medication were recently examined in two large European cohorts (Flemish Gut Flora Project: n = 1106; Dutch Lifelines-DEEP: n = 1135). In addition to the high levels of inter-individual variation reported, findings suggest that, while many factors may be associated with microbial variation, any individual factor would have a *very* modest effect size [56, 57]. These papers did not measure psychiatric variables as broadly or deeply as we did, in which we have demonstrated that effect sizes for healthy cohorts with regard to psychological measures are likely also very small.

These results should be considered in connection with several limitations. With respect to psychopathology, our sample was, on average, healthier than other non-clinical samples of young adults and had less variability on psychiatric measures than would have been expected. As such, restriction of range on psychiatric measures may have played a role in the lack of significant associations with microbial markers. In addition, our analysis focused on taxonomic and diversity measures of 16S rRNA sequencing data, which describes microbial composition but does not account for metabolic activity or functional impact of intestinal bacteria. We may have seen different results with RNA-seq or whole-genome metagenomic shotgun sequencing, longitudinal sampling, or had we analyzed microbial communities from intestinal biopsies. Our sample comprised adult females (age range: 19–50 years), which may differ with respect to these outcome measures from adult males or individuals in younger or older age brackets. Additionally, we excluded participants who had undergone any GI surgery with the exception of appendectomy or cholecystectomy. Recent studies reporting the influence of the appendix and bile acids and salts on the composition of the intestinal microbiota highlight the limitation of including of these surgeries in our study [69, 70]. As we are unable to determine what proportion of our study population had undergone an appendectomy or cholecystectomy, we cannot predict the effect of these surgeries on our results. However, given the relatively low population base rate of these surgeries, and given that our study does not report any significant associations, we believe that excluding subjects with these surgeries would not impact our results or conclusions. Lastly, our study did not take into account that diet or food allergies may influence our observations. Indeed, associations between the enteric microbiota and food intolerance have been reported [71] and changes in diet can alter the composition of this complex community of microorganisms within 24 hours [72].

The enteric microbe-gut-brain axis has attracted considerable attention in recent years, with much focus on the potential role of enteric microorganisms in the development or maintenance of psychiatric illness. Studies involving GF mouse models or clinical populations present extreme cases of psychopathology, which may not reflect microbial mechanisms in a healthy human population.

This study was the first specifically to examine associations between composition and diversity of the intestinal microbiota and psychiatric measures in healthy females, and our results do not reveal associations between the intestinal microbiota and low levels of symptomatology in a healthy population. However, the role of the intestinal microbiota in the pathophysiology of psychiatric illness and evidence of the enteric microbe-gut-brain axis may only be observable in the presence of wider variability of symptom measures and more severe psychopathology.

## Supporting Information

S1 Table Associations between clinical measures and composition and diversity of the intestinal microbiota. We considered associations between 17 different clinical and psychiatric measures from our human cohort (column B) and 232 bacterial taxa (12 phyla, 19 classes, 22 orders, 46 families, and 133 genera) (column A) present in at least 25% of our samples, as well as the Shannon diversity index. We evaluated 3,944 hypotheses [17 measures * (232 taxa + 5 Shannon diversity metrics)] using the non-parametric Kendall’s tau-b test for association (column D), and there were no associations that met established significance thresholds (column C), even after FDR correction (column E).
(TXT)
