# Correction: The Gut-Brain Axis in Healthy Females: Lack of Significant Association between Microbial Composition and Diversity with Psychiatric Measures

**DOI:** 10.1371/journal.pone.0221724

**Published:** 2019-08-22

**Authors:** Susan C. Kleiman, Emily C. Bulik-Sullivan, Elaine M. Glenny, Stephanie C. Zerwas, Eun Young Huh, Matthew C. B. Tsilimigras, Anthony A. Fodor, Cynthia M. Bulik, Ian M. Carroll

The final participant sample (n = 91) is incorrect throughout the article. The correct final participant sample is (n = 87).

The age range of participants is incorrectly listed as 19–50 years throughout the article. The correct age range is 19–51 years.

The mean (SD) age of participants is incorrectly listed as 29.0 (7.9) throughout the article. The correct mean (SD) age of participants is 29.8 (7.7) years.

The minimal effect sizes for >80% and >95% power are incorrectly listed as 0.08 and 0.14, respectively, throughout the article. The correct minimal effect sizes are 0.10 and 0.15.

The total number of 16S rRNA sequence reads is incorrectly listed as 15,391,194. The correct total number of 16S rRNA sequence reads is 15,240,990.

The mean number of 16S rRNA sequence reads is incorrectly listed as 169,134 per sample (range: 47,492–317,380 sequence reads) throughout the article. The correct number of 16S rRNA sequence reads is 175,184 per sample (range: 50,312–328,038 sequence reads).

The number of non-rare taxa reported by the RDP classifier is incorrectly listed as 232 non-rare taxa (12 phyla, 19 classes, 22 orders, 46 families, and 133 genera) throughout the article. The correct number of non-rare taxa reported by the RDP classifier is 227 non-rare taxa (11 phyla, 18 classes, 21 orders, 44 families, and 133 genera).

The percentage values of principal coordinates PC1, PC2, and PC3 are incorrectly listed as 11.5%, 5.16%, and 3.98%, respectively, throughout the article. The correct percentage values are 9.09%, 4.58%, and 3.88%.

There are a number of errors in Fig 1, “Histograms of p-values for associations with psychiatric measures by taxonomic level.” Please see the complete, correct Fig 1 here.

**Fig 1:**
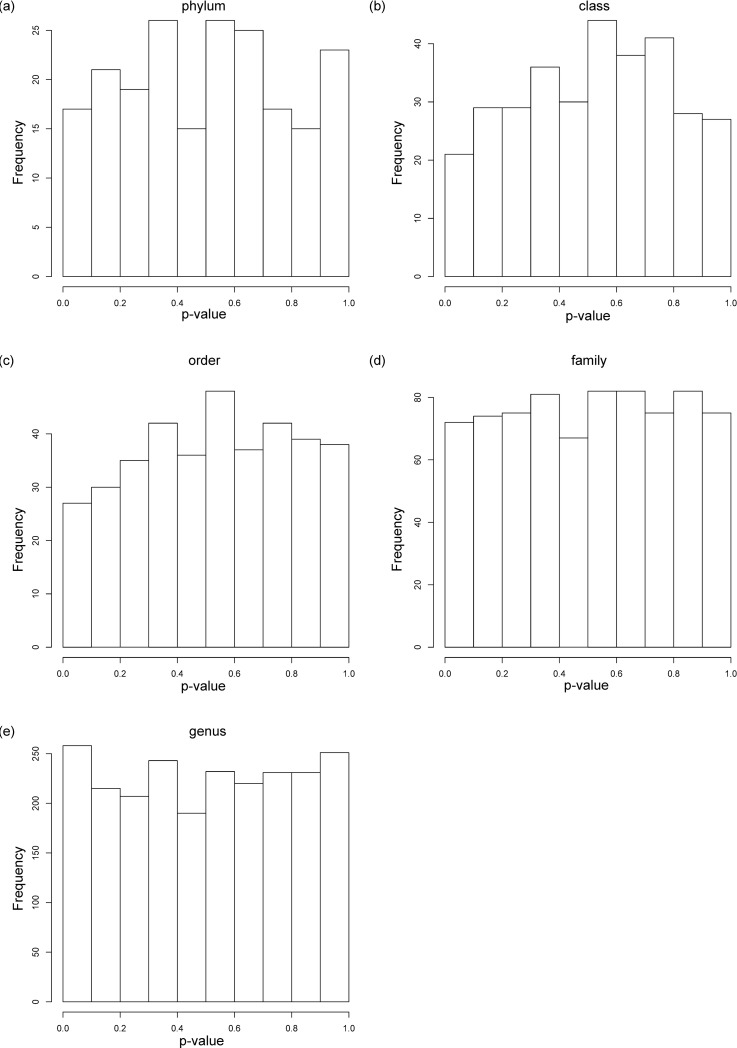
Histograms of p-values for associations with psychiatric measures by taxonomic level. Associations between psychiatric and microbial measures were examined using Kendall’s tau-b correlation coefficient, in conjunction with Benjamini and Hochberg’s False Discovery Rate procedure, using data generated by the RDP classifier. Psychiatric measures included: Beck Anxiety Inventory, Beck Depression Inventory-II, Eating Disorder Examination-Questionnaire, Perceived Stress Scale, and Mini-International Personality Item Pool. P-value frequencies were examined at each taxonomic level: (a) phylum; (b) class; (c) order; (d) family; and (e) genus.

There are a number of errors in Fig 2, “Principal coordinate plots of psychiatric measures by quartile,” and its corresponding caption. Please see the complete, correct Fig 2 and its corresponding caption here.

**Fig 2:**
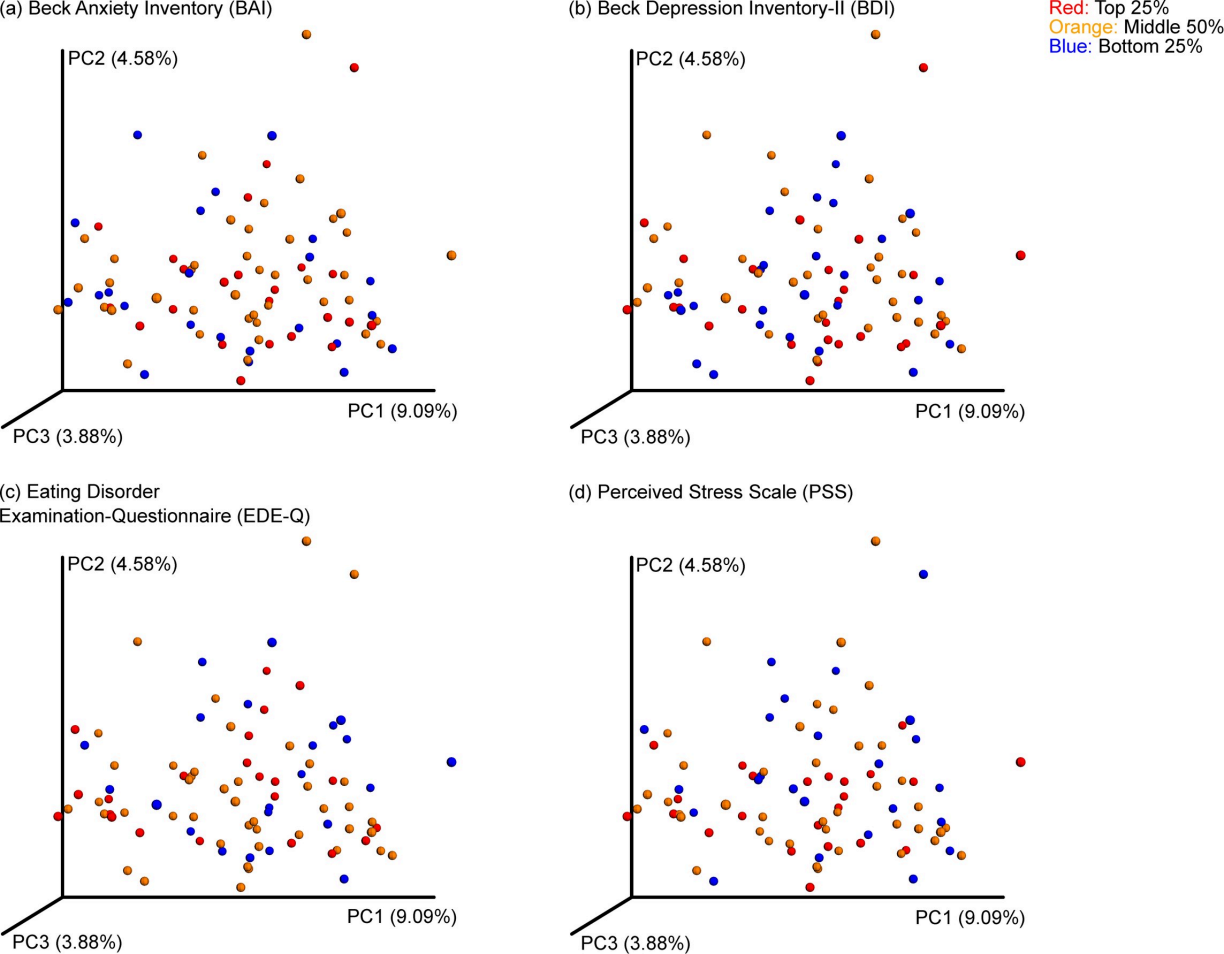
Principal coordinate plots of psychiatric measures by quartile. Principal coordinates were generated using unweighted UniFrac distances from the QIIME pipeline and allocated to quartiles (red: top quartile; orange: middle two quartiles; blue: bottom quartile) based on scores from the (a) Beck Anxiety Inventory; (b) Beck Depression Inventory-II; (c) Eating Disorder Examination-Questionnaire; and (d) Perceived Stress Scale. Plots are based on the first three principal coordinates, which explain 9.09% (PC1), 4.58% (PC2), and 3.88% (PC3) of the variance in microbial composition, and do not cluster by quartile—supporting a lack of association between microbial markers and these psychiatric measures in healthy individuals.

There are a number of errors in Table 1 and its corresponding caption. Please see the complete, correct Table 1 and its corresponding caption here.

**Table 1:** Demographic and clinical characteristics of participants in this study (n = 87) as compared to clinical and normative values.

Metric	Our cohort—mean (SD)	Our cohort—range	Possible range	Clinical/severity thresholds or “case” values—mean (SD)	Normative values in other healthy populations—mean (SD)
Age (years)	29.3 (7.7)	19–51	-----	-----	-----
BMI (kg/m2)	21.7 (1.9)	18.5–25.0	-----	18.5–24.9 = normal or healthy weight [41]	In 2015, 37.4% of NC women fell in this range [48]
BAI	4.8 (4.6)	0–19	0–63 [49]	Scores of <9 = “normal or no anxiety” [49]	6.6 (8.1) [50]
BDI-II	4.7 (5.6)	0–32	0–63 [42]	Scores of <13 = below threshold for depression [42]	8.32 (7.74) [51]
EDE-Q Total	0.6 (0.5)	0–2.7	0–6^a^	3.09 (0.83) [22]	1.52 (1.25) [46]
•Dietary restraint	0.4 (0.6)	0–2.8	0–6 [52]	2.65 (1.48) [22]	1.30 (1.40) [46]
•Eating concern	0.2 (0.2)	0–1.4	0–6 [52]	2.02 (0.95) [22]	0.76 (1.06) [46]
•Shape concern	1.1 (0.8)	0–4.6	0–6 [52]	4.01 (0.98) [22]	2.23 (1.65) [46]
•Weight concern	0.7 (0.8)	0–4.5	0–6 [52]	3.68 (1.08) [22]	1.79 (1.51) [46]
PSS (10-item)	12.3 (6.4)	0–30	0–40	-----^b^ [53]	23.2 [54]
Mini-IPIP:					
•Extraversion	12.7 (3.9)	4–20	4–20 [25]	-----	12.99 (3.83) [55]
•Neuroticism	9.9 (3.5)	4–17	4–20 [25]	-----	11.81 (3.72) [55]
•Agreeableness	16.5 (2.6)	11–20	4–20 [25]	-----	16.57 (2.85) [55]
•Conscientiousness	15.2 (2.9)	8–20	4–20 [25]	-----	13.22 (3.53) [55]
•Intellect/imagination	14.6 (3.1)	7–20	4–20 [25]	-----	15.81 (3.11) [55]

BMI, body mass index; BAI, Beck Anxiety Inventory; BDI, Beck Depression Inventory-II; EDE-Q, Eating Disorder Examination-Questionnaire; PSS, Perceived Stress Scale-10; Mini-IPIP, Mini-International Personality Item Pool.

^a^Average of subcategory values.

^b^The PSS is not used to index diagnostic thresholds. Higher scores reflect higher perceived stress.

-----No suitable clinical data available, e.g. there is no clinical threshold for Agreeableness.

There are a number of errors in S1 Table, “Associations between clinical measures and composition and diversity of the intestinal microbiota,” and its corresponding Supporting Information caption. Please see the complete, correct S1 Table and its corresponding caption here.

## Supporting information

S1 Table Associations between clinical measures and composition and diversity of the intestinal microbiota. We considered associations between 17 different clinical and psychiatric measures from our human cohort (column B) and 227 bacterial taxa (11 phyla, 18 classes, 21 orders, 44 families, and 133 genera) (column A) present in at least 25% of our samples, as well as the Shannon diversity index. We evaluated 3,944 hypotheses [17 measures * (227 taxa + 5 Shannon diversity metrics)] using the non-parametric Kendall’s tau-b test for association (column D), and there were no associations that met established significance thresholds (column C), even after FDR correction (column E).
(DOCX)
