# Supplementary material for: Correction: The Gut-Brain Axis in Healthy Females: Lack of Significant Association between Microbial Composition and Diversity with Psychiatric Measures
Source: PLoS One. 2019 Aug 22;14(8):e0221724. doi: 10.1371/journal.pone.0221724 (PMC6705793; doi:10.1371/journal.pone.0221724)
Supplement: S1 Table — We considered associations between 17 different clinical and psychiatric measures from our human cohort (column B) and 227 bacterial taxa (11 phyla, 18 classes, 21 orders, 44 families, and 133 genera) (column A) present in at least 25% of our samples, as well as the Shannon diversity index. We evaluated 3,944 hypotheses [17 measures * (227 taxa + 5 Shannon diversity metrics)] using the non-parametric Kendall’s tau-b test for association (column D), and there were no associations that met established significance thresholds (column C), even after FDR correction (column E). (DOCX) [file pone.0221724.s001.docx]

**S1 Table. Associations between clinical measures and composition and diversity of the intestinal microbiota.**

| **namesA** | **namesB** | **kendallP** | **rVals** | **adjKendall** |
| --- | --- | --- | --- | --- |
| Candidatus.Saccharibacteria | BDI.II.score | 0.005234 | 0.306123 | 0.841353 |
| Verrucomicrobia | Qualtrics.age | 0.009883 | 0.266991 | 0.841353 |
| Candidatus.Saccharibacteria | Mini.IPIP.Neuroticism | 0.013576 | 0.265481 | 0.841353 |
| Crenarchaeota | BMI | 0.016497 | -0.26674 | 0.841353 |
| Crenarchaeota | Weight.lbs | 0.029475 | -0.23663 | 0.898359 |
| Fusobacteria | BAI.score | 0.043605 | 0.228762 | 0.898359 |
| Candidatus.Saccharibacteria | PSS.10.score | 0.043697 | 0.213689 | 0.898359 |
| Proteobacteria | BAI.score | 0.047208 | 0.215686 | 0.898359 |
| Euryarchaeota | EDEQ4.Total | 0.047719 | 0.204214 | 0.898359 |
| Lentisphaerae | Mini.IPIP.Extraversion | 0.049151 | 0.208919 | 0.898359 |
| Candidatus.Saccharibacteria | BMI | 0.053499 | 0.196378 | 0.898359 |
| Verrucomicrobia | Height.inches | 0.057805 | 0.210092 | 0.898359 |
| Proteobacteria | PSS.10.score | 0.060971 | 0.208903 | 0.898359 |
| Proteobacteria | EDEQ4.Eating | 0.065789 | 0.202748 | 0.898359 |
| Lentisphaerae | EDEQ4.Restraint | 0.066056 | 0.195398 | 0.898359 |
| Lentisphaerae | BAI.score | 0.090655 | -0.1764 | 0.953402 |
| Candidatus.Saccharibacteria | EDEQ4.Eating | 0.090655 | 0.183616 | 0.953402 |
| Cyanobacteria.Chloroplast | PSS.10.score | 0.107799 | -0.17555 | 0.953402 |
| Bacteroidetes | Mini.IPIP.Extraversion | 0.113707 | 0.154464 | 0.953402 |
| Cyanobacteria.Chloroplast | BAI.score | 0.118129 | -0.1691 | 0.953402 |
| Euryarchaeota | EDEQ4.Shape | 0.118629 | 0.165852 | 0.953402 |
| Proteobacteria | Mini.IPIP.Extraversion | 0.122355 | 0.159006 | 0.953402 |
| Candidatus.Saccharibacteria | Weight.lbs | 0.12451 | 0.159785 | 0.953402 |
| Crenarchaeota | Qualtrics.age | 0.127556 | -0.1709 | 0.953402 |
| shannonDiversity (phylum) | BAI.score | 0.140102 | 0.152893 | 0.953402 |
| Candidatus.Saccharibacteria | BAI.score | 0.151318 | 0.15968 | 0.953402 |
| Lentisphaerae | Weight.lbs | 0.151766 | 0.15693 | 0.953402 |
| shannonDiversity (phylum) | BDI.II.score | 0.154653 | 0.160626 | 0.953402 |
| shannonDiversity (phylum) | BMI | 0.162195 | 0.13961 | 0.953402 |
| Cyanobacteria.Chloroplast | EDEQ4.Eating | 0.169791 | -0.14721 | 0.953402 |
| Lentisphaerae | PSS.10.score | 0.170492 | -0.14607 | 0.953402 |
| Proteobacteria | BDI.II.score | 0.172525 | 0.149056 | 0.953402 |
| Firmicutes | Qualtrics.age | 0.178599 | -0.15155 | 0.953402 |
| Verrucomicrobia | Weight.lbs | 0.179748 | 0.150969 | 0.953402 |
| shannonDiversity (phylum) | EDEQ4.Weight | 0.185091 | 0.147328 | 0.953402 |
| shannonDiversity (phylum) | EDEQ4.Restraint | 0.188297 | 0.148104 | 0.953402 |
| Verrucomicrobia | EDEQ4.Total | 0.194144 | 0.146489 | 0.953402 |
| Firmicutes | Mini.IPIP.Neuroticism | 0.199619 | 0.130044 | 0.953402 |
| shannonDiversity (phylum) | Qualtrics.age | 0.200921 | 0.134093 | 0.953402 |
| Crenarchaeota | Mini.IPIP.Imagination | 0.217523 | 0.129514 | 0.953402 |
| Bacteroidetes | Mini.IPIP.Neuroticism | 0.218309 | -0.13808 | 0.953402 |
| Verrucomicrobia | EDEQ4.Weight | 0.227901 | 0.140923 | 0.953402 |
| Lentisphaerae | Mini.IPIP.Conscientiousness | 0.22913 | 0.126769 | 0.953402 |
| Fusobacteria | EDEQ4.Weight | 0.232828 | 0.134847 | 0.953402 |
| shannonDiversity (phylum) | EDEQ4.Total | 0.234683 | 0.153071 | 0.953402 |
| Verrucomicrobia | Mini.IPIP.Conscientiousness | 0.236226 | 0.12441 | 0.953402 |
| Verrucomicrobia | BAI.score | 0.237869 | 0.127337 | 0.953402 |
| Firmicutes | EDEQ4.Restraint | 0.245333 | -0.12596 | 0.953402 |
| Lentisphaerae | Qualtrics.age | 0.250476 | 0.120067 | 0.953402 |
| Actinobacteria | BDI.II.score | 0.256404 | 0.120121 | 0.953402 |
| Euryarchaeota | Weight.lbs | 0.257598 | 0.11302 | 0.953402 |
| Firmicutes | EDEQ4.Total | 0.258604 | -0.12657 | 0.953402 |
| Verrucomicrobia | Mini.IPIP.Agreeableness | 0.270573 | -0.12171 | 0.953402 |
| Euryarchaeota | EDEQ4.Weight | 0.272489 | 0.118788 | 0.953402 |
| Crenarchaeota | EDEQ4.Weight | 0.277122 | -0.12315 | 0.953402 |
| Euryarchaeota | Mini.IPIP.Conscientiousness | 0.278055 | 0.126223 | 0.953402 |
| Fusobacteria | Height.inches | 0.298676 | -0.11076 | 0.953402 |
| Cyanobacteria.Chloroplast | Mini.IPIP.Neuroticism | 0.300173 | -0.11501 | 0.953402 |
| Firmicutes | EDEQ4.Weight | 0.301107 | -0.11448 | 0.953402 |
| Fusobacteria | PSS.10.score | 0.309209 | 0.112355 | 0.953402 |
| Proteobacteria | EDEQ4.Total | 0.314902 | 0.110744 | 0.953402 |
| Firmicutes | BMI | 0.316575 | -0.10273 | 0.953402 |
| Proteobacteria | EDEQ4.Restraint | 0.317219 | 0.098398 | 0.953402 |
| Cyanobacteria.Chloroplast | Height.inches | 0.317935 | 0.097749 | 0.953402 |
| Crenarchaeota | Mini.IPIP.Extraversion | 0.32412 | -0.09418 | 0.953402 |
| Crenarchaeota | BDI.II.score | 0.329023 | 0.103011 | 0.953402 |
| Actinobacteria | BMI | 0.338315 | 0.096748 | 0.953402 |
| Verrucomicrobia | PSS.10.score | 0.339465 | 0.110554 | 0.953402 |
| Cyanobacteria.Chloroplast | Mini.IPIP.Conscientiousness | 0.34296 | 0.096299 | 0.953402 |
| Candidatus.Saccharibacteria | Mini.IPIP.Imagination | 0.343265 | -0.10474 | 0.953402 |
| Euryarchaeota | EDEQ4.Eating | 0.345549 | 0.10136 | 0.953402 |
| Lentisphaerae | Mini.IPIP.Agreeableness | 0.348712 | 0.103475 | 0.953402 |
| Bacteroidetes | Qualtrics.age | 0.350576 | 0.101246 | 0.953402 |
| shannonDiversity (phylum) | Weight.lbs | 0.356251 | 0.110464 | 0.953402 |
| Lentisphaerae | EDEQ4.Total | 0.356926 | 0.092948 | 0.953402 |
| Verrucomicrobia | EDEQ4.Restraint | 0.359899 | 0.100069 | 0.953402 |
| Lentisphaerae | BMI | 0.366167 | 0.097722 | 0.953402 |
| Cyanobacteria.Chloroplast | Mini.IPIP.Imagination | 0.376131 | 0.082452 | 0.953402 |
| Bacteroidetes | EDEQ4.Restraint | 0.380438 | 0.104873 | 0.953402 |
| Bacteroidetes | EDEQ4.Total | 0.382692 | 0.11254 | 0.953402 |
| Verrucomicrobia | Mini.IPIP.Neuroticism | 0.388433 | 0.099902 | 0.953402 |
| Actinobacteria | EDEQ4.Restraint | 0.3931 | 0.091628 | 0.953402 |
| Verrucomicrobia | Mini.IPIP.Imagination | 0.396221 | -0.08788 | 0.953402 |
| Verrucomicrobia | EDEQ4.Shape | 0.401815 | 0.083744 | 0.953402 |
| Euryarchaeota | Qualtrics.age | 0.404668 | 0.090275 | 0.953402 |
| Euryarchaeota | BMI | 0.411755 | 0.099541 | 0.953402 |
| Proteobacteria | Height.inches | 0.413756 | -0.08276 | 0.953402 |
| Lentisphaerae | Mini.IPIP.Neuroticism | 0.428388 | -0.08663 | 0.953402 |
| Cyanobacteria.Chloroplast | Weight.lbs | 0.440587 | 0.085429 | 0.953402 |
| Proteobacteria | EDEQ4.Weight | 0.444104 | 0.080881 | 0.953402 |
| Candidatus.Saccharibacteria | Mini.IPIP.Agreeableness | 0.447773 | 0.075413 | 0.953402 |
| Bacteroidetes | EDEQ4.Weight | 0.453045 | 0.092366 | 0.953402 |
| Fusobacteria | BDI.II.score | 0.455975 | -0.07916 | 0.953402 |
| Candidatus.Saccharibacteria | Qualtrics.age | 0.457282 | -0.08455 | 0.953402 |
| Candidatus.Saccharibacteria | EDEQ4.Weight | 0.476126 | 0.073517 | 0.953402 |
| Bacteroidetes | EDEQ4.Eating | 0.478781 | 0.077323 | 0.953402 |
| Euryarchaeota | BDI.II.score | 0.483307 | 0.082659 | 0.953402 |
| shannonDiversity (phylum) | Mini.IPIP.Agreeableness | 0.496333 | -0.06088 | 0.953402 |
| Actinobacteria | Weight.lbs | 0.5012 | 0.095394 | 0.953402 |
| Cyanobacteria.Chloroplast | Qualtrics.age | 0.518485 | 0.066949 | 0.953402 |
| Firmicutes | BDI.II.score | 0.527282 | -0.06632 | 0.953402 |
| Crenarchaeota | Height.inches | 0.53037 | -0.07567 | 0.953402 |
| Firmicutes | BAI.score | 0.530626 | -0.07484 | 0.953402 |
| Crenarchaeota | Mini.IPIP.Conscientiousness | 0.533716 | 0.075608 | 0.953402 |
| Euryarchaeota | EDEQ4.Restraint | 0.536914 | 0.078272 | 0.953402 |
| shannonDiversity (phylum) | Mini.IPIP.Extraversion | 0.541446 | 0.057907 | 0.953402 |
| Actinobacteria | Mini.IPIP.Imagination | 0.54522 | 0.077747 | 0.953402 |
| Actinobacteria | PSS.10.score | 0.546825 | -0.0681 | 0.953402 |
| Crenarchaeota | EDEQ4.Eating | 0.548084 | 0.068055 | 0.953402 |
| Candidatus.Saccharibacteria | EDEQ4.Restraint | 0.548995 | 0.068614 | 0.953402 |
| Bacteroidetes | EDEQ4.Shape | 0.551379 | 0.064128 | 0.953402 |
| Fusobacteria | EDEQ4.Total | 0.552359 | 0.063269 | 0.953402 |
| Fusobacteria | Mini.IPIP.Extraversion | 0.55391 | 0.070638 | 0.953402 |
| Actinobacteria | BAI.score | 0.55508 | 0.061474 | 0.953402 |
| Bacteroidetes | BMI | 0.559689 | 0.064891 | 0.953402 |
| shannonDiversity (phylum) | EDEQ4.Eating | 0.563258 | 0.065439 | 0.953402 |
| Candidatus.Saccharibacteria | Mini.IPIP.Extraversion | 0.569097 | 0.061133 | 0.953402 |
| Actinobacteria | Mini.IPIP.Neuroticism | 0.570304 | 0.065603 | 0.953402 |
| Fusobacteria | Mini.IPIP.Agreeableness | 0.57067 | 0.064492 | 0.953402 |
| shannonDiversity (phylum) | Mini.IPIP.Neuroticism | 0.575321 | -0.04954 | 0.953402 |
| Firmicutes | PSS.10.score | 0.576546 | 0.069144 | 0.953402 |
| Euryarchaeota | Mini.IPIP.Agreeableness | 0.583031 | -0.05816 | 0.953402 |
| Cyanobacteria.Chloroplast | EDEQ4.Weight | 0.584275 | -0.06937 | 0.953402 |
| Lentisphaerae | Height.inches | 0.598387 | 0.065563 | 0.953402 |
| Bacteroidetes | BAI.score | 0.61074 | 0.044623 | 0.953402 |
| Firmicutes | Mini.IPIP.Extraversion | 0.611712 | -0.06164 | 0.953402 |
| Firmicutes | Weight.lbs | 0.614589 | -0.04452 | 0.953402 |
| Firmicutes | EDEQ4.Shape | 0.617012 | -0.05863 | 0.953402 |
| Bacteroidetes | Mini.IPIP.Conscientiousness | 0.618384 | -0.05834 | 0.953402 |
| Proteobacteria | BMI | 0.620418 | 0.054852 | 0.953402 |
| Proteobacteria | EDEQ4.Shape | 0.627406 | 0.061351 | 0.953402 |
| Firmicutes | EDEQ4.Eating | 0.631002 | -0.05504 | 0.953402 |
| Firmicutes | Mini.IPIP.Agreeableness | 0.641332 | 0.045196 | 0.953402 |
| Bacteroidetes | PSS.10.score | 0.643425 | -0.05142 | 0.953402 |
| Candidatus.Saccharibacteria | Mini.IPIP.Conscientiousness | 0.64501 | 0.047034 | 0.953402 |
| Crenarchaeota | PSS.10.score | 0.655378 | -0.04799 | 0.953402 |
| Cyanobacteria.Chloroplast | Mini.IPIP.Agreeableness | 0.658722 | -0.0414 | 0.953402 |
| shannonDiversity (phylum) | EDEQ4.Shape | 0.659028 | 0.058537 | 0.953402 |
| Fusobacteria | Weight.lbs | 0.667347 | -0.04618 | 0.953402 |
| Lentisphaerae | Mini.IPIP.Imagination | 0.669055 | -0.04894 | 0.953402 |
| Crenarchaeota | Mini.IPIP.Agreeableness | 0.669501 | -0.04548 | 0.953402 |
| Euryarchaeota | Mini.IPIP.Imagination | 0.673109 | -0.03806 | 0.953402 |
| Fusobacteria | EDEQ4.Eating | 0.67529 | 0.044571 | 0.953402 |
| Crenarchaeota | BAI.score | 0.675624 | 0.051008 | 0.953402 |
| Verrucomicrobia | Mini.IPIP.Extraversion | 0.685747 | -0.04951 | 0.953402 |
| Cyanobacteria.Chloroplast | BMI | 0.690793 | 0.038675 | 0.953402 |
| Cyanobacteria.Chloroplast | EDEQ4.Total | 0.69085 | -0.04287 | 0.953402 |
| Cyanobacteria.Chloroplast | Mini.IPIP.Extraversion | 0.692733 | -0.04383 | 0.953402 |
| Actinobacteria | Mini.IPIP.Extraversion | 0.696596 | 0.007293 | 0.953402 |
| Fusobacteria | Mini.IPIP.Conscientiousness | 0.701031 | -0.04567 | 0.953402 |
| Fusobacteria | Mini.IPIP.Neuroticism | 0.716057 | -0.03153 | 0.967388 |
| Crenarchaeota | EDEQ4.Shape | 0.723041 | 0.044358 | 0.970398 |
| Fusobacteria | EDEQ4.Shape | 0.731456 | -0.0399 | 0.972286 |
| shannonDiversity (phylum) | Mini.IPIP.Conscientiousness | 0.737033 | -0.0333 | 0.972286 |
| Bacteroidetes | Height.inches | 0.747647 | -0.04697 | 0.972286 |
| Fusobacteria | EDEQ4.Restraint | 0.750759 | -0.03237 | 0.972286 |
| Lentisphaerae | EDEQ4.Shape | 0.752616 | -0.04148 | 0.972286 |
| Euryarchaeota | Height.inches | 0.755296 | 0.041183 | 0.972286 |
| Proteobacteria | Mini.IPIP.Neuroticism | 0.762476 | 0.036679 | 0.972286 |
| Actinobacteria | Mini.IPIP.Conscientiousness | 0.765027 | 0.046811 | 0.972286 |
| Actinobacteria | Qualtrics.age | 0.768753 | -0.03754 | 0.972286 |
| Crenarchaeota | EDEQ4.Restraint | 0.775054 | 0.025612 | 0.972286 |
| Fusobacteria | BMI | 0.776876 | 0.034117 | 0.972286 |
| Lentisphaerae | EDEQ4.Eating | 0.787167 | 0.030149 | 0.976741 |
| Actinobacteria | EDEQ4.Weight | 0.790972 | -0.03002 | 0.976741 |
| Firmicutes | Height.inches | 0.798588 | 0.037532 | 0.976741 |
| Fusobacteria | Mini.IPIP.Imagination | 0.803152 | -0.03019 | 0.976741 |
| Verrucomicrobia | BDI.II.score | 0.804375 | 0.043169 | 0.976741 |
| Euryarchaeota | BAI.score | 0.824284 | 0.026425 | 0.982442 |
| Actinobacteria | Height.inches | 0.827278 | -0.00769 | 0.982442 |
| Euryarchaeota | PSS.10.score | 0.836463 | 0.033018 | 0.982442 |
| Euryarchaeota | Mini.IPIP.Extraversion | 0.842034 | -0.01135 | 0.982442 |
| Verrucomicrobia | BMI | 0.863105 | 0.018965 | 0.982442 |
| Candidatus.Saccharibacteria | EDEQ4.Shape | 0.867402 | -0.01289 | 0.982442 |
| Proteobacteria | Mini.IPIP.Agreeableness | 0.870776 | -0.02232 | 0.982442 |
| Cyanobacteria.Chloroplast | EDEQ4.Restraint | 0.874195 | 0.022669 | 0.982442 |
| shannonDiversity (phylum) | Mini.IPIP.Imagination | 0.882701 | -0.0185 | 0.982442 |
| Proteobacteria | Mini.IPIP.Imagination | 0.882701 | -0.01367 | 0.982442 |
| Proteobacteria | Weight.lbs | 0.891829 | 0.019161 | 0.982442 |
| Actinobacteria | Mini.IPIP.Agreeableness | 0.894117 | -0.0199 | 0.982442 |
| Lentisphaerae | EDEQ4.Weight | 0.896053 | 0.010962 | 0.982442 |
| Bacteroidetes | BDI.II.score | 0.902902 | -0.00956 | 0.982442 |
| Bacteroidetes | Weight.lbs | 0.903462 | -0.01691 | 0.982442 |
| Candidatus.Saccharibacteria | EDEQ4.Total | 0.903588 | 0.016997 | 0.982442 |
| Firmicutes | Mini.IPIP.Imagination | 0.906039 | -0.01194 | 0.982442 |
| Lentisphaerae | BDI.II.score | 0.914713 | -0.01442 | 0.982442 |
| Cyanobacteria.Chloroplast | BDI.II.score | 0.920181 | 0.004221 | 0.982442 |
| Fusobacteria | Qualtrics.age | 0.921483 | 0.010295 | 0.982442 |
| Proteobacteria | Mini.IPIP.Conscientiousness | 0.9265 | -0.00632 | 0.982442 |
| Firmicutes | Mini.IPIP.Conscientiousness | 0.926501 | 0.010834 | 0.982442 |
| shannonDiversity (phylum) | Height.inches | 0.932215 | 0.003564 | 0.982442 |
| Crenarchaeota | EDEQ4.Total | 0.932503 | -0.00432 | 0.982442 |
| Verrucomicrobia | EDEQ4.Eating | 0.935116 | -0.00765 | 0.982442 |
| Actinobacteria | EDEQ4.Shape | 0.935524 | -0.01877 | 0.982442 |
| Candidatus.Saccharibacteria | Height.inches | 0.939166 | -0.01355 | 0.982442 |
| Bacteroidetes | Mini.IPIP.Agreeableness | 0.964613 | 0.005023 | 0.982442 |
| Euryarchaeota | Mini.IPIP.Neuroticism | 0.967554 | -0.01566 | 0.982442 |
| Crenarchaeota | Mini.IPIP.Neuroticism | 0.973434 | 0.006911 | 0.982442 |
| Cyanobacteria.Chloroplast | EDEQ4.Shape | 0.973495 | 0.011683 | 0.982442 |
| Actinobacteria | EDEQ4.Eating | 0.974022 | 0.003766 | 0.982442 |
| Bacteroidetes | Mini.IPIP.Imagination | 0.982343 | 0.003968 | 0.982442 |
| Proteobacteria | Qualtrics.age | 0.982408 | 0.005023 | 0.982442 |
| shannonDiversity (phylum) | PSS.10.score | 0.982413 | 0.010827 | 0.982442 |
| Actinobacteria | EDEQ4.Total | 0.982442 | -0.00222 | 0.982442 |
| Gammaproteobacteria | PSS.10.score | 0.003972 | 0.311561 | 0.772362 |
| Deltaproteobacteria | EDEQ4.Restraint | 0.007761 | 0.289654 | 0.772362 |
| Verrucomicrobiae | Qualtrics.age | 0.008879 | 0.272871 | 0.772362 |
| Erysipelotrichia | Weight.lbs | 0.009565 | -0.28645 | 0.772362 |
| Thermoprotei | BMI | 0.015845 | -0.2697 | 0.858175 |
| Erysipelotrichia | BMI | 0.015941 | -0.26658 | 0.858175 |
| Gammaproteobacteria | BDI.II.score | 0.030003 | 0.246798 | 0.95947 |
| Thermoprotei | Weight.lbs | 0.03003 | -0.236 | 0.95947 |
| Erysipelotrichia | Mini.IPIP.Extraversion | 0.03537 | -0.23808 | 0.95947 |
| Fusobacteriia | BAI.score | 0.041983 | 0.230294 | 0.95947 |
| Lentisphaeria | Mini.IPIP.Extraversion | 0.049151 | 0.208919 | 0.95947 |
| Alphaproteobacteria | Height.inches | 0.052428 | 0.212338 | 0.95947 |
| Deltaproteobacteria | EDEQ4.Total | 0.057896 | 0.198935 | 0.95947 |
| Verrucomicrobiae | Height.inches | 0.058788 | 0.209311 | 0.95947 |
| Methanobacteria | EDEQ4.Total | 0.06148 | 0.200631 | 0.95947 |
| Lentisphaeria | EDEQ4.Restraint | 0.066056 | 0.195398 | 0.95947 |
| shannonDiversity (class) | EDEQ4.Total | 0.066665 | 0.19992 | 0.95947 |
| Betaproteobacteria | Mini.IPIP.Extraversion | 0.069191 | 0.190884 | 0.95947 |
| Alphaproteobacteria | Mini.IPIP.Agreeableness | 0.082227 | 0.187141 | 0.95947 |
| Lentisphaeria | BAI.score | 0.090655 | -0.1764 | 0.95947 |
| Bacteroidia | Mini.IPIP.Extraversion | 0.099361 | 0.161748 | 0.95947 |
| Bacilli | Mini.IPIP.Neuroticism | 0.103292 | 0.186052 | 0.95947 |
| shannonDiversity (class) | EDEQ4.Weight | 0.107626 | 0.184381 | 0.95947 |
| Chloroplast | PSS.10.score | 0.107799 | -0.17583 | 0.95947 |
| Epsilonproteobacteria | Mini.IPIP.Extraversion | 0.111789 | 0.166029 | 0.95947 |
| Chloroplast | BAI.score | 0.112991 | -0.17143 | 0.95947 |
| Thermoprotei | Qualtrics.age | 0.120345 | -0.17464 | 0.95947 |
| Negativicutes | Mini.IPIP.Extraversion | 0.120586 | 0.173117 | 0.95947 |
| Epsilonproteobacteria | Mini.IPIP.Conscientiousness | 0.12081 | -0.16418 | 0.95947 |
| Alphaproteobacteria | BDI.II.score | 0.12436 | 0.175725 | 0.95947 |
| Alphaproteobacteria | EDEQ4.Restraint | 0.124621 | 0.16698 | 0.95947 |
| shannonDiversity (class) | EDEQ4.Restraint | 0.125755 | 0.167231 | 0.95947 |
| Gammaproteobacteria | Mini.IPIP.Imagination | 0.136168 | -0.15669 | 0.95947 |
| Gammaproteobacteria | Mini.IPIP.Neuroticism | 0.136477 | 0.153594 | 0.95947 |
| Clostridia | EDEQ4.Total | 0.140354 | -0.17281 | 0.95947 |
| Methanobacteria | EDEQ4.Shape | 0.150763 | 0.156638 | 0.95947 |
| Lentisphaeria | Weight.lbs | 0.151766 | 0.15693 | 0.95947 |
| Verrucomicrobiae | Weight.lbs | 0.159223 | 0.156257 | 0.95947 |
| Gammaproteobacteria | BAI.score | 0.163238 | 0.160113 | 0.95947 |
| Betaproteobacteria | Mini.IPIP.Neuroticism | 0.163556 | -0.14392 | 0.95947 |
| Chloroplast | EDEQ4.Eating | 0.167264 | -0.14718 | 0.95947 |
| Lentisphaeria | PSS.10.score | 0.170492 | -0.14607 | 0.95947 |
| Deltaproteobacteria | EDEQ4.Weight | 0.17063 | 0.148853 | 0.95947 |
| Clostridia | EDEQ4.Weight | 0.173017 | -0.15372 | 0.95947 |
| Bacilli | Mini.IPIP.Extraversion | 0.180581 | -0.14564 | 0.95947 |
| Verrucomicrobiae | BAI.score | 0.181795 | 0.141934 | 0.95947 |
| Sphingobacteriia | Mini.IPIP.Agreeableness | 0.184891 | 0.134442 | 0.95947 |
| Bacteroidia | Mini.IPIP.Neuroticism | 0.191977 | -0.15058 | 0.95947 |
| Deltaproteobacteria | BAI.score | 0.192851 | 0.132792 | 0.95947 |
| shannonDiversity (class) | BDI.II.score | 0.194427 | 0.145759 | 0.95947 |
| Verrucomicrobiae | EDEQ4.Total | 0.207043 | 0.145806 | 0.95947 |
| Bacilli | Mini.IPIP.Imagination | 0.212484 | -0.12993 | 0.95947 |
| Verrucomicrobiae | EDEQ4.Weight | 0.216589 | 0.141015 | 0.95947 |
| Methanobacteria | EDEQ4.Restraint | 0.219102 | 0.136278 | 0.95947 |
| Clostridia | EDEQ4.Restraint | 0.221213 | -0.13915 | 0.95947 |
| Verrucomicrobiae | Mini.IPIP.Conscientiousness | 0.224751 | 0.127288 | 0.95947 |
| Gammaproteobacteria | EDEQ4.Shape | 0.224957 | 0.136233 | 0.95947 |
| Betaproteobacteria | Height.inches | 0.226544 | -0.12473 | 0.95947 |
| Fusobacteriia | EDEQ4.Weight | 0.226617 | 0.136059 | 0.95947 |
| Lentisphaeria | Mini.IPIP.Conscientiousness | 0.22913 | 0.126769 | 0.95947 |
| Thermoprotei | Mini.IPIP.Imagination | 0.237484 | 0.125293 | 0.95947 |
| Clostridia | Mini.IPIP.Neuroticism | 0.247141 | 0.117464 | 0.95947 |
| Clostridia | Qualtrics.age | 0.248509 | -0.12961 | 0.95947 |
| Erysipelotrichia | BAI.score | 0.249806 | 0.11373 | 0.95947 |
| Lentisphaeria | Qualtrics.age | 0.250476 | 0.120067 | 0.95947 |
| Clostridia | Height.inches | 0.25623 | 0.126715 | 0.95947 |
| Deltaproteobacteria | EDEQ4.Shape | 0.25735 | 0.121836 | 0.95947 |
| Erysipelotrichia | Height.inches | 0.262478 | -0.12846 | 0.95947 |
| Verrucomicrobiae | Mini.IPIP.Agreeableness | 0.264195 | -0.12346 | 0.95947 |
| Betaproteobacteria | BMI | 0.266319 | 0.121566 | 0.95947 |
| Deltaproteobacteria | Weight.lbs | 0.266956 | 0.119973 | 0.95947 |
| Thermoprotei | EDEQ4.Weight | 0.270549 | -0.12407 | 0.95947 |
| Actinobacteria | BDI.II.score | 0.27222 | 0.117283 | 0.95947 |
| Bacteroidia | Qualtrics.age | 0.273439 | 0.113866 | 0.95947 |
| Methanobacteria | EDEQ4.Eating | 0.283536 | 0.11569 | 0.95947 |
| Bacilli | Height.inches | 0.28517 | -0.12155 | 0.95947 |
| shannonDiversity (class) | EDEQ4.Shape | 0.289594 | 0.118317 | 0.95947 |
| Fusobacteriia | Height.inches | 0.29136 | -0.11154 | 0.95947 |
| Gammaproteobacteria | Height.inches | 0.291889 | -0.11537 | 0.95947 |
| shannonDiversity (class) | Qualtrics.age | 0.300027 | 0.108067 | 0.95947 |
| Chloroplast | Mini.IPIP.Neuroticism | 0.300173 | -0.11607 | 0.95947 |
| Alphaproteobacteria | EDEQ4.Weight | 0.30093 | 0.109335 | 0.95947 |
| Epsilonproteobacteria | BMI | 0.302312 | 0.113284 | 0.95947 |
| shannonDiversity (class) | Height.inches | 0.305646 | -0.10659 | 0.95947 |
| Clostridia | EDEQ4.Shape | 0.31016 | -0.11001 | 0.95947 |
| Fusobacteriia | PSS.10.score | 0.312974 | 0.112306 | 0.95947 |
| Bacilli | Qualtrics.age | 0.317492 | -0.11702 | 0.95947 |
| Chloroplast | Height.inches | 0.317935 | 0.097943 | 0.95947 |
| Epsilonproteobacteria | Qualtrics.age | 0.324412 | -0.1143 | 0.95947 |
| Actinobacteria | BMI | 0.338315 | 0.094997 | 0.95947 |
| Chloroplast | Mini.IPIP.Conscientiousness | 0.339203 | 0.09825 | 0.95947 |
| Erysipelotrichia | BDI.II.score | 0.342067 | 0.111388 | 0.95947 |
| Thermoprotei | Mini.IPIP.Extraversion | 0.342571 | -0.09124 | 0.95947 |
| Thermoprotei | BDI.II.score | 0.343948 | 0.099767 | 0.95947 |
| Bacteroidia | EDEQ4.Total | 0.343993 | 0.120435 | 0.95947 |
| Methanobacteria | Weight.lbs | 0.34402 | 0.097223 | 0.95947 |
| Lentisphaeria | Mini.IPIP.Agreeableness | 0.348712 | 0.103475 | 0.95947 |
| Verrucomicrobiae | PSS.10.score | 0.350728 | 0.110783 | 0.95947 |
| Actinobacteria | EDEQ4.Restraint | 0.351882 | 0.098838 | 0.95947 |
| Deltaproteobacteria | Mini.IPIP.Imagination | 0.352558 | -0.1042 | 0.95947 |
| Alphaproteobacteria | Weight.lbs | 0.355314 | 0.098678 | 0.95947 |
| Bacteroidia | EDEQ4.Restraint | 0.355876 | 0.110087 | 0.95947 |
| Lentisphaeria | EDEQ4.Total | 0.356926 | 0.092948 | 0.95947 |
| Methanobacteria | BDI.II.score | 0.362658 | 0.106014 | 0.95947 |
| Bacilli | Mini.IPIP.Agreeableness | 0.36309 | -0.1004 | 0.95947 |
| Lentisphaeria | BMI | 0.366167 | 0.097722 | 0.95947 |
| Chloroplast | Mini.IPIP.Imagination | 0.37215 | 0.082885 | 0.95947 |
| Verrucomicrobiae | EDEQ4.Shape | 0.373539 | 0.087655 | 0.95947 |
| Verrucomicrobiae | EDEQ4.Restraint | 0.376274 | 0.098904 | 0.95947 |
| Verrucomicrobiae | Mini.IPIP.Neuroticism | 0.376384 | 0.103006 | 0.95947 |
| Erysipelotrichia | EDEQ4.Eating | 0.37928 | 0.094619 | 0.95947 |
| shannonDiversity (class) | EDEQ4.Eating | 0.383709 | 0.098648 | 0.95947 |
| shannonDiversity (class) | BAI.score | 0.388074 | 0.092023 | 0.95947 |
| Betaproteobacteria | Mini.IPIP.Imagination | 0.396221 | 0.092939 | 0.95947 |
| Betaproteobacteria | Mini.IPIP.Conscientiousness | 0.398112 | 0.08935 | 0.95947 |
| Epsilonproteobacteria | BAI.score | 0.400482 | 0.092177 | 0.95947 |
| Bacteroidia | EDEQ4.Weight | 0.40514 | 0.099551 | 0.95947 |
| Sphingobacteriia | BMI | 0.405654 | 0.082136 | 0.95947 |
| Methanobacteria | Mini.IPIP.Conscientiousness | 0.410927 | 0.094927 | 0.95947 |
| Methanobacteria | Qualtrics.age | 0.412782 | 0.087622 | 0.95947 |
| Negativicutes | Qualtrics.age | 0.414575 | -0.08121 | 0.95947 |
| Betaproteobacteria | EDEQ4.Eating | 0.41559 | 0.087481 | 0.95947 |
| Deltaproteobacteria | Mini.IPIP.Extraversion | 0.416199 | -0.08414 | 0.95947 |
| Erysipelotrichia | Mini.IPIP.Conscientiousness | 0.427581 | -0.07993 | 0.95947 |
| Lentisphaeria | Mini.IPIP.Neuroticism | 0.428388 | -0.08663 | 0.95947 |
| Erysipelotrichia | Mini.IPIP.Imagination | 0.429895 | -0.09066 | 0.95947 |
| Betaproteobacteria | Mini.IPIP.Agreeableness | 0.433163 | 0.076637 | 0.95947 |
| Gammaproteobacteria | EDEQ4.Eating | 0.434489 | 0.084734 | 0.95947 |
| Epsilonproteobacteria | EDEQ4.Restraint | 0.434742 | -0.08092 | 0.95947 |
| Actinobacteria | Weight.lbs | 0.438035 | 0.101349 | 0.95947 |
| Chloroplast | Weight.lbs | 0.440587 | 0.086245 | 0.95947 |
| Gammaproteobacteria | Mini.IPIP.Agreeableness | 0.446294 | -0.0911 | 0.95947 |
| Sphingobacteriia | EDEQ4.Restraint | 0.452216 | 0.081718 | 0.95947 |
| Bacilli | Weight.lbs | 0.4556 | -0.08417 | 0.95947 |
| Verrucomicrobiae | Mini.IPIP.Imagination | 0.456207 | -0.07758 | 0.95947 |
| Deltaproteobacteria | PSS.10.score | 0.462421 | 0.07525 | 0.95947 |
| Fusobacteriia | BDI.II.score | 0.465617 | -0.07825 | 0.95947 |
| Betaproteobacteria | BDI.II.score | 0.47098 | -0.08303 | 0.95947 |
| Betaproteobacteria | EDEQ4.Restraint | 0.474288 | 0.072051 | 0.95947 |
| Gammaproteobacteria | Qualtrics.age | 0.480426 | -0.07159 | 0.95947 |
| shannonDiversity (class) | Mini.IPIP.Agreeableness | 0.482396 | -0.07905 | 0.95947 |
| Bacilli | EDEQ4.Total | 0.485871 | -0.0737 | 0.95947 |
| Deltaproteobacteria | EDEQ4.Eating | 0.488907 | 0.073664 | 0.95947 |
| Actinobacteria | PSS.10.score | 0.494385 | -0.0777 | 0.95947 |
| Gammaproteobacteria | Weight.lbs | 0.496534 | -0.06986 | 0.95947 |
| Epsilonproteobacteria | BDI.II.score | 0.504147 | 0.068885 | 0.95947 |
| Bacteroidia | EDEQ4.Eating | 0.514868 | 0.072971 | 0.95947 |
| Epsilonproteobacteria | PSS.10.score | 0.515068 | 0.074189 | 0.95947 |
| Alphaproteobacteria | Mini.IPIP.Extraversion | 0.516058 | 0.06311 | 0.95947 |
| Deltaproteobacteria | BMI | 0.516073 | 0.080325 | 0.95947 |
| Deltaproteobacteria | Height.inches | 0.517468 | 0.070542 | 0.95947 |
| Alphaproteobacteria | EDEQ4.Shape | 0.51865 | -0.07645 | 0.95947 |
| Alphaproteobacteria | EDEQ4.Total | 0.51967 | 0.065125 | 0.95947 |
| Bacilli | EDEQ4.Eating | 0.520136 | -0.06928 | 0.95947 |
| Clostridia | EDEQ4.Eating | 0.520136 | -0.06931 | 0.95947 |
| Sphingobacteriia | EDEQ4.Total | 0.520255 | 0.087714 | 0.95947 |
| Bacilli | EDEQ4.Shape | 0.52229 | -0.06674 | 0.95947 |
| Bacteroidia | EDEQ4.Shape | 0.52229 | 0.067043 | 0.95947 |
| Methanobacteria | EDEQ4.Weight | 0.523406 | 0.071584 | 0.95947 |
| Alphaproteobacteria | Mini.IPIP.Imagination | 0.524743 | 0.068415 | 0.95947 |
| Methanobacteria | BMI | 0.528087 | 0.072346 | 0.95947 |
| Chloroplast | Qualtrics.age | 0.52809 | 0.067105 | 0.95947 |
| Clostridia | PSS.10.score | 0.532254 | 0.065876 | 0.95947 |
| Epsilonproteobacteria | Mini.IPIP.Imagination | 0.533315 | -0.06841 | 0.95947 |
| Gammaproteobacteria | Mini.IPIP.Extraversion | 0.541446 | 0.068764 | 0.95947 |
| Fusobacteriia | EDEQ4.Total | 0.541881 | 0.064764 | 0.95947 |
| Thermoprotei | Mini.IPIP.Conscientiousness | 0.543502 | 0.075948 | 0.95947 |
| Sphingobacteriia | Mini.IPIP.Neuroticism | 0.552468 | 0.065442 | 0.95947 |
| Fusobacteriia | Mini.IPIP.Extraversion | 0.55391 | 0.070903 | 0.95947 |
| Epsilonproteobacteria | EDEQ4.Eating | 0.554674 | 0.064358 | 0.95947 |
| Sphingobacteriia | EDEQ4.Shape | 0.55587 | 0.068209 | 0.95947 |
| Negativicutes | EDEQ4.Weight | 0.557813 | 0.069089 | 0.95947 |
| Bacteroidia | BMI | 0.559689 | 0.065255 | 0.95947 |
| Clostridia | BMI | 0.559689 | -0.06546 | 0.95947 |
| Thermoprotei | Height.inches | 0.559967 | -0.07215 | 0.95947 |
| Actinobacteria | Mini.IPIP.Imagination | 0.560024 | 0.076162 | 0.95947 |
| Bacteroidia | Mini.IPIP.Conscientiousness | 0.562372 | -0.0644 | 0.95947 |
| Thermoprotei | EDEQ4.Eating | 0.564536 | 0.065116 | 0.95947 |
| Alphaproteobacteria | Qualtrics.age | 0.56637 | 0.065139 | 0.95947 |
| Chloroplast | EDEQ4.Weight | 0.568924 | -0.07128 | 0.95947 |
| Fusobacteriia | Mini.IPIP.Agreeableness | 0.57067 | 0.064039 | 0.95947 |
| Negativicutes | Mini.IPIP.Agreeableness | 0.584259 | 0.057979 | 0.95947 |
| Negativicutes | Mini.IPIP.Conscientiousness | 0.587528 | 0.060743 | 0.95947 |
| Sphingobacteriia | Qualtrics.age | 0.588827 | 0.058537 | 0.95947 |
| Methanobacteria | BAI.score | 0.588877 | 0.052058 | 0.95947 |
| Alphaproteobacteria | BMI | 0.590212 | -0.05528 | 0.95947 |
| Bacteroidia | PSS.10.score | 0.591686 | -0.06051 | 0.95947 |
| Bacilli | EDEQ4.Weight | 0.593459 | -0.06055 | 0.95947 |
| Lentisphaeria | Height.inches | 0.598387 | 0.065563 | 0.95947 |
| Betaproteobacteria | PSS.10.score | 0.601881 | 0.049727 | 0.95947 |
| Clostridia | Mini.IPIP.Agreeableness | 0.604739 | 0.055417 | 0.95947 |
| Gammaproteobacteria | EDEQ4.Restraint | 0.606216 | -0.04891 | 0.95947 |
| Negativicutes | EDEQ4.Total | 0.612742 | 0.058855 | 0.95947 |
| shannonDiversity (class) | Mini.IPIP.Conscientiousness | 0.613192 | -0.05104 | 0.95947 |
| Alphaproteobacteria | PSS.10.score | 0.621284 | 0.053511 | 0.95947 |
| Methanobacteria | Mini.IPIP.Extraversion | 0.623887 | 0.047806 | 0.95947 |
| Actinobacteria | BAI.score | 0.626339 | 0.049297 | 0.95947 |
| Actinobacteria | Mini.IPIP.Neuroticism | 0.6266 | 0.057473 | 0.95947 |
| Bacilli | Mini.IPIP.Conscientiousness | 0.628824 | 0.053099 | 0.95947 |
| Bacilli | EDEQ4.Restraint | 0.633352 | 0.053783 | 0.95947 |
| Thermoprotei | PSS.10.score | 0.634209 | -0.05202 | 0.95947 |
| Thermoprotei | Mini.IPIP.Agreeableness | 0.637375 | -0.05067 | 0.95947 |
| Erysipelotrichia | Mini.IPIP.Neuroticism | 0.642359 | 0.046989 | 0.95947 |
| Negativicutes | BMI | 0.646541 | 0.052363 | 0.95947 |
| Methanobacteria | Height.inches | 0.651591 | 0.049669 | 0.95947 |
| Betaproteobacteria | BAI.score | 0.65271 | 0.036597 | 0.95947 |
| shannonDiversity (class) | BMI | 0.657116 | 0.048679 | 0.95947 |
| Betaproteobacteria | Qualtrics.age | 0.659205 | 0.055714 | 0.95947 |
| Fusobacteriia | EDEQ4.Eating | 0.66258 | 0.046151 | 0.95947 |
| Chloroplast | Mini.IPIP.Agreeableness | 0.664103 | -0.04007 | 0.95947 |
| Fusobacteriia | Weight.lbs | 0.667347 | -0.04547 | 0.95947 |
| Sphingobacteriia | Height.inches | 0.668346 | -0.04788 | 0.95947 |
| Epsilonproteobacteria | Height.inches | 0.668656 | -0.04317 | 0.95947 |
| Negativicutes | Mini.IPIP.Imagination | 0.668736 | 0.049754 | 0.95947 |
| Lentisphaeria | Mini.IPIP.Imagination | 0.669055 | -0.04894 | 0.95947 |
| Epsilonproteobacteria | Mini.IPIP.Neuroticism | 0.679214 | 0.046351 | 0.95947 |
| Chloroplast | EDEQ4.Total | 0.680023 | -0.04329 | 0.95947 |
| Erysipelotrichia | EDEQ4.Restraint | 0.683443 | -0.04377 | 0.95947 |
| Sphingobacteriia | Weight.lbs | 0.683793 | 0.047295 | 0.95947 |
| Chloroplast | BMI | 0.690793 | 0.038712 | 0.95947 |
| Betaproteobacteria | EDEQ4.Shape | 0.691272 | -0.04199 | 0.95947 |
| Chloroplast | Mini.IPIP.Extraversion | 0.692733 | -0.04352 | 0.95947 |
| Sphingobacteriia | EDEQ4.Weight | 0.692959 | 0.060244 | 0.95947 |
| Betaproteobacteria | Weight.lbs | 0.694119 | 0.049556 | 0.95947 |
| Negativicutes | Weight.lbs | 0.694119 | 0.040834 | 0.95947 |
| Thermoprotei | BAI.score | 0.697411 | 0.049279 | 0.95947 |
| Clostridia | BDI.II.score | 0.697891 | -0.04753 | 0.95947 |
| Fusobacteriia | Mini.IPIP.Conscientiousness | 0.701031 | -0.04557 | 0.95947 |
| Actinobacteria | Mini.IPIP.Extraversion | 0.702044 | 0.006845 | 0.95947 |
| Deltaproteobacteria | Mini.IPIP.Conscientiousness | 0.706609 | -0.04274 | 0.95947 |
| Bacteroidia | BAI.score | 0.706746 | 0.030513 | 0.95947 |
| shannonDiversity (class) | Mini.IPIP.Extraversion | 0.707508 | -0.04093 | 0.95947 |
| Sphingobacteriia | EDEQ4.Eating | 0.712148 | 0.038753 | 0.95947 |
| Negativicutes | EDEQ4.Eating | 0.714109 | 0.026283 | 0.95947 |
| Gammaproteobacteria | EDEQ4.Weight | 0.717271 | -0.03855 | 0.95947 |
| Gammaproteobacteria | Mini.IPIP.Conscientiousness | 0.7204 | -0.02968 | 0.95947 |
| Bacilli | BMI | 0.727453 | -0.04291 | 0.95947 |
| Fusobacteriia | Mini.IPIP.Neuroticism | 0.727901 | -0.0303 | 0.95947 |
| Verrucomicrobiae | Mini.IPIP.Extraversion | 0.72951 | -0.04197 | 0.95947 |
| Clostridia | Weight.lbs | 0.73249 | 0.034989 | 0.95947 |
| Negativicutes | BAI.score | 0.73435 | 0.045631 | 0.95947 |
| shannonDiversity (class) | PSS.10.score | 0.735362 | 0.039657 | 0.95947 |
| Negativicutes | PSS.10.score | 0.735362 | -0.03481 | 0.95947 |
| Fusobacteriia | EDEQ4.Shape | 0.743357 | -0.03899 | 0.95947 |
| Thermoprotei | EDEQ4.Shape | 0.750879 | 0.040956 | 0.95947 |
| Thermoprotei | EDEQ4.Restraint | 0.751499 | 0.02797 | 0.95947 |
| Sphingobacteriia | Mini.IPIP.Imagination | 0.75212 | -0.05199 | 0.95947 |
| Lentisphaeria | EDEQ4.Shape | 0.752616 | -0.04148 | 0.95947 |
| Epsilonproteobacteria | Mini.IPIP.Agreeableness | 0.753259 | -0.03126 | 0.95947 |
| Erysipelotrichia | EDEQ4.Weight | 0.756679 | 0.040111 | 0.95947 |
| Negativicutes | Mini.IPIP.Neuroticism | 0.756864 | -0.0349 | 0.95947 |
| Bacilli | PSS.10.score | 0.757618 | 0.025443 | 0.95947 |
| Epsilonproteobacteria | Weight.lbs | 0.761196 | 0.040439 | 0.95947 |
| Fusobacteriia | EDEQ4.Restraint | 0.763309 | -0.03117 | 0.95947 |
| Fusobacteriia | BMI | 0.764836 | 0.035524 | 0.95947 |
| Actinobacteria | Mini.IPIP.Conscientiousness | 0.770665 | 0.046078 | 0.95947 |
| Erysipelotrichia | PSS.10.score | 0.774444 | 0.031514 | 0.95947 |
| Bacteroidia | Height.inches | 0.775827 | -0.03987 | 0.95947 |
| Methanobacteria | Mini.IPIP.Neuroticism | 0.781374 | -0.03563 | 0.95947 |
| Verrucomicrobiae | BMI | 0.783206 | 0.027627 | 0.95947 |
| shannonDiversity (class) | Mini.IPIP.Imagination | 0.784884 | -0.0351 | 0.95947 |
| Lentisphaeria | EDEQ4.Eating | 0.787167 | 0.030149 | 0.95947 |
| Methanobacteria | Mini.IPIP.Agreeableness | 0.789401 | 0.024959 | 0.95947 |
| Bacilli | BDI.II.score | 0.792954 | 0.029367 | 0.95947 |
| Sphingobacteriia | Mini.IPIP.Conscientiousness | 0.794711 | -0.03819 | 0.95947 |
| Clostridia | Mini.IPIP.Imagination | 0.796248 | -0.01938 | 0.95947 |
| Bacilli | BAI.score | 0.796255 | 0.032116 | 0.95947 |
| Negativicutes | Height.inches | 0.798588 | 0.036164 | 0.95947 |
| Actinobacteria | EDEQ4.Weight | 0.802498 | -0.0283 | 0.95947 |
| Fusobacteriia | Mini.IPIP.Imagination | 0.803152 | -0.03006 | 0.95947 |
| Methanobacteria | PSS.10.score | 0.805104 | 0.027781 | 0.95947 |
| Clostridia | Mini.IPIP.Conscientiousness | 0.810449 | 0.022044 | 0.95947 |
| Erysipelotrichia | Mini.IPIP.Agreeableness | 0.812955 | -0.02961 | 0.95947 |
| Verrucomicrobiae | BDI.II.score | 0.815838 | 0.045106 | 0.95947 |
| shannonDiversity (class) | Weight.lbs | 0.816886 | -0.01918 | 0.95947 |
| Sphingobacteriia | PSS.10.score | 0.830027 | -0.02908 | 0.967731 |
| Actinobacteria | Qualtrics.age | 0.831206 | -0.03026 | 0.967731 |
| Negativicutes | BDI.II.score | 0.833106 | 0.030808 | 0.967731 |
| Sphingobacteriia | Mini.IPIP.Extraversion | 0.841398 | 0.015021 | 0.967731 |
| Deltaproteobacteria | Mini.IPIP.Neuroticism | 0.842223 | 0.021299 | 0.967731 |
| Deltaproteobacteria | Qualtrics.age | 0.848427 | -0.01828 | 0.967731 |
| Bacteroidia | BDI.II.score | 0.850454 | -0.01836 | 0.967731 |
| Gammaproteobacteria | EDEQ4.Total | 0.85449 | 0.029655 | 0.967731 |
| Deltaproteobacteria | BDI.II.score | 0.864953 | -0.01968 | 0.967731 |
| Clostridia | BAI.score | 0.865268 | -0.0207 | 0.967731 |
| Alphaproteobacteria | Mini.IPIP.Conscientiousness | 0.867651 | 0.024729 | 0.967731 |
| Epsilonproteobacteria | EDEQ4.Weight | 0.870448 | 0.019703 | 0.967731 |
| Negativicutes | EDEQ4.Shape | 0.871468 | -0.01487 | 0.967731 |
| Betaproteobacteria | EDEQ4.Weight | 0.87247 | -0.02356 | 0.967731 |
| Epsilonproteobacteria | EDEQ4.Shape | 0.8826 | -0.01995 | 0.967731 |
| Alphaproteobacteria | EDEQ4.Eating | 0.883785 | 0.014392 | 0.967731 |
| Chloroplast | EDEQ4.Restraint | 0.886381 | 0.021797 | 0.967731 |
| Alphaproteobacteria | BAI.score | 0.888597 | -0.02025 | 0.967731 |
| Clostridia | Mini.IPIP.Extraversion | 0.888832 | -0.02107 | 0.967731 |
| Lentisphaeria | EDEQ4.Weight | 0.896053 | 0.010962 | 0.967731 |
| Verrucomicrobiae | EDEQ4.Eating | 0.896364 | -0.01287 | 0.967731 |
| Sphingobacteriia | BAI.score | 0.905352 | -0.02329 | 0.967731 |
| Actinobacteria | Mini.IPIP.Agreeableness | 0.905824 | -0.0198 | 0.967731 |
| Actinobacteria | Height.inches | 0.914593 | 0.002425 | 0.967731 |
| Deltaproteobacteria | Mini.IPIP.Agreeableness | 0.914608 | 0.009092 | 0.967731 |
| Lentisphaeria | BDI.II.score | 0.914713 | -0.01442 | 0.967731 |
| Alphaproteobacteria | Mini.IPIP.Neuroticism | 0.915641 | 0.009994 | 0.967731 |
| Actinobacteria | EDEQ4.Shape | 0.917995 | -0.01782 | 0.967731 |
| Negativicutes | EDEQ4.Restraint | 0.920329 | 0.013656 | 0.967731 |
| Fusobacteriia | Qualtrics.age | 0.921483 | 0.010325 | 0.967731 |
| shannonDiversity (class) | Mini.IPIP.Neuroticism | 0.923656 | -0.00765 | 0.967731 |
| Methanobacteria | Mini.IPIP.Imagination | 0.925242 | -0.00561 | 0.967731 |
| Chloroplast | BDI.II.score | 0.926075 | 0.003733 | 0.967731 |
| Thermoprotei | EDEQ4.Total | 0.92665 | -0.00435 | 0.967731 |
| Sphingobacteriia | BDI.II.score | 0.928782 | 0.003764 | 0.967731 |
| Bacteroidia | Weight.lbs | 0.944326 | -0.01231 | 0.974515 |
| Betaproteobacteria | EDEQ4.Total | 0.947361 | -0.00388 | 0.974515 |
| Erysipelotrichia | EDEQ4.Total | 0.947361 | -0.00264 | 0.974515 |
| Actinobacteria | EDEQ4.Total | 0.947361 | 0.00351 | 0.974515 |
| Bacteroidia | Mini.IPIP.Agreeableness | 0.95872 | 0.004564 | 0.982542 |
| Thermoprotei | Mini.IPIP.Neuroticism | 0.967533 | 0.005992 | 0.982542 |
| Erysipelotrichia | EDEQ4.Shape | 0.970667 | 0.011777 | 0.982542 |
| Chloroplast | EDEQ4.Shape | 0.973495 | 0.01172 | 0.982542 |
| Gammaproteobacteria | BMI | 0.973661 | 0.003146 | 0.982542 |
| Actinobacteria | EDEQ4.Eating | 0.974022 | 0.003766 | 0.982542 |
| Bacteroidia | Mini.IPIP.Imagination | 0.976458 | 0.00274 | 0.982542 |
| Epsilonproteobacteria | EDEQ4.Total | 0.996543 | 0.002978 | 0.999637 |
| Erysipelotrichia | Qualtrics.age | 1 | -0.00204 | 1 |
| Desulfovibrionales | EDEQ4.Restraint | 0.007413 | 0.291239 | 0.991459 |
| Verrucomicrobiales | Qualtrics.age | 0.00869 | 0.273136 | 0.991459 |
| Erysipelotrichales | Weight.lbs | 0.009771 | -0.28553 | 0.991459 |
| Erysipelotrichales | BMI | 0.016265 | -0.26551 | 0.991459 |
| Pasteurellales | Qualtrics.age | 0.020774 | -0.23599 | 0.991459 |
| Enterobacteriales | PSS.10.score | 0.025012 | 0.231006 | 0.991459 |
| Actinomycetales | BDI.II.score | 0.027834 | 0.237431 | 0.991459 |
| Desulfurococcales | Mini.IPIP.Imagination | 0.03437 | 0.228301 | 0.991459 |
| Erysipelotrichales | Mini.IPIP.Extraversion | 0.036674 | -0.23721 | 0.991459 |
| Fusobacteriales | BAI.score | 0.041983 | 0.230294 | 0.991459 |
| Victivallales | Mini.IPIP.Extraversion | 0.049151 | 0.208919 | 0.991459 |
| Desulfovibrionales | EDEQ4.Total | 0.055984 | 0.20116 | 0.991459 |
| Verrucomicrobiales | Height.inches | 0.059784 | 0.209054 | 0.991459 |
| Methanobacteriales | EDEQ4.Total | 0.06045 | 0.201401 | 0.991459 |
| Bacillales | EDEQ4.Restraint | 0.06098 | -0.20431 | 0.991459 |
| Pasteurellales | Mini.IPIP.Agreeableness | 0.065008 | -0.19983 | 0.991459 |
| Victivallales | EDEQ4.Restraint | 0.066056 | 0.195398 | 0.991459 |
| Rhodospirillales | Mini.IPIP.Agreeableness | 0.066696 | 0.199147 | 0.991459 |
| Burkholderiales | Mini.IPIP.Extraversion | 0.071473 | 0.188334 | 0.991459 |
| Bacillales | Height.inches | 0.075842 | -0.20068 | 0.991459 |
| Pasteurellales | PSS.10.score | 0.077763 | 0.195679 | 0.991459 |
| Rhodospirillales | EDEQ4.Restraint | 0.087129 | 0.182068 | 0.991459 |
| Victivallales | BAI.score | 0.090655 | -0.1764 | 0.991459 |
| Coriobacteriales | BMI | 0.093632 | 0.174266 | 0.991459 |
| Bacillales | Weight.lbs | 0.094453 | -0.19103 | 0.991459 |
| Coriobacteriales | Height.inches | 0.095338 | -0.18395 | 0.991459 |
| shannonDiversity (order) | EDEQ4.Total | 0.098834 | 0.189965 | 0.991459 |
| Bacteroidales | Mini.IPIP.Extraversion | 0.10088 | 0.162881 | 0.991459 |
| Desulfurococcales | Mini.IPIP.Agreeableness | 0.104499 | -0.17331 | 0.991459 |
| Lactobacillales | Mini.IPIP.Neuroticism | 0.106451 | 0.185539 | 0.991459 |
| Desulfurococcales | Qualtrics.age | 0.108295 | -0.172 | 0.991459 |
| Enterobacteriales | BAI.score | 0.109397 | 0.166791 | 0.991459 |
| Campylobacterales | Mini.IPIP.Extraversion | 0.111789 | 0.166029 | 0.991459 |
| Enterobacteriales | BDI.II.score | 0.11527 | 0.166244 | 0.991459 |
| Selenomonadales | Mini.IPIP.Extraversion | 0.117107 | 0.173922 | 0.991459 |
| shannonDiversity (order) | EDEQ4.Restraint | 0.118327 | 0.169571 | 0.991459 |
| Campylobacterales | Mini.IPIP.Conscientiousness | 0.12081 | -0.16418 | 0.991459 |
| Pasteurellales | Mini.IPIP.Extraversion | 0.137178 | 0.161637 | 0.991459 |
| Pasteurellales | BDI.II.score | 0.142127 | 0.172536 | 0.991459 |
| Clostridiales | EDEQ4.Total | 0.142338 | -0.17095 | 0.991459 |
| Rhodospirillales | Height.inches | 0.144283 | 0.151607 | 0.991459 |
| Methanobacteriales | EDEQ4.Shape | 0.148647 | 0.156833 | 0.991459 |
| Victivallales | Weight.lbs | 0.151766 | 0.15693 | 0.991459 |
| Desulfurococcales | PSS.10.score | 0.159185 | 0.154042 | 0.991459 |
| Verrucomicrobiales | Weight.lbs | 0.161412 | 0.156211 | 0.991459 |
| shannonDiversity (order) | EDEQ4.Weight | 0.161542 | 0.159175 | 0.991459 |
| Desulfovibrionales | EDEQ4.Weight | 0.163746 | 0.151662 | 0.991459 |
| Victivallales | PSS.10.score | 0.170492 | -0.14607 | 0.991459 |
| Lactobacillales | Mini.IPIP.Extraversion | 0.171188 | -0.14714 | 0.991459 |
| Clostridiales | EDEQ4.Weight | 0.175384 | -0.15162 | 0.991459 |
| Verrucomicrobiales | BAI.score | 0.184221 | 0.140972 | 0.991459 |
| Burkholderiales | Mini.IPIP.Neuroticism | 0.187004 | -0.13644 | 0.991459 |
| Sphingobacteriales | Mini.IPIP.Agreeableness | 0.187371 | 0.134433 | 0.991459 |
| Pasteurellales | Mini.IPIP.Neuroticism | 0.188157 | 0.144909 | 0.991459 |
| Actinomycetales | BAI.score | 0.189144 | 0.148779 | 0.991459 |
| Bacteroidales | Mini.IPIP.Neuroticism | 0.1945 | -0.14995 | 0.991459 |
| Lactobacillales | Mini.IPIP.Imagination | 0.196694 | -0.13364 | 0.991459 |
| Bacillales | EDEQ4.Total | 0.207347 | -0.13671 | 0.991459 |
| Desulfovibrionales | BAI.score | 0.208419 | 0.129539 | 0.991459 |
| Verrucomicrobiales | EDEQ4.Total | 0.209695 | 0.145277 | 0.991459 |
| Rhodospirillales | EDEQ4.Shape | 0.212226 | -0.13547 | 0.991459 |
| Burkholderiales | Height.inches | 0.215384 | -0.12783 | 0.991459 |
| Methanobacteriales | EDEQ4.Restraint | 0.216181 | 0.137093 | 0.991459 |
| Verrucomicrobiales | EDEQ4.Weight | 0.216589 | 0.141015 | 0.991459 |
| shannonDiversity (order) | BDI.II.score | 0.221082 | 0.138275 | 0.991459 |
| Verrucomicrobiales | Mini.IPIP.Conscientiousness | 0.221945 | 0.128278 | 0.991459 |
| Fusobacteriales | EDEQ4.Weight | 0.226617 | 0.136059 | 0.991459 |
| Desulfurococcales | Mini.IPIP.Extraversion | 0.228819 | -0.12827 | 0.991459 |
| Victivallales | Mini.IPIP.Conscientiousness | 0.22913 | 0.126769 | 0.991459 |
| Clostridiales | EDEQ4.Restraint | 0.233052 | -0.13535 | 0.991459 |
| Bifidobacteriales | PSS.10.score | 0.242682 | -0.11913 | 0.991459 |
| Clostridiales | Qualtrics.age | 0.245509 | -0.13034 | 0.991459 |
| Clostridiales | Mini.IPIP.Neuroticism | 0.250164 | 0.118306 | 0.991459 |
| Victivallales | Qualtrics.age | 0.250476 | 0.120067 | 0.991459 |
| Desulfovibrionales | EDEQ4.Shape | 0.251221 | 0.124066 | 0.991459 |
| Erysipelotrichales | BAI.score | 0.252854 | 0.113291 | 0.991459 |
| Enterobacteriales | Height.inches | 0.254659 | -0.11755 | 0.991459 |
| Pasteurellales | Mini.IPIP.Imagination | 0.255826 | -0.12734 | 0.991459 |
| Bacillales | BMI | 0.258992 | -0.12911 | 0.991459 |
| Burkholderiales | BMI | 0.260058 | 0.124584 | 0.991459 |
| Desulfovibrionales | Weight.lbs | 0.260672 | 0.120585 | 0.991459 |
| Clostridiales | Height.inches | 0.265641 | 0.125861 | 0.991459 |
| Erysipelotrichales | Height.inches | 0.265641 | -0.12808 | 0.991459 |
| Verrucomicrobiales | Mini.IPIP.Agreeableness | 0.267371 | -0.12309 | 0.991459 |
| Enterobacteriales | EDEQ4.Shape | 0.273147 | 0.125887 | 0.991459 |
| Desulfurococcales | Mini.IPIP.Conscientiousness | 0.276969 | -0.12955 | 0.991459 |
| Bacillales | Mini.IPIP.Extraversion | 0.281702 | -0.12057 | 0.991459 |
| Bacteroidales | Qualtrics.age | 0.283215 | 0.112095 | 0.991459 |
| Methanobacteriales | EDEQ4.Eating | 0.287271 | 0.114941 | 0.991459 |
| Fusobacteriales | Height.inches | 0.287747 | -0.11232 | 0.991459 |
| Lactobacillales | Height.inches | 0.288516 | -0.12012 | 0.991459 |
| shannonDiversity (order) | Qualtrics.age | 0.296613 | 0.111866 | 0.991459 |
| Campylobacterales | BMI | 0.302312 | 0.113284 | 0.991459 |
| Coriobacteriales | Mini.IPIP.Extraversion | 0.306488 | -0.10703 | 0.991459 |
| Lactobacillales | Qualtrics.age | 0.306935 | -0.11904 | 0.991459 |
| Actinomycetales | Mini.IPIP.Extraversion | 0.3135 | -0.10614 | 0.991459 |
| Clostridiales | EDEQ4.Shape | 0.313679 | -0.10966 | 0.991459 |
| Fusobacteriales | PSS.10.score | 0.31677 | 0.112081 | 0.991459 |
| Campylobacterales | Qualtrics.age | 0.324412 | -0.1143 | 0.991459 |
| Actinomycetales | PSS.10.score | 0.324814 | 0.11008 | 0.991459 |
| Enterobacteriales | Mini.IPIP.Imagination | 0.326477 | -0.10845 | 0.991459 |
| Coriobacteriales | BAI.score | 0.326519 | 0.095505 | 0.991459 |
| shannonDiversity (order) | EDEQ4.Shape | 0.328017 | 0.117376 | 0.991459 |
| Rhodospirillales | Mini.IPIP.Neuroticism | 0.329253 | -0.1088 | 0.991459 |
| Erysipelotrichales | BDI.II.score | 0.334606 | 0.112416 | 0.991459 |
| Desulfovibrionales | Mini.IPIP.Imagination | 0.337483 | -0.1069 | 0.991459 |
| Actinomycetales | Mini.IPIP.Imagination | 0.337534 | -0.09619 | 0.991459 |
| Bacteroidales | EDEQ4.Total | 0.340266 | 0.120718 | 0.991459 |
| shannonDiversity (order) | Height.inches | 0.341891 | -0.10062 | 0.991459 |
| Desulfurococcales | EDEQ4.Weight | 0.342551 | -0.10511 | 0.991459 |
| Desulfurococcales | Height.inches | 0.347019 | 0.098871 | 0.991459 |
| Methanobacteriales | Weight.lbs | 0.347848 | 0.096935 | 0.991459 |
| Victivallales | Mini.IPIP.Agreeableness | 0.348712 | 0.103475 | 0.991459 |
| Verrucomicrobiales | PSS.10.score | 0.354534 | 0.109943 | 0.991459 |
| Lactobacillales | Mini.IPIP.Agreeableness | 0.35534 | -0.10216 | 0.991459 |
| Bacteroidales | EDEQ4.Restraint | 0.355876 | 0.111242 | 0.991459 |
| Victivallales | EDEQ4.Total | 0.356926 | 0.092948 | 0.991459 |
| Pasteurellales | EDEQ4.Weight | 0.362278 | -0.09637 | 0.991459 |
| Enterobacteriales | EDEQ4.Weight | 0.362338 | 0.100825 | 0.991459 |
| Victivallales | BMI | 0.366167 | 0.097722 | 0.991459 |
| Enterobacteriales | Mini.IPIP.Neuroticism | 0.366496 | 0.093983 | 0.991459 |
| Methanobacteriales | BDI.II.score | 0.366638 | 0.10579 | 0.991459 |
| Pasteurellales | Mini.IPIP.Conscientiousness | 0.375753 | -0.09745 | 0.991459 |
| Verrucomicrobiales | EDEQ4.Shape | 0.377501 | 0.087408 | 0.991459 |
| Actinomycetales | EDEQ4.Shape | 0.377501 | -0.10674 | 0.991459 |
| Erysipelotrichales | EDEQ4.Eating | 0.37928 | 0.094619 | 0.991459 |
| Verrucomicrobiales | Mini.IPIP.Neuroticism | 0.380375 | 0.102448 | 0.991459 |
| Coriobacteriales | EDEQ4.Restraint | 0.380438 | 0.09475 | 0.991459 |
| Verrucomicrobiales | EDEQ4.Restraint | 0.380438 | 0.098207 | 0.991459 |
| Rhodospirillales | BAI.score | 0.384469 | -0.10139 | 0.991459 |
| Bacillales | Mini.IPIP.Agreeableness | 0.385925 | -0.09321 | 0.991459 |
| Bacillales | EDEQ4.Shape | 0.392557 | -0.08756 | 0.991459 |
| shannonDiversity (order) | EDEQ4.Eating | 0.397185 | 0.097053 | 0.991459 |
| Burkholderiales | Mini.IPIP.Conscientiousness | 0.398112 | 0.09056 | 0.991459 |
| Campylobacterales | BAI.score | 0.400482 | 0.092177 | 0.991459 |
| Burkholderiales | Mini.IPIP.Imagination | 0.404487 | 0.091363 | 0.991459 |
| Desulfovibrionales | Mini.IPIP.Extraversion | 0.407813 | -0.08655 | 0.991459 |
| Actinomycetales | Weight.lbs | 0.408253 | -0.09322 | 0.991459 |
| Methanobacteriales | Qualtrics.age | 0.408528 | 0.088179 | 0.991459 |
| Desulfurococcales | BAI.score | 0.408557 | 0.092106 | 0.991459 |
| Bacteroidales | EDEQ4.Weight | 0.409366 | 0.098791 | 0.991459 |
| Sphingobacteriales | BMI | 0.409841 | 0.082513 | 0.991459 |
| Selenomonadales | Qualtrics.age | 0.414575 | -0.0819 | 0.991459 |
| Methanobacteriales | Mini.IPIP.Conscientiousness | 0.415213 | 0.093426 | 0.991459 |
| shannonDiversity (order) | BAI.score | 0.417092 | 0.089678 | 0.991459 |
| Coriobacteriales | BDI.II.score | 0.418165 | 0.083959 | 0.991459 |
| Erysipelotrichales | Mini.IPIP.Conscientiousness | 0.419034 | -0.0814 | 0.991459 |
| Erysipelotrichales | Mini.IPIP.Imagination | 0.421325 | -0.0914 | 0.991459 |
| Bifidobacteriales | Mini.IPIP.Imagination | 0.425597 | 0.08614 | 0.991459 |
| Victivallales | Mini.IPIP.Neuroticism | 0.428388 | -0.08663 | 0.991459 |
| Burkholderiales | EDEQ4.Eating | 0.429718 | 0.084633 | 0.991459 |
| Desulfurococcales | EDEQ4.Shape | 0.433762 | 0.081401 | 0.991459 |
| Campylobacterales | EDEQ4.Restraint | 0.434742 | -0.08092 | 0.991459 |
| Burkholderiales | EDEQ4.Restraint | 0.437117 | 0.077447 | 0.991459 |
| Coriobacteriales | EDEQ4.Shape | 0.439998 | 0.079012 | 0.991459 |
| Sphingobacteriales | EDEQ4.Restraint | 0.442947 | 0.083083 | 0.991459 |
| Lactobacillales | Weight.lbs | 0.446768 | -0.08465 | 0.991459 |
| Burkholderiales | Mini.IPIP.Agreeableness | 0.450721 | 0.074461 | 0.991459 |
| Verrucomicrobiales | Mini.IPIP.Imagination | 0.45176 | -0.0778 | 0.991459 |
| Enterobacteriales | EDEQ4.Total | 0.454289 | 0.08381 | 0.991459 |
| Desulfovibrionales | PSS.10.score | 0.462421 | 0.07546 | 0.991459 |
| Pasteurellales | Height.inches | 0.466199 | 0.070731 | 0.991459 |
| Fusobacteriales | BDI.II.score | 0.47048 | -0.07811 | 0.991459 |
| Desulfurococcales | BMI | 0.474429 | -0.07678 | 0.991459 |
| Actinomycetales | Mini.IPIP.Neuroticism | 0.474588 | 0.085573 | 0.991459 |
| Bifidobacteriales | Height.inches | 0.479967 | 0.08436 | 0.991459 |
| Rhodospirillales | Qualtrics.age | 0.487088 | 0.0769 | 0.991459 |
| Burkholderiales | BDI.II.score | 0.48937 | -0.07943 | 0.991459 |
| Actinomycetales | BMI | 0.492698 | -0.07784 | 0.991459 |
| Desulfovibrionales | EDEQ4.Eating | 0.494034 | 0.072927 | 0.991459 |
| Campylobacterales | BDI.II.score | 0.504147 | 0.068885 | 0.991459 |
| Desulfovibrionales | Height.inches | 0.507941 | 0.071856 | 0.991459 |
| Actinomycetales | Mini.IPIP.Conscientiousness | 0.513679 | -0.06794 | 0.991459 |
| Lactobacillales | EDEQ4.Total | 0.513836 | -0.0694 | 0.991459 |
| Campylobacterales | PSS.10.score | 0.515068 | 0.074189 | 0.991459 |
| Sphingobacteriales | EDEQ4.Total | 0.515471 | 0.088577 | 0.991459 |
| Desulfovibrionales | BMI | 0.516073 | 0.080243 | 0.991459 |
| Bacillales | EDEQ4.Eating | 0.516611 | -0.07744 | 0.991459 |
| Bacteroidales | EDEQ4.Shape | 0.517519 | 0.067335 | 0.991459 |
| Methanobacteriales | EDEQ4.Weight | 0.518473 | 0.072358 | 0.991459 |
| Bacteroidales | EDEQ4.Eating | 0.520136 | 0.072234 | 0.991459 |
| Methanobacteriales | BMI | 0.523217 | 0.07268 | 0.991459 |
| Rhodospirillales | BMI | 0.524348 | -0.07049 | 0.991459 |
| Clostridiales | EDEQ4.Eating | 0.525432 | -0.06899 | 0.991459 |
| Clostridiales | PSS.10.score | 0.527442 | 0.065511 | 0.991459 |
| Campylobacterales | Mini.IPIP.Imagination | 0.533315 | -0.06841 | 0.991459 |
| Fusobacteriales | EDEQ4.Total | 0.53668 | 0.065262 | 0.991459 |
| Enterobacteriales | Weight.lbs | 0.539327 | -0.07424 | 0.991459 |
| Lactobacillales | EDEQ4.Eating | 0.541483 | -0.06658 | 0.991459 |
| Lactobacillales | EDEQ4.Shape | 0.541595 | -0.0629 | 0.991459 |
| Pasteurellales | EDEQ4.Total | 0.542539 | -0.05528 | 0.991459 |
| shannonDiversity (order) | Mini.IPIP.Agreeableness | 0.544297 | -0.06729 | 0.991459 |
| Sphingobacteriales | EDEQ4.Shape | 0.545973 | 0.069414 | 0.991459 |
| Campylobacterales | EDEQ4.Eating | 0.554674 | 0.064358 | 0.991459 |
| Clostridiales | BMI | 0.554761 | -0.0662 | 0.991459 |
| Coriobacteriales | EDEQ4.Total | 0.557301 | 0.053367 | 0.991459 |
| Bacteroidales | Mini.IPIP.Conscientiousness | 0.557404 | -0.06547 | 0.991459 |
| Sphingobacteriales | Mini.IPIP.Neuroticism | 0.557445 | 0.064649 | 0.991459 |
| Selenomonadales | EDEQ4.Weight | 0.557813 | 0.06971 | 0.991459 |
| Fusobacteriales | Mini.IPIP.Extraversion | 0.559207 | 0.070746 | 0.991459 |
| Desulfurococcales | EDEQ4.Eating | 0.562807 | 0.062504 | 0.991459 |
| Bacteroidales | BMI | 0.564638 | 0.064225 | 0.991459 |
| Fusobacteriales | Mini.IPIP.Agreeableness | 0.565294 | 0.065505 | 0.991459 |
| Rhodospirillales | BDI.II.score | 0.567672 | 0.056359 | 0.991459 |
| Burkholderiales | PSS.10.score | 0.57154 | 0.053223 | 0.991459 |
| Bacillales | Mini.IPIP.Imagination | 0.571659 | -0.06308 | 0.991459 |
| Selenomonadales | Mini.IPIP.Agreeableness | 0.574141 | 0.059117 | 0.991459 |
| Bacillales | BDI.II.score | 0.575851 | 0.065021 | 0.991459 |
| Actinomycetales | Height.inches | 0.576553 | -0.06341 | 0.991459 |
| Bacillales | EDEQ4.Weight | 0.582345 | -0.05452 | 0.991459 |
| Methanobacteriales | BAI.score | 0.583712 | 0.05244 | 0.991459 |
| Coriobacteriales | Weight.lbs | 0.58394 | 0.044533 | 0.991459 |
| Coriobacteriales | Mini.IPIP.Conscientiousness | 0.592621 | 0.064455 | 0.991459 |
| Selenomonadales | Mini.IPIP.Conscientiousness | 0.592621 | 0.060157 | 0.991459 |
| Sphingobacteriales | Qualtrics.age | 0.593942 | 0.056946 | 0.991459 |
| Bacteroidales | PSS.10.score | 0.596774 | -0.06101 | 0.991459 |
| Pasteurellales | EDEQ4.Restraint | 0.598113 | -0.05388 | 0.991459 |
| Victivallales | Height.inches | 0.598387 | 0.065563 | 0.991459 |
| shannonDiversity (order) | Mini.IPIP.Extraversion | 0.60143 | -0.05945 | 0.991459 |
| Selenomonadales | EDEQ4.Total | 0.602482 | 0.060213 | 0.991459 |
| Bifidobacteriales | Mini.IPIP.Conscientiousness | 0.60802 | 0.055574 | 0.991459 |
| Pasteurellales | EDEQ4.Eating | 0.610826 | 0.055275 | 0.991459 |
| Lactobacillales | EDEQ4.Weight | 0.614286 | -0.05772 | 0.991459 |
| Bacillales | Qualtrics.age | 0.616424 | -0.06344 | 0.991459 |
| Bifidobacteriales | EDEQ4.Restraint | 0.617008 | 0.053248 | 0.991459 |
| Lactobacillales | EDEQ4.Restraint | 0.617008 | 0.057412 | 0.991459 |
| Methanobacteriales | Mini.IPIP.Extraversion | 0.6186 | 0.048122 | 0.991459 |
| Clostridiales | Mini.IPIP.Agreeableness | 0.620307 | 0.055371 | 0.991459 |
| Rhodospirillales | Mini.IPIP.Extraversion | 0.620553 | 0.059859 | 0.991459 |
| Enterobacteriales | Qualtrics.age | 0.622373 | 0.050876 | 0.991459 |
| Desulfurococcales | BDI.II.score | 0.633896 | 0.048203 | 0.991459 |
| Bifidobacteriales | Weight.lbs | 0.640658 | 0.076261 | 0.991459 |
| Burkholderiales | BAI.score | 0.642107 | 0.038915 | 0.991459 |
| Erysipelotrichales | Mini.IPIP.Neuroticism | 0.642359 | 0.047749 | 0.991459 |
| Enterobacteriales | EDEQ4.Eating | 0.645526 | 0.051876 | 0.991459 |
| Selenomonadales | BMI | 0.646541 | 0.052718 | 0.991459 |
| Lactobacillales | Mini.IPIP.Conscientiousness | 0.649927 | 0.051247 | 0.991459 |
| shannonDiversity (order) | BMI | 0.65182 | 0.040346 | 0.991459 |
| Methanobacteriales | Height.inches | 0.657023 | 0.049146 | 0.991459 |
| Selenomonadales | Mini.IPIP.Imagination | 0.658028 | 0.050194 | 0.991459 |
| Coriobacteriales | EDEQ4.Eating | 0.660218 | 0.051293 | 0.991459 |
| Fusobacteriales | Weight.lbs | 0.66162 | -0.04656 | 0.991459 |
| Fusobacteriales | EDEQ4.Eating | 0.66258 | 0.046151 | 0.991459 |
| Sphingobacteriales | Weight.lbs | 0.667553 | 0.049401 | 0.991459 |
| Campylobacterales | Height.inches | 0.668656 | -0.04317 | 0.991459 |
| Victivallales | Mini.IPIP.Imagination | 0.669055 | -0.04894 | 0.991459 |
| Actinomycetales | EDEQ4.Total | 0.675849 | -0.04835 | 0.991459 |
| Campylobacterales | Mini.IPIP.Neuroticism | 0.679214 | 0.046351 | 0.991459 |
| Sphingobacteriales | Height.inches | 0.679228 | -0.04576 | 0.991459 |
| Burkholderiales | Qualtrics.age | 0.680625 | 0.05586 | 0.991459 |
| Erysipelotrichales | EDEQ4.Restraint | 0.683443 | -0.04304 | 0.991459 |
| Sphingobacteriales | EDEQ4.Weight | 0.692959 | 0.05987 | 0.991459 |
| Burkholderiales | Weight.lbs | 0.694119 | 0.049053 | 0.991459 |
| Selenomonadales | Weight.lbs | 0.694119 | 0.040952 | 0.991459 |
| Clostridiales | BDI.II.score | 0.697891 | -0.04579 | 0.991459 |
| Actinomycetales | EDEQ4.Weight | 0.700603 | 0.04877 | 0.991459 |
| Fusobacteriales | Mini.IPIP.Conscientiousness | 0.701031 | -0.04557 | 0.991459 |
| Bacteroidales | BAI.score | 0.70127 | 0.030879 | 0.991459 |
| Burkholderiales | EDEQ4.Shape | 0.702149 | -0.03834 | 0.991459 |
| Rhodospirillales | Weight.lbs | 0.703784 | 0.041828 | 0.991459 |
| Sphingobacteriales | EDEQ4.Eating | 0.706045 | 0.039139 | 0.991459 |
| Desulfovibrionales | Mini.IPIP.Conscientiousness | 0.706609 | -0.04248 | 0.991459 |
| Selenomonadales | EDEQ4.Eating | 0.708044 | 0.02702 | 0.991459 |
| Lactobacillales | BMI | 0.716463 | -0.04435 | 0.991459 |
| Bifidobacteriales | EDEQ4.Weight | 0.717271 | -0.04372 | 0.991459 |
| Fusobacteriales | Mini.IPIP.Neuroticism | 0.721971 | -0.03051 | 0.991459 |
| Bifidobacteriales | Mini.IPIP.Extraversion | 0.723988 | 0.034126 | 0.991459 |
| Verrucomicrobiales | Mini.IPIP.Extraversion | 0.723988 | -0.04274 | 0.991459 |
| Pasteurellales | EDEQ4.Shape | 0.724036 | -0.03625 | 0.991459 |
| Bifidobacteriales | BDI.II.score | 0.725438 | 0.040441 | 0.991459 |
| Clostridiales | Weight.lbs | 0.726965 | 0.034048 | 0.991459 |
| Selenomonadales | PSS.10.score | 0.735362 | -0.0344 | 0.991459 |
| Selenomonadales | BAI.score | 0.739914 | 0.044669 | 0.991459 |
| Fusobacteriales | EDEQ4.Shape | 0.749331 | -0.03778 | 0.991459 |
| Victivallales | EDEQ4.Shape | 0.752616 | -0.04148 | 0.991459 |
| Campylobacterales | Mini.IPIP.Agreeableness | 0.753259 | -0.03126 | 0.991459 |
| Erysipelotrichales | EDEQ4.Weight | 0.756679 | 0.039722 | 0.991459 |
| Selenomonadales | Mini.IPIP.Neuroticism | 0.756864 | -0.03457 | 0.991459 |
| Sphingobacteriales | Mini.IPIP.Imagination | 0.757768 | -0.05269 | 0.991459 |
| Campylobacterales | Weight.lbs | 0.761196 | 0.040439 | 0.991459 |
| Rhodospirillales | Mini.IPIP.Imagination | 0.761451 | 0.031791 | 0.991459 |
| Erysipelotrichales | PSS.10.score | 0.763214 | 0.032518 | 0.991459 |
| Fusobacteriales | EDEQ4.Restraint | 0.763309 | -0.03117 | 0.991459 |
| Fusobacteriales | BMI | 0.770849 | 0.035182 | 0.991459 |
| Coriobacteriales | Qualtrics.age | 0.774375 | 0.032592 | 0.991459 |
| Lactobacillales | PSS.10.score | 0.774444 | 0.022878 | 0.991459 |
| Enterobacteriales | Mini.IPIP.Extraversion | 0.779799 | 0.034946 | 0.991459 |
| Bacteroidales | Height.inches | 0.7815 | -0.0389 | 0.991459 |
| Methanobacteriales | Mini.IPIP.Agreeableness | 0.783614 | 0.025968 | 0.991459 |
| Lactobacillales | BAI.score | 0.784891 | 0.034068 | 0.991459 |
| Coriobacteriales | PSS.10.score | 0.785721 | 0.027944 | 0.991459 |
| Methanobacteriales | Mini.IPIP.Neuroticism | 0.787138 | -0.0351 | 0.991459 |
| Victivallales | EDEQ4.Eating | 0.787167 | 0.030149 | 0.991459 |
| Lactobacillales | BDI.II.score | 0.78726 | 0.029706 | 0.991459 |
| Verrucomicrobiales | BMI | 0.788848 | 0.027462 | 0.991459 |
| shannonDiversity (order) | Mini.IPIP.Imagination | 0.796248 | -0.03725 | 0.991459 |
| Methanobacteriales | PSS.10.score | 0.799322 | 0.028635 | 0.991459 |
| Sphingobacteriales | Mini.IPIP.Conscientiousness | 0.80045 | -0.03689 | 0.991459 |
| Actinomycetales | Mini.IPIP.Agreeableness | 0.801501 | -0.02639 | 0.991459 |
| Clostridiales | Mini.IPIP.Imagination | 0.801947 | -0.01916 | 0.991459 |
| Selenomonadales | Height.inches | 0.804305 | 0.035769 | 0.991459 |
| Bacillales | Mini.IPIP.Conscientiousness | 0.807173 | 0.024262 | 0.991459 |
| Fusobacteriales | Mini.IPIP.Imagination | 0.809279 | -0.02989 | 0.991459 |
| Bifidobacteriales | Mini.IPIP.Neuroticism | 0.81352 | 0.024621 | 0.991459 |
| Clostridiales | Mini.IPIP.Conscientiousness | 0.816176 | 0.022173 | 0.991459 |
| shannonDiversity (order) | Weight.lbs | 0.816886 | -0.01786 | 0.991459 |
| Verrucomicrobiales | BDI.II.score | 0.821584 | 0.044197 | 0.991459 |
| shannonDiversity (order) | Mini.IPIP.Conscientiousness | 0.821912 | -0.01742 | 0.991459 |
| Pasteurellales | Weight.lbs | 0.822568 | 0.025851 | 0.991459 |
| Sphingobacteriales | PSS.10.score | 0.82426 | -0.03009 | 0.991459 |
| Erysipelotrichales | Mini.IPIP.Agreeableness | 0.824448 | -0.02825 | 0.991459 |
| Bacteroidales | BDI.II.score | 0.833106 | -0.01974 | 0.993634 |
| Bacillales | PSS.10.score | 0.833761 | 0.027097 | 0.993634 |
| Selenomonadales | BDI.II.score | 0.83888 | 0.030487 | 0.993634 |
| Bifidobacteriales | BAI.score | 0.842121 | -0.01135 | 0.993634 |
| Desulfovibrionales | Mini.IPIP.Neuroticism | 0.842223 | 0.020887 | 0.993634 |
| Sphingobacteriales | Mini.IPIP.Extraversion | 0.847198 | 0.015196 | 0.993634 |
| Coriobacteriales | Mini.IPIP.Imagination | 0.853674 | 0.02277 | 0.993634 |
| Rhodospirillales | EDEQ4.Weight | 0.855076 | 0.018223 | 0.993634 |
| shannonDiversity (order) | Mini.IPIP.Neuroticism | 0.859575 | -0.0154 | 0.993634 |
| Rhodospirillales | PSS.10.score | 0.86372 | 0.017363 | 0.993634 |
| Rhodospirillales | EDEQ4.Total | 0.867136 | 0.010361 | 0.993634 |
| Campylobacterales | EDEQ4.Weight | 0.870448 | 0.019703 | 0.993634 |
| Clostridiales | BAI.score | 0.871073 | -0.02009 | 0.993634 |
| Desulfovibrionales | Qualtrics.age | 0.871525 | -0.0171 | 0.993634 |
| Actinomycetales | EDEQ4.Restraint | 0.871645 | -0.01602 | 0.993634 |
| Desulfovibrionales | BDI.II.score | 0.876597 | -0.01813 | 0.993634 |
| Campylobacterales | EDEQ4.Shape | 0.8826 | -0.01995 | 0.993634 |
| Clostridiales | Mini.IPIP.Extraversion | 0.883022 | -0.02023 | 0.993634 |
| Selenomonadales | EDEQ4.Shape | 0.883065 | -0.01336 | 0.993634 |
| Sphingobacteriales | BAI.score | 0.887714 | -0.02591 | 0.993634 |
| Desulfurococcales | Weight.lbs | 0.888647 | -0.00891 | 0.993634 |
| Bifidobacteriales | EDEQ4.Eating | 0.889927 | -0.00028 | 0.993634 |
| Verrucomicrobiales | EDEQ4.Eating | 0.889927 | -0.01396 | 0.993634 |
| Burkholderiales | EDEQ4.Weight | 0.890143 | -0.02097 | 0.993634 |
| Victivallales | EDEQ4.Weight | 0.896053 | 0.010962 | 0.993634 |
| Desulfurococcales | EDEQ4.Restraint | 0.900579 | -0.01682 | 0.993634 |
| Selenomonadales | EDEQ4.Restraint | 0.908123 | 0.014315 | 0.993634 |
| Desulfovibrionales | Mini.IPIP.Agreeableness | 0.908744 | 0.009789 | 0.993634 |
| Enterobacteriales | BMI | 0.90945 | -0.0137 | 0.993634 |
| Bifidobacteriales | Mini.IPIP.Agreeableness | 0.911685 | -0.01374 | 0.993634 |
| Victivallales | BDI.II.score | 0.914713 | -0.01442 | 0.993634 |
| Fusobacteriales | Qualtrics.age | 0.915225 | 0.010697 | 0.993634 |
| Methanobacteriales | Mini.IPIP.Imagination | 0.919281 | -0.00576 | 0.993634 |
| Pasteurellales | BAI.score | 0.926515 | -0.00385 | 0.993634 |
| Sphingobacteriales | BDI.II.score | 0.928782 | 0.003329 | 0.993634 |
| Coriobacteriales | Mini.IPIP.Neuroticism | 0.929513 | -0.00791 | 0.993634 |
| Rhodospirillales | Mini.IPIP.Conscientiousness | 0.93785 | -0.00332 | 0.993634 |
| Bacteroidales | Weight.lbs | 0.938477 | -0.01252 | 0.993634 |
| Rhodospirillales | EDEQ4.Eating | 0.938701 | -0.0138 | 0.993634 |
| Coriobacteriales | EDEQ4.Weight | 0.943455 | -0.00395 | 0.993634 |
| Bacillales | BAI.score | 0.944005 | 0.012203 | 0.993634 |
| Bifidobacteriales | EDEQ4.Shape | 0.947228 | -0.02534 | 0.993634 |
| Erysipelotrichales | EDEQ4.Total | 0.947361 | -0.00226 | 0.993634 |
| Bacteroidales | Mini.IPIP.Agreeableness | 0.952829 | 0.006152 | 0.993634 |
| Desulfurococcales | Mini.IPIP.Neuroticism | 0.953902 | -0.00257 | 0.993634 |
| Actinomycetales | EDEQ4.Eating | 0.954556 | -0.00533 | 0.993634 |
| shannonDiversity (order) | PSS.10.score | 0.958979 | 0.009786 | 0.993634 |
| Bifidobacteriales | Qualtrics.age | 0.964824 | -0.00725 | 0.993634 |
| Pasteurellales | BMI | 0.96488 | 0.006607 | 0.993634 |
| Bifidobacteriales | EDEQ4.Total | 0.964893 | -0.01027 | 0.993634 |
| Bifidobacteriales | BMI | 0.967811 | -0.00445 | 0.993634 |
| Coriobacteriales | Mini.IPIP.Agreeableness | 0.970508 | 0.001616 | 0.993634 |
| Erysipelotrichales | EDEQ4.Shape | 0.970667 | 0.011978 | 0.993634 |
| Actinomycetales | Qualtrics.age | 0.970684 | -0.01101 | 0.993634 |
| Enterobacteriales | EDEQ4.Restraint | 0.97238 | 0.001409 | 0.993634 |
| Bacillales | Mini.IPIP.Neuroticism | 0.979371 | 0.010895 | 0.995755 |
| Bacteroidales | Mini.IPIP.Imagination | 0.982343 | 0.003683 | 0.995755 |
| Burkholderiales | EDEQ4.Total | 0.982442 | -3.65E-05 | 0.995755 |
| Enterobacteriales | Mini.IPIP.Agreeableness | 0.9882 | 0.010078 | 0.998169 |
| Desulfurococcales | EDEQ4.Total | 0.990162 | 0.008498 | 0.998169 |
| Campylobacterales | EDEQ4.Total | 0.996543 | 0.002978 | 0.999729 |
| Enterobacteriales | Mini.IPIP.Conscientiousness | 0.997056 | 0.003997 | 0.999729 |
| Erysipelotrichales | Qualtrics.age | 1 | -0.0015 | 1 |
| Porphyromonadaceae | Qualtrics.age | 0.000468 | 0.378053 | 0.357667 |
| Lachnospiraceae | EDEQ4.Total | 0.005432 | -0.28966 | 0.928653 |
| Clostridiales_Incertae.Sedis.XI | Height.inches | 0.005706 | -0.29612 | 0.928653 |
| Clostridiaceae.1 | Height.inches | 0.006137 | 0.285239 | 0.928653 |
| Ruminococcaceae | Height.inches | 0.006864 | 0.278497 | 0.928653 |
| Desulfovibrionaceae | EDEQ4.Restraint | 0.007668 | 0.287568 | 0.928653 |
| Verrucomicrobiaceae | Qualtrics.age | 0.00869 | 0.272543 | 0.928653 |
| Carnobacteriaceae | Height.inches | 0.011613 | -0.28559 | 0.928653 |
| Erysipelotrichaceae | Weight.lbs | 0.012062 | -0.27988 | 0.928653 |
| Defluviitaleaceae | Mini.IPIP.Conscientiousness | 0.012439 | 0.266208 | 0.928653 |
| Bacillales_Incertae.Sedis.XI | Height.inches | 0.013898 | -0.27706 | 0.928653 |
| Erysipelotrichaceae | BMI | 0.015624 | -0.26712 | 0.928653 |
| Eubacteriaceae | BMI | 0.015781 | -0.24601 | 0.928653 |
| Pasteurellaceae | Qualtrics.age | 0.019978 | -0.23619 | 0.990385 |
| Corynebacteriaceae | Mini.IPIP.Agreeableness | 0.022652 | 0.241993 | 0.990385 |
| Lachnospiraceae | EDEQ4.Restraint | 0.023228 | -0.24565 | 0.990385 |
| Acidaminococcaceae | Mini.IPIP.Neuroticism | 0.023633 | -0.24754 | 0.990385 |
| Enterobacteriaceae | PSS.10.score | 0.025979 | 0.231791 | 0.990385 |
| Pyrodictiaceae | Mini.IPIP.Imagination | 0.029359 | 0.240708 | 0.990385 |
| Clostridiaceae.2 | Weight.lbs | 0.031527 | 0.246377 | 0.990385 |
| Carnobacteriaceae | Weight.lbs | 0.034459 | -0.2436 | 0.990385 |
| Erysipelotrichaceae | Mini.IPIP.Extraversion | 0.03537 | -0.24083 | 0.990385 |
| Lachnospiraceae | EDEQ4.Shape | 0.038089 | -0.2218 | 0.990385 |
| Lachnospiraceae | Qualtrics.age | 0.042494 | -0.21409 | 0.990385 |
| Defluviitaleaceae | PSS.10.score | 0.046483 | -0.20691 | 0.990385 |
| Gracilibacteraceae | EDEQ4.Weight | 0.047637 | 0.216103 | 0.990385 |
| shannonDiversity (family) | EDEQ4.Total | 0.049303 | 0.214433 | 0.990385 |
| Clostridiaceae.2 | Height.inches | 0.049966 | 0.205637 | 0.990385 |
| Micrococcaceae | EDEQ4.Shape | 0.050298 | -0.21501 | 0.990385 |
| Prevotellaceae | EDEQ4.Eating | 0.050721 | 0.210513 | 0.990385 |
| Bacillales_Incertae.Sedis.XI | EDEQ4.Restraint | 0.051519 | -0.21604 | 0.990385 |
| Clostridiales_Incertae.Sedis.XIII | Mini.IPIP.Extraversion | 0.051663 | -0.20531 | 0.990385 |
| Victivallaceae | Mini.IPIP.Extraversion | 0.052302 | 0.20579 | 0.990385 |
| Micrococcaceae | BDI.II.score | 0.053038 | 0.206984 | 0.990385 |
| Defluviitaleaceae | BAI.score | 0.055268 | -0.20524 | 0.990385 |
| Carnobacteriaceae | BAI.score | 0.056854 | 0.200925 | 0.990385 |
| Verrucomicrobiaceae | Height.inches | 0.058788 | 0.210092 | 0.990385 |
| Methanobacteriaceae | EDEQ4.Total | 0.06045 | 0.200844 | 0.990385 |
| Aerococcaceae | Height.inches | 0.061205 | -0.20647 | 0.990385 |
| Prevotellaceae | Height.inches | 0.061819 | -0.20009 | 0.990385 |
| Gracilibacteraceae | Qualtrics.age | 0.062281 | 0.207018 | 0.990385 |
| Victivallaceae | EDEQ4.Restraint | 0.063367 | 0.19617 | 0.990385 |
| Fusobacteriaceae | BAI.score | 0.064923 | 0.211048 | 0.990385 |
| Corynebacteriaceae | BAI.score | 0.065742 | 0.199585 | 0.990385 |
| Defluviitaleaceae | Mini.IPIP.Extraversion | 0.067843 | -0.19523 | 0.990385 |
| Pasteurellaceae | Mini.IPIP.Agreeableness | 0.068301 | -0.20049 | 0.990385 |
| Micrococcaceae | PSS.10.score | 0.069932 | 0.196211 | 0.990385 |
| Eubacteriaceae | Height.inches | 0.069967 | 0.197982 | 0.990385 |
| Rikenellaceae | Height.inches | 0.0717 | 0.201311 | 0.990385 |
| Corynebacteriaceae | Height.inches | 0.072463 | -0.19775 | 0.990385 |
| Pasteurellaceae | PSS.10.score | 0.075319 | 0.19399 | 0.990385 |
| Sutterellaceae | Mini.IPIP.Extraversion | 0.076224 | 0.18243 | 0.990385 |
| Leuconostocaceae | Height.inches | 0.077424 | -0.18553 | 0.990385 |
| Desulfovibrionaceae | EDEQ4.Total | 0.079506 | 0.18607 | 0.990385 |
| Micrococcaceae | Mini.IPIP.Imagination | 0.08021 | -0.17853 | 0.990385 |
| Lactobacillaceae | EDEQ4.Total | 0.081411 | 0.196204 | 0.990385 |
| Clostridiales_Incertae.Sedis.XIII | Height.inches | 0.084822 | 0.181044 | 0.990385 |
| Coriobacteriaceae | BMI | 0.08533 | 0.17629 | 0.990385 |
| Aerococcaceae | BDI.II.score | 0.086329 | 0.182567 | 0.990385 |
| Oxalobacteraceae | EDEQ4.Eating | 0.087339 | 0.186101 | 0.990385 |
| Chitinophagaceae | Height.inches | 0.088139 | -0.17566 | 0.990385 |
| Pyrodictiaceae | Mini.IPIP.Agreeableness | 0.08841 | -0.18088 | 0.990385 |
| Victivallaceae | BAI.score | 0.088969 | -0.17835 | 0.990385 |
| Eubacteriaceae | Mini.IPIP.Neuroticism | 0.092106 | -0.17963 | 0.990385 |
| Lachnospiraceae | Height.inches | 0.092437 | -0.18812 | 0.990385 |
| Clostridiales_Incertae.Sedis.XI | Weight.lbs | 0.092822 | -0.19171 | 0.990385 |
| Defluviitaleaceae | Qualtrics.age | 0.093057 | 0.187749 | 0.990385 |
| Defluviitaleaceae | BDI.II.score | 0.093323 | -0.17999 | 0.990385 |
| Sutterellaceae | BMI | 0.093632 | 0.186092 | 0.990385 |
| Porphyromonadaceae | Mini.IPIP.Agreeableness | 0.097659 | 0.181952 | 0.990385 |
| Clostridiales_Incertae.Sedis.XI | EDEQ4.Restraint | 0.09947 | -0.18439 | 0.990385 |
| Coriobacteriaceae | Height.inches | 0.099826 | -0.17966 | 0.990385 |
| Defluviitaleaceae | Height.inches | 0.100729 | 0.171074 | 0.990385 |
| Clostridiaceae.2 | BMI | 0.102532 | 0.169386 | 0.990385 |
| Veillonellaceae | Qualtrics.age | 0.104293 | -0.18512 | 0.990385 |
| Sutterellaceae | Height.inches | 0.104481 | -0.17364 | 0.990385 |
| Actinomycetaceae | BDI.II.score | 0.104607 | 0.182977 | 0.990385 |
| Enterobacteriaceae | BDI.II.score | 0.106981 | 0.168458 | 0.990385 |
| Lactobacillaceae | EDEQ4.Restraint | 0.107774 | 0.178619 | 0.990385 |
| Campylobacteraceae | Mini.IPIP.Extraversion | 0.109845 | 0.167042 | 0.990385 |
| Bacillales_Incertae.Sedis.XI | Weight.lbs | 0.110387 | -0.17713 | 0.990385 |
| Oxalobacteraceae | Height.inches | 0.112578 | 0.169856 | 0.990385 |
| Rhodospirillaceae | Mini.IPIP.Agreeableness | 0.112705 | 0.170519 | 0.990385 |
| Gracilibacteraceae | EDEQ4.Total | 0.114216 | 0.174265 | 0.990385 |
| Enterobacteriaceae | BAI.score | 0.11609 | 0.164849 | 0.990385 |
| Chloroplast | BAI.score | 0.116396 | -0.17119 | 0.990385 |
| shannonDiversity (family) | EDEQ4.Restraint | 0.116525 | 0.170478 | 0.990385 |
| Lachnospiraceae | Mini.IPIP.Imagination | 0.117821 | 0.164905 | 0.990385 |
| Campylobacteraceae | Mini.IPIP.Conscientiousness | 0.118736 | -0.16533 | 0.990385 |
| Rhodospirillaceae | EDEQ4.Restraint | 0.118884 | 0.167877 | 0.990385 |
| Acidaminococcaceae | EDEQ4.Shape | 0.118973 | 0.175967 | 0.990385 |
| Ruminococcaceae | Weight.lbs | 0.120016 | 0.166532 | 0.990385 |
| Chloroplast | PSS.10.score | 0.123128 | -0.16909 | 0.990385 |
| Leuconostocaceae | Weight.lbs | 0.123486 | -0.16703 | 0.990385 |
| Leuconostocaceae | Mini.IPIP.Imagination | 0.123963 | -0.16275 | 0.990385 |
| Micrococcaceae | EDEQ4.Total | 0.12682 | -0.16583 | 0.990385 |
| Ruminococcaceae | Mini.IPIP.Imagination | 0.132332 | -0.17281 | 0.990385 |
| Pasteurellaceae | Mini.IPIP.Extraversion | 0.13333 | 0.161463 | 0.990385 |
| Pasteurellaceae | BDI.II.score | 0.134264 | 0.173611 | 0.990385 |
| shannonDiversity (family) | Qualtrics.age | 0.137614 | 0.164183 | 0.990385 |
| Ruminococcaceae | Mini.IPIP.Neuroticism | 0.138429 | 0.156212 | 0.990385 |
| Gracilibacteraceae | EDEQ4.Restraint | 0.13907 | 0.160818 | 0.990385 |
| Acidaminococcaceae | PSS.10.score | 0.141678 | -0.14498 | 0.990385 |
| Incertae.Sedis.XI | Mini.IPIP.Neuroticism | 0.149 | -0.16047 | 0.990385 |
| Incertae.Sedis.XI | Mini.IPIP.Agreeableness | 0.151143 | 0.154028 | 0.990385 |
| Victivallaceae | Weight.lbs | 0.154317 | 0.156413 | 0.990385 |
| Clostridiaceae.1 | Weight.lbs | 0.154913 | 0.15328 | 0.990385 |
| Aerococcaceae | Mini.IPIP.Neuroticism | 0.156505 | 0.167467 | 0.990385 |
| Lactobacillaceae | EDEQ4.Shape | 0.156788 | 0.178367 | 0.990385 |
| Lachnospiraceae | Weight.lbs | 0.157057 | -0.15402 | 0.990385 |
| Enterococcaceae | Mini.IPIP.Neuroticism | 0.158777 | -0.14698 | 0.990385 |
| Methanobacteriaceae | EDEQ4.Shape | 0.159454 | 0.154473 | 0.990385 |
| Bacillales_Incertae.Sedis.XI | Qualtrics.age | 0.160673 | -0.15945 | 0.990385 |
| Verrucomicrobiaceae | Weight.lbs | 0.161412 | 0.156348 | 0.990385 |
| Lachnospiraceae | EDEQ4.Eating | 0.16144 | -0.14201 | 0.990385 |
| Defluviitaleaceae | BMI | 0.161999 | -0.15579 | 0.990385 |
| Micrococcaceae | Mini.IPIP.Neuroticism | 0.162192 | 0.159064 | 0.990385 |
| Lachnospiraceae | EDEQ4.Weight | 0.16379 | -0.15743 | 0.990385 |
| Prevotellaceae | EDEQ4.Shape | 0.164543 | 0.148988 | 0.990385 |
| Fusobacteriaceae | EDEQ4.Weight | 0.164983 | 0.157332 | 0.990385 |
| Gracilibacteraceae | EDEQ4.Eating | 0.169661 | 0.147313 | 0.990385 |
| Clostridiaceae.1 | Mini.IPIP.Conscientiousness | 0.171009 | -0.16377 | 0.990385 |
| Lactobacillaceae | EDEQ4.Weight | 0.171766 | 0.150838 | 0.990385 |
| Victivallaceae | PSS.10.score | 0.173275 | -0.14611 | 0.990385 |
| Streptococcaceae | Mini.IPIP.Extraversion | 0.173501 | -0.15366 | 0.990385 |
| Peptostreptococcaceae | BAI.score | 0.177015 | -0.14732 | 0.990385 |
| Rhodospirillaceae | Height.inches | 0.180869 | 0.139277 | 0.990385 |
| Prevotellaceae | EDEQ4.Total | 0.181845 | 0.144748 | 0.990385 |
| Sutterellaceae | Mini.IPIP.Conscientiousness | 0.182837 | 0.142926 | 0.990385 |
| Chloroplast | EDEQ4.Eating | 0.182855 | -0.14297 | 0.990385 |
| shannonDiversity (family) | EDEQ4.Shape | 0.18315 | 0.148229 | 0.990385 |
| Corynebacteriaceae | Weight.lbs | 0.183327 | -0.14465 | 0.990385 |
| shannonDiversity (family) | BDI.II.score | 0.184453 | 0.147834 | 0.990385 |
| Pyrodictiaceae | PSS.10.score | 0.186359 | 0.148287 | 0.990385 |
| Corynebacteriaceae | PSS.10.score | 0.187217 | 0.148442 | 0.990385 |
| Enterococcaceae | Mini.IPIP.Imagination | 0.190443 | 0.138795 | 0.990385 |
| Sutterellaceae | Qualtrics.age | 0.190761 | 0.136331 | 0.990385 |
| Eubacteriaceae | Mini.IPIP.Extraversion | 0.191597 | -0.14123 | 0.990385 |
| Streptococcaceae | Mini.IPIP.Neuroticism | 0.191977 | 0.144117 | 0.990385 |
| Desulfovibrionaceae | EDEQ4.Weight | 0.193826 | 0.141613 | 0.990385 |
| shannonDiversity (family) | EDEQ4.Weight | 0.19519 | 0.152158 | 0.990385 |
| Clostridiales_Incertae.Sedis.XIII | BAI.score | 0.197988 | -0.13088 | 0.990385 |
| Pasteurellaceae | Mini.IPIP.Neuroticism | 0.198248 | 0.141494 | 0.990385 |
| Verrucomicrobiaceae | EDEQ4.Total | 0.199231 | 0.146426 | 0.990385 |
| Peptostreptococcaceae | Weight.lbs | 0.199589 | 0.153809 | 0.990385 |
| Eubacteriaceae | PSS.10.score | 0.199769 | -0.14744 | 0.990385 |
| Enterococcaceae | EDEQ4.Total | 0.200906 | -0.14665 | 0.990385 |
| Lactobacillaceae | Mini.IPIP.Agreeableness | 0.202048 | -0.14459 | 0.990385 |
| Rikenellaceae | EDEQ4.Restraint | 0.204273 | 0.135259 | 0.990385 |
| Verrucomicrobiaceae | BAI.score | 0.204493 | 0.135382 | 0.990385 |
| Micrococcaceae | EDEQ4.Weight | 0.206759 | -0.13509 | 0.990385 |
| Acidaminococcaceae | EDEQ4.Total | 0.207043 | 0.133508 | 0.990385 |
| Corynebacteriaceae | EDEQ4.Eating | 0.208197 | 0.141899 | 0.990385 |
| Peptostreptococcaceae | BMI | 0.21094 | 0.138944 | 0.990385 |
| Lachnospiraceae | Mini.IPIP.Conscientiousness | 0.210974 | 0.14332 | 0.990385 |
| Acidaminococcaceae | Qualtrics.age | 0.211471 | 0.132011 | 0.990385 |
| Methanobacteriaceae | EDEQ4.Restraint | 0.213288 | 0.138133 | 0.990385 |
| Gracilibacteraceae | Mini.IPIP.Imagination | 0.214566 | -0.13521 | 0.990385 |
| Clostridiales_Incertae.Sedis.XIII | Qualtrics.age | 0.215526 | 0.139473 | 0.990385 |
| Lactobacillaceae | Mini.IPIP.Neuroticism | 0.218224 | 0.136832 | 0.990385 |
| Acidaminococcaceae | BDI.II.score | 0.218304 | -0.13168 | 0.990385 |
| Veillonellaceae | EDEQ4.Shape | 0.219387 | -0.13191 | 0.990385 |
| Clostridiales_Incertae.Sedis.XI | BDI.II.score | 0.219413 | -0.126 | 0.990385 |
| Prevotellaceae | EDEQ4.Weight | 0.222194 | 0.134156 | 0.990385 |
| Leuconostocaceae | Mini.IPIP.Conscientiousness | 0.222762 | -0.13466 | 0.990385 |
| Incertae.Sedis.XI | EDEQ4.Eating | 0.223442 | -0.13023 | 0.990385 |
| Rikenellaceae | EDEQ4.Weight | 0.225034 | 0.12749 | 0.990385 |
| Rhodospirillaceae | EDEQ4.Shape | 0.225806 | -0.13288 | 0.990385 |
| Streptococcaceae | Height.inches | 0.226544 | -0.13723 | 0.990385 |
| Sutterellaceae | Mini.IPIP.Neuroticism | 0.226693 | -0.12828 | 0.990385 |
| Verrucomicrobiaceae | EDEQ4.Weight | 0.227901 | 0.140561 | 0.990385 |
| Pyrodictiaceae | Mini.IPIP.Extraversion | 0.232797 | -0.12534 | 0.990385 |
| Porphyromonadaceae | Mini.IPIP.Imagination | 0.234934 | -0.12823 | 0.990385 |
| Porphyromonadaceae | Weight.lbs | 0.235163 | 0.133771 | 0.990385 |
| Lachnospiraceae | Mini.IPIP.Neuroticism | 0.235303 | -0.13322 | 0.990385 |
| Desulfovibrionaceae | BAI.score | 0.23632 | 0.122835 | 0.990385 |
| Clostridiaceae.2 | EDEQ4.Shape | 0.236349 | 0.126852 | 0.990385 |
| Prevotellaceae | Weight.lbs | 0.238075 | -0.12476 | 0.990385 |
| Porphyromonadaceae | Height.inches | 0.23811 | 0.130352 | 0.990385 |
| Gracilibacteraceae | Mini.IPIP.Conscientiousness | 0.238943 | -0.12355 | 0.990385 |
| Verrucomicrobiaceae | Mini.IPIP.Conscientiousness | 0.239159 | 0.12232 | 0.990385 |
| Victivallaceae | Mini.IPIP.Conscientiousness | 0.239644 | 0.124449 | 0.990385 |
| Bifidobacteriaceae | PSS.10.score | 0.239732 | -0.12131 | 0.990385 |
| Veillonellaceae | Mini.IPIP.Neuroticism | 0.241171 | 0.138614 | 0.990385 |
| Gracilibacteraceae | Mini.IPIP.Neuroticism | 0.243122 | 0.121088 | 0.990385 |
| Desulfovibrionaceae | Mini.IPIP.Imagination | 0.243685 | -0.12985 | 0.990385 |
| Victivallaceae | Qualtrics.age | 0.246836 | 0.121234 | 0.990385 |
| Bacteroidaceae | Mini.IPIP.Neuroticism | 0.247141 | -0.13456 | 0.990385 |
| Micrococcaceae | BMI | 0.247553 | -0.11567 | 0.990385 |
| Enterococcaceae | BMI | 0.247869 | -0.12308 | 0.990385 |
| Pasteurellaceae | Mini.IPIP.Imagination | 0.252752 | -0.12639 | 0.990385 |
| Enterococcaceae | Weight.lbs | 0.2534 | -0.12241 | 0.990385 |
| Enterobacteriaceae | Height.inches | 0.254659 | -0.11662 | 0.990385 |
| Aerococcaceae | EDEQ4.Weight | 0.256577 | 0.128335 | 0.990385 |
| Gracilibacteraceae | BAI.score | 0.261665 | -0.11341 | 0.990385 |
| Rikenellaceae | EDEQ4.Total | 0.261709 | 0.12616 | 0.990385 |
| Clostridiaceae.2 | Mini.IPIP.Imagination | 0.263661 | 0.115179 | 0.990385 |
| Pyrodictiaceae | Qualtrics.age | 0.264052 | -0.12351 | 0.990385 |
| Bacillales_Incertae.Sedis.XI | EDEQ4.Total | 0.265217 | -0.10863 | 0.990385 |
| Fusobacteriaceae | PSS.10.score | 0.265744 | 0.119184 | 0.990385 |
| Incertae.Sedis.XI | Mini.IPIP.Conscientiousness | 0.267754 | -0.12891 | 0.990385 |
| Streptococcaceae | Mini.IPIP.Imagination | 0.268469 | -0.11474 | 0.990385 |
| Verrucomicrobiaceae | Mini.IPIP.Agreeableness | 0.270573 | -0.12219 | 0.990385 |
| Erysipelotrichaceae | BDI.II.score | 0.27222 | 0.122022 | 0.990385 |
| Enterobacteriaceae | EDEQ4.Shape | 0.276379 | 0.124873 | 0.990385 |
| Clostridiales_Incertae.Sedis.XI | Qualtrics.age | 0.276379 | -0.13039 | 0.990385 |
| Fusobacteriaceae | BDI.II.score | 0.276945 | -0.11819 | 0.990385 |
| Streptococcaceae | Weight.lbs | 0.278253 | -0.11512 | 0.990385 |
| Ruminococcaceae | PSS.10.score | 0.280082 | 0.122605 | 0.990385 |
| Oxalobacteraceae | Mini.IPIP.Imagination | 0.287066 | -0.11739 | 0.990385 |
| Erysipelotrichaceae | Height.inches | 0.288516 | -0.11981 | 0.990385 |
| Sutterellaceae | BDI.II.score | 0.288692 | -0.1167 | 0.990385 |
| Sutterellaceae | Mini.IPIP.Imagination | 0.291447 | 0.122929 | 0.990385 |
| Carnobacteriaceae | Mini.IPIP.Imagination | 0.294544 | 0.102396 | 0.990385 |
| Carnobacteriaceae | Qualtrics.age | 0.294621 | -0.11424 | 0.990385 |
| Ruminococcaceae | EDEQ4.Total | 0.297565 | 0.12162 | 0.990385 |
| Aerococcaceae | EDEQ4.Total | 0.297881 | 0.113086 | 0.990385 |
| Desulfovibrionaceae | Weight.lbs | 0.298182 | 0.11229 | 0.990385 |
| Campylobacteraceae | BMI | 0.298267 | 0.113607 | 0.990385 |
| Methanobacteriaceae | EDEQ4.Eating | 0.298672 | 0.112877 | 0.990385 |
| Desulfovibrionaceae | EDEQ4.Shape | 0.29967 | 0.110847 | 0.990385 |
| Erysipelotrichaceae | BAI.score | 0.301703 | 0.104063 | 0.990385 |
| Eubacteriaceae | Qualtrics.age | 0.301741 | -0.12928 | 0.990385 |
| Chloroplast | Mini.IPIP.Neuroticism | 0.303638 | -0.11369 | 0.990385 |
| Chloroplast | Height.inches | 0.303757 | 0.103014 | 0.990385 |
| Incertae.Sedis.XI | PSS.10.score | 0.305473 | -0.10864 | 0.990385 |
| Ruminococcaceae | Mini.IPIP.Extraversion | 0.306488 | -0.10677 | 0.990385 |
| Enterococcaceae | EDEQ4.Weight | 0.306709 | -0.10877 | 0.990385 |
| shannonDiversity (family) | Mini.IPIP.Conscientiousness | 0.310232 | -0.11304 | 0.990385 |
| Rhodospirillaceae | BAI.score | 0.31932 | -0.11808 | 0.990385 |
| Oxalobacteraceae | BAI.score | 0.31937 | -0.10738 | 0.990385 |
| Veillonellaceae | BDI.II.score | 0.320003 | 0.096401 | 0.990385 |
| Chitinophagaceae | BMI | 0.321558 | 0.100076 | 0.990385 |
| Streptococcaceae | Qualtrics.age | 0.324661 | -0.1106 | 0.990385 |
| Rikenellaceae | Weight.lbs | 0.326426 | 0.111222 | 0.990385 |
| Enterobacteriaceae | Mini.IPIP.Imagination | 0.326477 | -0.10828 | 0.990385 |
| Pyrodictiaceae | EDEQ4.Shape | 0.327415 | 0.106666 | 0.990385 |
| Coriobacteriaceae | Mini.IPIP.Extraversion | 0.327838 | -0.10433 | 0.990385 |
| Prevotellaceae | Qualtrics.age | 0.328285 | -0.09738 | 0.990385 |
| shannonDiversity (family) | EDEQ4.Eating | 0.328611 | 0.102848 | 0.990385 |
| Campylobacteraceae | Qualtrics.age | 0.328693 | -0.11404 | 0.990385 |
| Coriobacteriaceae | BAI.score | 0.33017 | 0.093554 | 0.990385 |
| Acidaminococcaceae | EDEQ4.Weight | 0.333611 | 0.109062 | 0.990385 |
| Lactobacillaceae | EDEQ4.Eating | 0.338707 | 0.104314 | 0.990385 |
| Rhodospirillaceae | Mini.IPIP.Neuroticism | 0.340176 | -0.10544 | 0.990385 |
| Actinomycetaceae | Height.inches | 0.345661 | -0.10006 | 0.990385 |
| Peptostreptococcaceae | BDI.II.score | 0.345836 | 0.104455 | 0.990385 |
| Ruminococcaceae | EDEQ4.Shape | 0.346529 | 0.11923 | 0.990385 |
| Enterobacteriaceae | EDEQ4.Weight | 0.346815 | 0.101317 | 0.990385 |
| Methanobacteriaceae | Weight.lbs | 0.347848 | 0.097056 | 0.990385 |
| Enterococcaceae | EDEQ4.Eating | 0.348038 | -0.10189 | 0.990385 |
| Victivallaceae | Mini.IPIP.Agreeableness | 0.348712 | 0.103984 | 0.990385 |
| Clostridiales_Incertae.Sedis.XI | EDEQ4.Eating | 0.350992 | -0.1004 | 0.990385 |
| Pasteurellaceae | Mini.IPIP.Conscientiousness | 0.352352 | -0.10265 | 0.990385 |
| Chloroplast | Mini.IPIP.Conscientiousness | 0.354391 | 0.096741 | 0.990385 |
| Coriobacteriaceae | EDEQ4.Restraint | 0.355876 | 0.099497 | 0.990385 |
| Clostridiaceae.1 | Mini.IPIP.Imagination | 0.356444 | -0.10245 | 0.990385 |
| Enterococcaceae | BAI.score | 0.357183 | -0.09704 | 0.990385 |
| Erysipelotrichaceae | EDEQ4.Eating | 0.35761 | 0.100344 | 0.990385 |
| Actinomycetaceae | Mini.IPIP.Extraversion | 0.357771 | -0.10072 | 0.990385 |
| Eubacteriaceae | BAI.score | 0.358383 | -0.09786 | 0.990385 |
| Methanobacteriaceae | BDI.II.score | 0.358705 | 0.10593 | 0.990385 |
| Enterobacteriaceae | Mini.IPIP.Neuroticism | 0.358723 | 0.094312 | 0.990385 |
| Oxalobacteraceae | Weight.lbs | 0.359818 | 0.097192 | 0.990385 |
| Micrococcaceae | Height.inches | 0.360716 | 0.107511 | 0.990385 |
| Verrucomicrobiaceae | PSS.10.score | 0.362224 | 0.108272 | 0.990385 |
| Verrucomicrobiaceae | EDEQ4.Restraint | 0.36395 | 0.100041 | 0.990385 |
| Acidaminococcaceae | Mini.IPIP.Conscientiousness | 0.365976 | 0.094777 | 0.990385 |
| Leuconostocaceae | EDEQ4.Eating | 0.367092 | 0.097324 | 0.990385 |
| Leuconostocaceae | BDI.II.score | 0.367471 | 0.093552 | 0.990385 |
| Micrococcaceae | Mini.IPIP.Conscientiousness | 0.367648 | -0.09603 | 0.990385 |
| Clostridiales_Incertae.Sedis.XI | Mini.IPIP.Imagination | 0.367816 | -0.09175 | 0.990385 |
| Chitinophagaceae | Mini.IPIP.Agreeableness | 0.369476 | 0.09677 | 0.990385 |
| Clostridiales_Incertae.Sedis.XIII | Weight.lbs | 0.36982 | 0.094605 | 0.990385 |
| Pasteurellaceae | EDEQ4.Weight | 0.3702 | -0.09749 | 0.990385 |
| Sutterellaceae | Mini.IPIP.Agreeableness | 0.370945 | 0.089245 | 0.990385 |
| Victivallaceae | EDEQ4.Total | 0.370967 | 0.09219 | 0.990385 |
| Leuconostocaceae | PSS.10.score | 0.374393 | 0.082791 | 0.990385 |
| Corynebacteriaceae | EDEQ4.Shape | 0.374838 | 0.094172 | 0.990385 |
| Clostridiaceae.2 | BDI.II.score | 0.374897 | 0.103619 | 0.990385 |
| shannonDiversity (family) | BMI | 0.376627 | 0.093748 | 0.990385 |
| Lachnospiraceae | BDI.II.score | 0.376941 | -0.09805 | 0.990385 |
| Verrucomicrobiaceae | EDEQ4.Shape | 0.377501 | 0.087006 | 0.990385 |
| Peptostreptococcaceae | Qualtrics.age | 0.377765 | -0.10168 | 0.990385 |
| Leuconostocaceae | BAI.score | 0.378521 | 0.091818 | 0.990385 |
| Victivallaceae | BMI | 0.380434 | 0.096018 | 0.990385 |
| Pyrodictiaceae | Mini.IPIP.Conscientiousness | 0.382427 | -0.0997 | 0.990385 |
| Prevotellaceae | BAI.score | 0.384031 | 0.09031 | 0.990385 |
| shannonDiversity (family) | Weight.lbs | 0.387739 | 0.100646 | 0.990385 |
| Acidaminococcaceae | Weight.lbs | 0.387739 | 0.097358 | 0.990385 |
| Erysipelotrichaceae | Mini.IPIP.Conscientiousness | 0.389923 | -0.09213 | 0.990385 |
| Rhodospirillaceae | BMI | 0.390246 | -0.0952 | 0.990385 |
| Leuconostocaceae | Mini.IPIP.Neuroticism | 0.391068 | 0.092883 | 0.990385 |
| Verrucomicrobiaceae | Mini.IPIP.Neuroticism | 0.3925 | 0.099234 | 0.990385 |
| Bacteroidaceae | EDEQ4.Restraint | 0.3931 | 0.094168 | 0.990385 |
| Ruminococcaceae | Mini.IPIP.Conscientiousness | 0.394005 | -0.08848 | 0.990385 |
| Clostridiales_Incertae.Sedis.XI | EDEQ4.Total | 0.396552 | -0.0779 | 0.990385 |
| Aerococcaceae | Mini.IPIP.Conscientiousness | 0.397183 | 0.093115 | 0.990385 |
| Prevotellaceae | Mini.IPIP.Extraversion | 0.397524 | 0.090333 | 0.990385 |
| Enterococcaceae | BDI.II.score | 0.398155 | -0.09069 | 0.990385 |
| Enterococcaceae | EDEQ4.Restraint | 0.399482 | -0.09064 | 0.990385 |
| Actinomycetaceae | Weight.lbs | 0.399971 | -0.09665 | 0.990385 |
| Chloroplast | Mini.IPIP.Imagination | 0.400567 | 0.077707 | 0.990385 |
| Desulfovibrionaceae | Mini.IPIP.Extraversion | 0.403622 | -0.08785 | 0.990385 |
| Micrococcaceae | Mini.IPIP.Agreeableness | 0.405211 | -0.09619 | 0.990385 |
| Campylobacteraceae | BAI.score | 0.405381 | 0.091496 | 0.990385 |
| Coriobacteriaceae | BDI.II.score | 0.405527 | 0.085281 | 0.990385 |
| Oxalobacteraceae | EDEQ4.Restraint | 0.405772 | 0.090947 | 0.990385 |
| Leuconostocaceae | EDEQ4.Weight | 0.405946 | 0.091313 | 0.990385 |
| Victivallaceae | Mini.IPIP.Neuroticism | 0.412987 | -0.08798 | 0.990385 |
| Bifidobacteriaceae | Height.inches | 0.413756 | 0.096063 | 0.990385 |
| Peptostreptococcaceae | EDEQ4.Shape | 0.414316 | 0.092662 | 0.990385 |
| Pyrodictiaceae | EDEQ4.Weight | 0.414778 | -0.09173 | 0.990385 |
| Corynebacteriaceae | EDEQ4.Restraint | 0.414986 | 0.086335 | 0.990385 |
| Eubacteriaceae | Weight.lbs | 0.418746 | -0.08351 | 0.990385 |
| Prevotellaceae | Mini.IPIP.Conscientiousness | 0.419034 | -0.09036 | 0.990385 |
| Clostridiaceae.1 | Mini.IPIP.Neuroticism | 0.421691 | 0.091845 | 0.990385 |
| Bacillales_Incertae.Sedis.XI | BDI.II.score | 0.423157 | -0.07907 | 0.990385 |
| Chloroplast | Weight.lbs | 0.423293 | 0.088784 | 0.990385 |
| Sutterellaceae | EDEQ4.Restraint | 0.423623 | 0.083558 | 0.990385 |
| Actinomycetaceae | BAI.score | 0.425612 | 0.094195 | 0.990385 |
| Methanobacteriaceae | Qualtrics.age | 0.425702 | 0.086628 | 0.990385 |
| Clostridiaceae.1 | Qualtrics.age | 0.427301 | 0.081822 | 0.990385 |
| Methanobacteriaceae | Mini.IPIP.Conscientiousness | 0.428229 | 0.093239 | 0.990385 |
| Peptostreptococcaceae | EDEQ4.Weight | 0.430884 | -0.09855 | 0.990385 |
| Acidaminococcaceae | Mini.IPIP.Extraversion | 0.431167 | 0.090333 | 0.990385 |
| Ruminococcaceae | Qualtrics.age | 0.431593 | 0.087794 | 0.990385 |
| Coriobacteriaceae | EDEQ4.Shape | 0.435655 | 0.081597 | 0.990385 |
| Carnobacteriaceae | PSS.10.score | 0.435775 | 0.09115 | 0.990385 |
| Rikenellaceae | Mini.IPIP.Imagination | 0.438567 | -0.07934 | 0.990385 |
| Lachnospiraceae | BMI | 0.438889 | -0.06473 | 0.990385 |
| Pasteurellaceae | Height.inches | 0.439485 | 0.07379 | 0.990385 |
| Campylobacteraceae | EDEQ4.Restraint | 0.440102 | -0.08033 | 0.990385 |
| Veillonellaceae | PSS.10.score | 0.440396 | 0.082254 | 0.990385 |
| Carnobacteriaceae | EDEQ4.Total | 0.440872 | -0.0841 | 0.990385 |
| Erysipelotrichaceae | Mini.IPIP.Imagination | 0.44294 | -0.09039 | 0.990385 |
| Eubacteriaceae | BDI.II.score | 0.446325 | -0.07218 | 0.990385 |
| Actinomycetaceae | Mini.IPIP.Imagination | 0.447337 | -0.06449 | 0.990385 |
| Verrucomicrobiaceae | Mini.IPIP.Imagination | 0.447337 | -0.07866 | 0.990385 |
| Pyrodictiaceae | Height.inches | 0.44875 | 0.079874 | 0.990385 |
| Lachnospiraceae | PSS.10.score | 0.449152 | -0.08273 | 0.990385 |
| Chitinophagaceae | EDEQ4.Shape | 0.450565 | 0.08263 | 0.990385 |
| Fusobacteriaceae | Weight.lbs | 0.453062 | -0.07693 | 0.990385 |
| Bifidobacteriaceae | Mini.IPIP.Imagination | 0.456207 | 0.083886 | 0.990385 |
| Clostridiaceae.1 | PSS.10.score | 0.46247 | 0.080319 | 0.990385 |
| Chitinophagaceae | EDEQ4.Total | 0.462955 | 0.082031 | 0.990385 |
| Enterobacteriaceae | EDEQ4.Total | 0.463185 | 0.083436 | 0.990385 |
| Carnobacteriaceae | EDEQ4.Weight | 0.464098 | -0.08981 | 0.990385 |
| Clostridiaceae.2 | PSS.10.score | 0.466909 | 0.079385 | 0.990385 |
| Bacteroidaceae | Mini.IPIP.Agreeableness | 0.468675 | 0.079365 | 0.990385 |
| Clostridiaceae.2 | Mini.IPIP.Conscientiousness | 0.469492 | -0.07955 | 0.990385 |
| Oxalobacteraceae | Mini.IPIP.Neuroticism | 0.474041 | -0.07736 | 0.990385 |
| Porphyromonadaceae | Mini.IPIP.Extraversion | 0.475458 | 0.079229 | 0.990385 |
| Clostridiales_Incertae.Sedis.XIII | EDEQ4.Restraint | 0.476664 | 0.083965 | 0.990385 |
| Micrococcaceae | Mini.IPIP.Extraversion | 0.47747 | 0.074005 | 0.990385 |
| shannonDiversity (family) | Mini.IPIP.Agreeableness | 0.477798 | -0.07807 | 0.990385 |
| shannonDiversity (family) | Mini.IPIP.Imagination | 0.478808 | -0.09158 | 0.990385 |
| Bacteroidaceae | Mini.IPIP.Extraversion | 0.480021 | 0.067878 | 0.990385 |
| Veillonellaceae | Mini.IPIP.Extraversion | 0.480021 | 0.083058 | 0.990385 |
| Peptostreptococcaceae | Mini.IPIP.Conscientiousness | 0.480947 | -0.0801 | 0.990385 |
| Actinomycetaceae | EDEQ4.Weight | 0.485124 | 0.091912 | 0.990385 |
| Desulfovibrionaceae | PSS.10.score | 0.487367 | 0.072287 | 0.990385 |
| Incertae.Sedis.XI | EDEQ4.Shape | 0.489991 | 0.070914 | 0.990385 |
| Veillonellaceae | Mini.IPIP.Conscientiousness | 0.490182 | 0.073796 | 0.990385 |
| Leuconostocaceae | EDEQ4.Total | 0.491897 | 0.08552 | 0.990385 |
| Prevotellaceae | BDI.II.score | 0.494028 | 0.080974 | 0.990385 |
| Defluviitaleaceae | EDEQ4.Weight | 0.494558 | -0.07057 | 0.990385 |
| Chloroplast | Qualtrics.age | 0.494873 | 0.071092 | 0.990385 |
| Clostridiaceae.2 | Mini.IPIP.Extraversion | 0.496129 | 0.069877 | 0.990385 |
| Desulfovibrionaceae | Height.inches | 0.500824 | 0.073515 | 0.990385 |
| Defluviitaleaceae | EDEQ4.Restraint | 0.501104 | 0.0707 | 0.990385 |
| Lactobacillaceae | PSS.10.score | 0.501294 | -0.08007 | 0.990385 |
| Peptostreptococcaceae | Height.inches | 0.503257 | 0.073549 | 0.990385 |
| Ruminococcaceae | EDEQ4.Restraint | 0.503267 | 0.082088 | 0.990385 |
| Streptococcaceae | EDEQ4.Shape | 0.503344 | -0.06935 | 0.990385 |
| Coriobacteriaceae | EDEQ4.Total | 0.504422 | 0.059611 | 0.990385 |
| Micrococcaceae | BAI.score | 0.506475 | 0.06897 | 0.990385 |
| Porphyromonadaceae | EDEQ4.Shape | 0.508047 | -0.06679 | 0.990385 |
| Methanobacteriaceae | EDEQ4.Weight | 0.508681 | 0.073517 | 0.990385 |
| Campylobacteraceae | PSS.10.score | 0.509481 | 0.074706 | 0.990385 |
| Campylobacteraceae | BDI.II.score | 0.509737 | 0.068636 | 0.990385 |
| Carnobacteriaceae | BMI | 0.513493 | -0.08493 | 0.990385 |
| Streptococcaceae | EDEQ4.Total | 0.513836 | -0.07366 | 0.990385 |
| Pyrodictiaceae | BAI.score | 0.515277 | 0.070199 | 0.990385 |
| Aerococcaceae | BMI | 0.515808 | 0.077029 | 0.990385 |
| Clostridiaceae.2 | Mini.IPIP.Neuroticism | 0.518879 | 0.071333 | 0.990385 |
| Aerococcaceae | EDEQ4.Eating | 0.520208 | 0.068013 | 0.990385 |
| Lachnospiraceae | Mini.IPIP.Extraversion | 0.522136 | 0.073069 | 0.990385 |
| Leuconostocaceae | Qualtrics.age | 0.524258 | 0.067449 | 0.990385 |
| Rikenellaceae | Qualtrics.age | 0.527312 | 0.061376 | 0.990385 |
| Enterobacteriaceae | Weight.lbs | 0.529654 | -0.07452 | 0.990385 |
| Veillonellaceae | Weight.lbs | 0.529678 | -0.06264 | 0.990385 |
| Aerococcaceae | EDEQ4.Shape | 0.531703 | 0.068023 | 0.990385 |
| Gracilibacteraceae | Mini.IPIP.Extraversion | 0.532974 | -0.05929 | 0.990385 |
| Methanobacteriaceae | BMI | 0.532981 | 0.071252 | 0.990385 |
| Fusobacteriaceae | Height.inches | 0.537347 | -0.06047 | 0.990385 |
| Clostridiales_Incertae.Sedis.XI | Mini.IPIP.Neuroticism | 0.537967 | -0.0753 | 0.990385 |
| Campylobacteraceae | Mini.IPIP.Imagination | 0.539056 | -0.06817 | 0.990385 |
| Clostridiales_Incertae.Sedis.XI | Mini.IPIP.Agreeableness | 0.539156 | 0.07204 | 0.990385 |
| Lactobacillaceae | BDI.II.score | 0.539355 | 0.065626 | 0.990385 |
| Actinomycetaceae | BMI | 0.540106 | -0.06015 | 0.990385 |
| Carnobacteriaceae | Mini.IPIP.Agreeableness | 0.541603 | -0.07596 | 0.990385 |
| Corynebacteriaceae | Mini.IPIP.Extraversion | 0.543182 | 0.067249 | 0.990385 |
| Chitinophagaceae | Mini.IPIP.Conscientiousness | 0.543749 | -0.07408 | 0.990385 |
| Bacillales_Incertae.Sedis.XI | EDEQ4.Shape | 0.543951 | -0.05899 | 0.990385 |
| Rikenellaceae | Mini.IPIP.Conscientiousness | 0.547533 | -0.07174 | 0.990385 |
| Acidaminococcaceae | BMI | 0.549854 | 0.082743 | 0.990385 |
| Acidaminococcaceae | Mini.IPIP.Imagination | 0.550133 | -0.06721 | 0.990385 |
| Streptococcaceae | EDEQ4.Eating | 0.552318 | -0.06326 | 0.990385 |
| Clostridiaceae.2 | EDEQ4.Total | 0.55234 | 0.066856 | 0.990385 |
| Porphyromonadaceae | EDEQ4.Restraint | 0.553569 | 0.058042 | 0.990385 |
| Clostridiales_Incertae.Sedis.XIII | PSS.10.score | 0.554181 | -0.06342 | 0.990385 |
| Pasteurellaceae | EDEQ4.Total | 0.557226 | -0.05199 | 0.990385 |
| Eubacteriaceae | EDEQ4.Shape | 0.558772 | 0.062968 | 0.990385 |
| Coriobacteriaceae | Weight.lbs | 0.558956 | 0.049702 | 0.990385 |
| Peptostreptococcaceae | Mini.IPIP.Neuroticism | 0.560333 | 0.067819 | 0.990385 |
| Campylobacteraceae | EDEQ4.Eating | 0.561129 | 0.063488 | 0.990385 |
| Desulfovibrionaceae | BMI | 0.562087 | 0.069502 | 0.990385 |
| Bacillales_Incertae.Sedis.XI | BAI.score | 0.5627 | 0.063449 | 0.990385 |
| Micrococcaceae | EDEQ4.Restraint | 0.56369 | -0.06158 | 0.990385 |
| Defluviitaleaceae | EDEQ4.Shape | 0.564451 | -0.06153 | 0.990385 |
| Rhodospirillaceae | BDI.II.score | 0.564857 | 0.056167 | 0.990385 |
| Porphyromonadaceae | Mini.IPIP.Neuroticism | 0.565308 | -0.06941 | 0.990385 |
| Fusobacteriaceae | EDEQ4.Restraint | 0.566014 | -0.05995 | 0.990385 |
| Lactobacillaceae | BMI | 0.567047 | -0.06015 | 0.990385 |
| Defluviitaleaceae | Mini.IPIP.Neuroticism | 0.571124 | -0.05758 | 0.990385 |
| Chloroplast | EDEQ4.Weight | 0.574019 | -0.07106 | 0.990385 |
| Veillonellaceae | BAI.score | 0.575032 | -0.0681 | 0.990385 |
| Fusobacteriaceae | EDEQ4.Total | 0.575732 | 0.059743 | 0.990385 |
| Leuconostocaceae | Mini.IPIP.Extraversion | 0.575782 | -0.06012 | 0.990385 |
| Streptococcaceae | Mini.IPIP.Conscientiousness | 0.577403 | 0.059772 | 0.990385 |
| Aerococcaceae | PSS.10.score | 0.579155 | 0.054256 | 0.990385 |
| Desulfovibrionaceae | EDEQ4.Eating | 0.579799 | 0.061541 | 0.990385 |
| Pasteurellaceae | EDEQ4.Eating | 0.582589 | 0.058607 | 0.990385 |
| Carnobacteriaceae | EDEQ4.Eating | 0.585236 | 0.060373 | 0.990385 |
| Enterococcaceae | Height.inches | 0.585551 | -0.05598 | 0.990385 |
| Actinomycetaceae | EDEQ4.Shape | 0.586294 | -0.06289 | 0.990385 |
| Aerococcaceae | Mini.IPIP.Imagination | 0.587838 | -0.06655 | 0.990385 |
| Enterococcaceae | EDEQ4.Shape | 0.59031 | -0.05694 | 0.990385 |
| Peptostreptococcaceae | EDEQ4.Eating | 0.591058 | 0.067307 | 0.990385 |
| shannonDiversity (family) | PSS.10.score | 0.591686 | 0.056546 | 0.990385 |
| Bacteroidaceae | BDI.II.score | 0.591929 | 0.061553 | 0.990385 |
| Victivallaceae | Height.inches | 0.592201 | 0.06708 | 0.990385 |
| Streptococcaceae | Mini.IPIP.Agreeableness | 0.594459 | -0.0615 | 0.990385 |
| Bifidobacteriaceae | EDEQ4.Restraint | 0.595509 | 0.055263 | 0.990385 |
| Bacillales_Incertae.Sedis.XI | Mini.IPIP.Imagination | 0.595903 | -0.06145 | 0.990385 |
| Ruminococcaceae | BDI.II.score | 0.597049 | -0.04884 | 0.990385 |
| Leuconostocaceae | BMI | 0.597148 | -0.05934 | 0.990385 |
| Bifidobacteriaceae | Weight.lbs | 0.599176 | 0.081001 | 0.990385 |
| Methanobacteriaceae | BAI.score | 0.599269 | 0.051331 | 0.990385 |
| Streptococcaceae | BMI | 0.599857 | -0.05978 | 0.990385 |
| Aerococcaceae | Mini.IPIP.Extraversion | 0.601684 | -0.0648 | 0.990385 |
| Aerococcaceae | Weight.lbs | 0.60198 | -0.06929 | 0.990385 |
| Incertae.Sedis.XI | Weight.lbs | 0.602728 | 0.053329 | 0.990385 |
| Bacillales_Incertae.Sedis.XI | Mini.IPIP.Neuroticism | 0.603975 | -0.05443 | 0.990385 |
| Coriobacteriaceae | Mini.IPIP.Conscientiousness | 0.60802 | 0.059405 | 0.990385 |
| Methanobacteriaceae | Mini.IPIP.Extraversion | 0.608086 | 0.049684 | 0.990385 |
| Oxalobacteraceae | PSS.10.score | 0.608579 | -0.05498 | 0.990385 |
| Corynebacteriaceae | EDEQ4.Total | 0.610343 | 0.060071 | 0.990385 |
| Peptostreptococcaceae | Mini.IPIP.Extraversion | 0.611712 | 0.05798 | 0.990385 |
| Veillonellaceae | EDEQ4.Total | 0.612742 | -0.06168 | 0.990385 |
| Desulfovibrionaceae | Mini.IPIP.Conscientiousness | 0.613127 | -0.05528 | 0.990385 |
| Incertae.Sedis.XI | BAI.score | 0.615324 | -0.04438 | 0.990385 |
| Eubacteriaceae | Mini.IPIP.Conscientiousness | 0.615783 | -0.04955 | 0.990385 |
| Lactobacillaceae | Mini.IPIP.Imagination | 0.615845 | -0.05994 | 0.990385 |
| Oxalobacteraceae | Mini.IPIP.Extraversion | 0.616656 | 0.051232 | 0.990385 |
| Leuconostocaceae | EDEQ4.Shape | 0.619627 | 0.066838 | 0.990385 |
| Prevotellaceae | Mini.IPIP.Imagination | 0.621109 | 0.053869 | 0.990385 |
| Enterobacteriaceae | Qualtrics.age | 0.622373 | 0.050922 | 0.990385 |
| Gracilibacteraceae | Mini.IPIP.Agreeableness | 0.624628 | -0.05299 | 0.990385 |
| Pasteurellaceae | EDEQ4.Restraint | 0.625097 | -0.04913 | 0.990385 |
| Coriobacteriaceae | EDEQ4.Eating | 0.625226 | 0.055463 | 0.990385 |
| Porphyromonadaceae | BAI.score | 0.626339 | -0.05778 | 0.990385 |
| shannonDiversity (family) | Mini.IPIP.Neuroticism | 0.6266 | 0.05067 | 0.990385 |
| Chitinophagaceae | EDEQ4.Restraint | 0.630852 | 0.051036 | 0.990385 |
| Gracilibacteraceae | BDI.II.score | 0.631118 | 0.055287 | 0.990385 |
| Bacillales_Incertae.Sedis.XI | EDEQ4.Eating | 0.632702 | -0.05244 | 0.990385 |
| Enterobacteriaceae | EDEQ4.Eating | 0.633882 | 0.053703 | 0.990385 |
| Methanobacteriaceae | Height.inches | 0.635407 | 0.051257 | 0.990385 |
| Veillonellaceae | EDEQ4.Eating | 0.636801 | 0.041802 | 0.990385 |
| Bacteroidaceae | BAI.score | 0.636833 | 0.054501 | 0.990385 |
| Oxalobacteraceae | EDEQ4.Total | 0.64048 | 0.051478 | 0.990385 |
| Porphyromonadaceae | BMI | 0.64128 | 0.055326 | 0.990385 |
| Erysipelotrichaceae | Mini.IPIP.Neuroticism | 0.642359 | 0.043684 | 0.990385 |
| Prevotellaceae | Mini.IPIP.Neuroticism | 0.642359 | 0.045195 | 0.990385 |
| Carnobacteriaceae | EDEQ4.Shape | 0.642935 | -0.0503 | 0.990385 |
| Leuconostocaceae | Mini.IPIP.Agreeableness | 0.644217 | -0.04925 | 0.990385 |
| Sutterellaceae | Weight.lbs | 0.645926 | 0.066589 | 0.990385 |
| Gracilibacteraceae | PSS.10.score | 0.646155 | 0.05254 | 0.990385 |
| Pyrodictiaceae | EDEQ4.Eating | 0.646789 | 0.051647 | 0.990385 |
| Clostridiaceae.2 | EDEQ4.Eating | 0.648435 | 0.049106 | 0.990385 |
| Victivallaceae | Mini.IPIP.Imagination | 0.649714 | -0.0524 | 0.990385 |
| Incertae.Sedis.XI | Mini.IPIP.Extraversion | 0.649973 | -0.04387 | 0.990385 |
| Clostridiales_Incertae.Sedis.XIII | Mini.IPIP.Neuroticism | 0.650298 | -0.05496 | 0.990385 |
| Bacteroidaceae | EDEQ4.Weight | 0.651484 | 0.048909 | 0.990385 |
| Bacteroidaceae | BMI | 0.65182 | 0.051114 | 0.990385 |
| Actinomycetaceae | Qualtrics.age | 0.653893 | 0.036637 | 0.990385 |
| Bifidobacteriaceae | Mini.IPIP.Conscientiousness | 0.655248 | 0.045949 | 0.990385 |
| Sutterellaceae | EDEQ4.Weight | 0.656872 | -0.04763 | 0.990385 |
| Bacillales_Incertae.Sedis.XI | BMI | 0.661158 | -0.04749 | 0.990385 |
| Clostridiales_Incertae.Sedis.XIII | EDEQ4.Total | 0.662491 | 0.061927 | 0.990385 |
| Rhodospirillaceae | Qualtrics.age | 0.662491 | 0.048876 | 0.990385 |
| Chitinophagaceae | Mini.IPIP.Extraversion | 0.664711 | -0.0424 | 0.990385 |
| Aerococcaceae | EDEQ4.Restraint | 0.667423 | 0.045652 | 0.990385 |
| Clostridiaceae.2 | Mini.IPIP.Agreeableness | 0.667987 | 0.042133 | 0.990385 |
| Fusobacteriaceae | Mini.IPIP.Extraversion | 0.668704 | 0.055806 | 0.990385 |
| Clostridiaceae.1 | EDEQ4.Total | 0.670494 | 0.056147 | 0.990385 |
| Eubacteriaceae | Mini.IPIP.Agreeableness | 0.673408 | 0.058227 | 0.990385 |
| Defluviitaleaceae | Mini.IPIP.Imagination | 0.673463 | 0.047816 | 0.990385 |
| Aerococcaceae | Mini.IPIP.Agreeableness | 0.674173 | 0.053931 | 0.990385 |
| Campylobacteraceae | Height.inches | 0.675027 | -0.04241 | 0.990385 |
| Clostridiales_Incertae.Sedis.XI | PSS.10.score | 0.677844 | -0.06316 | 0.990385 |
| Clostridiales_Incertae.Sedis.XI | BMI | 0.678285 | -0.03436 | 0.990385 |
| Porphyromonadaceae | EDEQ4.Weight | 0.678602 | 0.052088 | 0.990385 |
| Lactobacillaceae | Height.inches | 0.678674 | -0.04668 | 0.990385 |
| Prevotellaceae | Mini.IPIP.Agreeableness | 0.678817 | -0.0437 | 0.990385 |
| Chitinophagaceae | EDEQ4.Weight | 0.679414 | 0.055067 | 0.990385 |
| Acidaminococcaceae | EDEQ4.Restraint | 0.683443 | 0.046449 | 0.990385 |
| Fusobacteriaceae | BMI | 0.684222 | -0.03862 | 0.990385 |
| Peptostreptococcaceae | Mini.IPIP.Agreeableness | 0.68424 | 0.057088 | 0.990385 |
| Enterococcaceae | PSS.10.score | 0.684483 | -0.04165 | 0.990385 |
| Campylobacteraceae | Mini.IPIP.Neuroticism | 0.685602 | 0.045163 | 0.990385 |
| Incertae.Sedis.XI | EDEQ4.Restraint | 0.686015 | 0.040977 | 0.990385 |
| Defluviitaleaceae | EDEQ4.Total | 0.686045 | -0.03302 | 0.990385 |
| Peptostreptococcaceae | PSS.10.score | 0.686115 | 0.041237 | 0.990385 |
| Clostridiales_Incertae.Sedis.XIII | BMI | 0.686546 | -0.04335 | 0.990385 |
| Clostridiales_Incertae.Sedis.XIII | EDEQ4.Eating | 0.686963 | -0.04424 | 0.990385 |
| Eubacteriaceae | Mini.IPIP.Imagination | 0.687636 | -0.054 | 0.990385 |
| Enterococcaceae | Mini.IPIP.Agreeableness | 0.688377 | -0.04534 | 0.990385 |
| shannonDiversity (family) | BAI.score | 0.690364 | 0.043414 | 0.990385 |
| Pyrodictiaceae | BMI | 0.691567 | -0.04395 | 0.990385 |
| Incertae.Sedis.XI | BDI.II.score | 0.691708 | -0.04328 | 0.990385 |
| Chloroplast | Mini.IPIP.Agreeableness | 0.696746 | -0.03625 | 0.990385 |
| Carnobacteriaceae | BDI.II.score | 0.700457 | 0.040849 | 0.990385 |
| Lactobacillaceae | BAI.score | 0.701217 | 0.039224 | 0.990385 |
| Micrococcaceae | EDEQ4.Eating | 0.701838 | 0.041814 | 0.990385 |
| Rikenellaceae | BDI.II.score | 0.70337 | -0.05158 | 0.990385 |
| Oxalobacteraceae | BMI | 0.705043 | -0.04612 | 0.990385 |
| Veillonellaceae | BMI | 0.705532 | -0.03765 | 0.990385 |
| Acidaminococcaceae | BAI.score | 0.706746 | 0.059587 | 0.990385 |
| Chloroplast | EDEQ4.Total | 0.707209 | -0.04149 | 0.990385 |
| Sutterellaceae | EDEQ4.Eating | 0.708044 | 0.039358 | 0.990385 |
| Chloroplast | Mini.IPIP.Extraversion | 0.709155 | -0.04263 | 0.990385 |
| Chitinophagaceae | Mini.IPIP.Neuroticism | 0.710921 | 0.049287 | 0.990385 |
| Clostridiaceae.1 | BAI.score | 0.712237 | -0.04951 | 0.990385 |
| Chloroplast | BMI | 0.712639 | 0.037156 | 0.990385 |
| Erysipelotrichaceae | PSS.10.score | 0.713326 | 0.033732 | 0.990385 |
| Clostridiales_Incertae.Sedis.XIII | Mini.IPIP.Agreeableness | 0.714352 | -0.03996 | 0.990385 |
| Fusobacteriaceae | Mini.IPIP.Agreeableness | 0.715026 | 0.044941 | 0.990385 |
| Fusobacteriaceae | Mini.IPIP.Imagination | 0.71563 | 0.042902 | 0.990385 |
| Streptococcaceae | EDEQ4.Weight | 0.717271 | -0.04222 | 0.990385 |
| Lachnospiraceae | BAI.score | 0.717744 | -0.03817 | 0.990385 |
| Verrucomicrobiaceae | Mini.IPIP.Extraversion | 0.71848 | -0.04438 | 0.990385 |
| Clostridiaceae.1 | EDEQ4.Shape | 0.718579 | 0.05258 | 0.990385 |
| Clostridiales_Incertae.Sedis.XIII | EDEQ4.Weight | 0.720061 | 0.045386 | 0.990385 |
| Veillonellaceae | EDEQ4.Weight | 0.722858 | -0.04066 | 0.990385 |
| Bifidobacteriaceae | BDI.II.score | 0.725438 | 0.04214 | 0.990385 |
| Oxalobacteraceae | Qualtrics.age | 0.725649 | 0.042131 | 0.990385 |
| Bifidobacteriaceae | EDEQ4.Weight | 0.728459 | -0.04038 | 0.990385 |
| Erysipelotrichaceae | EDEQ4.Weight | 0.728459 | 0.043393 | 0.990385 |
| Clostridiales_Incertae.Sedis.XI | EDEQ4.Shape | 0.729448 | -0.0232 | 0.990385 |
| Victivallaceae | EDEQ4.Shape | 0.732481 | -0.04344 | 0.990385 |
| Rikenellaceae | Mini.IPIP.Agreeableness | 0.733754 | 0.040339 | 0.990385 |
| Ruminococcaceae | EDEQ4.Weight | 0.734075 | 0.037673 | 0.990385 |
| Pasteurellaceae | EDEQ4.Shape | 0.735093 | -0.03364 | 0.990385 |
| Lachnospiraceae | Mini.IPIP.Agreeableness | 0.73933 | 0.030918 | 0.990385 |
| Peptostreptococcaceae | EDEQ4.Restraint | 0.740771 | 0.034445 | 0.990385 |
| Bacteroidaceae | EDEQ4.Total | 0.74132 | 0.048006 | 0.990385 |
| Eubacteriaceae | EDEQ4.Restraint | 0.743678 | -0.03497 | 0.990385 |
| Prevotellaceae | BMI | 0.744043 | 0.036042 | 0.990385 |
| Coriobacteriaceae | Qualtrics.age | 0.746386 | 0.037715 | 0.990385 |
| Porphyromonadaceae | Mini.IPIP.Conscientiousness | 0.748191 | 0.031559 | 0.990385 |
| Oxalobacteraceae | BDI.II.score | 0.748602 | 0.035908 | 0.990385 |
| Fusobacteriaceae | Mini.IPIP.Conscientiousness | 0.749052 | -0.03937 | 0.990385 |
| Corynebacteriaceae | BDI.II.score | 0.750008 | -0.04086 | 0.990385 |
| Bifidobacteriaceae | Mini.IPIP.Extraversion | 0.751737 | 0.027171 | 0.990385 |
| Streptococcaceae | EDEQ4.Restraint | 0.752424 | 0.040079 | 0.990385 |
| Methanobacteriaceae | Mini.IPIP.Neuroticism | 0.752736 | -0.03752 | 0.990385 |
| Aerococcaceae | Qualtrics.age | 0.75323 | -0.03974 | 0.990385 |
| Campylobacteraceae | Weight.lbs | 0.754589 | 0.040967 | 0.990385 |
| Oxalobacteraceae | EDEQ4.Weight | 0.755582 | -0.03278 | 0.990385 |
| Oxalobacteraceae | EDEQ4.Shape | 0.755994 | 0.034535 | 0.990385 |
| Coriobacteriaceae | PSS.10.score | 0.757618 | 0.030117 | 0.990385 |
| Campylobacteraceae | Mini.IPIP.Agreeableness | 0.759899 | -0.02964 | 0.990385 |
| Carnobacteriaceae | Mini.IPIP.Extraversion | 0.759986 | -0.03383 | 0.990385 |
| Clostridiaceae.2 | EDEQ4.Restraint | 0.761178 | 0.031746 | 0.990385 |
| Corynebacteriaceae | EDEQ4.Weight | 0.762037 | 0.03089 | 0.990385 |
| Eubacteriaceae | EDEQ4.Weight | 0.765209 | -0.02489 | 0.990385 |
| Methanobacteriaceae | Mini.IPIP.Agreeableness | 0.766326 | 0.028471 | 0.990385 |
| Incertae.Sedis.XI | BMI | 0.767841 | 0.017119 | 0.990385 |
| Porphyromonadaceae | EDEQ4.Total | 0.769195 | 0.03528 | 0.990385 |
| Clostridiaceae.1 | BMI | 0.771956 | -0.0235 | 0.990385 |
| Erysipelotrichaceae | EDEQ4.Restraint | 0.775897 | -0.03179 | 0.990385 |
| Corynebacteriaceae | Qualtrics.age | 0.776998 | -0.04192 | 0.990385 |
| Acidaminococcaceae | Height.inches | 0.7815 | -0.02545 | 0.990385 |
| Chitinophagaceae | EDEQ4.Eating | 0.781839 | 0.033368 | 0.990385 |
| Rhodospirillaceae | EDEQ4.Weight | 0.785792 | 0.026576 | 0.990385 |
| Victivallaceae | EDEQ4.Eating | 0.787167 | 0.029723 | 0.990385 |
| Ruminococcaceae | EDEQ4.Eating | 0.788198 | -0.03367 | 0.990385 |
| Enterobacteriaceae | Mini.IPIP.Extraversion | 0.791113 | 0.03203 | 0.990385 |
| Rikenellaceae | PSS.10.score | 0.791377 | -0.03727 | 0.990385 |
| Clostridiales_Incertae.Sedis.XI | Mini.IPIP.Conscientiousness | 0.793211 | 0.025954 | 0.990385 |
| Lactobacillaceae | Mini.IPIP.Conscientiousness | 0.793295 | 0.033283 | 0.990385 |
| Clostridiales_Incertae.Sedis.XIII | BDI.II.score | 0.795804 | -0.03726 | 0.990385 |
| Clostridiaceae.1 | Mini.IPIP.Extraversion | 0.796798 | 0.025663 | 0.990385 |
| Pyrodictiaceae | BDI.II.score | 0.797468 | 0.027473 | 0.990385 |
| Methanobacteriaceae | PSS.10.score | 0.799322 | 0.027057 | 0.990385 |
| Veillonellaceae | EDEQ4.Restraint | 0.799577 | -0.02785 | 0.990385 |
| Rhodospirillaceae | Mini.IPIP.Extraversion | 0.801507 | 0.031755 | 0.990385 |
| shannonDiversity (family) | Height.inches | 0.804305 | 0.021944 | 0.990385 |
| Verrucomicrobiaceae | BDI.II.score | 0.804375 | 0.047025 | 0.990385 |
| Verrucomicrobiaceae | BMI | 0.805841 | 0.025976 | 0.990385 |
| Gracilibacteraceae | EDEQ4.Shape | 0.805941 | 0.018441 | 0.990385 |
| Bacteroidaceae | Mini.IPIP.Imagination | 0.807656 | -0.03124 | 0.990385 |
| Bifidobacteriaceae | BAI.score | 0.807662 | -0.01907 | 0.990385 |
| Lactobacillaceae | Qualtrics.age | 0.808314 | 0.027355 | 0.990385 |
| Pyrodictiaceae | Mini.IPIP.Neuroticism | 0.811036 | -0.02653 | 0.990385 |
| Chitinophagaceae | Qualtrics.age | 0.811174 | 0.017922 | 0.990385 |
| Clostridiaceae.1 | Mini.IPIP.Agreeableness | 0.812955 | -0.02252 | 0.990385 |
| Corynebacteriaceae | Mini.IPIP.Conscientiousness | 0.813649 | -0.02609 | 0.990385 |
| Rhodospirillaceae | Mini.IPIP.Conscientiousness | 0.816938 | -0.02231 | 0.990385 |
| Defluviitaleaceae | Weight.lbs | 0.817785 | 0.018336 | 0.990385 |
| Erysipelotrichaceae | Mini.IPIP.Agreeableness | 0.818697 | -0.02702 | 0.990385 |
| Clostridiaceae.1 | EDEQ4.Weight | 0.819866 | 0.020922 | 0.990385 |
| Carnobacteriaceae | Mini.IPIP.Neuroticism | 0.822006 | -0.0231 | 0.990385 |
| Clostridiales_Incertae.Sedis.XIII | EDEQ4.Shape | 0.822519 | -0.01675 | 0.990385 |
| Pasteurellaceae | Weight.lbs | 0.822568 | 0.028216 | 0.990385 |
| Chitinophagaceae | Weight.lbs | 0.822756 | -0.03642 | 0.990385 |
| Ruminococcaceae | Mini.IPIP.Agreeableness | 0.824448 | 0.017869 | 0.990385 |
| Peptostreptococcaceae | Mini.IPIP.Imagination | 0.824844 | 0.021368 | 0.990385 |
| Rhodospirillaceae | Weight.lbs | 0.827225 | 0.023005 | 0.990385 |
| Desulfovibrionaceae | Qualtrics.age | 0.828309 | -0.02638 | 0.990385 |
| Incertae.Sedis.XI | Height.inches | 0.83039 | 0.028661 | 0.990385 |
| Chitinophagaceae | BAI.score | 0.8309 | -0.03377 | 0.990385 |
| Lactobacillaceae | Weight.lbs | 0.83116 | -0.01438 | 0.990385 |
| Actinomycetaceae | PSS.10.score | 0.831258 | 0.028529 | 0.990385 |
| Porphyromonadaceae | PSS.10.score | 0.831258 | -0.01747 | 0.990385 |
| Veillonellaceae | Height.inches | 0.833046 | -0.01212 | 0.990385 |
| Clostridiaceae.1 | BDI.II.score | 0.833106 | 0.021176 | 0.990385 |
| Micrococcaceae | Qualtrics.age | 0.833974 | -0.02195 | 0.990385 |
| Sutterellaceae | PSS.10.score | 0.836994 | 0.022549 | 0.990385 |
| Clostridiales_Incertae.Sedis.XIII | Mini.IPIP.Imagination | 0.839231 | -0.02903 | 0.990385 |
| Carnobacteriaceae | Mini.IPIP.Conscientiousness | 0.841968 | -0.02634 | 0.990385 |
| Enterococcaceae | Mini.IPIP.Conscientiousness | 0.842883 | 0.013327 | 0.990385 |
| Corynebacteriaceae | Mini.IPIP.Neuroticism | 0.842982 | -0.03088 | 0.990385 |
| Desulfovibrionaceae | BDI.II.score | 0.844635 | -0.02342 | 0.990385 |
| Bacillales_Incertae.Sedis.XI | Mini.IPIP.Extraversion | 0.846437 | -0.02007 | 0.990385 |
| Rhodospirillaceae | EDEQ4.Total | 0.84682 | 0.013272 | 0.990385 |
| Carnobacteriaceae | EDEQ4.Restraint | 0.847385 | -0.02119 | 0.990385 |
| Rikenellaceae | Mini.IPIP.Neuroticism | 0.84801 | 0.02311 | 0.990385 |
| Prevotellaceae | PSS.10.score | 0.848491 | 0.029843 | 0.990385 |
| Gracilibacteraceae | Weight.lbs | 0.848528 | -0.02052 | 0.990385 |
| Fusobacteriaceae | EDEQ4.Shape | 0.849563 | -0.02395 | 0.990385 |
| Chloroplast | EDEQ4.Restraint | 0.849916 | 0.024184 | 0.990385 |
| Chitinophagaceae | BDI.II.score | 0.851079 | 0.015998 | 0.990385 |
| Actinomycetaceae | EDEQ4.Eating | 0.851471 | -0.01761 | 0.990385 |
| Pyrodictiaceae | EDEQ4.Total | 0.854236 | 0.025004 | 0.990385 |
| Enterococcaceae | Qualtrics.age | 0.855624 | -0.03029 | 0.990385 |
| Acidaminococcaceae | EDEQ4.Eating | 0.857858 | 0.019154 | 0.990385 |
| Bacteroidaceae | EDEQ4.Eating | 0.857858 | -0.01959 | 0.990385 |
| Incertae.Sedis.XI | Mini.IPIP.Imagination | 0.858684 | 0.010767 | 0.990385 |
| Actinomycetaceae | EDEQ4.Restraint | 0.859542 | 0.019882 | 0.990385 |
| Bifidobacteriaceae | Mini.IPIP.Neuroticism | 0.859575 | 0.021984 | 0.990385 |
| Fusobacteriaceae | EDEQ4.Eating | 0.861799 | 0.018845 | 0.990385 |
| Fusobacteriaceae | Mini.IPIP.Neuroticism | 0.861925 | -0.01826 | 0.990385 |
| Desulfovibrionaceae | Mini.IPIP.Neuroticism | 0.862446 | 0.018082 | 0.990385 |
| Campylobacteraceae | EDEQ4.Weight | 0.863512 | 0.019932 | 0.990385 |
| Bacillales_Incertae.Sedis.XI | PSS.10.score | 0.867062 | 0.018368 | 0.99099 |
| Porphyromonadaceae | BDI.II.score | 0.867875 | -0.01847 | 0.99099 |
| Pyrodictiaceae | EDEQ4.Restraint | 0.867926 | -0.02117 | 0.99099 |
| Acidaminococcaceae | Mini.IPIP.Agreeableness | 0.876601 | -0.01689 | 0.994086 |
| Rikenellaceae | Mini.IPIP.Extraversion | 0.877218 | -0.01212 | 0.994086 |
| Gracilibacteraceae | BMI | 0.877336 | -0.01809 | 0.994086 |
| Clostridiaceae.1 | EDEQ4.Restraint | 0.877708 | -0.01105 | 0.994086 |
| Ruminococcaceae | BMI | 0.88044 | 0.000739 | 0.994086 |
| Incertae.Sedis.XI | Qualtrics.age | 0.880907 | -0.01899 | 0.994086 |
| Campylobacteraceae | EDEQ4.Shape | 0.8826 | -0.01995 | 0.994086 |
| shannonDiversity (family) | Mini.IPIP.Extraversion | 0.883022 | -0.02807 | 0.994086 |
| Bacteroidaceae | Qualtrics.age | 0.883129 | 0.019424 | 0.994086 |
| Leuconostocaceae | EDEQ4.Restraint | 0.885998 | 0.017426 | 0.994086 |
| Enterobacteriaceae | BMI | 0.886225 | -0.0154 | 0.994086 |
| Rikenellaceae | BMI | 0.886231 | 0.013139 | 0.994086 |
| Chloroplast | BDI.II.score | 0.890778 | 0.007494 | 0.996559 |
| Ruminococcaceae | BAI.score | 0.894362 | 0.015751 | 0.996559 |
| Incertae.Sedis.XI | EDEQ4.Total | 0.896673 | 0.016482 | 0.996559 |
| Clostridiales_Incertae.Sedis.XI | BAI.score | 0.897218 | 0.006353 | 0.996559 |
| Clostridiaceae.2 | BAI.score | 0.897269 | -0.01386 | 0.996559 |
| Bacillales_Incertae.Sedis.XI | EDEQ4.Weight | 0.897757 | -0.01389 | 0.996559 |
| Actinomycetaceae | Mini.IPIP.Agreeableness | 0.899968 | -0.01555 | 0.996559 |
| Bifidobacteriaceae | Mini.IPIP.Agreeableness | 0.899968 | -0.017 | 0.996559 |
| Clostridiales_Incertae.Sedis.XIII | Mini.IPIP.Conscientiousness | 0.90016 | -0.01597 | 0.996559 |
| Victivallaceae | EDEQ4.Weight | 0.903185 | 0.010683 | 0.998054 |
| Clostridiaceae.2 | EDEQ4.Weight | 0.904902 | -0.01377 | 0.998054 |
| Clostridiales_Incertae.Sedis.XI | Mini.IPIP.Extraversion | 0.90624 | -0.00412 | 0.998054 |
| Rikenellaceae | EDEQ4.Eating | 0.909259 | -0.01398 | 0.998054 |
| Bacteroidaceae | Weight.lbs | 0.909287 | 0.003544 | 0.998054 |
| Veillonellaceae | Mini.IPIP.Imagination | 0.911886 | 0.02233 | 0.998054 |
| Incertae.Sedis.XI | EDEQ4.Weight | 0.913916 | -0.01853 | 0.998054 |
| Corynebacteriaceae | BMI | 0.914084 | 0.00579 | 0.998054 |
| Victivallaceae | BDI.II.score | 0.914713 | -0.01259 | 0.998054 |
| Methanobacteriaceae | Mini.IPIP.Imagination | 0.919281 | -0.00452 | 0.998054 |
| Pasteurellaceae | BAI.score | 0.920655 | -0.00552 | 0.998054 |
| Bifidobacteriaceae | BMI | 0.921097 | -0.00982 | 0.998054 |
| Verrucomicrobiaceae | EDEQ4.Eating | 0.922177 | -0.00957 | 0.998054 |
| Lactobacillaceae | Mini.IPIP.Extraversion | 0.923792 | -0.0018 | 0.998054 |
| Rikenellaceae | EDEQ4.Shape | 0.923834 | 0.010178 | 0.998054 |
| Bifidobacteriaceae | Qualtrics.age | 0.923876 | -0.00205 | 0.998054 |
| Oxalobacteraceae | Mini.IPIP.Agreeableness | 0.92566 | 0.005058 | 0.998054 |
| Clostridiaceae.1 | EDEQ4.Eating | 0.928644 | -0.01056 | 0.998054 |
| Coriobacteriaceae | Mini.IPIP.Imagination | 0.929457 | 0.015348 | 0.998054 |
| Defluviitaleaceae | EDEQ4.Eating | 0.931149 | 0.010307 | 0.998054 |
| Eubacteriaceae | EDEQ4.Eating | 0.931879 | 0.003549 | 0.998054 |
| Porphyromonadaceae | EDEQ4.Eating | 0.935116 | -0.00879 | 0.998054 |
| Peptostreptococcaceae | EDEQ4.Total | 0.935686 | 0.003692 | 0.998054 |
| Coriobacteriaceae | Mini.IPIP.Neuroticism | 0.941237 | -0.00679 | 0.998054 |
| Enterococcaceae | Mini.IPIP.Extraversion | 0.941314 | -0.00654 | 0.998054 |
| Streptococcaceae | PSS.10.score | 0.941425 | -0.01254 | 0.998054 |
| Chitinophagaceae | Mini.IPIP.Imagination | 0.943251 | -0.0067 | 0.998054 |
| Bacillales_Incertae.Sedis.XI | Mini.IPIP.Agreeableness | 0.943305 | 0.010743 | 0.998054 |
| Erysipelotrichaceae | Qualtrics.age | 0.947257 | 0.006118 | 0.998054 |
| Bacteroidaceae | Height.inches | 0.949871 | -0.01348 | 0.998054 |
| Bacteroidaceae | Mini.IPIP.Conscientiousness | 0.949982 | 0.005206 | 0.998054 |
| Bifidobacteriaceae | EDEQ4.Shape | 0.953084 | -0.02736 | 0.998054 |
| Erysipelotrichaceae | EDEQ4.Shape | 0.953084 | 0.008424 | 0.998054 |
| Clostridiaceae.2 | Qualtrics.age | 0.953105 | -0.01226 | 0.998054 |
| Bifidobacteriaceae | EDEQ4.Eating | 0.954556 | 0.006351 | 0.998054 |
| Streptococcaceae | BDI.II.score | 0.955778 | -0.01039 | 0.998054 |
| Prevotellaceae | EDEQ4.Restraint | 0.95705 | -0.00666 | 0.998054 |
| Aerococcaceae | BAI.score | 0.958554 | -0.00825 | 0.998054 |
| Desulfovibrionaceae | Mini.IPIP.Agreeableness | 0.958713 | 0.000996 | 0.998054 |
| Pasteurellaceae | BMI | 0.959031 | 0.007072 | 0.998054 |
| Enterobacteriaceae | EDEQ4.Restraint | 0.960113 | 6.21E-05 | 0.998054 |
| Rhodospirillaceae | EDEQ4.Eating | 0.963657 | 0.001717 | 0.998054 |
| Veillonellaceae | Mini.IPIP.Agreeableness | 0.964613 | -0.00583 | 0.998054 |
| Rikenellaceae | BAI.score | 0.964695 | 0.006084 | 0.998054 |
| Sutterellaceae | EDEQ4.Shape | 0.964804 | 0.005537 | 0.998054 |
| Bacteroidaceae | PSS.10.score | 0.964835 | -0.00896 | 0.998054 |
| Pyrodictiaceae | Weight.lbs | 0.969966 | 0.00707 | 0.998054 |
| Sutterellaceae | BAI.score | 0.970576 | -0.00377 | 0.998054 |
| Streptococcaceae | BAI.score | 0.970577 | 0.013946 | 0.998054 |
| Bifidobacteriaceae | EDEQ4.Total | 0.970741 | -0.00768 | 0.998054 |
| Gracilibacteraceae | Height.inches | 0.970808 | -0.00565 | 0.998054 |
| Chitinophagaceae | PSS.10.score | 0.973207 | 0.002669 | 0.998054 |
| Eubacteriaceae | EDEQ4.Total | 0.973666 | 0.004134 | 0.998054 |
| Coriobacteriaceae | Mini.IPIP.Agreeableness | 0.976404 | -0.00116 | 0.998054 |
| Actinomycetaceae | EDEQ4.Total | 0.976591 | 0.002753 | 0.998054 |
| Sutterellaceae | EDEQ4.Total | 0.976591 | 0.002507 | 0.998054 |
| Oxalobacteraceae | Mini.IPIP.Conscientiousness | 0.977387 | 0.003617 | 0.998054 |
| Chloroplast | EDEQ4.Shape | 0.979384 | 0.013031 | 0.998054 |
| Actinomycetaceae | Mini.IPIP.Conscientiousness | 0.979393 | 0.008497 | 0.998054 |
| Clostridiales_Incertae.Sedis.XI | EDEQ4.Weight | 0.98212 | -0.00559 | 0.998054 |
| Enterobacteriaceae | Mini.IPIP.Agreeableness | 0.982301 | 0.011098 | 0.998054 |
| Bacteroidaceae | EDEQ4.Shape | 0.982398 | -0.00548 | 0.998054 |
| Fusobacteriaceae | Qualtrics.age | 0.98391 | -0.00105 | 0.998264 |
| Micrococcaceae | Weight.lbs | 0.985329 | 0.003427 | 0.99838 |
| Corynebacteriaceae | Mini.IPIP.Imagination | 0.988063 | -0.0005 | 0.999826 |
| Defluviitaleaceae | Mini.IPIP.Agreeableness | 0.991053 | -0.00383 | 1 |
| Rhodospirillaceae | PSS.10.score | 0.993431 | -0.00092 | 1 |
| Bacillales_Incertae.Sedis.XI | Mini.IPIP.Conscientiousness | 0.997019 | -0.0019 | 1 |
| Coriobacteriaceae | EDEQ4.Weight | 0.997021 | 0.001789 | 1 |
| Enterobacteriaceae | Mini.IPIP.Conscientiousness | 0.997056 | 0.002337 | 1 |
| Actinomycetaceae | Mini.IPIP.Neuroticism | 1 | 0.005549 | 1 |
| Campylobacteraceae | EDEQ4.Total | 1 | 0.003258 | 1 |
| Erysipelotrichaceae | EDEQ4.Total | 1 | 0.003865 | 1 |
| Rhodospirillaceae | Mini.IPIP.Imagination | 1 | -0.00457 | 1 |
| Butyricimonas | EDEQ4.Restraint | 0.000235 | 0.408541 | 0.511617 |
| Pseudobutyrivibrio | Height.inches | 0.000449 | 0.371772 | 0.511617 |
| Acetanaerobacterium | Mini.IPIP.Extraversion | 0.000743 | -0.35603 | 0.564082 |
| Erysipelotrichaceae_incertae_sedis | Mini.IPIP.Extraversion | 0.001271 | -0.34685 | 0.579659 |
| Lactovum | Weight.lbs | 0.001272 | -0.35045 | 0.579659 |
| Granulicatella | Height.inches | 0.002275 | -0.32685 | 0.72441 |
| Acetivibrio | Height.inches | 0.00251 | 0.316562 | 0.72441 |
| Erysipelotrichaceae_incertae_sedis | BMI | 0.003071 | -0.3211 | 0.72441 |
| Megasphaera | EDEQ4.Eating | 0.003131 | 0.329561 | 0.72441 |
| Butyricimonas | Qualtrics.age | 0.00318 | 0.315794 | 0.72441 |
| Lactococcus | BMI | 0.003841 | -0.30628 | 0.725671 |
| Enterobacter | Mini.IPIP.Conscientiousness | 0.004529 | -0.30452 | 0.725671 |
| Prevotella | EDEQ4.Eating | 0.00461 | 0.298493 | 0.725671 |
| Murdochiella | EDEQ4.Eating | 0.004991 | -0.30107 | 0.725671 |
| Parasporobacterium | EDEQ4.Total | 0.005332 | 0.289997 | 0.725671 |
| Saccharibacteria_genera_incertae_sedis | BDI.II.score | 0.005359 | 0.304958 | 0.725671 |
| Butyricimonas | EDEQ4.Total | 0.005978 | 0.288975 | 0.725671 |
| Anaerovorax | Qualtrics.age | 0.006418 | 0.294091 | 0.725671 |
| Clostridium.sensu.stricto | Height.inches | 0.007002 | 0.30185 | 0.725671 |
| Slackia | BDI.II.score | 0.00728 | 0.285567 | 0.725671 |
| Lactonifactor | PSS.10.score | 0.007481 | -0.2797 | 0.725671 |
| Lactovum | BMI | 0.007545 | -0.27904 | 0.725671 |
| Akkermansia | Qualtrics.age | 0.007796 | 0.276826 | 0.725671 |
| Acetanaerobacterium | BMI | 0.008078 | -0.29262 | 0.725671 |
| Lactococcus | Weight.lbs | 0.008136 | -0.27442 | 0.725671 |
| Peptostreptococcus | EDEQ4.Eating | 0.008696 | -0.28798 | 0.725671 |
| Butyricicoccus | EDEQ4.Eating | 0.009185 | -0.28004 | 0.725671 |
| Actinobacillus | PSS.10.score | 0.010093 | 0.274161 | 0.725671 |
| Gallibacterium | Mini.IPIP.Conscientiousness | 0.010395 | -0.27513 | 0.725671 |
| Sarcina | Height.inches | 0.010805 | 0.264073 | 0.725671 |
| Actinobacillus | BDI.II.score | 0.010846 | 0.280116 | 0.725671 |
| Granulicatella | Weight.lbs | 0.010894 | -0.27249 | 0.725671 |
| Enterorhabdus | Mini.IPIP.Extraversion | 0.011102 | -0.2658 | 0.725671 |
| Parasporobacterium | Height.inches | 0.011473 | 0.258005 | 0.725671 |
| Syntrophococcus | Mini.IPIP.Imagination | 0.011955 | -0.26622 | 0.725671 |
| Parabacteroides | Qualtrics.age | 0.012452 | 0.287209 | 0.725671 |
| Hallella | Height.inches | 0.012724 | -0.27045 | 0.725671 |
| Corynebacterium | Mini.IPIP.Agreeableness | 0.0132 | 0.268421 | 0.725671 |
| Finegoldia | Height.inches | 0.013235 | -0.26598 | 0.725671 |
| Slackia | Weight.lbs | 0.013585 | 0.271275 | 0.725671 |
| Gallibacterium | PSS.10.score | 0.013868 | 0.264132 | 0.725671 |
| Hallella | EDEQ4.Eating | 0.014431 | 0.262147 | 0.725671 |
| Sporobacter | Height.inches | 0.014805 | 0.263708 | 0.725671 |
| Veillonella | Qualtrics.age | 0.015128 | -0.2661 | 0.725671 |
| Sarcina | Mini.IPIP.Neuroticism | 0.015869 | 0.26161 | 0.725671 |
| Varibaculum | PSS.10.score | 0.01632 | 0.255942 | 0.725671 |
| Paraprevotella | BDI.II.score | 0.016425 | 0.254286 | 0.725671 |
| Defluviitalea | Mini.IPIP.Conscientiousness | 0.016621 | 0.257982 | 0.725671 |
| Anaerofilum | EDEQ4.Total | 0.016776 | 0.255728 | 0.725671 |
| Barnesiella | Qualtrics.age | 0.016891 | 0.237909 | 0.725671 |
| Clostridium.XlVa | Mini.IPIP.Neuroticism | 0.016924 | -0.24948 | 0.725671 |
| Gemella | Height.inches | 0.01743 | -0.26748 | 0.725671 |
| Pseudoflavonifractor | Mini.IPIP.Neuroticism | 0.017439 | -0.24204 | 0.725671 |
| Saccharibacteria_genera_incertae_sedis | Mini.IPIP.Neuroticism | 0.017454 | 0.261326 | 0.725671 |
| Rothia | EDEQ4.Shape | 0.017897 | -0.26401 | 0.725671 |
| Sporobacter | Qualtrics.age | 0.017945 | 0.258589 | 0.725671 |
| Erysipelotrichaceae_incertae_sedis | Weight.lbs | 0.018475 | -0.26896 | 0.725671 |
| Eggerthella | Weight.lbs | 0.01903 | -0.25002 | 0.725671 |
| Peptostreptococcus | EDEQ4.Total | 0.019165 | -0.25382 | 0.725671 |
| Parvimonas | EDEQ4.Restraint | 0.019206 | -0.25111 | 0.725671 |
| Clostridium.sensu.stricto | Weight.lbs | 0.019953 | 0.261353 | 0.725671 |
| Gallibacterium | Mini.IPIP.Agreeableness | 0.019964 | -0.24767 | 0.725671 |
| Turicibacter | Mini.IPIP.Extraversion | 0.020069 | -0.25176 | 0.725671 |
| Turicibacter | Mini.IPIP.Agreeableness | 0.020836 | -0.25264 | 0.734431 |
| Acetivibrio | Mini.IPIP.Conscientiousness | 0.021464 | -0.2443 | 0.734431 |
| Parasporobacterium | Weight.lbs | 0.022236 | 0.240406 | 0.734431 |
| shannonDiversity (genus) | Qualtrics.age | 0.022694 | 0.242279 | 0.734431 |
| Lactonifactor | Mini.IPIP.Conscientiousness | 0.022804 | 0.261112 | 0.734431 |
| Phascolarctobacterium | Mini.IPIP.Neuroticism | 0.023182 | -0.24434 | 0.734431 |
| Veillonella | Mini.IPIP.Neuroticism | 0.023858 | 0.234574 | 0.734431 |
| Syntrophococcus | Weight.lbs | 0.024374 | -0.23708 | 0.734431 |
| Parasporobacterium | EDEQ4.Restraint | 0.025198 | 0.236528 | 0.734431 |
| Subdoligranulum | Mini.IPIP.Extraversion | 0.025318 | -0.2339 | 0.734431 |
| Sarcina | Mini.IPIP.Agreeableness | 0.025505 | -0.22621 | 0.734431 |
| Anaerofilum | Qualtrics.age | 0.025612 | 0.255107 | 0.734431 |
| Sporacetigenium | Height.inches | 0.025913 | 0.255047 | 0.734431 |
| Sporacetigenium | Weight.lbs | 0.026586 | 0.256421 | 0.734431 |
| Acidaminococcus | EDEQ4.Total | 0.026616 | 0.238692 | 0.734431 |
| Eubacterium | BMI | 0.026947 | -0.221 | 0.734431 |
| Roseburia | PSS.10.score | 0.026987 | 0.233351 | 0.734431 |
| Anaerofustis | BMI | 0.027837 | -0.23593 | 0.734431 |
| Pseudobutyrivibrio | BMI | 0.027878 | -0.24081 | 0.734431 |
| Haemophilus | Qualtrics.age | 0.028103 | -0.22883 | 0.734431 |
| Anaerosporobacter | Mini.IPIP.Neuroticism | 0.028303 | -0.23407 | 0.734431 |
| Lachnoanaerobaculum | Qualtrics.age | 0.028699 | 0.239951 | 0.734431 |
| Eubacterium | Mini.IPIP.Neuroticism | 0.028833 | -0.23014 | 0.734431 |
| Anaerotruncus | Mini.IPIP.Extraversion | 0.029128 | -0.22756 | 0.734431 |
| Acidaminococcus | EDEQ4.Restraint | 0.029172 | 0.235676 | 0.734431 |
| Faecalibacterium | Weight.lbs | 0.029292 | 0.237486 | 0.734431 |
| Anaerofilum | EDEQ4.Weight | 0.029431 | 0.233595 | 0.734431 |
| Prevotella | EDEQ4.Total | 0.029624 | 0.223973 | 0.734431 |
| Prevotella | Height.inches | 0.029661 | -0.22989 | 0.734431 |
| Paralactobacillus | Mini.IPIP.Agreeableness | 0.03064 | -0.23241 | 0.750521 |
| Adlercreutzia | Mini.IPIP.Extraversion | 0.031166 | -0.22527 | 0.755274 |
| Enterorhabdus | Mini.IPIP.Agreeableness | 0.031662 | -0.22747 | 0.75922 |
| Prevotella | EDEQ4.Shape | 0.032048 | 0.2327 | 0.760466 |
| Acetanaerobacterium | Height.inches | 0.03285 | 0.223287 | 0.768099 |
| Faecalibacterium | Qualtrics.age | 0.033044 | -0.21563 | 0.768099 |
| Enterorhabdus | EDEQ4.Eating | 0.033543 | 0.234516 | 0.771819 |
| Oscillibacter | Height.inches | 0.034085 | 0.22985 | 0.774794 |
| Lactovum | Mini.IPIP.Agreeableness | 0.034523 | 0.216427 | 0.774794 |
| Desulfovibrio | EDEQ4.Restraint | 0.034767 | 0.232357 | 0.774794 |
| Pseudoflavonifractor | Height.inches | 0.035032 | 0.240603 | 0.774794 |
| Eggerthia | BMI | 0.036213 | -0.22829 | 0.781361 |
| Oscillibacter | Mini.IPIP.Neuroticism | 0.03764 | 0.225533 | 0.781361 |
| Veillonella | BDI.II.score | 0.037739 | 0.22087 | 0.781361 |
| Hallella | EDEQ4.Total | 0.037835 | 0.2262 | 0.781361 |
| Megamonas | Mini.IPIP.Imagination | 0.037907 | 0.216288 | 0.781361 |
| Gallibacterium | Mini.IPIP.Imagination | 0.038523 | -0.22575 | 0.781361 |
| Lactovum | EDEQ4.Eating | 0.039129 | 0.220321 | 0.781361 |
| Tannerella | Mini.IPIP.Agreeableness | 0.039263 | -0.22225 | 0.781361 |
| Weissella | Mini.IPIP.Agreeableness | 0.039263 | -0.22659 | 0.781361 |
| Olsenella | Mini.IPIP.Imagination | 0.039457 | -0.2272 | 0.781361 |
| Lachnoanaerobaculum | EDEQ4.Weight | 0.039486 | 0.212577 | 0.781361 |
| Methanobrevibacter | EDEQ4.Total | 0.040054 | 0.220223 | 0.781361 |
| Mogibacterium | Mini.IPIP.Extraversion | 0.040075 | -0.22149 | 0.781361 |
| Paralactobacillus | EDEQ4.Shape | 0.040688 | 0.214219 | 0.781361 |
| Lachnobacterium | Qualtrics.age | 0.040831 | 0.219215 | 0.781361 |
| Saccharibacteria_genera_incertae_sedis | PSS.10.score | 0.041377 | 0.219008 | 0.781361 |
| Pyrolobus | Mini.IPIP.Imagination | 0.042137 | 0.22356 | 0.781361 |
| Hespellia | Qualtrics.age | 0.042409 | 0.19955 | 0.781361 |
| Barnesiella | Weight.lbs | 0.042828 | 0.209713 | 0.781361 |
| Anaerostipes | Qualtrics.age | 0.043249 | -0.22017 | 0.781361 |
| Hespellia | EDEQ4.Restraint | 0.043369 | 0.211962 | 0.781361 |
| Gardnerella | Mini.IPIP.Conscientiousness | 0.043489 | 0.221581 | 0.781361 |
| Parasporobacterium | Mini.IPIP.Conscientiousness | 0.043523 | -0.21333 | 0.781361 |
| Anaerostipes | EDEQ4.Shape | 0.043898 | -0.2088 | 0.781361 |
| Hydrogenoanaerobacterium | Height.inches | 0.044462 | 0.225055 | 0.781361 |
| Defluviitalea | PSS.10.score | 0.044873 | -0.20649 | 0.781361 |
| Hydrogenoanaerobacterium | Qualtrics.age | 0.046607 | 0.209946 | 0.781361 |
| Desulfovibrio | EDEQ4.Eating | 0.048061 | 0.214584 | 0.781361 |
| Asaccharobacter | Mini.IPIP.Extraversion | 0.048133 | -0.21513 | 0.781361 |
| Veillonella | PSS.10.score | 0.048501 | 0.217498 | 0.781361 |
| Lachnobacterium | Height.inches | 0.048524 | 0.213955 | 0.781361 |
| Alistipes | Height.inches | 0.048708 | 0.222318 | 0.781361 |
| Porphyromonas | BDI.II.score | 0.048767 | -0.20752 | 0.781361 |
| Anaerofilum | EDEQ4.Restraint | 0.049029 | 0.208273 | 0.781361 |
| Anaerosporobacter | BMI | 0.049252 | -0.2232 | 0.781361 |
| Enterorhabdus | EDEQ4.Weight | 0.04978 | 0.212499 | 0.781361 |
| Parasporobacterium | Qualtrics.age | 0.049803 | 0.223811 | 0.781361 |
| Enterobacter | EDEQ4.Eating | 0.050248 | 0.213534 | 0.781361 |
| Butyricimonas | EDEQ4.Weight | 0.051473 | 0.205947 | 0.781361 |
| Ethanoligenens | Mini.IPIP.Neuroticism | 0.051621 | -0.20643 | 0.781361 |
| Saccharibacteria_genera_incertae_sedis | BMI | 0.051653 | 0.198625 | 0.781361 |
| Anaerofilum | Mini.IPIP.Extraversion | 0.05192 | -0.21992 | 0.781361 |
| Marvinbryantia | Height.inches | 0.052186 | 0.203794 | 0.781361 |
| Collinsella | BMI | 0.052297 | 0.19221 | 0.781361 |
| Victivallis | Mini.IPIP.Extraversion | 0.052302 | 0.207343 | 0.781361 |
| Anaerovorax | Height.inches | 0.052699 | 0.200797 | 0.781361 |
| Rothia | EDEQ4.Total | 0.054268 | -0.21024 | 0.781361 |
| Acidaminococcus | Mini.IPIP.Imagination | 0.054365 | -0.20749 | 0.781361 |
| Akkermansia | Height.inches | 0.054938 | 0.214216 | 0.781361 |
| Ethanoligenens | PSS.10.score | 0.055111 | -0.21716 | 0.781361 |
| Sporobacter | EDEQ4.Total | 0.055542 | 0.218827 | 0.781361 |
| Slackia | BMI | 0.055728 | 0.208529 | 0.781361 |
| Faecalibacterium | Height.inches | 0.05588 | 0.203919 | 0.781361 |
| Coprobacillus | EDEQ4.Eating | 0.057112 | 0.204501 | 0.781361 |
| Defluviitalea | BAI.score | 0.057191 | -0.20264 | 0.781361 |
| Abiotrophia | BDI.II.score | 0.057257 | 0.200655 | 0.781361 |
| Gemella | EDEQ4.Restraint | 0.057381 | -0.21316 | 0.781361 |
| Holdemania | Height.inches | 0.057799 | -0.20312 | 0.781361 |
| Blautia | EDEQ4.Eating | 0.057848 | -0.20506 | 0.781361 |
| Holdemania | Weight.lbs | 0.057885 | -0.1847 | 0.781361 |
| Parasporobacterium | EDEQ4.Weight | 0.057897 | 0.202608 | 0.781361 |
| Gemmiger | Qualtrics.age | 0.057914 | 0.207816 | 0.781361 |
| Clostridium.XlVb | BAI.score | 0.057973 | 0.226297 | 0.781361 |
| Hallella | Mini.IPIP.Extraversion | 0.058481 | 0.204447 | 0.781361 |
| Anaerovorax | EDEQ4.Total | 0.058578 | 0.211797 | 0.781361 |
| Anaerofilum | EDEQ4.Shape | 0.059316 | 0.199567 | 0.781361 |
| Eggerthia | PSS.10.score | 0.05941 | -0.20405 | 0.781361 |
| Abiotrophia | Height.inches | 0.05965 | -0.20433 | 0.781361 |
| Natronincola | Weight.lbs | 0.059663 | 0.212411 | 0.781361 |
| Peptostreptococcus | EDEQ4.Restraint | 0.059878 | -0.20864 | 0.781361 |
| Lactovum | Height.inches | 0.060014 | -0.20477 | 0.781361 |
| Peptostreptococcus | Weight.lbs | 0.060116 | -0.21441 | 0.781361 |
| Atopobium | EDEQ4.Shape | 0.060369 | -0.18452 | 0.781361 |
| Blautia | Weight.lbs | 0.061374 | -0.20547 | 0.781459 |
| Parvimonas | Height.inches | 0.061514 | -0.20445 | 0.781459 |
| Anaerosporobacter | Qualtrics.age | 0.061903 | 0.192386 | 0.781459 |
| Eggerthella | BMI | 0.062371 | -0.18834 | 0.781459 |
| Peptostreptococcus | EDEQ4.Weight | 0.062402 | -0.20165 | 0.781459 |
| Hallella | Weight.lbs | 0.062864 | -0.2149 | 0.781459 |
| Fusobacterium | BAI.score | 0.063057 | 0.20458 | 0.781459 |
| Victivallis | EDEQ4.Restraint | 0.063367 | 0.19625 | 0.781459 |
| Blautia | EDEQ4.Total | 0.063464 | -0.20049 | 0.781459 |
| Veillonella | Mini.IPIP.Extraversion | 0.065334 | 0.200877 | 0.792073 |
| Actinobacillus | Mini.IPIP.Conscientiousness | 0.06601 | -0.19855 | 0.792073 |
| Moryella | Mini.IPIP.Conscientiousness | 0.066384 | 0.205032 | 0.792073 |
| Leuconostoc | Weight.lbs | 0.066399 | -0.1944 | 0.792073 |
| Anaerovorax | EDEQ4.Weight | 0.066509 | 0.198301 | 0.792073 |
| Haemophilus | Mini.IPIP.Agreeableness | 0.067016 | -0.19568 | 0.792073 |
| Holdemania | Qualtrics.age | 0.067214 | -0.20065 | 0.792073 |
| Rothia | BDI.II.score | 0.06743 | 0.198135 | 0.792073 |
| Rothia | PSS.10.score | 0.06747 | 0.204848 | 0.792073 |
| Lachnoanaerobaculum | EDEQ4.Total | 0.067803 | 0.198333 | 0.792073 |
| Tannerella | EDEQ4.Restraint | 0.069286 | 0.189598 | 0.794414 |
| Oscillibacter | BMI | 0.069379 | -0.18592 | 0.794414 |
| Dorea | BMI | 0.069379 | 0.182345 | 0.794414 |
| Anaerofilum | Height.inches | 0.069398 | 0.199001 | 0.794414 |
| Hallella | EDEQ4.Weight | 0.070036 | 0.195789 | 0.795955 |
| Anaerococcus | Mini.IPIP.Extraversion | 0.070472 | 0.189519 | 0.795955 |
| Hespellia | Mini.IPIP.Agreeableness | 0.070581 | 0.186591 | 0.795955 |
| Pseudoflavonifractor | BDI.II.score | 0.07122 | -0.19385 | 0.799207 |
| Butyricimonas | EDEQ4.Eating | 0.07174 | 0.199348 | 0.801096 |
| Acetitomaculum | EDEQ4.Eating | 0.072459 | 0.197745 | 0.801322 |
| Lactococcus | Mini.IPIP.Agreeableness | 0.07295 | 0.177566 | 0.801322 |
| Coprococcus | BAI.score | 0.073033 | -0.19263 | 0.801322 |
| Clostridium.sensu.stricto | Mini.IPIP.Neuroticism | 0.073167 | 0.199929 | 0.801322 |
| Coprococcus | Qualtrics.age | 0.074084 | 0.197789 | 0.807482 |
| Parabacteroides | Mini.IPIP.Agreeableness | 0.074747 | 0.201428 | 0.809298 |
| Escherichia.Shigella | PSS.10.score | 0.074961 | 0.189235 | 0.809298 |
| Finegoldia | EDEQ4.Eating | 0.076374 | -0.19501 | 0.810489 |
| Papillibacter | BMI | 0.076375 | -0.19552 | 0.810489 |
| Eubacterium | Height.inches | 0.076472 | 0.193484 | 0.810489 |
| Acetanaerobacterium | Mini.IPIP.Conscientiousness | 0.076525 | 0.188072 | 0.810489 |
| Pseudoflavonifractor | Qualtrics.age | 0.077106 | 0.192388 | 0.810489 |
| Odoribacter | BAI.score | 0.077206 | -0.19047 | 0.810489 |
| Actinomyces | BDI.II.score | 0.077834 | 0.198303 | 0.813157 |
| Olsenella | BAI.score | 0.078426 | 0.194258 | 0.813157 |
| Weissella | EDEQ4.Eating | 0.078682 | 0.188736 | 0.813157 |
| Acetanaerobacterium | Mini.IPIP.Agreeableness | 0.079059 | -0.18074 | 0.813157 |
| Gallibacterium | BDI.II.score | 0.079245 | 0.195077 | 0.813157 |
| Saccharibacteria_genera_incertae_sedis | EDEQ4.Eating | 0.079992 | 0.187354 | 0.817139 |
| Megamonas | Mini.IPIP.Conscientiousness | 0.080891 | 0.187817 | 0.818948 |
| Syntrophococcus | Height.inches | 0.081868 | -0.1903 | 0.818948 |
| Leuconostoc | Mini.IPIP.Imagination | 0.082244 | -0.17922 | 0.818948 |
| Robinsoniella | Mini.IPIP.Neuroticism | 0.082854 | -0.19476 | 0.818948 |
| Atopobium | Mini.IPIP.Extraversion | 0.082873 | -0.18152 | 0.818948 |
| Butyricimonas | Weight.lbs | 0.083067 | 0.203251 | 0.818948 |
| Odoribacter | EDEQ4.Shape | 0.083229 | -0.19478 | 0.818948 |
| Shuttleworthia | PSS.10.score | 0.083248 | 0.182584 | 0.818948 |
| Lachnospiracea_incertae_sedis | Mini.IPIP.Conscientiousness | 0.083526 | 0.18904 | 0.818948 |
| Peptostreptococcus | EDEQ4.Shape | 0.084282 | -0.18041 | 0.818948 |
| Corynebacterium | BAI.score | 0.084822 | 0.195797 | 0.818948 |
| Anaerococcus | Height.inches | 0.084959 | -0.18835 | 0.818948 |
| Defluviitalea | Mini.IPIP.Extraversion | 0.085084 | -0.18591 | 0.818948 |
| Escherichia.Shigella | BAI.score | 0.085202 | 0.181892 | 0.818948 |
| Butyricimonas | Height.inches | 0.085821 | 0.191986 | 0.821433 |
| Clostridium.XlVa | Mini.IPIP.Conscientiousness | 0.086194 | 0.172486 | 0.821549 |
| Pyrolobus | Mini.IPIP.Agreeableness | 0.087276 | -0.18185 | 0.82708 |
| Acidaminococcus | EDEQ4.Shape | 0.087501 | 0.183376 | 0.82708 |
| Victivallis | BAI.score | 0.088969 | -0.17803 | 0.832496 |
| Anaerosporobacter | BDI.II.score | 0.08902 | -0.18293 | 0.832496 |
| Saccharibacteria_genera_incertae_sedis | Weight.lbs | 0.089996 | 0.176022 | 0.832496 |
| Parasporobacterium | EDEQ4.Shape | 0.090129 | 0.177508 | 0.832496 |
| Enterobacter | BDI.II.score | 0.090266 | 0.182182 | 0.832496 |
| Enterobacter | Mini.IPIP.Agreeableness | 0.090266 | -0.18296 | 0.832496 |
| Haemophilus | PSS.10.score | 0.091462 | 0.193036 | 0.836804 |
| Actinobacillus | Mini.IPIP.Agreeableness | 0.09148 | -0.18704 | 0.836804 |
| Robinsoniella | BMI | 0.091835 | -0.18973 | 0.836804 |
| Peptoniphilus | Height.inches | 0.093446 | -0.19183 | 0.84809 |
| Sarcina | Weight.lbs | 0.095354 | 0.186041 | 0.861972 |
| Collinsella | Height.inches | 0.096816 | -0.19006 | 0.866694 |
| Lachnobacterium | BMI | 0.097102 | -0.16748 | 0.866694 |
| Defluviitalea | Height.inches | 0.097665 | 0.173404 | 0.866694 |
| Murdochiella | Mini.IPIP.Imagination | 0.097793 | 0.177823 | 0.866694 |
| Blautia | EDEQ4.Restraint | 0.098095 | -0.18067 | 0.866694 |
| Bilophila | BDI.II.score | 0.098159 | -0.17808 | 0.866694 |
| Lactococcus | BAI.score | 0.100707 | -0.16469 | 0.874758 |
| Eggerthia | EDEQ4.Total | 0.100935 | -0.17453 | 0.874758 |
| Eggerthella | Mini.IPIP.Extraversion | 0.101644 | -0.17341 | 0.874758 |
| Defluviitalea | Qualtrics.age | 0.10207 | 0.181529 | 0.874758 |
| Marvinbryantia | Mini.IPIP.Agreeableness | 0.102224 | 0.174719 | 0.874758 |
| Campylobacter | Mini.IPIP.Extraversion | 0.102332 | 0.169306 | 0.874758 |
| Weissella | Mini.IPIP.Extraversion | 0.102337 | -0.17266 | 0.874758 |
| Mogibacterium | Mini.IPIP.Agreeableness | 0.102948 | -0.17827 | 0.874758 |
| Coprobacillus | BMI | 0.103119 | -0.1639 | 0.874758 |
| Lactovum | Mini.IPIP.Conscientiousness | 0.103322 | -0.1715 | 0.874758 |
| Paralactobacillus | Mini.IPIP.Extraversion | 0.103536 | -0.16698 | 0.874758 |
| Blautia | Mini.IPIP.Conscientiousness | 0.103681 | 0.18255 | 0.874758 |
| Sutterella | Mini.IPIP.Extraversion | 0.104718 | 0.171964 | 0.880253 |
| Eggerthia | Mini.IPIP.Extraversion | 0.105475 | -0.18211 | 0.880843 |
| Atopobium | EDEQ4.Total | 0.105562 | -0.15337 | 0.880843 |
| Enterobacter | PSS.10.score | 0.106793 | 0.173486 | 0.88786 |
| Acetanaerobacterium | Qualtrics.age | 0.109887 | 0.182943 | 0.90262 |
| Methanobrevibacter | EDEQ4.Shape | 0.110242 | 0.17305 | 0.90262 |
| Sporobacterium | EDEQ4.Eating | 0.110259 | -0.17495 | 0.90262 |
| Ruminococcus | PSS.10.score | 0.110827 | 0.169858 | 0.90262 |
| Sporobacterium | Mini.IPIP.Conscientiousness | 0.110853 | 0.176022 | 0.90262 |
| Anaerosporobacter | Height.inches | 0.110945 | 0.173583 | 0.90262 |
| Tepidibacter | BMI | 0.112459 | -0.16964 | 0.904991 |
| Hydrotalea | Height.inches | 0.112554 | -0.16037 | 0.904991 |
| Peptostreptococcus | Height.inches | 0.113007 | -0.17981 | 0.904991 |
| Asaccharobacter | BDI.II.score | 0.113439 | -0.17333 | 0.904991 |
| Metascardovia | Mini.IPIP.Imagination | 0.113775 | 0.163724 | 0.904991 |
| Defluviitalea | BDI.II.score | 0.11389 | -0.17185 | 0.904991 |
| Faecalibacterium | PSS.10.score | 0.114155 | 0.173436 | 0.904991 |
| Acidaminococcus | Weight.lbs | 0.114415 | 0.177244 | 0.904991 |
| Oribacterium | EDEQ4.Eating | 0.11576 | 0.168244 | 0.910994 |
| Turicibacter | Height.inches | 0.115974 | 0.170261 | 0.910994 |
| Anaerofilum | Weight.lbs | 0.116808 | 0.160363 | 0.914392 |
| Blautia | BMI | 0.118962 | -0.16469 | 0.923611 |
| Rothia | Mini.IPIP.Imagination | 0.119046 | -0.15217 | 0.923611 |
| Abiotrophia | Mini.IPIP.Neuroticism | 0.119373 | 0.183506 | 0.923611 |
| Anaerofustis | Weight.lbs | 0.12124 | -0.17405 | 0.923611 |
| Clostridium.XlVb | Height.inches | 0.121251 | -0.16467 | 0.923611 |
| Streptococcus | Mini.IPIP.Extraversion | 0.122355 | -0.16709 | 0.923611 |
| Lactobacillus | EDEQ4.Total | 0.123209 | 0.178503 | 0.923611 |
| Roseburia | Mini.IPIP.Neuroticism | 0.123405 | 0.165698 | 0.923611 |
| Corynebacterium | EDEQ4.Eating | 0.12416 | 0.171492 | 0.923611 |
| Sporobacter | EDEQ4.Shape | 0.124284 | 0.184419 | 0.923611 |
| Corynebacterium | Height.inches | 0.124473 | -0.17168 | 0.923611 |
| Paraprevotella | Height.inches | 0.124473 | -0.15956 | 0.923611 |
| Barnesiella | Height.inches | 0.124805 | 0.163849 | 0.923611 |
| Anaerostipes | BAI.score | 0.124924 | -0.16119 | 0.923611 |
| Streptophyta | PSS.10.score | 0.124932 | -0.17025 | 0.923611 |
| Campylobacter | Mini.IPIP.Conscientiousness | 0.125041 | -0.16456 | 0.923611 |
| Flavonifractor | Mini.IPIP.Neuroticism | 0.12521 | -0.16152 | 0.923611 |
| Natronincola | BMI | 0.125938 | 0.165179 | 0.923611 |
| Gemella | Weight.lbs | 0.126147 | -0.17213 | 0.923611 |
| Weissella | Mini.IPIP.Imagination | 0.126411 | -0.16267 | 0.923611 |
| Megasphaera | BDI.II.score | 0.1265 | 0.171527 | 0.923611 |
| Acetitomaculum | EDEQ4.Total | 0.127241 | 0.158014 | 0.926057 |
| Butyricimonas | PSS.10.score | 0.127922 | 0.169814 | 0.927119 |
| Haemophilus | Mini.IPIP.Extraversion | 0.128393 | 0.175063 | 0.927119 |
| Adlercreutzia | BDI.II.score | 0.129057 | -0.16279 | 0.927119 |
| Rothia | EDEQ4.Weight | 0.129083 | -0.1631 | 0.927119 |
| Coprococcus | EDEQ4.Restraint | 0.129602 | 0.166515 | 0.927119 |
| Syntrophococcus | BMI | 0.129985 | -0.16193 | 0.927119 |
| Slackia | EDEQ4.Eating | 0.13081 | 0.162476 | 0.927119 |
| Prevotella | EDEQ4.Weight | 0.131504 | 0.155693 | 0.927119 |
| Prevotella | Weight.lbs | 0.131821 | -0.16301 | 0.927119 |
| Clostridium.XlVa | BDI.II.score | 0.132423 | -0.16419 | 0.927119 |
| Megasphaera | Mini.IPIP.Agreeableness | 0.132608 | -0.16646 | 0.927119 |
| Roseburia | EDEQ4.Total | 0.132629 | -0.17632 | 0.927119 |
| Desulfovibrio | Mini.IPIP.Agreeableness | 0.132926 | -0.16706 | 0.927119 |
| Olsenella | BDI.II.score | 0.133444 | 0.161657 | 0.927119 |
| Odoribacter | Height.inches | 0.134122 | 0.176253 | 0.927119 |
| Turicibacter | Qualtrics.age | 0.134673 | -0.16619 | 0.927119 |
| Leuconostoc | Height.inches | 0.13478 | -0.15839 | 0.927119 |
| Ruminococcus | Qualtrics.age | 0.135677 | 0.170347 | 0.927119 |
| Paralactobacillus | EDEQ4.Restraint | 0.136497 | 0.155799 | 0.927119 |
| Pseudobutyrivibrio | Qualtrics.age | 0.137133 | 0.158802 | 0.927119 |
| Faecalibacterium | Mini.IPIP.Neuroticism | 0.138429 | 0.162063 | 0.927119 |
| Streptophyta | BAI.score | 0.138551 | -0.1615 | 0.927119 |
| Rothia | Mini.IPIP.Neuroticism | 0.138858 | 0.170732 | 0.927119 |
| Varibaculum | Mini.IPIP.Conscientiousness | 0.139426 | -0.15717 | 0.927119 |
| Enterobacter | EDEQ4.Total | 0.13947 | 0.157526 | 0.927119 |
| Porphyromonas | Weight.lbs | 0.13957 | -0.15416 | 0.927119 |
| Alistipes | EDEQ4.Restraint | 0.139621 | 0.153977 | 0.927119 |
| Pseudoflavonifractor | BAI.score | 0.140099 | -0.15606 | 0.927119 |
| Paludibacter | Qualtrics.age | 0.140686 | -0.16693 | 0.927119 |
| Veillonella | Mini.IPIP.Conscientiousness | 0.140934 | -0.15931 | 0.927119 |
| Marvinbryantia | EDEQ4.Restraint | 0.141686 | 0.162975 | 0.927119 |
| Coprococcus | Mini.IPIP.Imagination | 0.142082 | 0.158536 | 0.927119 |
| Escherichia.Shigella | Height.inches | 0.142504 | -0.15668 | 0.927119 |
| Porphyromonas | Mini.IPIP.Neuroticism | 0.142707 | -0.16202 | 0.927119 |
| Enterorhabdus | Qualtrics.age | 0.14285 | 0.162694 | 0.927119 |
| Granulicatella | Qualtrics.age | 0.142982 | -0.15482 | 0.927119 |
| Gallibacterium | Qualtrics.age | 0.14348 | -0.14822 | 0.927119 |
| Enterorhabdus | EDEQ4.Total | 0.143602 | 0.168655 | 0.927119 |
| Anaerostipes | PSS.10.score | 0.14368 | -0.15488 | 0.927119 |
| shannonDiversity (genus) | EDEQ4.Restraint | 0.143792 | 0.160461 | 0.927119 |
| Clostridium.IV | Height.inches | 0.144074 | 0.167627 | 0.927119 |
| Peptoniphilus | EDEQ4.Eating | 0.14452 | -0.15893 | 0.927372 |
| Papillibacter | Height.inches | 0.145049 | 0.157335 | 0.928152 |
| Varibaculum | EDEQ4.Weight | 0.146463 | 0.156904 | 0.93414 |
| Natronincola | Height.inches | 0.147086 | 0.161518 | 0.93414 |
| Eubacterium | BAI.score | 0.148182 | -0.14765 | 0.93414 |
| Clostridium.XI | BAI.score | 0.148204 | -0.16229 | 0.93414 |
| Pseudobutyrivibrio | Mini.IPIP.Extraversion | 0.14839 | -0.16125 | 0.93414 |
| Candidatus.Carsonella | Mini.IPIP.Conscientiousness | 0.14923 | 0.170852 | 0.93414 |
| Victivallis | Weight.lbs | 0.149247 | 0.157227 | 0.93414 |
| Slackia | EDEQ4.Restraint | 0.14975 | 0.15703 | 0.93414 |
| Anaerofustis | Mini.IPIP.Extraversion | 0.150074 | -0.15376 | 0.93414 |
| Dialister | Mini.IPIP.Neuroticism | 0.150593 | 0.151067 | 0.93414 |
| Actinobacillus | Qualtrics.age | 0.151018 | -0.14503 | 0.93414 |
| Sporobacterium | BDI.II.score | 0.151556 | -0.15216 | 0.93414 |
| Eggerthia | EDEQ4.Eating | 0.152188 | -0.15944 | 0.93414 |
| shannonDiversity (genus) | EDEQ4.Total | 0.152585 | 0.155104 | 0.93414 |
| Eggerthia | EDEQ4.Shape | 0.153258 | -0.15704 | 0.93414 |
| Defluviitalea | BMI | 0.153277 | -0.15853 | 0.93414 |
| Bilophila | Mini.IPIP.Imagination | 0.154161 | -0.15698 | 0.93414 |
| Finegoldia | EDEQ4.Restraint | 0.154172 | -0.15755 | 0.93414 |
| Hallella | EDEQ4.Restraint | 0.154181 | 0.153941 | 0.93414 |
| Bilophila | EDEQ4.Restraint | 0.154307 | 0.147709 | 0.93414 |
| Akkermansia | Weight.lbs | 0.154913 | 0.158449 | 0.93414 |
| Pseudobutyrivibrio | Mini.IPIP.Imagination | 0.155044 | -0.14736 | 0.93414 |
| Peptostreptococcus | Mini.IPIP.Imagination | 0.156235 | 0.155009 | 0.93414 |
| Blautia | Height.inches | 0.156656 | -0.14905 | 0.93414 |
| Ethanoligenens | Mini.IPIP.Agreeableness | 0.156732 | 0.155383 | 0.93414 |
| Roseburia | EDEQ4.Restraint | 0.156877 | -0.14844 | 0.93414 |
| Clostridium.XlVa | Weight.lbs | 0.157057 | -0.15627 | 0.93414 |
| Sarcina | Qualtrics.age | 0.158223 | 0.146926 | 0.936429 |
| Gemella | Qualtrics.age | 0.158465 | -0.1572 | 0.936429 |
| Lactobacillus | EDEQ4.Restraint | 0.1589 | 0.153564 | 0.936429 |
| Ruminococcus2 | EDEQ4.Weight | 0.159318 | 0.151314 | 0.936429 |
| Peptoniphilus | EDEQ4.Restraint | 0.159497 | -0.15668 | 0.936429 |
| Syntrophococcus | EDEQ4.Weight | 0.161369 | 0.152329 | 0.939027 |
| Actinobacillus | Mini.IPIP.Imagination | 0.162563 | -0.14637 | 0.939027 |
| Peptostreptococcus | BDI.II.score | 0.162574 | -0.15236 | 0.939027 |
| Anaerotruncus | BMI | 0.16322 | -0.15726 | 0.939027 |
| Anaerofustis | Qualtrics.age | 0.163505 | -0.14818 | 0.939027 |
| Lactobacillus | EDEQ4.Weight | 0.16354 | 0.150859 | 0.939027 |
| Lactococcus | Qualtrics.age | 0.163653 | -0.16243 | 0.939027 |
| Syntrophococcus | Mini.IPIP.Agreeableness | 0.164287 | -0.13828 | 0.939027 |
| Candidatus.Carsonella | BMI | 0.164581 | -0.13275 | 0.939027 |
| Dialister | Qualtrics.age | 0.164777 | -0.15822 | 0.939027 |
| Enterobacter | Height.inches | 0.164821 | 0.151602 | 0.939027 |
| Eubacterium | PSS.10.score | 0.164886 | -0.15069 | 0.939027 |
| Murdochiella | Mini.IPIP.Neuroticism | 0.166796 | -0.15401 | 0.942423 |
| Actinobacillus | Mini.IPIP.Neuroticism | 0.167816 | 0.149929 | 0.942423 |
| Sporacetigenium | BDI.II.score | 0.168123 | 0.156755 | 0.942423 |
| Haemophilus | BDI.II.score | 0.168712 | 0.163453 | 0.942423 |
| Gordonibacter | Mini.IPIP.Imagination | 0.168785 | -0.15846 | 0.942423 |
| Eggerthella | EDEQ4.Restraint | 0.169645 | -0.14462 | 0.942423 |
| Dorea | Height.inches | 0.170054 | -0.15984 | 0.942423 |
| Gordonibacter | EDEQ4.Eating | 0.170069 | -0.15448 | 0.942423 |
| Victivallis | PSS.10.score | 0.170492 | -0.14641 | 0.942423 |
| Anaerosporobacter | PSS.10.score | 0.170559 | -0.14125 | 0.942423 |
| Clostridium.XlVb | EDEQ4.Weight | 0.170675 | 0.153409 | 0.942423 |
| Sutterella | EDEQ4.Restraint | 0.171299 | 0.14823 | 0.942423 |
| Roseburia | EDEQ4.Shape | 0.171346 | -0.14024 | 0.942423 |
| Paraprevotella | Mini.IPIP.Agreeableness | 0.171677 | 0.137296 | 0.942423 |
| Hallella | EDEQ4.Shape | 0.171868 | 0.159093 | 0.942423 |
| Leuconostoc | Mini.IPIP.Conscientiousness | 0.172138 | -0.15518 | 0.942423 |
| Clostridium.XlVa | EDEQ4.Restraint | 0.173262 | -0.14664 | 0.942423 |
| Anaerovorax | Mini.IPIP.Conscientiousness | 0.173675 | -0.13524 | 0.942423 |
| Eggerthella | BDI.II.score | 0.173688 | -0.14677 | 0.942423 |
| Blautia | Qualtrics.age | 0.173899 | -0.14713 | 0.942423 |
| Acetivibrio | Mini.IPIP.Extraversion | 0.17417 | 0.14901 | 0.942423 |
| Moryella | BDI.II.score | 0.174839 | -0.14313 | 0.943799 |
| Syntrophococcus | Qualtrics.age | 0.176116 | -0.14703 | 0.946714 |
| Catenibacterium | Mini.IPIP.Imagination | 0.177002 | -0.14839 | 0.946714 |
| Anaerostipes | Mini.IPIP.Imagination | 0.177002 | 0.15926 | 0.946714 |
| Peptostreptococcus | BMI | 0.177112 | -0.14574 | 0.946714 |
| Lachnospira | EDEQ4.Eating | 0.177661 | 0.148779 | 0.946714 |
| Metascardovia | EDEQ4.Eating | 0.177873 | -0.14796 | 0.946714 |
| Hydrogenoanaerobacterium | EDEQ4.Restraint | 0.179188 | 0.152737 | 0.947879 |
| Anaerotruncus | Height.inches | 0.179373 | 0.146602 | 0.947879 |
| Desulfovibrio | EDEQ4.Shape | 0.180092 | 0.143674 | 0.947879 |
| Corynebacterium | PSS.10.score | 0.180334 | 0.148835 | 0.947879 |
| Blautia | Mini.IPIP.Extraversion | 0.180581 | -0.13918 | 0.947879 |
| Saccharibacteria_genera_incertae_sedis | BAI.score | 0.181571 | 0.151175 | 0.947879 |
| Turicibacter | Mini.IPIP.Neuroticism | 0.182079 | 0.153251 | 0.947879 |
| Blautia | Mini.IPIP.Neuroticism | 0.182126 | -0.14517 | 0.947879 |
| Solobacterium | Height.inches | 0.182463 | -0.14495 | 0.947879 |
| Clostridium.IV | EDEQ4.Weight | 0.182628 | 0.154244 | 0.947879 |
| Sporacetigenium | BMI | 0.183189 | 0.144254 | 0.947879 |
| Tannerella | BAI.score | 0.184038 | -0.1379 | 0.947879 |
| Oxalobacter | Height.inches | 0.18404 | 0.14278 | 0.947879 |
| Sporobacter | Mini.IPIP.Imagination | 0.184207 | -0.14023 | 0.947879 |
| Parvimonas | BDI.II.score | 0.184353 | -0.14262 | 0.947879 |
| Butyricicoccus | EDEQ4.Weight | 0.185091 | -0.14712 | 0.947879 |
| Lactobacillus | Mini.IPIP.Neuroticism | 0.18551 | 0.14655 | 0.947879 |
| Oscillibacter | EDEQ4.Shape | 0.185581 | -0.14661 | 0.947879 |
| Murdochiella | PSS.10.score | 0.186058 | -0.14361 | 0.94819 |
| Anaerostipes | EDEQ4.Total | 0.186693 | -0.14148 | 0.949302 |
| Acidaminococcus | EDEQ4.Eating | 0.187532 | 0.151645 | 0.950488 |
| Collinsella | EDEQ4.Restraint | 0.188297 | 0.148476 | 0.950488 |
| Adlercreutzia | Mini.IPIP.Conscientiousness | 0.188355 | 0.14392 | 0.950488 |
| Lactonifactor | Mini.IPIP.Neuroticism | 0.189478 | -0.14693 | 0.950488 |
| Granulicatella | BAI.score | 0.189902 | 0.141922 | 0.950488 |
| Faecalibacterium | BMI | 0.190303 | 0.156724 | 0.950488 |
| Metascardovia | BAI.score | 0.190348 | -0.13337 | 0.950488 |
| Anaerotruncus | Qualtrics.age | 0.190678 | 0.140006 | 0.950488 |
| Metascardovia | Mini.IPIP.Neuroticism | 0.190682 | -0.14746 | 0.950488 |
| Porphyromonas | PSS.10.score | 0.191624 | -0.13857 | 0.9531 |
| Pseudobutyrivibrio | Mini.IPIP.Agreeableness | 0.192092 | -0.15067 | 0.953345 |
| Methanobrevibacter | EDEQ4.Restraint | 0.192955 | 0.144888 | 0.955549 |
| Lachnoanaerobaculum | BMI | 0.194155 | 0.128598 | 0.956598 |
| Subdoligranulum | Height.inches | 0.194265 | 0.151782 | 0.956598 |
| Phascolarctobacterium | BDI.II.score | 0.194427 | -0.14063 | 0.956598 |
| Candidatus.Carsonella | Mini.IPIP.Imagination | 0.195707 | 0.139933 | 0.959419 |
| Roseburia | Height.inches | 0.196818 | -0.1512 | 0.959419 |
| Clostridium.XVIII | BDI.II.score | 0.196981 | 0.141067 | 0.959419 |
| Clostridium.XVIII | Weight.lbs | 0.197025 | -0.14279 | 0.959419 |
| Oxalobacter | EDEQ4.Eating | 0.197312 | 0.136431 | 0.959419 |
| Sporobacterium | Mini.IPIP.Imagination | 0.198575 | -0.13913 | 0.959419 |
| Paraprevotella | EDEQ4.Shape | 0.198846 | 0.133249 | 0.959419 |
| Slackia | EDEQ4.Shape | 0.198892 | 0.135238 | 0.959419 |
| Clostridium.sensu.stricto | Mini.IPIP.Imagination | 0.199135 | -0.13766 | 0.959419 |
| Pseudoflavonifractor | Mini.IPIP.Extraversion | 0.199212 | -0.14771 | 0.959419 |
| Rothia | Height.inches | 0.200066 | 0.14184 | 0.960809 |
| Desulfovibrio | EDEQ4.Total | 0.200344 | 0.134344 | 0.960809 |
| Papillibacter | EDEQ4.Eating | 0.20116 | -0.14079 | 0.961388 |
| Akkermansia | BAI.score | 0.201874 | 0.139212 | 0.961388 |
| Candidatus.Carsonella | Height.inches | 0.202316 | 0.136247 | 0.961388 |
| Eggerthia | Height.inches | 0.202323 | 0.146459 | 0.961388 |
| Bilophila | EDEQ4.Weight | 0.20266 | 0.142374 | 0.961388 |
| Lactobacillus | EDEQ4.Shape | 0.202997 | 0.165277 | 0.961388 |
| Prevotella | Mini.IPIP.Agreeableness | 0.204747 | -0.14257 | 0.967663 |
| Streptophyta | EDEQ4.Eating | 0.205283 | -0.1368 | 0.96819 |
| Adlercreutzia | EDEQ4.Eating | 0.206397 | -0.14245 | 0.968937 |
| Lactovum | Mini.IPIP.Extraversion | 0.206513 | 0.131885 | 0.968937 |
| Alistipes | EDEQ4.Total | 0.207043 | 0.136151 | 0.968937 |
| Bacteroides | Mini.IPIP.Neuroticism | 0.207481 | -0.14709 | 0.968937 |
| Candidatus.Carsonella | Qualtrics.age | 0.207785 | -0.14499 | 0.968937 |
| Actinobacillus | BAI.score | 0.208081 | 0.129735 | 0.968937 |
| Phascolarctobacterium | EDEQ4.Shape | 0.208545 | 0.134598 | 0.968937 |
| Varibaculum | EDEQ4.Total | 0.209462 | 0.12761 | 0.968937 |
| Hydrogenoanaerobacterium | BAI.score | 0.2096 | -0.13212 | 0.968937 |
| Akkermansia | EDEQ4.Total | 0.209695 | 0.144402 | 0.968937 |
| Acetitomaculum | EDEQ4.Shape | 0.210369 | 0.137579 | 0.969872 |
| Eggerthella | Mini.IPIP.Neuroticism | 0.211492 | -0.14456 | 0.969872 |
| Robinsoniella | Weight.lbs | 0.211619 | -0.12655 | 0.969872 |
| Peptoniphilus | Qualtrics.age | 0.212534 | -0.13594 | 0.969872 |
| shannonDiversity (genus) | Height.inches | 0.212657 | 0.137076 | 0.969872 |
| Lactonifactor | Height.inches | 0.212657 | 0.137297 | 0.969872 |
| Clostridium.XlVb | Mini.IPIP.Conscientiousness | 0.213679 | 0.134154 | 0.969872 |
| Holdemania | EDEQ4.Weight | 0.213815 | 0.128207 | 0.969872 |
| Slackia | EDEQ4.Total | 0.213819 | 0.132703 | 0.969872 |
| Anaerofustis | Mini.IPIP.Imagination | 0.214301 | -0.12148 | 0.969872 |
| Acetivibrio | Weight.lbs | 0.214875 | 0.139093 | 0.969872 |
| Sporobacterium | Mini.IPIP.Agreeableness | 0.215007 | -0.13615 | 0.969872 |
| Haemophilus | Mini.IPIP.Neuroticism | 0.216556 | 0.132983 | 0.971241 |
| Veillonella | EDEQ4.Weight | 0.216579 | -0.12945 | 0.971241 |
| Alistipes | EDEQ4.Weight | 0.216589 | 0.133015 | 0.971241 |
| Lactobacillus | Mini.IPIP.Agreeableness | 0.217994 | -0.13549 | 0.972187 |
| Anaerostipes | BMI | 0.219082 | -0.13067 | 0.972187 |
| Clostridium.XlVb | Mini.IPIP.Extraversion | 0.219217 | 0.139421 | 0.972187 |
| Corynebacterium | Weight.lbs | 0.219343 | -0.13636 | 0.972187 |
| Roseburia | EDEQ4.Weight | 0.219379 | -0.14722 | 0.972187 |
| Ruminococcus | EDEQ4.Shape | 0.219387 | 0.121185 | 0.972187 |
| Dorea | PSS.10.score | 0.219788 | -0.14108 | 0.972187 |
| Pseudoflavonifractor | PSS.10.score | 0.221169 | -0.13759 | 0.973124 |
| Clostridium.XlVb | EDEQ4.Restraint | 0.221213 | 0.13101 | 0.973124 |
| Eggerthia | EDEQ4.Weight | 0.221487 | -0.13448 | 0.973124 |
| Parasutterella | BMI | 0.221708 | 0.135532 | 0.973124 |
| Tepidibacter | Mini.IPIP.Neuroticism | 0.222513 | -0.13297 | 0.974373 |
| Natronincola | Mini.IPIP.Imagination | 0.22366 | 0.126946 | 0.974373 |
| Peptoniphilus | Weight.lbs | 0.223715 | -0.13837 | 0.974373 |
| Syntrophococcus | Mini.IPIP.Extraversion | 0.223978 | -0.13372 | 0.974373 |
| Sporobacter | EDEQ4.Restraint | 0.224131 | 0.136262 | 0.974373 |
| Acetanaerobacterium | BDI.II.score | 0.225285 | -0.1314 | 0.976896 |
| Clostridium.III | Mini.IPIP.Imagination | 0.225569 | -0.12993 | 0.976896 |
| Ruminococcus | Mini.IPIP.Neuroticism | 0.226693 | 0.124615 | 0.979897 |
| Finegoldia | Weight.lbs | 0.227429 | -0.13445 | 0.981219 |
| Varibaculum | Height.inches | 0.228702 | -0.1417 | 0.984844 |
| Coprobacillus | BAI.score | 0.230449 | 0.125532 | 0.985771 |
| Acidaminococcus | EDEQ4.Weight | 0.231118 | 0.133376 | 0.985771 |
| Gemmiger | Mini.IPIP.Extraversion | 0.233328 | -0.12341 | 0.985771 |
| Methanobrevibacter | EDEQ4.Eating | 0.233796 | 0.129129 | 0.985771 |
| Atopobium | Mini.IPIP.Conscientiousness | 0.234251 | -0.12814 | 0.985771 |
| Acidaminococcus | BMI | 0.234637 | 0.129855 | 0.985771 |
| Sporobacterium | Height.inches | 0.235094 | 0.128022 | 0.985771 |
| Parasporobacterium | EDEQ4.Eating | 0.23559 | 0.126883 | 0.985771 |
| Porphyromonas | Mini.IPIP.Imagination | 0.235616 | -0.13287 | 0.985771 |
| Eggerthia | EDEQ4.Restraint | 0.235751 | -0.12435 | 0.985771 |
| Porphyromonas | Mini.IPIP.Agreeableness | 0.236112 | 0.128764 | 0.985771 |
| Clostridium.XlVa | EDEQ4.Shape | 0.236399 | -0.14902 | 0.985771 |
| Asaccharobacter | EDEQ4.Eating | 0.239261 | -0.13217 | 0.985771 |
| Akkermansia | EDEQ4.Weight | 0.239627 | 0.135426 | 0.985771 |
| Victivallis | Mini.IPIP.Conscientiousness | 0.239644 | 0.125542 | 0.985771 |
| Hydrogenoanaerobacterium | EDEQ4.Total | 0.240443 | 0.115141 | 0.985771 |
| Clostridium.XlVa | EDEQ4.Eating | 0.241077 | -0.13519 | 0.985771 |
| Corynebacterium | EDEQ4.Restraint | 0.241189 | 0.125758 | 0.985771 |
| Holdemania | PSS.10.score | 0.241192 | -0.12594 | 0.985771 |
| Lactonifactor | BDI.II.score | 0.241237 | -0.13381 | 0.985771 |
| Parasporobacterium | Mini.IPIP.Extraversion | 0.241804 | 0.124477 | 0.985771 |
| Subdoligranulum | BMI | 0.24189 | -0.13372 | 0.985771 |
| Paludibacter | Mini.IPIP.Conscientiousness | 0.242604 | 0.126438 | 0.985771 |
| Acetitomaculum | EDEQ4.Weight | 0.242931 | 0.120209 | 0.985771 |
| Haemophilus | Mini.IPIP.Imagination | 0.243385 | -0.12772 | 0.985771 |
| Lactonifactor | BAI.score | 0.243786 | -0.12645 | 0.985771 |
| shannonDiversity (genus) | Mini.IPIP.Neuroticism | 0.244143 | 0.140519 | 0.985771 |
| Streptococcus | Mini.IPIP.Neuroticism | 0.244143 | 0.132242 | 0.985771 |
| Parabacteroides | BMI | 0.244854 | 0.13734 | 0.985771 |
| Oribacterium | PSS.10.score | 0.246636 | 0.125515 | 0.985771 |
| Clostridium.IV | Weight.lbs | 0.246962 | 0.112437 | 0.985771 |
| Lactococcus | EDEQ4.Restraint | 0.248463 | 0.126285 | 0.985771 |
| Sporobacterium | Weight.lbs | 0.248488 | 0.12152 | 0.985771 |
| Clostridium.XlVa | PSS.10.score | 0.248658 | -0.12828 | 0.985771 |
| Paraprevotella | Weight.lbs | 0.24866 | -0.12336 | 0.985771 |
| Tepidibacter | EDEQ4.Restraint | 0.249294 | -0.13451 | 0.985771 |
| Butyricicoccus | EDEQ4.Total | 0.249443 | -0.1191 | 0.985771 |
| Akkermansia | Mini.IPIP.Conscientiousness | 0.251145 | 0.124502 | 0.985771 |
| Anaerofustis | EDEQ4.Restraint | 0.251307 | -0.13121 | 0.985771 |
| Parvimonas | Mini.IPIP.Conscientiousness | 0.251773 | 0.114471 | 0.985771 |
| Olsenella | BMI | 0.253156 | 0.124915 | 0.985771 |
| Anaerococcus | Weight.lbs | 0.253635 | -0.11906 | 0.985771 |
| Victivallis | Qualtrics.age | 0.254153 | 0.118768 | 0.985771 |
| Turicibacter | EDEQ4.Restraint | 0.256398 | -0.12452 | 0.985771 |
| Marvinbryantia | EDEQ4.Weight | 0.256425 | -0.1277 | 0.985771 |
| Paralactobacillus | EDEQ4.Total | 0.256442 | 0.128336 | 0.985771 |
| Enterobacter | EDEQ4.Shape | 0.256538 | 0.125131 | 0.985771 |
| Acetitomaculum | Height.inches | 0.256784 | -0.12146 | 0.985771 |
| Leuconostoc | BAI.score | 0.256942 | 0.122345 | 0.985771 |
| Roseburia | Mini.IPIP.Extraversion | 0.257226 | 0.124816 | 0.985771 |
| Bifidobacterium | PSS.10.score | 0.257815 | -0.12146 | 0.985771 |
| Fusobacterium | PSS.10.score | 0.258498 | 0.12196 | 0.985771 |
| Anaerofustis | PSS.10.score | 0.258808 | -0.11504 | 0.985771 |
| Streptococcus | Mini.IPIP.Imagination | 0.259013 | -0.11824 | 0.985771 |
| Lachnospira | BDI.II.score | 0.259214 | -0.11813 | 0.985771 |
| Granulicatella | EDEQ4.Total | 0.259818 | -0.12432 | 0.985771 |
| Clostridium.III | BMI | 0.260179 | -0.11886 | 0.985771 |
| Parasutterella | Mini.IPIP.Imagination | 0.260436 | 0.133739 | 0.985771 |
| Parvimonas | EDEQ4.Eating | 0.260517 | -0.12321 | 0.985771 |
| Ethanoligenens | Qualtrics.age | 0.260716 | -0.12509 | 0.985771 |
| Oscillibacter | Qualtrics.age | 0.260768 | 0.133253 | 0.985771 |
| Catenibacterium | PSS.10.score | 0.260918 | 0.113604 | 0.985771 |
| Holdemania | BDI.II.score | 0.261069 | -0.12665 | 0.985771 |
| Coprococcus | BDI.II.score | 0.262652 | -0.11738 | 0.985771 |
| Sporacetigenium | PSS.10.score | 0.262985 | 0.117742 | 0.985771 |
| Pyrolobus | EDEQ4.Shape | 0.263322 | 0.124198 | 0.985771 |
| Parasporobacterium | BAI.score | 0.264058 | -0.13141 | 0.985771 |
| Turicibacter | BDI.II.score | 0.264179 | 0.120738 | 0.985771 |
| Finegoldia | EDEQ4.Total | 0.264373 | -0.12525 | 0.985771 |
| Parabacteroides | Mini.IPIP.Imagination | 0.265291 | -0.12255 | 0.985771 |
| Methanobrevibacter | BDI.II.score | 0.266208 | 0.131352 | 0.985771 |
| Metascardovia | PSS.10.score | 0.267018 | -0.12226 | 0.985771 |
| Hydrotalea | EDEQ4.Weight | 0.267109 | 0.134104 | 0.985771 |
| Clostridium.XVIII | Height.inches | 0.26883 | -0.12186 | 0.985771 |
| Barnesiella | BMI | 0.269435 | 0.115951 | 0.985771 |
| Lactonifactor | BMI | 0.269488 | -0.09907 | 0.985771 |
| Escherichia.Shigella | BDI.II.score | 0.269939 | 0.121381 | 0.985771 |
| Hydrogenoanaerobacterium | EDEQ4.Shape | 0.270118 | 0.116158 | 0.985771 |
| Clostridium.XI | EDEQ4.Weight | 0.270785 | -0.13082 | 0.985771 |
| Megamonas | Mini.IPIP.Neuroticism | 0.270895 | -0.12162 | 0.985771 |
| Parasutterella | Qualtrics.age | 0.271693 | 0.116444 | 0.985771 |
| Acetivibrio | Mini.IPIP.Neuroticism | 0.271712 | -0.1213 | 0.985771 |
| Mogibacterium | Height.inches | 0.271958 | 0.122435 | 0.985771 |
| Veillonella | Mini.IPIP.Agreeableness | 0.272173 | -0.11356 | 0.985771 |
| Weissella | BAI.score | 0.273466 | 0.12136 | 0.985771 |
| Solobacterium | BMI | 0.273607 | 0.119598 | 0.985771 |
| Porphyromonas | BMI | 0.273772 | -0.11141 | 0.985771 |
| Eubacterium | EDEQ4.Weight | 0.274021 | -0.1209 | 0.985771 |
| Clostridium.XlVa | EDEQ4.Total | 0.274386 | -0.13321 | 0.985771 |
| Pyrolobus | PSS.10.score | 0.274681 | 0.123755 | 0.985771 |
| Gemella | EDEQ4.Total | 0.274893 | -0.10609 | 0.985771 |
| Roseburia | BAI.score | 0.274919 | 0.110331 | 0.985771 |
| Dorea | Mini.IPIP.Neuroticism | 0.275282 | -0.11346 | 0.985771 |
| Clostridium.III | Height.inches | 0.275438 | 0.125535 | 0.985771 |
| Sutterella | Mini.IPIP.Imagination | 0.27593 | -0.10385 | 0.985771 |
| Phascolarctobacterium | PSS.10.score | 0.276823 | -0.11783 | 0.985771 |
| Ethanoligenens | BDI.II.score | 0.278679 | -0.12297 | 0.985771 |
| Anaerovorax | EDEQ4.Restraint | 0.279451 | 0.129886 | 0.985771 |
| Erysipelotrichaceae_incertae_sedis | Mini.IPIP.Conscientiousness | 0.279629 | -0.10394 | 0.985771 |
| Lachnospiracea_incertae_sedis | Qualtrics.age | 0.27993 | -0.12303 | 0.985771 |
| Leuconostoc | BMI | 0.280416 | -0.11708 | 0.985771 |
| Metascardovia | Height.inches | 0.280702 | 0.123764 | 0.985771 |
| Alistipes | Weight.lbs | 0.281525 | 0.126976 | 0.985771 |
| Odoribacter | BDI.II.score | 0.281937 | -0.10802 | 0.985771 |
| Clostridium.sensu.stricto | Mini.IPIP.Extraversion | 0.28263 | 0.114609 | 0.985771 |
| Subdoligranulum | EDEQ4.Shape | 0.282948 | -0.11338 | 0.985771 |
| Parasporobacterium | BMI | 0.283141 | 0.117162 | 0.985771 |
| Clostridium.XlVa | Height.inches | 0.28517 | -0.12858 | 0.985771 |
| Hespellia | EDEQ4.Shape | 0.285188 | 0.104701 | 0.985771 |
| Pyrolobus | Mini.IPIP.Extraversion | 0.285391 | -0.11237 | 0.985771 |
| Hydrotalea | EDEQ4.Total | 0.285474 | 0.132382 | 0.985771 |
| Granulicatella | EDEQ4.Shape | 0.286025 | -0.10889 | 0.985771 |
| Clostridium.IV | EDEQ4.Eating | 0.286213 | -0.12008 | 0.985771 |
| Megasphaera | EDEQ4.Shape | 0.287841 | 0.116 | 0.985771 |
| Clostridium.XI | Weight.lbs | 0.288146 | 0.125451 | 0.985771 |
| Eggerthia | Qualtrics.age | 0.288179 | -0.11125 | 0.985771 |
| Saccharibacteria_genera_incertae_sedis | Mini.IPIP.Imagination | 0.288273 | -0.11695 | 0.985771 |
| Paraprevotella | EDEQ4.Weight | 0.28842 | -0.11292 | 0.985771 |
| Coprobacillus | Weight.lbs | 0.288511 | -0.10525 | 0.985771 |
| Weissella | BMI | 0.288627 | 0.122013 | 0.985771 |
| Moryella | PSS.10.score | 0.289106 | -0.13428 | 0.985771 |
| Prevotella | Mini.IPIP.Conscientiousness | 0.289582 | -0.12998 | 0.985771 |
| Subdoligranulum | Qualtrics.age | 0.289861 | 0.109108 | 0.985771 |
| Clostridium.XI | Qualtrics.age | 0.289862 | -0.10822 | 0.985771 |
| shannonDiversity (genus) | EDEQ4.Weight | 0.290758 | 0.128139 | 0.985771 |
| Paraprevotella | Qualtrics.age | 0.290768 | -0.11582 | 0.985771 |
| Clostridium.XlVb | EDEQ4.Total | 0.290813 | 0.115549 | 0.985771 |
| Ruminococcus | Mini.IPIP.Imagination | 0.291447 | -0.11302 | 0.985771 |
| Parabacteroides | EDEQ4.Restraint | 0.291862 | 0.115473 | 0.985771 |
| Faecalibacterium | BDI.II.score | 0.292065 | 0.107678 | 0.985771 |
| Peptostreptococcus | Mini.IPIP.Agreeableness | 0.292113 | 0.105229 | 0.985771 |
| Lactococcus | EDEQ4.Shape | 0.292945 | -0.12564 | 0.985771 |
| Holdemania | Mini.IPIP.Neuroticism | 0.293507 | -0.12087 | 0.985771 |
| Robinsoniella | EDEQ4.Shape | 0.293701 | 0.10313 | 0.985771 |
| Porphyromonas | Height.inches | 0.294535 | -0.11415 | 0.985771 |
| Faecalibacterium | Mini.IPIP.Imagination | 0.294834 | -0.11402 | 0.985771 |
| Leuconostoc | Mini.IPIP.Neuroticism | 0.295058 | 0.112903 | 0.985771 |
| Olsenella | Mini.IPIP.Neuroticism | 0.295882 | 0.1094 | 0.985771 |
| Fusobacterium | Weight.lbs | 0.296275 | -0.11044 | 0.985771 |
| Streptophyta | Height.inches | 0.296827 | 0.105844 | 0.985771 |
| Desulfovibrio | Mini.IPIP.Imagination | 0.296941 | -0.11242 | 0.985771 |
| Veillonella | EDEQ4.Eating | 0.297382 | 0.115017 | 0.985771 |
| Anaerovorax | Mini.IPIP.Imagination | 0.29781 | -0.10704 | 0.985771 |
| Finegoldia | EDEQ4.Weight | 0.298019 | -0.11689 | 0.985771 |
| Blautia | Mini.IPIP.Imagination | 0.298248 | 0.11121 | 0.985771 |
| Acetivibrio | EDEQ4.Restraint | 0.298439 | 0.107202 | 0.985771 |
| Ruminococcus | Height.inches | 0.298715 | 0.116592 | 0.985771 |
| Subdoligranulum | EDEQ4.Restraint | 0.298964 | 0.124278 | 0.985771 |
| Parasutterella | Mini.IPIP.Conscientiousness | 0.299655 | 0.110084 | 0.985771 |
| Paraprevotella | Mini.IPIP.Neuroticism | 0.299743 | -0.10682 | 0.985771 |
| Pseudoflavonifractor | Weight.lbs | 0.299979 | 0.099642 | 0.985771 |
| Eggerthella | Height.inches | 0.300435 | -0.11671 | 0.985771 |
| Akkermansia | Mini.IPIP.Agreeableness | 0.300578 | -0.11441 | 0.985771 |
| Robinsoniella | BAI.score | 0.302532 | -0.12741 | 0.985771 |
| Clostridium.XVIII | BMI | 0.302601 | -0.10602 | 0.985771 |
| Porphyromonas | Qualtrics.age | 0.302924 | -0.11567 | 0.985771 |
| Dialister | EDEQ4.Shape | 0.3032 | -0.11668 | 0.985771 |
| Clostridium.III | Qualtrics.age | 0.303845 | 0.107506 | 0.985771 |
| Sporobacterium | EDEQ4.Restraint | 0.303903 | 0.109571 | 0.985771 |
| Hydrogenoanaerobacterium | PSS.10.score | 0.304 | -0.10993 | 0.985771 |
| Gordonibacter | Mini.IPIP.Conscientiousness | 0.304895 | 0.113273 | 0.985771 |
| Butyricicoccus | Weight.lbs | 0.305154 | 0.108336 | 0.985771 |
| Lachnoanaerobaculum | EDEQ4.Shape | 0.305169 | 0.111748 | 0.985771 |
| Butyricimonas | BDI.II.score | 0.30599 | 0.112319 | 0.985771 |
| Turicibacter | BMI | 0.306002 | -0.12047 | 0.985771 |
| Campylobacter | BMI | 0.306393 | 0.110886 | 0.985771 |
| Eggerthella | EDEQ4.Eating | 0.306925 | -0.11133 | 0.985771 |
| Abiotrophia | EDEQ4.Weight | 0.307644 | 0.115882 | 0.985771 |
| Eggerthella | BAI.score | 0.308659 | 0.107124 | 0.985771 |
| Faecalibacterium | EDEQ4.Eating | 0.308858 | 0.116489 | 0.985771 |
| Sporacetigenium | Mini.IPIP.Extraversion | 0.308908 | 0.110335 | 0.985771 |
| Anaerostipes | Mini.IPIP.Neuroticism | 0.309028 | -0.11131 | 0.985771 |
| Streptococcus | Height.inches | 0.309152 | -0.11735 | 0.985771 |
| Eubacterium | Mini.IPIP.Agreeableness | 0.309265 | 0.118515 | 0.985771 |
| Moryella | EDEQ4.Weight | 0.309582 | 0.111713 | 0.985771 |
| Faecalibacterium | Mini.IPIP.Conscientiousness | 0.310232 | -0.10559 | 0.985771 |
| Subdoligranulum | Mini.IPIP.Conscientiousness | 0.310232 | 0.112311 | 0.985771 |
| Tannerella | Mini.IPIP.Conscientiousness | 0.310331 | 0.10977 | 0.985771 |
| Shuttleworthia | Mini.IPIP.Imagination | 0.311201 | -0.11111 | 0.985771 |
| Butyricimonas | Mini.IPIP.Agreeableness | 0.311245 | 0.110607 | 0.985771 |
| Butyricimonas | BMI | 0.311438 | 0.101947 | 0.985771 |
| Marvinbryantia | EDEQ4.Shape | 0.311905 | 0.112334 | 0.985771 |
| Gordonibacter | Height.inches | 0.312595 | 0.102077 | 0.985771 |
| Hallella | Mini.IPIP.Conscientiousness | 0.313399 | -0.09911 | 0.985771 |
| Odoribacter | Weight.lbs | 0.313812 | 0.106974 | 0.985771 |
| Acidaminococcus | Mini.IPIP.Agreeableness | 0.31616 | -0.10904 | 0.985771 |
| Gardnerella | Mini.IPIP.Agreeableness | 0.316244 | 0.121167 | 0.985771 |
| Rothia | Mini.IPIP.Extraversion | 0.316363 | 0.109436 | 0.985771 |
| Sarcina | PSS.10.score | 0.316405 | 0.101943 | 0.985771 |
| Gardnerella | EDEQ4.Eating | 0.316535 | 0.110732 | 0.985771 |
| Methanobrevibacter | Weight.lbs | 0.317039 | 0.099611 | 0.985771 |
| Anaerostipes | Mini.IPIP.Extraversion | 0.317045 | 0.113419 | 0.985771 |
| Streptophyta | Mini.IPIP.Neuroticism | 0.317765 | -0.10554 | 0.985771 |
| Clostridium.XVIII | Mini.IPIP.Agreeableness | 0.318171 | 0.109924 | 0.985771 |
| Ruminococcus2 | EDEQ4.Total | 0.318447 | 0.104737 | 0.985771 |
| Methanobrevibacter | Qualtrics.age | 0.318903 | 0.101395 | 0.985771 |
| Veillonella | EDEQ4.Shape | 0.318995 | -0.10045 | 0.985771 |
| Anaerorhabdus | Mini.IPIP.Extraversion | 0.319157 | -0.11488 | 0.985771 |
| Gallibacterium | EDEQ4.Shape | 0.319919 | 0.115586 | 0.985771 |
| Pseudobutyrivibrio | PSS.10.score | 0.319932 | -0.11476 | 0.985771 |
| Campylobacter | Qualtrics.age | 0.320168 | -0.11423 | 0.985771 |
| Ruminococcus2 | EDEQ4.Restraint | 0.320956 | 0.103947 | 0.985771 |
| Hydrotalea | EDEQ4.Shape | 0.321281 | 0.117985 | 0.985771 |
| Eggerthia | Weight.lbs | 0.321484 | -0.09631 | 0.985771 |
| Marvinbryantia | BDI.II.score | 0.321794 | -0.11433 | 0.985771 |
| Ruminococcus | EDEQ4.Total | 0.322018 | 0.096605 | 0.985771 |
| Metascardovia | BDI.II.score | 0.322121 | -0.11416 | 0.985771 |
| Acetivibrio | PSS.10.score | 0.322401 | -0.1026 | 0.985771 |
| Pseudoflavonifractor | EDEQ4.Shape | 0.322588 | -0.09273 | 0.985771 |
| shannonDiversity (genus) | Mini.IPIP.Imagination | 0.32288 | -0.1019 | 0.985771 |
| Moryella | Weight.lbs | 0.324344 | -0.11271 | 0.985771 |
| Anaerosporobacter | EDEQ4.Weight | 0.324352 | 0.123136 | 0.985771 |
| Porphyromonas | Mini.IPIP.Extraversion | 0.32492 | -0.11073 | 0.985771 |
| Pseudobutyrivibrio | Mini.IPIP.Conscientiousness | 0.325477 | 0.101766 | 0.985771 |
| Sarcina | EDEQ4.Shape | 0.325778 | 0.12043 | 0.985771 |
| Abiotrophia | Mini.IPIP.Conscientiousness | 0.325907 | 0.105268 | 0.985771 |
| Oribacterium | Mini.IPIP.Agreeableness | 0.326694 | -0.09852 | 0.985771 |
| Natronincola | EDEQ4.Shape | 0.326701 | 0.105503 | 0.985771 |
| Sutterella | EDEQ4.Total | 0.326968 | 0.108659 | 0.985771 |
| Slackia | Height.inches | 0.328241 | 0.110139 | 0.985771 |
| Olsenella | EDEQ4.Shape | 0.32831 | 0.111444 | 0.985771 |
| Mogibacterium | Mini.IPIP.Neuroticism | 0.328618 | -0.08257 | 0.985771 |
| Enterobacter | Mini.IPIP.Neuroticism | 0.329386 | 0.104176 | 0.985771 |
| Asaccharobacter | PSS.10.score | 0.33005 | -0.10435 | 0.985771 |
| Asaccharobacter | Height.inches | 0.330535 | 0.104498 | 0.985771 |
| Abiotrophia | EDEQ4.Total | 0.330609 | 0.103092 | 0.985771 |
| Anaerofilum | BAI.score | 0.330757 | 0.099809 | 0.985771 |
| Adlercreutzia | PSS.10.score | 0.331403 | -0.10873 | 0.985771 |
| Eubacterium | Mini.IPIP.Extraversion | 0.331461 | -0.10959 | 0.985771 |
| Corynebacterium | EDEQ4.Shape | 0.331496 | 0.101896 | 0.985771 |
| Fusobacterium | BMI | 0.332204 | -0.1031 | 0.985771 |
| Ethanoligenens | EDEQ4.Eating | 0.332611 | 0.105311 | 0.985771 |
| Collinsella | EDEQ4.Eating | 0.332658 | 0.105322 | 0.985771 |
| Coprobacillus | EDEQ4.Weight | 0.33273 | 0.11098 | 0.985771 |
| Anaerococcus | EDEQ4.Restraint | 0.333941 | -0.10285 | 0.985771 |
| Paralactobacillus | BAI.score | 0.334029 | -0.10973 | 0.985771 |
| Leuconostoc | PSS.10.score | 0.334157 | 0.092873 | 0.985771 |
| Lactovum | EDEQ4.Shape | 0.335193 | 0.109952 | 0.985771 |
| Olsenella | EDEQ4.Eating | 0.335275 | 0.10574 | 0.985771 |
| Butyricicoccus | EDEQ4.Shape | 0.335343 | -0.10408 | 0.985771 |
| Bacteroides | EDEQ4.Restraint | 0.33619 | 0.106745 | 0.985771 |
| Granulicatella | EDEQ4.Restraint | 0.336262 | -0.10571 | 0.985771 |
| Parvimonas | EDEQ4.Total | 0.336316 | -0.08899 | 0.985771 |
| Asaccharobacter | Mini.IPIP.Conscientiousness | 0.337159 | 0.109347 | 0.985771 |
| Asaccharobacter | Qualtrics.age | 0.33725 | 0.103322 | 0.985771 |
| Murdochiella | BDI.II.score | 0.337608 | -0.10189 | 0.985771 |
| Gemmiger | Mini.IPIP.Neuroticism | 0.337915 | 0.113389 | 0.985771 |
| Lachnoanaerobaculum | Weight.lbs | 0.338129 | 0.114419 | 0.985771 |
| Syntrophococcus | BDI.II.score | 0.33913 | 0.108733 | 0.985771 |
| Victivallis | Mini.IPIP.Agreeableness | 0.339608 | 0.105578 | 0.985771 |
| Oribacterium | BDI.II.score | 0.339669 | 0.109146 | 0.985771 |
| Oxalobacter | EDEQ4.Restraint | 0.340216 | 0.104192 | 0.985771 |
| Acetanaerobacterium | EDEQ4.Eating | 0.340838 | -0.10905 | 0.985771 |
| Adlercreutzia | Height.inches | 0.341217 | 0.107837 | 0.985771 |
| Eubacterium | BDI.II.score | 0.34204 | -0.10511 | 0.985771 |
| Barnesiella | Mini.IPIP.Extraversion | 0.342543 | 0.107086 | 0.985771 |
| Streptophyta | Mini.IPIP.Conscientiousness | 0.34296 | 0.101626 | 0.985771 |
| Rothia | BMI | 0.34321 | -0.09492 | 0.985771 |
| Akkermansia | EDEQ4.Restraint | 0.343979 | 0.103565 | 0.985771 |
| Gemmiger | EDEQ4.Restraint | 0.343979 | 0.101387 | 0.985771 |
| Flavonifractor | PSS.10.score | 0.346948 | -0.09999 | 0.985771 |
| Bilophila | EDEQ4.Total | 0.347343 | 0.094256 | 0.985771 |
| Clostridium.IV | EDEQ4.Total | 0.347747 | 0.094517 | 0.985771 |
| Anaerotruncus | EDEQ4.Eating | 0.349084 | -0.10343 | 0.985771 |
| Streptophyta | Weight.lbs | 0.350531 | 0.102443 | 0.985771 |
| Asaccharobacter | Mini.IPIP.Agreeableness | 0.351301 | 0.084259 | 0.985771 |
| Eggerthia | Mini.IPIP.Agreeableness | 0.352528 | -0.11539 | 0.985771 |
| Oscillibacter | EDEQ4.Weight | 0.352611 | -0.09033 | 0.985771 |
| Sporobacter | EDEQ4.Weight | 0.352611 | 0.11747 | 0.985771 |
| Flavonifractor | BDI.II.score | 0.353455 | -0.09481 | 0.985771 |
| Haemophilus | Mini.IPIP.Conscientiousness | 0.35396 | -0.09752 | 0.985771 |
| Lachnobacterium | EDEQ4.Eating | 0.354233 | -0.1013 | 0.985771 |
| Atopobium | BAI.score | 0.354855 | -0.09377 | 0.985771 |
| Varibaculum | BMI | 0.355515 | 0.091755 | 0.985771 |
| Paralactobacillus | BMI | 0.355605 | -0.10206 | 0.985771 |
| Pyrolobus | Mini.IPIP.Conscientiousness | 0.35587 | -0.10372 | 0.985771 |
| shannonDiversity (genus) | BAI.score | 0.356459 | 0.094891 | 0.985771 |
| Solobacterium | Qualtrics.age | 0.357535 | 0.104514 | 0.985771 |
| Akkermansia | PSS.10.score | 0.358366 | 0.113038 | 0.985771 |
| Gemmiger | PSS.10.score | 0.358366 | 0.08586 | 0.985771 |
| Sutterella | BDI.II.score | 0.358952 | 0.098094 | 0.985771 |
| Lachnoanaerobaculum | BAI.score | 0.359484 | -0.09914 | 0.985771 |
| Lactovum | BDI.II.score | 0.359567 | 0.092727 | 0.985771 |
| Anaerovorax | EDEQ4.Shape | 0.359978 | 0.109936 | 0.985771 |
| Hespellia | EDEQ4.Total | 0.360012 | 0.091996 | 0.985771 |
| Sporobacter | Weight.lbs | 0.360096 | 0.11177 | 0.985771 |
| Holdemania | Mini.IPIP.Imagination | 0.360294 | -0.09482 | 0.985771 |
| Alistipes | Mini.IPIP.Imagination | 0.360305 | -0.09467 | 0.985771 |
| Dorea | BAI.score | 0.36032 | 0.094754 | 0.985771 |
| Coprobacillus | BDI.II.score | 0.360463 | 0.09493 | 0.985771 |
| Atopobium | EDEQ4.Eating | 0.360594 | -0.09754 | 0.985771 |
| Megamonas | EDEQ4.Restraint | 0.360889 | -0.10098 | 0.985771 |
| Solobacterium | Mini.IPIP.Imagination | 0.361572 | -0.09309 | 0.985771 |
| Catenibacterium | Mini.IPIP.Extraversion | 0.36163 | -0.10546 | 0.985771 |
| Peptoniphilus | BDI.II.score | 0.36271 | -0.09872 | 0.985771 |
| Barnesiella | EDEQ4.Total | 0.362966 | 0.115258 | 0.985771 |
| Sporacetigenium | Mini.IPIP.Neuroticism | 0.36351 | 0.095862 | 0.985771 |
| Clostridium.XI | BMI | 0.364862 | 0.108218 | 0.985771 |
| Candidatus.Carsonella | EDEQ4.Weight | 0.365264 | -0.09241 | 0.985771 |
| Enterorhabdus | EDEQ4.Shape | 0.366647 | 0.094437 | 0.985771 |
| Bacteroides | Mini.IPIP.Agreeableness | 0.367005 | 0.094773 | 0.985771 |
| Acetivibrio | Mini.IPIP.Imagination | 0.367025 | -0.0956 | 0.985771 |
| Parasutterella | PSS.10.score | 0.36792 | 0.096429 | 0.985771 |
| Coprococcus | Height.inches | 0.368833 | 0.09463 | 0.985771 |
| Leuconostoc | EDEQ4.Eating | 0.369766 | 0.094586 | 0.985771 |
| Acetivibrio | Mini.IPIP.Agreeableness | 0.370006 | 0.098163 | 0.985771 |
| Paralactobacillus | Mini.IPIP.Imagination | 0.370651 | -0.09555 | 0.985771 |
| Victivallis | BMI | 0.370885 | 0.096589 | 0.985771 |
| Victivallis | EDEQ4.Total | 0.370967 | 0.091618 | 0.985771 |
| Escherichia.Shigella | EDEQ4.Weight | 0.371621 | 0.094204 | 0.985771 |
| Lactonifactor | EDEQ4.Restraint | 0.372137 | -0.10856 | 0.985771 |
| Enterobacter | EDEQ4.Weight | 0.372798 | 0.09902 | 0.985771 |
| Pseudobutyrivibrio | EDEQ4.Shape | 0.373007 | 0.099985 | 0.985771 |
| Tannerella | BMI | 0.373145 | -0.09391 | 0.985771 |
| Sporobacter | Mini.IPIP.Extraversion | 0.373362 | -0.09225 | 0.985771 |
| Clostridium.sensu.stricto | BMI | 0.374509 | 0.104315 | 0.985771 |
| Gordonibacter | BDI.II.score | 0.374861 | -0.10189 | 0.985771 |
| Clostridium.XVIII | EDEQ4.Eating | 0.374882 | 0.098567 | 0.985771 |
| Ruminococcus2 | EDEQ4.Eating | 0.374882 | 0.09783 | 0.985771 |
| Paraprevotella | EDEQ4.Restraint | 0.375171 | -0.09348 | 0.985771 |
| Holdemania | EDEQ4.Shape | 0.375505 | 0.088141 | 0.985771 |
| Adlercreutzia | BMI | 0.375948 | -0.08178 | 0.985771 |
| Sutterella | EDEQ4.Weight | 0.376117 | 0.093452 | 0.985771 |
| Blautia | BDI.II.score | 0.376941 | -0.09546 | 0.985771 |
| Marvinbryantia | Mini.IPIP.Extraversion | 0.377313 | 0.098835 | 0.985771 |
| Acetitomaculum | EDEQ4.Restraint | 0.377455 | 0.082723 | 0.985771 |
| Blautia | EDEQ4.Shape | 0.377501 | -0.10287 | 0.985771 |
| Lactonifactor | EDEQ4.Shape | 0.377501 | -0.0986 | 0.985771 |
| Odoribacter | Mini.IPIP.Conscientiousness | 0.377745 | -0.09011 | 0.985771 |
| Sporacetigenium | Mini.IPIP.Conscientiousness | 0.37878 | -0.08647 | 0.985771 |
| Finegoldia | BDI.II.score | 0.37936 | -0.09999 | 0.985771 |
| Acetanaerobacterium | PSS.10.score | 0.379895 | -0.09989 | 0.985771 |
| Desulfovibrio | Mini.IPIP.Extraversion | 0.380105 | -0.09436 | 0.985771 |
| Holdemania | EDEQ4.Restraint | 0.380428 | -0.09916 | 0.985771 |
| Veillonella | EDEQ4.Total | 0.380684 | -0.08345 | 0.985771 |
| Weissella | Mini.IPIP.Neuroticism | 0.381336 | 0.088527 | 0.985771 |
| Leuconostoc | BDI.II.score | 0.381809 | 0.091511 | 0.985771 |
| Metascardovia | Mini.IPIP.Extraversion | 0.381837 | -0.09024 | 0.985771 |
| Moryella | Mini.IPIP.Agreeableness | 0.38188 | 0.093648 | 0.985771 |
| Atopobium | Weight.lbs | 0.382212 | 0.090672 | 0.985771 |
| Actinobacillus | BMI | 0.382216 | 0.090502 | 0.985771 |
| Streptococcus | EDEQ4.Eating | 0.383709 | -0.09191 | 0.985771 |
| Anaerostipes | Weight.lbs | 0.383713 | -0.1025 | 0.985771 |
| Olsenella | Qualtrics.age | 0.383902 | -0.12055 | 0.985771 |
| Anaerosporobacter | BAI.score | 0.38402 | -0.1235 | 0.985771 |
| Actinobacillus | EDEQ4.Eating | 0.384068 | 0.096211 | 0.985771 |
| Gallibacterium | Mini.IPIP.Extraversion | 0.384867 | 0.0965 | 0.985771 |
| Papillibacter | BDI.II.score | 0.384928 | -0.10367 | 0.985771 |
| Weissella | BDI.II.score | 0.385136 | 0.088839 | 0.985771 |
| Butyricicoccus | Mini.IPIP.Extraversion | 0.385327 | 0.095534 | 0.985771 |
| Acetanaerobacterium | Mini.IPIP.Neuroticism | 0.386397 | -0.09382 | 0.985771 |
| Lactococcus | Height.inches | 0.386821 | -0.09411 | 0.985771 |
| Marvinbryantia | BMI | 0.388612 | -0.11039 | 0.985771 |
| Catenibacterium | BMI | 0.388624 | -0.08963 | 0.985771 |
| Anaerovorax | Mini.IPIP.Extraversion | 0.388638 | -0.09341 | 0.985771 |
| Gardnerella | EDEQ4.Restraint | 0.38872 | 0.092329 | 0.985771 |
| Akkermansia | EDEQ4.Shape | 0.389543 | 0.085608 | 0.985771 |
| Clostridium.XVIII | Qualtrics.age | 0.389805 | -0.09628 | 0.985771 |
| Haemophilus | EDEQ4.Weight | 0.390174 | -0.09898 | 0.985771 |
| Bilophila | BMI | 0.390249 | 0.09307 | 0.985771 |
| Atopobium | Qualtrics.age | 0.390377 | 0.095885 | 0.985771 |
| Enterorhabdus | BMI | 0.390661 | -0.09471 | 0.985771 |
| Varibaculum | BAI.score | 0.390723 | 0.088138 | 0.985771 |
| Lachnospira | PSS.10.score | 0.391771 | 0.089966 | 0.985771 |
| Parasporobacterium | PSS.10.score | 0.39196 | 0.100079 | 0.985771 |
| Streptophyta | Mini.IPIP.Imagination | 0.392317 | 0.08042 | 0.985771 |
| Paludibacter | Mini.IPIP.Agreeableness | 0.392477 | -0.0953 | 0.985771 |
| Anaerosporobacter | Mini.IPIP.Agreeableness | 0.393074 | 0.086143 | 0.985771 |
| Coprococcus | Mini.IPIP.Extraversion | 0.393433 | 0.073636 | 0.985771 |
| Faecalibacterium | Mini.IPIP.Extraversion | 0.393433 | 0.088935 | 0.985771 |
| Anaerorhabdus | Mini.IPIP.Imagination | 0.39372 | -0.08987 | 0.985771 |
| Catenibacterium | Qualtrics.age | 0.39387 | 0.08921 | 0.985771 |
| Atopobium | EDEQ4.Restraint | 0.393951 | -0.08587 | 0.985771 |
| Blautia | PSS.10.score | 0.394019 | -0.10115 | 0.985771 |
| Pyrolobus | Qualtrics.age | 0.394099 | -0.09422 | 0.985771 |
| Pseudoflavonifractor | Mini.IPIP.Imagination | 0.394168 | -0.09175 | 0.985771 |
| Ethanoligenens | BMI | 0.394656 | -0.08683 | 0.985771 |
| Anaerofilum | Mini.IPIP.Conscientiousness | 0.395118 | 0.095678 | 0.985771 |
| Fusobacterium | Mini.IPIP.Conscientiousness | 0.395375 | -0.09557 | 0.985771 |
| Parabacteroides | Weight.lbs | 0.395868 | 0.094326 | 0.985771 |
| Sporacetigenium | EDEQ4.Shape | 0.39665 | 0.094072 | 0.985771 |
| Flavonifractor | BMI | 0.396749 | -0.08782 | 0.985771 |
| Sporobacter | BMI | 0.396749 | -0.09254 | 0.985771 |
| Olsenella | Mini.IPIP.Agreeableness | 0.397016 | -0.09535 | 0.985771 |
| Candidatus.Carsonella | BAI.score | 0.397251 | -0.09154 | 0.985771 |
| Clostridium.IV | PSS.10.score | 0.398109 | -0.08665 | 0.985771 |
| Oscillibacter | Mini.IPIP.Conscientiousness | 0.398112 | -0.0869 | 0.985771 |
| Parabacteroides | Mini.IPIP.Conscientiousness | 0.398112 | 0.082769 | 0.985771 |
| Paludibacter | BAI.score | 0.398597 | 0.105117 | 0.985771 |
| Peptoniphilus | EDEQ4.Total | 0.399094 | -0.0825 | 0.985771 |
| Paraprevotella | BAI.score | 0.399255 | 0.087763 | 0.985771 |
| Hydrogenoanaerobacterium | Mini.IPIP.Conscientiousness | 0.399257 | 0.095052 | 0.985771 |
| Pseudobutyrivibrio | EDEQ4.Restraint | 0.399482 | -0.08969 | 0.985771 |
| Anaerovorax | Weight.lbs | 0.399637 | 0.095675 | 0.985771 |
| Clostridium.sensu.stricto | Mini.IPIP.Conscientiousness | 0.40004 | -0.10589 | 0.985771 |
| Marvinbryantia | Mini.IPIP.Imagination | 0.400331 | 0.099703 | 0.985771 |
| Akkermansia | Mini.IPIP.Neuroticism | 0.400713 | 0.096661 | 0.985771 |
| Streptococcus | EDEQ4.Shape | 0.401815 | -0.08702 | 0.987415 |
| Methanobrevibacter | BMI | 0.404195 | 0.083314 | 0.98838 |
| Enterorhabdus | Height.inches | 0.404215 | 0.086336 | 0.98838 |
| Blautia | EDEQ4.Weight | 0.40514 | -0.08696 | 0.98838 |
| Bifidobacterium | Height.inches | 0.405356 | 0.101078 | 0.98838 |
| Gemmiger | BDI.II.score | 0.405527 | -0.09207 | 0.98838 |
| Bacteroides | Mini.IPIP.Extraversion | 0.405783 | 0.083735 | 0.98838 |
| Actinomyces | Mini.IPIP.Extraversion | 0.405783 | -0.08459 | 0.98838 |
| Anaerorhabdus | EDEQ4.Shape | 0.405893 | 0.090252 | 0.98838 |
| Butyricicoccus | Qualtrics.age | 0.406217 | -0.09254 | 0.98838 |
| Methanobrevibacter | EDEQ4.Weight | 0.406752 | 0.090931 | 0.98838 |
| Metascardovia | EDEQ4.Weight | 0.40698 | -0.08779 | 0.98838 |
| Clostridium.XlVb | Mini.IPIP.Agreeableness | 0.407582 | 0.104506 | 0.988788 |
| Oscillibacter | Mini.IPIP.Extraversion | 0.409951 | -0.09622 | 0.990264 |
| Actinobacillus | Mini.IPIP.Extraversion | 0.41019 | 0.096799 | 0.990264 |
| Lactococcus | PSS.10.score | 0.410519 | -0.08728 | 0.990264 |
| Acidaminococcus | BDI.II.score | 0.410775 | 0.096556 | 0.990264 |
| Phascolarctobacterium | EDEQ4.Total | 0.411306 | 0.080816 | 0.990264 |
| Lachnospiracea_incertae_sedis | EDEQ4.Total | 0.411306 | -0.09212 | 0.990264 |
| Corynebacterium | EDEQ4.Total | 0.412396 | 0.088797 | 0.990264 |
| Anaerococcus | Mini.IPIP.Conscientiousness | 0.412423 | -0.08761 | 0.990264 |
| Peptostreptococcus | Mini.IPIP.Neuroticism | 0.413183 | -0.09013 | 0.990264 |
| Coprococcus | BMI | 0.413304 | -0.08858 | 0.990264 |
| Clostridium.XlVb | BMI | 0.413304 | 0.092599 | 0.990264 |
| Rothia | EDEQ4.Restraint | 0.414124 | -0.08614 | 0.990264 |
| Prevotella | EDEQ4.Restraint | 0.414754 | 0.086014 | 0.990264 |
| Acetanaerobacterium | Mini.IPIP.Imagination | 0.414952 | -0.09968 | 0.990264 |
| Campylobacter | BAI.score | 0.415288 | 0.09048 | 0.990264 |
| Candidatus.Carsonella | EDEQ4.Shape | 0.415386 | 0.084248 | 0.990264 |
| Anaerosporobacter | EDEQ4.Eating | 0.415581 | -0.08238 | 0.990264 |
| shannonDiversity (genus) | Weight.lbs | 0.416637 | 0.093632 | 0.991744 |
| Sporobacter | BAI.score | 0.417092 | -0.08209 | 0.991791 |
| Asaccharobacter | BMI | 0.419413 | -0.08181 | 0.995114 |
| Hydrogenoanaerobacterium | Weight.lbs | 0.420026 | 0.099355 | 0.995114 |
| Defluviitalea | EDEQ4.Restraint | 0.420836 | 0.084134 | 0.995114 |
| Gordonibacter | PSS.10.score | 0.420969 | -0.07917 | 0.995114 |
| Natronincola | BAI.score | 0.422217 | -0.088 | 0.995114 |
| Parasutterella | BDI.II.score | 0.422295 | -0.08507 | 0.995114 |
| Actinomyces | EDEQ4.Shape | 0.422777 | -0.0898 | 0.995114 |
| Gemella | BDI.II.score | 0.423157 | -0.07808 | 0.995114 |
| Clostridium.XVIII | PSS.10.score | 0.42318 | 0.081186 | 0.995114 |
| Victivallis | Mini.IPIP.Neuroticism | 0.423217 | -0.08633 | 0.995114 |
| Flavonifractor | Mini.IPIP.Conscientiousness | 0.423295 | 0.083429 | 0.995114 |
| Granulicatella | Mini.IPIP.Imagination | 0.423783 | 0.083924 | 0.995235 |
| Saccharibacteria_genera_incertae_sedis | EDEQ4.Weight | 0.425287 | 0.080868 | 0.996205 |
| Tannerella | BDI.II.score | 0.425512 | 0.085904 | 0.996205 |
| Acidaminococcus | Height.inches | 0.426018 | 0.091455 | 0.996205 |
| Clostridium.IV | BDI.II.score | 0.426717 | -0.08595 | 0.996205 |
| Anaerotruncus | EDEQ4.Shape | 0.426959 | -0.09223 | 0.996205 |
| Streptococcus | Qualtrics.age | 0.427301 | -0.09649 | 0.996205 |
| Megasphaera | PSS.10.score | 0.427496 | 0.090982 | 0.996205 |
| Olsenella | Weight.lbs | 0.427695 | 0.082607 | 0.996205 |
| Lactovum | PSS.10.score | 0.428268 | -0.08352 | 0.996522 |
| Tannerella | Mini.IPIP.Imagination | 0.429299 | -0.07961 | 0.996925 |
| Lactovum | Qualtrics.age | 0.430898 | -0.08348 | 0.996925 |
| Anaerosporobacter | Weight.lbs | 0.431539 | -0.0833 | 0.996925 |
| Papillibacter | PSS.10.score | 0.431687 | -0.08235 | 0.996925 |
| Clostridium.XlVb | PSS.10.score | 0.431738 | 0.089302 | 0.996925 |
| Dialister | PSS.10.score | 0.431738 | 0.08293 | 0.996925 |
| Murdochiella | Qualtrics.age | 0.43232 | -0.08462 | 0.996925 |
| Papillibacter | Mini.IPIP.Neuroticism | 0.432367 | -0.08161 | 0.996925 |
| Anaerorhabdus | PSS.10.score | 0.432735 | -0.08434 | 0.996925 |
| Lachnospira | Mini.IPIP.Extraversion | 0.432818 | 0.092739 | 0.996925 |
| Hydrotalea | BMI | 0.433378 | 0.08633 | 0.997207 |
| Coprobacillus | Mini.IPIP.Imagination | 0.43415 | -0.09224 | 0.997975 |
| Escherichia.Shigella | Weight.lbs | 0.435217 | -0.09129 | 0.998902 |
| Desulfovibrio | BDI.II.score | 0.43545 | 0.082689 | 0.998902 |
| Gallibacterium | Mini.IPIP.Neuroticism | 0.435869 | 0.092659 | 0.998902 |
| Escherichia.Shigella | Mini.IPIP.Imagination | 0.437925 | -0.08128 | 0.999113 |
| Barnesiella | PSS.10.score | 0.438172 | -0.07942 | 0.999113 |
| Akkermansia | Mini.IPIP.Imagination | 0.438567 | -0.08138 | 0.999113 |
| Ruminococcus2 | BAI.score | 0.438581 | 0.077454 | 0.999113 |
| Dialister | BDI.II.score | 0.439735 | 0.083793 | 0.999113 |
| shannonDiversity (genus) | PSS.10.score | 0.440396 | 0.084016 | 0.999113 |
| Clostridium.III | EDEQ4.Restraint | 0.440465 | -0.08215 | 0.999113 |
| Clostridium.sensu.stricto | Mini.IPIP.Agreeableness | 0.441762 | -0.08919 | 0.999113 |
| Erysipelotrichaceae_incertae_sedis | Mini.IPIP.Agreeableness | 0.441892 | -0.0838 | 0.999113 |
| Sporobacter | BDI.II.score | 0.444124 | -0.08234 | 0.999113 |
| Campylobacter | EDEQ4.Restraint | 0.445501 | -0.08 | 0.999113 |
| Streptococcus | EDEQ4.Total | 0.445516 | -0.0876 | 0.999113 |
| Varibaculum | EDEQ4.Shape | 0.445914 | 0.087442 | 0.999113 |
| Weissella | Height.inches | 0.447877 | -0.07981 | 0.999113 |
| Parasutterella | Mini.IPIP.Agreeableness | 0.448375 | 0.076675 | 0.999113 |
| Clostridium.XI | BDI.II.score | 0.448539 | 0.085061 | 0.999113 |
| Robinsoniella | Qualtrics.age | 0.44873 | 0.080415 | 0.999113 |
| Alistipes | Mini.IPIP.Conscientiousness | 0.449386 | -0.09233 | 0.999113 |
| Oxalobacter | Qualtrics.age | 0.449489 | 0.080487 | 0.999113 |
| Metascardovia | Qualtrics.age | 0.449972 | 0.082983 | 0.999113 |
| Oxalobacter | Mini.IPIP.Imagination | 0.450347 | -0.0894 | 0.999113 |
| Abiotrophia | PSS.10.score | 0.451957 | 0.077776 | 0.999113 |
| Dialister | BMI | 0.452016 | -0.07097 | 0.999113 |
| Hydrogenoanaerobacterium | BDI.II.score | 0.452344 | -0.08042 | 0.999113 |
| Shuttleworthia | Height.inches | 0.45265 | -0.07971 | 0.999113 |
| Enterorhabdus | Mini.IPIP.Conscientiousness | 0.452656 | 0.080579 | 0.999113 |
| Lachnospiracea_incertae_sedis | PSS.10.score | 0.453567 | 0.079826 | 0.999113 |
| Methanobrevibacter | Mini.IPIP.Conscientiousness | 0.454083 | 0.091816 | 0.999113 |
| Parvimonas | Weight.lbs | 0.454944 | -0.08898 | 0.999113 |
| Oribacterium | Qualtrics.age | 0.455147 | -0.07977 | 0.999113 |
| Paludibacter | EDEQ4.Shape | 0.455609 | -0.0775 | 0.999113 |
| Murdochiella | BAI.score | 0.455823 | -0.08304 | 0.999113 |
| Anaerotruncus | Mini.IPIP.Imagination | 0.456125 | -0.08612 | 0.999113 |
| Butyricimonas | EDEQ4.Shape | 0.456133 | 0.08077 | 0.999113 |
| Butyricicoccus | BMI | 0.45644 | 0.090867 | 0.999113 |
| Sporacetigenium | EDEQ4.Restraint | 0.456841 | 0.083096 | 0.999113 |
| Acetitomaculum | BAI.score | 0.456906 | -0.08791 | 0.999113 |
| Ruminococcus2 | BDI.II.score | 0.457442 | 0.076704 | 0.999113 |
| Rothia | Mini.IPIP.Conscientiousness | 0.457652 | -0.07986 | 0.999113 |
| Robinsoniella | EDEQ4.Total | 0.458505 | 0.074457 | 0.999113 |
| Sutterella | BAI.score | 0.458608 | 0.087743 | 0.999113 |
| Anaerotruncus | BDI.II.score | 0.459601 | -0.08422 | 0.999113 |
| Parvimonas | PSS.10.score | 0.45972 | 0.080647 | 0.999113 |
| Clostridium.XVIII | EDEQ4.Restraint | 0.460149 | -0.07385 | 0.999113 |
| Lachnospiracea_incertae_sedis | EDEQ4.Restraint | 0.460149 | -0.07442 | 0.999113 |
| Enterobacter | Mini.IPIP.Imagination | 0.460504 | -0.08585 | 0.999113 |
| Anaerofilum | BMI | 0.46226 | 0.078687 | 0.999113 |
| Phascolarctobacterium | Qualtrics.age | 0.462329 | 0.072151 | 0.999113 |
| Subdoligranulum | PSS.10.score | 0.46247 | -0.08282 | 0.999113 |
| Streptophyta | Qualtrics.age | 0.462807 | 0.075281 | 0.999113 |
| Catenibacterium | Weight.lbs | 0.464529 | -0.08445 | 0.999113 |
| Megamonas | Mini.IPIP.Agreeableness | 0.46465 | -0.0689 | 0.999113 |
| Abiotrophia | EDEQ4.Shape | 0.465053 | 0.080122 | 0.999113 |
| Lachnospiracea_incertae_sedis | BMI | 0.465361 | -0.05206 | 0.999113 |
| Haemophilus | EDEQ4.Eating | 0.465891 | 0.076477 | 0.999113 |
| Porphyromonas | EDEQ4.Eating | 0.467118 | -0.07939 | 0.999113 |
| Coprobacillus | PSS.10.score | 0.46717 | 0.076923 | 0.999113 |
| Anaerorhabdus | BAI.score | 0.46778 | 0.078822 | 0.999113 |
| Lactococcus | EDEQ4.Eating | 0.468722 | 0.076406 | 0.999113 |
| Tannerella | EDEQ4.Eating | 0.468851 | 0.074727 | 0.999113 |
| Escherichia.Shigella | Mini.IPIP.Conscientiousness | 0.468917 | 0.080693 | 0.999113 |
| Clostridium.XI | Mini.IPIP.Neuroticism | 0.470045 | 0.078001 | 0.999113 |
| Robinsoniella | BDI.II.score | 0.470846 | -0.08096 | 0.999113 |
| Saccharibacteria_genera_incertae_sedis | Mini.IPIP.Agreeableness | 0.470919 | 0.075839 | 0.999113 |
| Tannerella | Height.inches | 0.471049 | 0.073693 | 0.999113 |
| Ruminococcus2 | Mini.IPIP.Conscientiousness | 0.471809 | 0.083182 | 0.999113 |
| Pseudoflavonifractor | EDEQ4.Restraint | 0.471912 | 0.081778 | 0.999113 |
| Atopobium | EDEQ4.Weight | 0.474933 | -0.06386 | 0.999113 |
| Collinsella | EDEQ4.Shape | 0.475622 | 0.073402 | 0.999113 |
| Megasphaera | Mini.IPIP.Conscientiousness | 0.476274 | -0.07525 | 0.999113 |
| Lactonifactor | EDEQ4.Total | 0.476736 | -0.08249 | 0.999113 |
| Granulicatella | BMI | 0.477179 | -0.09075 | 0.999113 |
| Abiotrophia | EDEQ4.Eating | 0.477697 | 0.073247 | 0.999113 |
| Acetivibrio | BDI.II.score | 0.478008 | -0.0852 | 0.999113 |
| Gordonibacter | BAI.score | 0.478742 | -0.08819 | 0.999113 |
| Fusobacterium | Mini.IPIP.Imagination | 0.479383 | -0.07797 | 0.999113 |
| Asaccharobacter | EDEQ4.Weight | 0.480283 | 0.076238 | 0.999113 |
| Desulfovibrio | BAI.score | 0.480909 | 0.07891 | 0.999113 |
| Veillonella | Mini.IPIP.Imagination | 0.481092 | -0.06761 | 0.999113 |
| Acetivibrio | EDEQ4.Total | 0.48139 | 0.075734 | 0.999113 |
| Streptococcus | Weight.lbs | 0.482675 | -0.07901 | 0.999113 |
| Anaerococcus | Mini.IPIP.Agreeableness | 0.482935 | 0.087506 | 0.999113 |
| Paralactobacillus | Weight.lbs | 0.483106 | -0.08567 | 0.999113 |
| Sporacetigenium | Qualtrics.age | 0.484038 | -0.08462 | 0.999113 |
| Natronincola | Mini.IPIP.Conscientiousness | 0.484372 | -0.06632 | 0.999113 |
| Clostridium.sensu.stricto | BDI.II.score | 0.484612 | 0.0753 | 0.999113 |
| Corynebacterium | Mini.IPIP.Extraversion | 0.484739 | 0.075892 | 0.999113 |
| Parasutterella | Weight.lbs | 0.484842 | 0.084392 | 0.999113 |
| Granulicatella | PSS.10.score | 0.485729 | 0.072491 | 0.999113 |
| Tannerella | EDEQ4.Total | 0.486269 | 0.074559 | 0.999113 |
| Odoribacter | Qualtrics.age | 0.48723 | 0.075739 | 0.999113 |
| Tannerella | Mini.IPIP.Neuroticism | 0.487281 | 0.074041 | 0.999113 |
| Pyrolobus | EDEQ4.Weight | 0.487696 | -0.07825 | 0.999113 |
| Clostridium.XVIII | BAI.score | 0.488031 | 0.080322 | 0.999113 |
| Eggerthella | EDEQ4.Total | 0.488167 | -0.07794 | 0.999113 |
| Anaerovorax | PSS.10.score | 0.488514 | 0.071386 | 0.999113 |
| Peptoniphilus | PSS.10.score | 0.489762 | -0.06862 | 0.999113 |
| Phascolarctobacterium | EDEQ4.Weight | 0.489805 | 0.070248 | 0.999113 |
| Streptococcus | Mini.IPIP.Conscientiousness | 0.490182 | 0.077132 | 0.999113 |
| Gordonibacter | Mini.IPIP.Extraversion | 0.49145 | -0.07053 | 0.999113 |
| Bilophila | PSS.10.score | 0.491708 | 0.082202 | 0.999113 |
| Bifidobacterium | Mini.IPIP.Imagination | 0.492657 | 0.076244 | 0.999113 |
| Ruminococcus2 | Mini.IPIP.Neuroticism | 0.492998 | 0.061218 | 0.999113 |
| Tepidibacter | Mini.IPIP.Imagination | 0.493731 | -0.06979 | 0.999113 |
| Actinomyces | Height.inches | 0.49387 | -0.06458 | 0.999113 |
| Ruminococcus2 | EDEQ4.Shape | 0.49401 | 0.08379 | 0.999113 |
| Gordonibacter | Weight.lbs | 0.494128 | 0.070167 | 0.999113 |
| Lachnospira | Mini.IPIP.Imagination | 0.494986 | -0.07403 | 0.999113 |
| Peptostreptococcus | PSS.10.score | 0.49533 | 0.072193 | 0.999113 |
| Butyricimonas | Mini.IPIP.Neuroticism | 0.496249 | 0.069279 | 0.999113 |
| Acidaminococcus | Mini.IPIP.Conscientiousness | 0.496685 | 0.073893 | 0.999113 |
| Paralactobacillus | EDEQ4.Eating | 0.496802 | 0.074345 | 0.999113 |
| Porphyromonas | BAI.score | 0.497248 | -0.07462 | 0.999113 |
| Leuconostoc | EDEQ4.Total | 0.497355 | 0.082653 | 0.999113 |
| Syntrophococcus | EDEQ4.Shape | 0.497368 | -0.06964 | 0.999113 |
| Pyrolobus | EDEQ4.Eating | 0.498486 | 0.076054 | 0.999113 |
| Murdochiella | Mini.IPIP.Agreeableness | 0.498487 | 0.072028 | 0.999113 |
| Actinomyces | EDEQ4.Weight | 0.49924 | 0.079945 | 0.999113 |
| Hydrogenoanaerobacterium | Mini.IPIP.Neuroticism | 0.499625 | -0.06401 | 0.999113 |
| Streptococcus | Mini.IPIP.Agreeableness | 0.501026 | -0.07711 | 0.999113 |
| Paralactobacillus | Height.inches | 0.501524 | -0.07468 | 0.999113 |
| Papillibacter | EDEQ4.Weight | 0.501568 | -0.05929 | 0.999113 |
| Clostridium.XlVa | Mini.IPIP.Imagination | 0.502008 | 0.060466 | 0.999113 |
| Saccharibacteria_genera_incertae_sedis | EDEQ4.Restraint | 0.50241 | 0.078511 | 0.999113 |
| Escherichia.Shigella | EDEQ4.Shape | 0.502742 | 0.084421 | 0.999113 |
| Dorea | Qualtrics.age | 0.503581 | -0.07425 | 0.999113 |
| Barnesiella | EDEQ4.Weight | 0.503948 | 0.084791 | 0.999113 |
| Leuconostoc | EDEQ4.Weight | 0.504146 | 0.077985 | 0.999113 |
| Gardnerella | BMI | 0.504211 | -0.06223 | 0.999113 |
| Paralactobacillus | BDI.II.score | 0.504325 | 0.072361 | 0.999113 |
| Anaerofustis | BAI.score | 0.504446 | -0.08375 | 0.999113 |
| Gallibacterium | EDEQ4.Total | 0.504569 | 0.075493 | 0.999113 |
| Eggerthia | BAI.score | 0.505568 | -0.06948 | 0.999113 |
| Sporacetigenium | EDEQ4.Eating | 0.506117 | 0.080008 | 0.999113 |
| Acetitomaculum | BMI | 0.506363 | 0.076722 | 0.999113 |
| Ethanoligenens | Mini.IPIP.Conscientiousness | 0.508888 | 0.081383 | 0.999113 |
| Lactobacillus | EDEQ4.Eating | 0.509388 | 0.072789 | 0.999113 |
| Paraprevotella | Mini.IPIP.Conscientiousness | 0.509496 | 0.077156 | 0.999113 |
| Gallibacterium | EDEQ4.Eating | 0.510529 | 0.076112 | 0.999113 |
| Parasutterella | EDEQ4.Restraint | 0.510538 | 0.067179 | 0.999113 |
| Paraprevotella | EDEQ4.Eating | 0.511067 | -0.0688 | 0.999113 |
| Lactococcus | EDEQ4.Weight | 0.511158 | -0.06989 | 0.999113 |
| Roseburia | BMI | 0.511389 | 0.075549 | 0.999113 |
| Acetivibrio | Qualtrics.age | 0.511758 | 0.072304 | 0.999113 |
| Lactovum | EDEQ4.Restraint | 0.512371 | 0.069819 | 0.999113 |
| Eggerthia | Mini.IPIP.Imagination | 0.512757 | 0.081851 | 0.999113 |
| Lachnospiracea_incertae_sedis | EDEQ4.Weight | 0.51357 | -0.08423 | 0.999113 |
| Collinsella | Mini.IPIP.Conscientiousness | 0.513679 | 0.0765 | 0.999113 |
| Parvimonas | EDEQ4.Shape | 0.513976 | -0.05975 | 0.999113 |
| Saccharibacteria_genera_incertae_sedis | Qualtrics.age | 0.514201 | -0.07794 | 0.999113 |
| Anaerococcus | EDEQ4.Weight | 0.514399 | -0.07097 | 0.999113 |
| Methanobrevibacter | BAI.score | 0.515323 | 0.066313 | 0.999113 |
| Enterobacter | EDEQ4.Restraint | 0.515572 | 0.072121 | 0.999113 |
| Gemmiger | BMI | 0.51612 | -0.06879 | 0.999113 |
| Anaerovorax | BAI.score | 0.51617 | -0.06559 | 0.999113 |
| Actinomyces | BAI.score | 0.516221 | 0.080194 | 0.999113 |
| Paralactobacillus | Mini.IPIP.Neuroticism | 0.516283 | -0.06699 | 0.999113 |
| Parasutterella | Mini.IPIP.Neuroticism | 0.516418 | -0.07297 | 0.999113 |
| Weissella | Mini.IPIP.Conscientiousness | 0.517132 | -0.06869 | 0.999113 |
| Sporobacterium | BAI.score | 0.517497 | -0.07353 | 0.999113 |
| Paralactobacillus | PSS.10.score | 0.517568 | -0.07016 | 0.999113 |
| Collinsella | EDEQ4.Total | 0.518577 | 0.063714 | 0.999113 |
| Acetivibrio | BMI | 0.51982 | -0.0626 | 0.999113 |
| Collinsella | Weight.lbs | 0.520094 | 0.063146 | 0.999113 |
| Coprobacillus | EDEQ4.Shape | 0.520665 | 0.056299 | 0.999113 |
| Campylobacter | PSS.10.score | 0.520687 | 0.070238 | 0.999113 |
| Oxalobacter | Weight.lbs | 0.520956 | 0.067073 | 0.999113 |
| Clostridium.III | BDI.II.score | 0.521308 | -0.07352 | 0.999113 |
| Coprococcus | Mini.IPIP.Neuroticism | 0.521316 | -0.07238 | 0.999113 |
| Murdochiella | EDEQ4.Restraint | 0.521865 | -0.07271 | 0.999113 |
| Bilophila | EDEQ4.Eating | 0.522459 | -0.07357 | 0.999113 |
| Dorea | BDI.II.score | 0.522462 | -0.06793 | 0.999113 |
| Tepidibacter | Mini.IPIP.Agreeableness | 0.52247 | -0.06377 | 0.999113 |
| Clostridium.IV | Qualtrics.age | 0.52252 | 0.06501 | 0.999113 |
| Megamonas | EDEQ4.Eating | 0.522902 | -0.06625 | 0.999113 |
| Anaerostipes | EDEQ4.Weight | 0.523242 | -0.07742 | 0.999113 |
| Pseudoflavonifractor | BMI | 0.523254 | -0.07261 | 0.999113 |
| Hydrotalea | Mini.IPIP.Agreeableness | 0.523269 | 0.079715 | 0.999113 |
| Peptoniphilus | Mini.IPIP.Agreeableness | 0.523269 | 0.058533 | 0.999113 |
| Oscillibacter | EDEQ4.Total | 0.52334 | -0.07754 | 0.999113 |
| Fusobacterium | Mini.IPIP.Extraversion | 0.524383 | 0.075549 | 0.999113 |
| Lactobacillus | PSS.10.score | 0.524791 | -0.08115 | 0.999113 |
| Pseudoflavonifractor | EDEQ4.Weight | 0.525672 | -0.05871 | 0.999113 |
| Clostridium.IV | BAI.score | 0.525802 | -0.07145 | 0.999113 |
| Acetitomaculum | Mini.IPIP.Neuroticism | 0.526473 | -0.07188 | 0.999113 |
| shannonDiversity (genus) | Mini.IPIP.Extraversion | 0.52693 | -0.05899 | 0.999113 |
| Olsenella | Mini.IPIP.Conscientiousness | 0.527397 | -0.07234 | 0.999113 |
| Anaerorhabdus | Height.inches | 0.527602 | -0.06997 | 0.999113 |
| Enterorhabdus | Mini.IPIP.Imagination | 0.528191 | -0.06805 | 0.999113 |
| Anaerococcus | EDEQ4.Shape | 0.528365 | 0.069655 | 0.999113 |
| Clostridium.sensu.stricto | EDEQ4.Shape | 0.529368 | 0.066329 | 0.999113 |
| Lachnobacterium | BAI.score | 0.529512 | 0.071378 | 0.999113 |
| Actinomyces | Mini.IPIP.Imagination | 0.530613 | -0.05734 | 0.999113 |
| Weissella | EDEQ4.Shape | 0.530891 | 0.072463 | 0.999113 |
| Sarcina | BDI.II.score | 0.532018 | 0.071466 | 0.999113 |
| Subdoligranulum | BDI.II.score | 0.532124 | -0.07429 | 0.999113 |
| Clostridium.XI | Mini.IPIP.Conscientiousness | 0.53289 | -0.06414 | 0.999113 |
| Candidatus.Carsonella | Mini.IPIP.Extraversion | 0.533255 | -0.06416 | 0.999113 |
| Campylobacter | Mini.IPIP.Imagination | 0.533315 | -0.06838 | 0.999113 |
| Bilophila | Mini.IPIP.Extraversion | 0.533825 | -0.0782 | 0.999113 |
| Moryella | Height.inches | 0.533881 | -0.07209 | 0.999113 |
| Atopobium | BDI.II.score | 0.533996 | 0.056015 | 0.999113 |
| Anaerorhabdus | Mini.IPIP.Neuroticism | 0.534008 | -0.06712 | 0.999113 |
| Sarcina | Mini.IPIP.Extraversion | 0.534012 | -0.0661 | 0.999113 |
| Clostridium.IV | EDEQ4.Shape | 0.536736 | 0.059789 | 0.999113 |
| Lachnospiracea_incertae_sedis | Height.inches | 0.53684 | 0.062094 | 0.999113 |
| Oribacterium | Mini.IPIP.Conscientiousness | 0.537308 | -0.0699 | 0.999113 |
| Oribacterium | EDEQ4.Weight | 0.537487 | -0.0771 | 0.999113 |
| Coprobacillus | Mini.IPIP.Extraversion | 0.537529 | -0.07652 | 0.999113 |
| Oxalobacter | EDEQ4.Total | 0.5378 | 0.061972 | 0.999113 |
| Campylobacter | BDI.II.score | 0.538166 | 0.064769 | 0.999113 |
| Bifidobacterium | EDEQ4.Restraint | 0.538224 | 0.063724 | 0.999113 |
| Oxalobacter | BAI.score | 0.538325 | -0.06405 | 0.999113 |
| Parasutterella | EDEQ4.Eating | 0.538683 | 0.066415 | 0.999113 |
| Escherichia.Shigella | Mini.IPIP.Agreeableness | 0.538833 | 0.066293 | 0.999113 |
| Megasphaera | Mini.IPIP.Extraversion | 0.539302 | 0.059657 | 0.999113 |
| Holdemania | Mini.IPIP.Conscientiousness | 0.540177 | 0.0742 | 0.999113 |
| Lactobacillus | BDI.II.score | 0.541633 | 0.065748 | 0.999113 |
| Eubacterium | EDEQ4.Total | 0.542592 | -0.0566 | 0.999113 |
| Clostridium.XVIII | Mini.IPIP.Conscientiousness | 0.54263 | -0.07207 | 0.999113 |
| Natronincola | BDI.II.score | 0.543238 | 0.073227 | 0.999113 |
| Coprobacillus | EDEQ4.Total | 0.543587 | 0.069113 | 0.999113 |
| Bilophila | Weight.lbs | 0.543885 | 0.072123 | 0.999113 |
| Pyrolobus | BMI | 0.544175 | -0.06657 | 0.999113 |
| Gordonibacter | Qualtrics.age | 0.544181 | 0.060271 | 0.999113 |
| Mogibacterium | Mini.IPIP.Conscientiousness | 0.545005 | 0.071745 | 0.999113 |
| Dialister | Mini.IPIP.Imagination | 0.54522 | -0.05249 | 0.999113 |
| Pseudobutyrivibrio | Weight.lbs | 0.545861 | 0.071435 | 0.999113 |
| Parasutterella | Height.inches | 0.546523 | -0.06824 | 0.999113 |
| Haemophilus | EDEQ4.Total | 0.547154 | -0.06101 | 0.999113 |
| Finegoldia | BAI.score | 0.547781 | -0.06492 | 0.999113 |
| Papillibacter | EDEQ4.Restraint | 0.548389 | 0.070768 | 0.999113 |
| Oscillibacter | EDEQ4.Restraint | 0.54843 | 0.05555 | 0.999113 |
| Ruminococcus2 | Weight.lbs | 0.54911 | -0.07083 | 0.999113 |
| Hydrogenoanaerobacterium | Mini.IPIP.Agreeableness | 0.549162 | -0.06313 | 0.999113 |
| Ethanoligenens | BAI.score | 0.550103 | -0.06488 | 0.999113 |
| Odoribacter | EDEQ4.Restraint | 0.550927 | -0.0702 | 0.999113 |
| Papillibacter | Mini.IPIP.Extraversion | 0.551189 | -0.04975 | 0.999113 |
| Phascolarctobacterium | Mini.IPIP.Extraversion | 0.551232 | 0.073535 | 0.999113 |
| Clostridium.XI | Height.inches | 0.55157 | 0.060744 | 0.999113 |
| Paludibacter | EDEQ4.Restraint | 0.552139 | 0.064301 | 0.999113 |
| Parvimonas | Mini.IPIP.Imagination | 0.552672 | -0.05673 | 0.999113 |
| Acetitomaculum | Mini.IPIP.Agreeableness | 0.552865 | 0.067786 | 0.999113 |
| Candidatus.Carsonella | Mini.IPIP.Agreeableness | 0.553287 | -0.06093 | 0.999113 |
| Varibaculum | Mini.IPIP.Agreeableness | 0.553674 | 0.064718 | 0.999113 |
| Butyricimonas | BAI.score | 0.553782 | 0.076675 | 0.999113 |
| Ethanoligenens | EDEQ4.Shape | 0.553796 | -0.0626 | 0.999113 |
| Shuttleworthia | BAI.score | 0.553919 | -0.06569 | 0.999113 |
| Gemmiger | Weight.lbs | 0.554022 | -0.06288 | 0.999113 |
| Clostridium.XlVa | BMI | 0.554761 | -0.06587 | 0.999113 |
| Lactococcus | EDEQ4.Total | 0.55483 | -0.07608 | 0.999113 |
| Ruminococcus | BAI.score | 0.55508 | 0.071837 | 0.999113 |
| Acidaminococcus | Qualtrics.age | 0.555486 | 0.05592 | 0.999113 |
| Natronincola | PSS.10.score | 0.55557 | 0.057359 | 0.999113 |
| Actinobacillus | Weight.lbs | 0.555888 | 0.063332 | 0.999113 |
| Adlercreutzia | Mini.IPIP.Agreeableness | 0.556087 | 0.05348 | 0.999113 |
| Paludibacter | Mini.IPIP.Neuroticism | 0.556419 | -0.06908 | 0.999113 |
| Roseburia | Qualtrics.age | 0.556523 | -0.0672 | 0.999113 |
| Olsenella | EDEQ4.Total | 0.556814 | 0.054548 | 0.999113 |
| Adlercreutzia | EDEQ4.Weight | 0.557267 | 0.062835 | 0.999113 |
| Oribacterium | Mini.IPIP.Neuroticism | 0.557426 | -0.07861 | 0.999113 |
| Candidatus.Carsonella | EDEQ4.Restraint | 0.55788 | -0.05745 | 0.999113 |
| Ethanoligenens | Weight.lbs | 0.558913 | -0.06416 | 0.999113 |
| Eggerthella | Qualtrics.age | 0.55899 | -0.06585 | 0.999113 |
| Granulicatella | EDEQ4.Eating | 0.559026 | 0.063768 | 0.999113 |
| Murdochiella | EDEQ4.Weight | 0.55942 | -0.05871 | 0.999113 |
| Hespellia | Mini.IPIP.Neuroticism | 0.559466 | -0.06461 | 0.999113 |
| Odoribacter | EDEQ4.Eating | 0.560448 | -0.06516 | 0.999113 |
| Erysipelotrichaceae_incertae_sedis | BDI.II.score | 0.561641 | 0.063463 | 0.999113 |
| Mogibacterium | BMI | 0.562087 | -0.08531 | 0.999113 |
| Gemella | Mini.IPIP.Neuroticism | 0.562995 | -0.05873 | 0.999113 |
| Hallella | BDI.II.score | 0.563478 | -0.06465 | 0.999113 |
| Odoribacter | EDEQ4.Total | 0.564642 | -0.06444 | 0.999113 |
| Ruminococcus | Mini.IPIP.Extraversion | 0.566074 | -0.07654 | 0.999113 |
| Erysipelotrichaceae_incertae_sedis | EDEQ4.Shape | 0.566217 | 0.060648 | 0.999113 |
| Lachnobacterium | Mini.IPIP.Conscientiousness | 0.566313 | 0.057425 | 0.999113 |
| Paludibacter | EDEQ4.Eating | 0.566331 | -0.06408 | 0.999113 |
| Actinobacillus | EDEQ4.Weight | 0.566357 | -0.06662 | 0.999113 |
| Peptoniphilus | Mini.IPIP.Neuroticism | 0.567075 | -0.06368 | 0.999113 |
| Coprococcus | Mini.IPIP.Conscientiousness | 0.567362 | -0.05009 | 0.999113 |
| Campylobacter | EDEQ4.Eating | 0.567621 | 0.062202 | 0.999113 |
| Sarcina | Mini.IPIP.Conscientiousness | 0.56769 | -0.06782 | 0.999113 |
| Gemella | BAI.score | 0.567751 | 0.061668 | 0.999113 |
| Defluviitalea | EDEQ4.Weight | 0.568773 | -0.06023 | 0.999113 |
| Gemella | EDEQ4.Shape | 0.568936 | -0.05642 | 0.999113 |
| Megasphaera | EDEQ4.Total | 0.569908 | 0.059009 | 0.999113 |
| Lachnospiracea_incertae_sedis | BAI.score | 0.570012 | 0.061492 | 0.999113 |
| Erysipelotrichaceae_incertae_sedis | Mini.IPIP.Neuroticism | 0.570304 | 0.069248 | 0.999113 |
| Acetitomaculum | BDI.II.score | 0.570839 | 0.05163 | 0.999113 |
| Lactococcus | Mini.IPIP.Extraversion | 0.571054 | 0.047809 | 0.999113 |
| Fusobacterium | EDEQ4.Restraint | 0.571061 | -0.06309 | 0.999113 |
| Defluviitalea | Mini.IPIP.Neuroticism | 0.571124 | -0.05656 | 0.999113 |
| Megamonas | Mini.IPIP.Extraversion | 0.573733 | -0.05604 | 0.999113 |
| Actinomyces | Weight.lbs | 0.573884 | -0.065 | 0.999113 |
| Roseburia | Weight.lbs | 0.573884 | -0.05232 | 0.999113 |
| Streptophyta | EDEQ4.Weight | 0.574019 | -0.06534 | 0.999113 |
| Saccharibacteria_genera_incertae_sedis | Mini.IPIP.Extraversion | 0.574259 | 0.059673 | 0.999113 |
| Barnesiella | EDEQ4.Eating | 0.574266 | 0.068171 | 0.999113 |
| Abiotrophia | Mini.IPIP.Imagination | 0.574319 | -0.06787 | 0.999113 |
| Marvinbryantia | Mini.IPIP.Conscientiousness | 0.574876 | -0.04796 | 0.999113 |
| Olsenella | Mini.IPIP.Extraversion | 0.575117 | -0.06331 | 0.999113 |
| Clostridium.XlVb | Mini.IPIP.Neuroticism | 0.575321 | -0.05934 | 0.999113 |
| Gemella | Mini.IPIP.Imagination | 0.575358 | -0.06145 | 0.999113 |
| Sutterella | EDEQ4.Eating | 0.575363 | 0.056886 | 0.999113 |
| Corynebacterium | Mini.IPIP.Conscientiousness | 0.57655 | -0.05879 | 0.999113 |
| Gardnerella | EDEQ4.Total | 0.579573 | 0.054465 | 0.999113 |
| Anaerosporobacter | EDEQ4.Total | 0.579676 | 0.058181 | 0.999113 |
| Victivallis | Height.inches | 0.579918 | 0.067899 | 0.999113 |
| Sutterella | BMI | 0.580349 | 0.055636 | 0.999113 |
| Anaerostipes | Height.inches | 0.581612 | -0.05534 | 0.999113 |
| Porphyromonas | Mini.IPIP.Conscientiousness | 0.582125 | -0.05835 | 0.999113 |
| Moryella | BMI | 0.582411 | -0.06173 | 0.999113 |
| Escherichia.Shigella | Mini.IPIP.Extraversion | 0.583097 | 0.056688 | 0.999113 |
| Veillonella | Height.inches | 0.584141 | 0.058568 | 0.999113 |
| Lachnoanaerobaculum | Mini.IPIP.Agreeableness | 0.584158 | 0.053988 | 0.999113 |
| Clostridium.sensu.stricto | EDEQ4.Total | 0.584605 | 0.05845 | 0.999113 |
| Murdochiella | BMI | 0.585089 | 0.053078 | 0.999113 |
| Sporobacterium | Mini.IPIP.Extraversion | 0.586091 | -0.05695 | 0.999113 |
| Paludibacter | BDI.II.score | 0.586321 | -0.05399 | 0.999113 |
| Eubacterium | Weight.lbs | 0.586446 | -0.05244 | 0.999113 |
| Hespellia | Mini.IPIP.Conscientiousness | 0.586703 | -0.0588 | 0.999113 |
| Granulicatella | EDEQ4.Weight | 0.586852 | -0.05835 | 0.999113 |
| Acetanaerobacterium | EDEQ4.Shape | 0.588818 | -0.05865 | 0.999113 |
| Rothia | Mini.IPIP.Agreeableness | 0.588829 | -0.06275 | 0.999113 |
| Hydrogenoanaerobacterium | Mini.IPIP.Extraversion | 0.588859 | -0.06294 | 0.999113 |
| Desulfovibrio | BMI | 0.589839 | 0.044416 | 0.999113 |
| Haemophilus | EDEQ4.Restraint | 0.589891 | -0.05845 | 0.999113 |
| Flavonifractor | Mini.IPIP.Extraversion | 0.591227 | -0.05984 | 0.999113 |
| Gordonibacter | Mini.IPIP.Agreeableness | 0.591834 | 0.06235 | 0.999113 |
| Coprococcus | EDEQ4.Total | 0.5923 | 0.052154 | 0.999113 |
| Clostridium.IV | Mini.IPIP.Conscientiousness | 0.592621 | 0.066289 | 0.999113 |
| Abiotrophia | Weight.lbs | 0.593503 | -0.07354 | 0.999113 |
| Anaerofilum | BDI.II.score | 0.593736 | -0.05823 | 0.999113 |
| Bifidobacterium | Weight.lbs | 0.594077 | 0.084672 | 0.999113 |
| Varibaculum | EDEQ4.Restraint | 0.594094 | -0.0566 | 0.999113 |
| Robinsoniella | EDEQ4.Eating | 0.594746 | -0.05816 | 0.999113 |
| Fusobacterium | BDI.II.score | 0.594978 | -0.06215 | 0.999113 |
| Defluviitalea | EDEQ4.Shape | 0.594981 | -0.05713 | 0.999113 |
| Clostridium.IV | EDEQ4.Restraint | 0.595509 | 0.062549 | 0.999113 |
| Lachnospiracea_incertae_sedis | Mini.IPIP.Neuroticism | 0.595594 | 0.074924 | 0.999113 |
| Metascardovia | Weight.lbs | 0.595832 | 0.063608 | 0.999113 |
| Slackia | Mini.IPIP.Conscientiousness | 0.595875 | -0.0511 | 0.999113 |
| shannonDiversity (genus) | EDEQ4.Eating | 0.596693 | 0.058028 | 0.999113 |
| Collinsella | BDI.II.score | 0.597049 | 0.061791 | 0.999113 |
| Gardnerella | BAI.score | 0.597769 | -0.05263 | 0.999113 |
| Lactococcus | Mini.IPIP.Imagination | 0.597854 | -0.05785 | 0.999113 |
| Ethanoligenens | EDEQ4.Weight | 0.598597 | -0.05094 | 0.999113 |
| Pyrolobus | Height.inches | 0.598679 | 0.055383 | 0.999113 |
| Tepidibacter | PSS.10.score | 0.599464 | -0.05727 | 0.999113 |
| Rothia | BAI.score | 0.599926 | 0.050489 | 0.999113 |
| Slackia | Qualtrics.age | 0.599994 | -0.06408 | 0.999113 |
| Shuttleworthia | Mini.IPIP.Agreeableness | 0.600499 | 0.05674 | 0.999113 |
| Abiotrophia | Mini.IPIP.Extraversion | 0.600924 | -0.05615 | 0.999113 |
| Hallella | Mini.IPIP.Imagination | 0.600996 | -0.06297 | 0.999113 |
| Lachnospiracea_incertae_sedis | Mini.IPIP.Extraversion | 0.60143 | -0.06073 | 0.999113 |
| Haemophilus | Height.inches | 0.60176 | 0.051671 | 0.999113 |
| Atopobium | Mini.IPIP.Agreeableness | 0.602088 | -0.0566 | 0.999113 |
| Moryella | Qualtrics.age | 0.602478 | 0.058449 | 0.999113 |
| Lactovum | BAI.score | 0.603768 | 0.054256 | 0.999113 |
| Peptostreptococcus | Mini.IPIP.Conscientiousness | 0.604729 | 0.052672 | 0.999113 |
| Marvinbryantia | EDEQ4.Total | 0.605031 | 0.061737 | 0.999113 |
| Clostridium.III | EDEQ4.Total | 0.605061 | -0.0538 | 0.999113 |
| Anaerofustis | EDEQ4.Weight | 0.605139 | 0.053973 | 0.999113 |
| Hespellia | EDEQ4.Weight | 0.605651 | -0.05111 | 0.999113 |
| Corynebacterium | EDEQ4.Weight | 0.606112 | 0.053373 | 0.999113 |
| Anaerostipes | BDI.II.score | 0.607349 | -0.0738 | 0.999113 |
| Bifidobacterium | Mini.IPIP.Conscientiousness | 0.60802 | 0.053713 | 0.999113 |
| Oribacterium | Height.inches | 0.609419 | 0.055858 | 0.999113 |
| Marvinbryantia | Weight.lbs | 0.609423 | 0.049118 | 0.999113 |
| Atopobium | Mini.IPIP.Neuroticism | 0.610984 | 0.054681 | 0.999113 |
| Parasporobacterium | Mini.IPIP.Imagination | 0.611926 | -0.0591 | 0.999113 |
| Alistipes | Qualtrics.age | 0.612041 | 0.048664 | 0.999113 |
| Dialister | Height.inches | 0.612399 | 0.052376 | 0.999113 |
| Pseudoflavonifractor | Mini.IPIP.Agreeableness | 0.612499 | 0.052492 | 0.999113 |
| Bacteroides | BDI.II.score | 0.612528 | 0.059137 | 0.999113 |
| Anaerotruncus | EDEQ4.Total | 0.612675 | -0.06093 | 0.999113 |
| Turicibacter | Mini.IPIP.Imagination | 0.613279 | -0.0694 | 0.999113 |
| Anaerorhabdus | EDEQ4.Weight | 0.61328 | 0.057156 | 0.999113 |
| Papillibacter | BAI.score | 0.61329 | -0.06263 | 0.999113 |
| Methanobrevibacter | Mini.IPIP.Agreeableness | 0.613514 | 0.055685 | 0.999113 |
| Paraprevotella | BMI | 0.614905 | -0.05713 | 0.999113 |
| Butyricicoccus | Mini.IPIP.Agreeableness | 0.615099 | -0.05411 | 0.999113 |
| Clostridium.XlVa | Mini.IPIP.Agreeableness | 0.615099 | 0.058493 | 0.999113 |
| Acetivibrio | BAI.score | 0.615384 | -0.05482 | 0.999113 |
| Collinsella | BAI.score | 0.615921 | 0.052522 | 0.999113 |
| Gemmiger | BAI.score | 0.615921 | 0.039501 | 0.999113 |
| Parabacteroides | BAI.score | 0.615921 | 0.060585 | 0.999113 |
| Eubacterium | Qualtrics.age | 0.617188 | -0.05926 | 0.999113 |
| Lachnoanaerobaculum | PSS.10.score | 0.617618 | 0.057107 | 0.999113 |
| Asaccharobacter | Mini.IPIP.Neuroticism | 0.618634 | -0.05755 | 0.999113 |
| Megasphaera | Qualtrics.age | 0.619131 | -0.05732 | 0.999113 |
| Abiotrophia | BMI | 0.620044 | 0.062424 | 0.999113 |
| Subdoligranulum | Mini.IPIP.Agreeableness | 0.620307 | -0.05287 | 0.999113 |
| Turicibacter | Mini.IPIP.Conscientiousness | 0.620949 | -0.05792 | 0.999113 |
| Leuconostoc | Qualtrics.age | 0.621175 | 0.054055 | 0.999113 |
| Gardnerella | EDEQ4.Weight | 0.62208 | 0.051202 | 0.999113 |
| Clostridium.XVIII | EDEQ4.Shape | 0.6222 | -0.04554 | 0.999113 |
| Eubacterium | EDEQ4.Eating | 0.622329 | -0.05978 | 0.999113 |
| Gardnerella | PSS.10.score | 0.622401 | -0.05095 | 0.999113 |
| Gardnerella | Qualtrics.age | 0.627496 | -0.05826 | 0.999113 |
| Clostridium.III | Mini.IPIP.Agreeableness | 0.627663 | -0.04995 | 0.999113 |
| Erysipelotrichaceae_incertae_sedis | EDEQ4.Restraint | 0.627884 | -0.0587 | 0.999113 |
| Mogibacterium | EDEQ4.Eating | 0.628054 | -0.06249 | 0.999113 |
| Faecalibacterium | EDEQ4.Total | 0.628275 | 0.054689 | 0.999113 |
| Enterobacter | Weight.lbs | 0.628361 | 0.05359 | 0.999113 |
| Dorea | Mini.IPIP.Conscientiousness | 0.628824 | 0.044098 | 0.999113 |
| Desulfovibrio | Qualtrics.age | 0.629003 | -0.05461 | 0.999113 |
| Anaerorhabdus | EDEQ4.Eating | 0.629041 | 0.059239 | 0.999113 |
| Natronincola | EDEQ4.Weight | 0.629196 | -0.05711 | 0.999113 |
| Megasphaera | Mini.IPIP.Neuroticism | 0.629533 | 0.053401 | 0.999113 |
| Murdochiella | EDEQ4.Total | 0.630832 | -0.05385 | 0.999113 |
| Lactonifactor | EDEQ4.Eating | 0.631002 | -0.05255 | 0.999113 |
| Bilophila | Mini.IPIP.Conscientiousness | 0.631166 | -0.05119 | 0.999113 |
| Methanobrevibacter | Mini.IPIP.Extraversion | 0.631189 | 0.044759 | 0.999113 |
| Catenibacterium | BAI.score | 0.631577 | 0.06021 | 0.999113 |
| Weissella | EDEQ4.Total | 0.63238 | 0.052275 | 0.999113 |
| Gemella | EDEQ4.Eating | 0.632702 | -0.05329 | 0.999113 |
| Anaerovorax | Mini.IPIP.Agreeableness | 0.633035 | 0.053129 | 0.999113 |
| Lachnobacterium | Mini.IPIP.Imagination | 0.633273 | 0.048072 | 0.999113 |
| Streptococcus | EDEQ4.Weight | 0.635428 | -0.05528 | 0.999113 |
| Anaerotruncus | EDEQ4.Restraint | 0.636035 | -0.05315 | 0.999113 |
| Solobacterium | Mini.IPIP.Neuroticism | 0.636615 | 0.05279 | 0.999113 |
| Blautia | BAI.score | 0.636833 | -0.0646 | 0.999113 |
| Hydrotalea | EDEQ4.Restraint | 0.637254 | 0.055646 | 0.999113 |
| Streptophyta | Mini.IPIP.Agreeableness | 0.637375 | -0.04236 | 0.999113 |
| Collinsella | Qualtrics.age | 0.638061 | 0.053285 | 0.999113 |
| Hespellia | EDEQ4.Eating | 0.639 | -0.05361 | 0.999113 |
| Escherichia.Shigella | EDEQ4.Eating | 0.639262 | 0.05092 | 0.999113 |
| Metascardovia | EDEQ4.Total | 0.639487 | -0.05064 | 0.999113 |
| Tepidibacter | BDI.II.score | 0.639734 | 0.051132 | 0.999113 |
| Pseudobutyrivibrio | BAI.score | 0.641072 | -0.05656 | 0.999113 |
| Tepidibacter | Weight.lbs | 0.641589 | -0.04987 | 0.999113 |
| Moryella | EDEQ4.Total | 0.64283 | 0.046402 | 0.999113 |
| Anaerorhabdus | Qualtrics.age | 0.64322 | -0.05214 | 0.999113 |
| Porphyromonas | EDEQ4.Weight | 0.644157 | 0.056997 | 0.999113 |
| Ethanoligenens | EDEQ4.Restraint | 0.644316 | 0.050455 | 0.999113 |
| Enterobacter | BMI | 0.645867 | -0.04855 | 0.999113 |
| Hydrotalea | Mini.IPIP.Extraversion | 0.646587 | -0.04522 | 0.999113 |
| Catenibacterium | Mini.IPIP.Agreeableness | 0.646634 | 0.04797 | 0.999113 |
| Anaerotruncus | BAI.score | 0.647339 | -0.03306 | 0.999113 |
| Clostridium.XI | Mini.IPIP.Extraversion | 0.648295 | 0.047488 | 0.999113 |
| Turicibacter | EDEQ4.Eating | 0.648435 | -0.05237 | 0.999113 |
| Acetanaerobacterium | Weight.lbs | 0.64856 | -0.05226 | 0.999113 |
| Victivallis | Mini.IPIP.Imagination | 0.649714 | -0.05148 | 0.999113 |
| Adlercreutzia | Mini.IPIP.Neuroticism | 0.649841 | -0.04649 | 0.999113 |
| Papillibacter | Mini.IPIP.Imagination | 0.650007 | 0.045315 | 0.999113 |
| Acetivibrio | EDEQ4.Eating | 0.650241 | -0.05322 | 0.999113 |
| Gemella | BMI | 0.650424 | -0.05034 | 0.999113 |
| Prevotella | Mini.IPIP.Extraversion | 0.650935 | 0.048229 | 0.999113 |
| Paludibacter | EDEQ4.Weight | 0.65095 | 0.055114 | 0.999113 |
| Prevotella | Qualtrics.age | 0.651235 | -0.0464 | 0.999113 |
| Parabacteroides | EDEQ4.Weight | 0.651484 | 0.053627 | 0.999113 |
| Oxalobacter | Mini.IPIP.Neuroticism | 0.65205 | -0.05409 | 0.999113 |
| Atopobium | BMI | 0.652066 | 0.055663 | 0.999113 |
| Collinsella | Mini.IPIP.Imagination | 0.6527 | 0.059696 | 0.999113 |
| Tepidibacter | Height.inches | 0.652944 | 0.051785 | 0.999113 |
| Sporacetigenium | EDEQ4.Weight | 0.653468 | -0.05338 | 0.999113 |
| Sporacetigenium | EDEQ4.Total | 0.653806 | 0.057173 | 0.999113 |
| Adlercreutzia | EDEQ4.Total | 0.654069 | -0.04459 | 0.999113 |
| Lactobacillus | BMI | 0.654267 | -0.04431 | 0.999113 |
| Gemmiger | EDEQ4.Total | 0.654531 | 0.044579 | 0.999113 |
| Actinomyces | EDEQ4.Restraint | 0.655425 | 0.057918 | 0.999113 |
| Murdochiella | EDEQ4.Shape | 0.656859 | 0.046389 | 0.999113 |
| Clostridium.III | Mini.IPIP.Extraversion | 0.657939 | 0.044332 | 0.999113 |
| Ethanoligenens | Mini.IPIP.Extraversion | 0.658876 | -0.04022 | 0.999113 |
| Varibaculum | Mini.IPIP.Imagination | 0.659299 | -0.04929 | 0.999113 |
| Candidatus.Carsonella | EDEQ4.Eating | 0.659543 | -0.04678 | 0.999113 |
| Hydrogenoanaerobacterium | BMI | 0.660396 | -0.05695 | 0.999113 |
| Butyricicoccus | Mini.IPIP.Conscientiousness | 0.660587 | -0.04958 | 0.999113 |
| Marvinbryantia | Qualtrics.age | 0.66186 | -0.04179 | 0.999113 |
| Phascolarctobacterium | Mini.IPIP.Imagination | 0.663373 | -0.04552 | 0.999113 |
| Hespellia | Mini.IPIP.Extraversion | 0.663544 | -0.04709 | 0.999113 |
| Streptophyta | BMI | 0.663845 | 0.048422 | 0.999113 |
| Adlercreutzia | Qualtrics.age | 0.664089 | 0.047882 | 0.999113 |
| Clostridium.IV | Mini.IPIP.Extraversion | 0.664244 | 0.04216 | 0.999113 |
| Coprococcus | EDEQ4.Shape | 0.664361 | -0.0421 | 0.999113 |
| Ruminococcus | BDI.II.score | 0.665355 | -0.04958 | 0.999113 |
| Parabacteroides | EDEQ4.Eating | 0.666126 | 0.040792 | 0.999113 |
| Megamonas | PSS.10.score | 0.666631 | -0.05104 | 0.999113 |
| Mogibacterium | EDEQ4.Shape | 0.666975 | -0.03921 | 0.999113 |
| Papillibacter | Qualtrics.age | 0.667172 | 0.04644 | 0.999113 |
| Escherichia.Shigella | EDEQ4.Total | 0.667377 | 0.058589 | 0.999113 |
| Bacteroides | EDEQ4.Weight | 0.667702 | 0.046749 | 0.999113 |
| Megasphaera | Weight.lbs | 0.667938 | 0.04049 | 0.999113 |
| Gardnerella | Mini.IPIP.Imagination | 0.668647 | -0.03859 | 0.999113 |
| Clostridium.XVIII | Mini.IPIP.Imagination | 0.668736 | 0.037934 | 0.999113 |
| Natronincola | EDEQ4.Total | 0.669642 | 0.046256 | 0.999113 |
| shannonDiversity (genus) | EDEQ4.Shape | 0.66971 | 0.058765 | 0.999113 |
| Roseburia | BDI.II.score | 0.670737 | 0.054812 | 0.999113 |
| Sporacetigenium | Mini.IPIP.Imagination | 0.670741 | 0.046176 | 0.999113 |
| Lachnospira | Mini.IPIP.Agreeableness | 0.670806 | -0.0479 | 0.999113 |
| Pyrolobus | BAI.score | 0.67203 | 0.045273 | 0.999113 |
| Weissella | PSS.10.score | 0.672902 | 0.046237 | 0.999113 |
| Faecalibacterium | Mini.IPIP.Agreeableness | 0.67341 | 0.054379 | 0.999113 |
| Lactonifactor | Mini.IPIP.Agreeableness | 0.67341 | 0.054379 | 0.999113 |
| Syntrophococcus | EDEQ4.Total | 0.674257 | 0.046899 | 0.999113 |
| Campylobacter | Height.inches | 0.675027 | -0.04211 | 0.999113 |
| Lachnoanaerobaculum | Mini.IPIP.Conscientiousness | 0.675173 | -0.03617 | 0.999113 |
| Clostridium.sensu.stricto | PSS.10.score | 0.675252 | 0.047338 | 0.999113 |
| Coprococcus | PSS.10.score | 0.675342 | -0.05551 | 0.999113 |
| Sarcina | Mini.IPIP.Imagination | 0.675665 | -0.04539 | 0.999113 |
| Flavonifractor | Height.inches | 0.676023 | 0.045312 | 0.999113 |
| Gardnerella | BDI.II.score | 0.676047 | 0.053669 | 0.999113 |
| Moryella | Mini.IPIP.Neuroticism | 0.676602 | -0.05476 | 0.999113 |
| Oxalobacter | PSS.10.score | 0.676795 | -0.04953 | 0.999113 |
| Acetanaerobacterium | BAI.score | 0.676815 | -0.02759 | 0.999113 |
| Acetitomaculum | PSS.10.score | 0.677481 | 0.048493 | 0.999113 |
| Ruminococcus | EDEQ4.Restraint | 0.677803 | 0.050479 | 0.999113 |
| Flavonifractor | Weight.lbs | 0.677905 | -0.05219 | 0.999113 |
| Bacteroides | BMI | 0.678472 | 0.046245 | 0.999113 |
| Mogibacterium | BDI.II.score | 0.678784 | 0.027029 | 0.999113 |
| Parabacteroides | Mini.IPIP.Neuroticism | 0.679751 | -0.04667 | 0.999113 |
| Lachnospira | BAI.score | 0.679961 | 0.051846 | 0.999113 |
| Acidaminococcus | Mini.IPIP.Neuroticism | 0.680377 | 0.051475 | 0.999113 |
| Natronincola | Mini.IPIP.Agreeableness | 0.680715 | 0.04673 | 0.999113 |
| Megasphaera | EDEQ4.Restraint | 0.681051 | -0.04537 | 0.999113 |
| Catenibacterium | Height.inches | 0.681439 | -0.05763 | 0.999113 |
| Pyrolobus | Mini.IPIP.Neuroticism | 0.681545 | -0.04356 | 0.999113 |
| Bifidobacterium | BDI.II.score | 0.681549 | 0.043591 | 0.999113 |
| Acetivibrio | EDEQ4.Shape | 0.681946 | 0.051848 | 0.999113 |
| Hydrotalea | Weight.lbs | 0.682301 | -0.04535 | 0.999113 |
| Lachnospira | Mini.IPIP.Neuroticism | 0.683014 | -0.04091 | 0.999113 |
| Anaerosporobacter | Mini.IPIP.Extraversion | 0.683038 | -0.03971 | 0.999113 |
| Eggerthia | Mini.IPIP.Conscientiousness | 0.683396 | -0.05031 | 0.999113 |
| Catenibacterium | EDEQ4.Restraint | 0.683443 | 0.049447 | 0.999113 |
| Sporacetigenium | Mini.IPIP.Agreeableness | 0.683583 | 0.044559 | 0.999113 |
| Holdemania | EDEQ4.Eating | 0.683967 | -0.04387 | 0.999113 |
| Catenibacterium | EDEQ4.Eating | 0.683973 | 0.046759 | 0.999113 |
| Lactobacillus | Height.inches | 0.683975 | -0.04593 | 0.999113 |
| Coprobacillus | Mini.IPIP.Agreeableness | 0.684099 | -0.03722 | 0.999113 |
| Collinsella | Mini.IPIP.Agreeableness | 0.68424 | 0.046776 | 0.999113 |
| Roseburia | Mini.IPIP.Agreeableness | 0.68424 | 0.044774 | 0.999113 |
| Peptostreptococcus | Qualtrics.age | 0.684749 | 0.05034 | 0.999113 |
| Faecalibacterium | BAI.score | 0.684935 | 0.048179 | 0.999113 |
| Gardnerella | Mini.IPIP.Neuroticism | 0.685074 | 0.047157 | 0.999113 |
| Finegoldia | Qualtrics.age | 0.686381 | -0.04636 | 0.999113 |
| Clostridium.XVIII | EDEQ4.Total | 0.686608 | -0.04732 | 0.999113 |
| Escherichia.Shigella | Mini.IPIP.Neuroticism | 0.687456 | 0.037896 | 0.999113 |
| Murdochiella | Mini.IPIP.Extraversion | 0.687462 | -0.04358 | 0.999113 |
| Peptostreptococcus | Mini.IPIP.Extraversion | 0.687478 | -0.03981 | 0.999113 |
| Haemophilus | EDEQ4.Shape | 0.688318 | -0.04482 | 0.999113 |
| Gallibacterium | BAI.score | 0.688584 | -0.03499 | 0.999113 |
| Subdoligranulum | Weight.lbs | 0.688699 | 0.039765 | 0.999113 |
| Alistipes | Mini.IPIP.Agreeableness | 0.68968 | 0.048355 | 0.999113 |
| Sutterella | EDEQ4.Shape | 0.689903 | 0.052525 | 0.999113 |
| Tannerella | Mini.IPIP.Extraversion | 0.68996 | -0.04238 | 0.999113 |
| Gallibacterium | BMI | 0.690095 | -0.04287 | 0.999113 |
| Atopobium | Height.inches | 0.690176 | 0.042988 | 0.999113 |
| Flavonifractor | Mini.IPIP.Imagination | 0.690355 | -0.04006 | 0.999113 |
| Shuttleworthia | EDEQ4.Weight | 0.690853 | 0.042548 | 0.999113 |
| Fusobacterium | EDEQ4.Weight | 0.69129 | 0.055088 | 0.999113 |
| Paludibacter | Mini.IPIP.Imagination | 0.691388 | -0.04072 | 0.999113 |
| Ruminococcus2 | Qualtrics.age | 0.691434 | 0.045422 | 0.999113 |
| Oxalobacter | Mini.IPIP.Extraversion | 0.691462 | 0.042995 | 0.999113 |
| Parvimonas | Mini.IPIP.Agreeableness | 0.691565 | 0.047862 | 0.999113 |
| Clostridium.XI | EDEQ4.Total | 0.692011 | -0.02678 | 0.999113 |
| Parasporobacterium | BDI.II.score | 0.693126 | -0.04842 | 0.999113 |
| Anaerofustis | EDEQ4.Shape | 0.693525 | 0.039289 | 0.999113 |
| Granulicatella | Mini.IPIP.Agreeableness | 0.694003 | -0.04542 | 0.999113 |
| Paralactobacillus | EDEQ4.Weight | 0.695177 | -0.04528 | 0.999113 |
| Oxalobacter | BMI | 0.695214 | -0.05091 | 0.999113 |
| Pyrolobus | EDEQ4.Total | 0.695326 | 0.047869 | 0.999113 |
| Anaerofilum | Mini.IPIP.Agreeableness | 0.695462 | 0.04159 | 0.999113 |
| Lactococcus | Mini.IPIP.Conscientiousness | 0.695691 | -0.04153 | 0.999113 |
| Clostridium.XlVa | BAI.score | 0.695809 | 0.040262 | 0.999113 |
| Pseudobutyrivibrio | EDEQ4.Total | 0.696343 | 0.04125 | 0.999113 |
| Parasutterella | EDEQ4.Weight | 0.697757 | -0.04398 | 0.999113 |
| Abiotrophia | Mini.IPIP.Agreeableness | 0.698203 | 0.04345 | 0.999113 |
| Natronincola | Mini.IPIP.Extraversion | 0.69854 | 0.04345 | 0.999113 |
| Dialister | Weight.lbs | 0.699556 | -0.04936 | 0.999113 |
| Lachnobacterium | BDI.II.score | 0.699858 | -0.0385 | 0.999113 |
| Faecalibacterium | EDEQ4.Weight | 0.700603 | 0.049595 | 0.999113 |
| Blautia | Mini.IPIP.Agreeableness | 0.700607 | 0.053617 | 0.999113 |
| Dorea | Mini.IPIP.Imagination | 0.701261 | -0.04204 | 0.999113 |
| Clostridium.IV | Mini.IPIP.Neuroticism | 0.701485 | -0.02843 | 0.999113 |
| Lachnospiracea_incertae_sedis | EDEQ4.Eating | 0.701998 | -0.04446 | 0.999113 |
| Clostridium.sensu.stricto | Qualtrics.age | 0.702223 | -0.04317 | 0.999113 |
| Anaerorhabdus | BMI | 0.702343 | 0.036488 | 0.999113 |
| Acidaminococcus | BAI.score | 0.702668 | 0.045458 | 0.999113 |
| Methanobrevibacter | Height.inches | 0.703355 | 0.043479 | 0.999113 |
| Acetitomaculum | Mini.IPIP.Conscientiousness | 0.704163 | -0.04107 | 0.999113 |
| Olsenella | EDEQ4.Weight | 0.705621 | 0.036205 | 0.999113 |
| Dorea | Mini.IPIP.Agreeableness | 0.706094 | 0.051349 | 0.999113 |
| Clostridium.XI | EDEQ4.Restraint | 0.706178 | 0.040882 | 0.999113 |
| Lachnoanaerobaculum | Mini.IPIP.Extraversion | 0.706366 | 0.042997 | 0.999113 |
| Ruminococcus2 | Mini.IPIP.Imagination | 0.706737 | -0.04473 | 0.999113 |
| Varibaculum | BDI.II.score | 0.706887 | -0.03664 | 0.999113 |
| Lachnobacterium | PSS.10.score | 0.707092 | 0.031412 | 0.999113 |
| Oribacterium | Mini.IPIP.Extraversion | 0.707203 | 0.050317 | 0.999113 |
| Parabacteroides | Mini.IPIP.Extraversion | 0.707508 | 0.051016 | 0.999113 |
| Methanobrevibacter | PSS.10.score | 0.707967 | 0.037416 | 0.999113 |
| Bacteroides | EDEQ4.Total | 0.708315 | 0.04983 | 0.999113 |
| Fusobacterium | Qualtrics.age | 0.708479 | -0.04198 | 0.999113 |
| Asaccharobacter | EDEQ4.Restraint | 0.708924 | -0.04084 | 0.999113 |
| Candidatus.Carsonella | PSS.10.score | 0.709972 | -0.03538 | 0.999113 |
| Phascolarctobacterium | Weight.lbs | 0.710475 | 0.039902 | 0.999113 |
| Actinomyces | BMI | 0.71099 | -0.0463 | 0.999113 |
| Campylobacter | Mini.IPIP.Neuroticism | 0.711378 | 0.044623 | 0.999113 |
| Bacteroides | BAI.score | 0.712237 | 0.046511 | 0.999113 |
| Tepidibacter | EDEQ4.Shape | 0.712245 | 0.047974 | 0.999113 |
| Subdoligranulum | Mini.IPIP.Neuroticism | 0.712445 | 0.040662 | 0.999113 |
| Rothia | Qualtrics.age | 0.712852 | -0.04087 | 0.999113 |
| Parasutterella | EDEQ4.Shape | 0.713008 | 0.036637 | 0.999113 |
| Clostridium.XI | EDEQ4.Shape | 0.713088 | 0.050305 | 0.999113 |
| Varibaculum | EDEQ4.Eating | 0.713537 | -0.04009 | 0.999113 |
| Sarcina | EDEQ4.Weight | 0.714093 | -0.0394 | 0.999113 |
| Butyricicoccus | Height.inches | 0.714275 | 0.040655 | 0.999113 |
| Hespellia | Mini.IPIP.Imagination | 0.714378 | -0.04248 | 0.999113 |
| Parvimonas | Mini.IPIP.Neuroticism | 0.71503 | -0.04402 | 0.999113 |
| Ethanoligenens | EDEQ4.Total | 0.716487 | -0.03913 | 0.999113 |
| Pseudoflavonifractor | EDEQ4.Eating | 0.717147 | -0.04272 | 0.999113 |
| Acetivibrio | EDEQ4.Weight | 0.717314 | 0.046708 | 0.999113 |
| Lachnospira | BMI | 0.718476 | 0.037106 | 0.999113 |
| Mogibacterium | PSS.10.score | 0.718763 | -0.03181 | 0.999113 |
| Lactovum | EDEQ4.Total | 0.71887 | 0.040155 | 0.999113 |
| Victivallis | EDEQ4.Shape | 0.719158 | -0.04455 | 0.999113 |
| Streptophyta | EDEQ4.Restraint | 0.719488 | 0.040306 | 0.999113 |
| Phascolarctobacterium | Mini.IPIP.Conscientiousness | 0.7204 | 0.0399 | 0.999113 |
| Prevotella | Mini.IPIP.Imagination | 0.720488 | -0.03378 | 0.999113 |
| Oxalobacter | Mini.IPIP.Agreeableness | 0.720569 | 0.035557 | 0.999113 |
| Robinsoniella | Mini.IPIP.Agreeableness | 0.721199 | 0.039322 | 0.999113 |
| Lachnoanaerobaculum | BDI.II.score | 0.721755 | -0.04605 | 0.999113 |
| Gallibacterium | Weight.lbs | 0.721833 | -0.03404 | 0.999113 |
| Varibaculum | Qualtrics.age | 0.722423 | -0.04341 | 0.999113 |
| Actinomyces | Mini.IPIP.Agreeableness | 0.722646 | -0.03735 | 0.999113 |
| Flavonifractor | Mini.IPIP.Agreeableness | 0.722646 | 0.045004 | 0.999113 |
| Gemmiger | EDEQ4.Weight | 0.722858 | 0.031685 | 0.999113 |
| Turicibacter | BAI.score | 0.723236 | -0.0486 | 0.999113 |
| Akkermansia | Mini.IPIP.Extraversion | 0.723988 | -0.04559 | 0.999113 |
| Lactovum | Mini.IPIP.Neuroticism | 0.72478 | 0.038797 | 0.999113 |
| Clostridium.III | BAI.score | 0.724875 | 0.032 | 0.999113 |
| Shuttleworthia | EDEQ4.Restraint | 0.724924 | -0.03501 | 0.999113 |
| Olsenella | PSS.10.score | 0.725248 | 0.039346 | 0.999113 |
| Butyricicoccus | BDI.II.score | 0.725438 | -0.01944 | 0.999113 |
| Finegoldia | PSS.10.score | 0.726024 | -0.03791 | 0.999113 |
| Odoribacter | Mini.IPIP.Neuroticism | 0.726181 | -0.02251 | 0.999113 |
| Papillibacter | Weight.lbs | 0.726936 | -0.0355 | 0.999113 |
| Anaerotruncus | PSS.10.score | 0.727023 | -0.03826 | 0.999113 |
| Lachnospira | EDEQ4.Total | 0.727144 | 0.033715 | 0.999113 |
| Ruminococcus | BMI | 0.727453 | -0.0338 | 0.999113 |
| Clostridium.XlVb | Mini.IPIP.Imagination | 0.728792 | -0.03967 | 0.999113 |
| Oscillibacter | BAI.score | 0.7288 | 0.044651 | 0.999113 |
| Alistipes | Mini.IPIP.Neuroticism | 0.728997 | 0.03798 | 0.999113 |
| Eubacterium | EDEQ4.Restraint | 0.729164 | -0.03953 | 0.999113 |
| Gardnerella | Mini.IPIP.Extraversion | 0.729434 | -0.03156 | 0.999113 |
| Bifidobacterium | Mini.IPIP.Extraversion | 0.72951 | 0.029959 | 0.999113 |
| Paludibacter | PSS.10.score | 0.729741 | -0.03728 | 0.999113 |
| Parabacteroides | EDEQ4.Total | 0.730262 | 0.035089 | 0.999113 |
| Lachnoanaerobaculum | EDEQ4.Restraint | 0.731432 | 0.030437 | 0.999113 |
| Enterorhabdus | EDEQ4.Restraint | 0.731632 | 0.040384 | 0.999113 |
| Weissella | EDEQ4.Restraint | 0.732001 | 0.0373 | 0.999113 |
| Shuttleworthia | Weight.lbs | 0.733071 | -0.03755 | 0.999113 |
| Granulicatella | Mini.IPIP.Conscientiousness | 0.733249 | -0.04507 | 0.999113 |
| Megasphaera | BMI | 0.733727 | 0.0407 | 0.999113 |
| Bifidobacterium | EDEQ4.Weight | 0.734075 | -0.03786 | 0.999113 |
| Paraprevotella | PSS.10.score | 0.73416 | -0.03655 | 0.999113 |
| Lactobacillus | BAI.score | 0.734196 | 0.043135 | 0.999113 |
| Defluviitalea | Mini.IPIP.Imagination | 0.73434 | 0.044909 | 0.999113 |
| Streptophyta | EDEQ4.Total | 0.734773 | -0.03098 | 0.999113 |
| Leuconostoc | EDEQ4.Shape | 0.735129 | 0.047563 | 0.999113 |
| Parabacteroides | EDEQ4.Shape | 0.735141 | -0.04088 | 0.999113 |
| Oribacterium | BAI.score | 0.736855 | 0.033864 | 0.999113 |
| Shuttleworthia | BMI | 0.736984 | -0.03574 | 0.999113 |
| Veillonella | BAI.score | 0.737124 | 0.04577 | 0.999113 |
| Slackia | PSS.10.score | 0.737351 | -0.02571 | 0.999113 |
| Anaerovorax | EDEQ4.Eating | 0.737803 | 0.042047 | 0.999113 |
| Barnesiella | EDEQ4.Shape | 0.737885 | 0.040012 | 0.999113 |
| Clostridium.XlVb | Weight.lbs | 0.73803 | -0.02935 | 0.999113 |
| Lachnospira | Weight.lbs | 0.738189 | 0.038828 | 0.999113 |
| Hespellia | Height.inches | 0.738704 | 0.020398 | 0.999113 |
| Anaerorhabdus | EDEQ4.Total | 0.739574 | 0.037555 | 0.999113 |
| Tannerella | EDEQ4.Weight | 0.73958 | 0.038933 | 0.999113 |
| Mogibacterium | Mini.IPIP.Imagination | 0.73986 | 0.046692 | 0.999113 |
| Saccharibacteria_genera_incertae_sedis | Mini.IPIP.Conscientiousness | 0.740455 | 0.036864 | 0.999113 |
| Lactonifactor | Mini.IPIP.Extraversion | 0.740596 | 0.026193 | 0.999113 |
| Holdemania | BMI | 0.741264 | -0.02919 | 0.999113 |
| Robinsoniella | Mini.IPIP.Imagination | 0.741337 | -0.03716 | 0.999113 |
| Lachnospiracea_incertae_sedis | BDI.II.score | 0.742141 | -0.02967 | 0.999113 |
| Eubacterium | Mini.IPIP.Conscientiousness | 0.742592 | -0.03482 | 0.999113 |
| Solobacterium | Weight.lbs | 0.743232 | 0.029506 | 0.999113 |
| Oxalobacter | EDEQ4.Shape | 0.743513 | 0.034749 | 0.999113 |
| Atopobium | Mini.IPIP.Imagination | 0.743674 | -0.03144 | 0.999113 |
| Eggerthella | PSS.10.score | 0.743682 | -0.02145 | 0.999113 |
| Robinsoniella | Mini.IPIP.Conscientiousness | 0.744054 | 0.040846 | 0.999113 |
| Anaerostipes | EDEQ4.Eating | 0.744698 | 0.035834 | 0.999113 |
| Megamonas | BMI | 0.744855 | -0.03926 | 0.999113 |
| Finegoldia | Mini.IPIP.Neuroticism | 0.74534 | -0.03642 | 0.999113 |
| Lachnobacterium | Mini.IPIP.Extraversion | 0.745493 | -0.02991 | 0.999113 |
| Peptoniphilus | Mini.IPIP.Imagination | 0.74587 | -0.02711 | 0.999113 |
| Gordonibacter | EDEQ4.Restraint | 0.746548 | 0.032144 | 0.999113 |
| Acetanaerobacterium | EDEQ4.Total | 0.746863 | -0.03183 | 0.999113 |
| Tannerella | Qualtrics.age | 0.746957 | 0.038247 | 0.999113 |
| Enterorhabdus | BDI.II.score | 0.74753 | 0.03505 | 0.999113 |
| Streptococcus | BDI.II.score | 0.747736 | -0.03329 | 0.999113 |
| Robinsoniella | PSS.10.score | 0.747915 | -0.04256 | 0.999113 |
| Streptophyta | Mini.IPIP.Extraversion | 0.747991 | -0.03356 | 0.999113 |
| Papillibacter | EDEQ4.Shape | 0.74901 | 0.037079 | 0.999113 |
| Metascardovia | EDEQ4.Restraint | 0.749419 | -0.03758 | 0.999113 |
| Shuttleworthia | Mini.IPIP.Conscientiousness | 0.749589 | 0.032811 | 0.999113 |
| Tepidibacter | BAI.score | 0.749674 | -0.04058 | 0.999113 |
| Sporobacter | Mini.IPIP.Agreeableness | 0.750521 | -0.03867 | 0.999113 |
| Adlercreutzia | EDEQ4.Shape | 0.751487 | -0.02838 | 0.999113 |
| Clostridium.XlVa | Mini.IPIP.Extraversion | 0.751737 | -0.04296 | 0.999113 |
| Dorea | Mini.IPIP.Extraversion | 0.751737 | -0.02429 | 0.999113 |
| Hallella | BMI | 0.75264 | -0.03802 | 0.999113 |
| Barnesiella | Mini.IPIP.Imagination | 0.753849 | 0.026676 | 0.999113 |
| Anaerococcus | Qualtrics.age | 0.754201 | -0.0369 | 0.999113 |
| Sutterella | Height.inches | 0.754972 | -0.03931 | 0.999113 |
| Coprobacillus | Qualtrics.age | 0.755262 | -0.03283 | 0.999113 |
| Clostridium.IV | Mini.IPIP.Agreeableness | 0.756136 | 0.045196 | 0.999113 |
| Lachnospiracea_incertae_sedis | Mini.IPIP.Imagination | 0.756678 | -0.0379 | 0.999113 |
| Megasphaera | Mini.IPIP.Imagination | 0.756738 | -0.0349 | 0.999113 |
| Rothia | Weight.lbs | 0.75719 | 0.035281 | 0.999113 |
| Natronincola | EDEQ4.Restraint | 0.757649 | 0.037597 | 0.999113 |
| Lactobacillus | Mini.IPIP.Conscientiousness | 0.759262 | 0.034547 | 0.999113 |
| Dialister | Mini.IPIP.Conscientiousness | 0.759403 | 0.033153 | 0.999113 |
| Anaerococcus | Mini.IPIP.Neuroticism | 0.759583 | 0.025833 | 0.999113 |
| Gordonibacter | Mini.IPIP.Neuroticism | 0.759625 | -0.03666 | 0.999113 |
| Marvinbryantia | Mini.IPIP.Neuroticism | 0.759663 | -0.04327 | 0.999113 |
| Campylobacter | Mini.IPIP.Agreeableness | 0.759899 | -0.02967 | 0.999113 |
| Turicibacter | EDEQ4.Shape | 0.760188 | 0.026361 | 0.999113 |
| Lactobacillus | Qualtrics.age | 0.7602 | 0.036201 | 0.999113 |
| Ruminococcus | Weight.lbs | 0.76032 | 0.030523 | 0.999113 |
| Leuconostoc | EDEQ4.Restraint | 0.761644 | 0.029851 | 0.999113 |
| Syntrophococcus | Mini.IPIP.Neuroticism | 0.762098 | 0.029508 | 0.999113 |
| Abiotrophia | EDEQ4.Restraint | 0.762219 | 0.031405 | 0.999113 |
| Dorea | EDEQ4.Shape | 0.763015 | 0.045426 | 0.999113 |
| Clostridium.XI | PSS.10.score | 0.763214 | 0.0217 | 0.999113 |
| Granulicatella | BDI.II.score | 0.763705 | 0.039339 | 0.999113 |
| Sporacetigenium | BAI.score | 0.764611 | -0.03243 | 0.999113 |
| Murdochiella | Weight.lbs | 0.764812 | 0.031947 | 0.999113 |
| Anaerostipes | Mini.IPIP.Conscientiousness | 0.765027 | 0.032466 | 0.999113 |
| Roseburia | Mini.IPIP.Conscientiousness | 0.765027 | 0.036206 | 0.999113 |
| Sporobacterium | EDEQ4.Shape | 0.765321 | 0.033354 | 0.999113 |
| Campylobacter | Weight.lbs | 0.76782 | 0.039276 | 0.999113 |
| Eggerthella | Mini.IPIP.Conscientiousness | 0.767843 | 0.026073 | 0.999113 |
| Catenibacterium | EDEQ4.Weight | 0.76806 | -0.04237 | 0.999113 |
| Mogibacterium | Weight.lbs | 0.76869 | 0.034711 | 0.999113 |
| Lactonifactor | Qualtrics.age | 0.768753 | 0.039313 | 0.999113 |
| Acetitomaculum | Mini.IPIP.Extraversion | 0.770682 | 0.037127 | 0.999113 |
| Lachnobacterium | EDEQ4.Shape | 0.770834 | 0.032975 | 0.999113 |
| Gemmiger | Mini.IPIP.Agreeableness | 0.773059 | 0.031432 | 0.999113 |
| Hespellia | BMI | 0.774273 | -0.02691 | 0.999113 |
| Moryella | EDEQ4.Eating | 0.774686 | 0.025836 | 0.999113 |
| Clostridium.XI | EDEQ4.Eating | 0.775695 | 0.048769 | 0.999113 |
| Clostridium.XlVb | EDEQ4.Eating | 0.775695 | 0.0312 | 0.999113 |
| Parabacteroides | Height.inches | 0.775827 | -0.01689 | 0.999113 |
| Sarcina | EDEQ4.Total | 0.776339 | 0.045392 | 0.999113 |
| Hespellia | BDI.II.score | 0.778265 | -0.02041 | 0.999113 |
| Anaerotruncus | Mini.IPIP.Agreeableness | 0.778686 | -0.028 | 0.999113 |
| Bilophila | BAI.score | 0.779049 | 0.039206 | 0.999113 |
| Lactobacillus | Mini.IPIP.Imagination | 0.779089 | -0.03738 | 0.999113 |
| Dialister | BAI.score | 0.779226 | -0.03385 | 0.999113 |
| Metascardovia | BMI | 0.780971 | -0.03478 | 0.999113 |
| Gardnerella | Height.inches | 0.781439 | 0.029236 | 0.999113 |
| Alistipes | BDI.II.score | 0.781578 | -0.02978 | 0.999113 |
| Faecalibacterium | EDEQ4.Restraint | 0.781799 | -0.0409 | 0.999113 |
| Gemmiger | Mini.IPIP.Conscientiousness | 0.781975 | -0.02925 | 0.999113 |
| Dorea | Weight.lbs | 0.782811 | 0.033171 | 0.999113 |
| Ruminococcus2 | BMI | 0.783206 | -0.05492 | 0.999113 |
| Pseudoflavonifractor | EDEQ4.Total | 0.783247 | -0.02651 | 0.999113 |
| Lachnospira | Height.inches | 0.783755 | 0.038111 | 0.999113 |
| Ethanoligenens | Height.inches | 0.784319 | -0.02523 | 0.999113 |
| Megasphaera | EDEQ4.Weight | 0.785054 | 0.025341 | 0.999113 |
| Coprobacillus | Mini.IPIP.Conscientiousness | 0.785644 | 0.02597 | 0.999113 |
| Dialister | EDEQ4.Total | 0.786068 | -0.03946 | 0.999113 |
| Defluviitalea | EDEQ4.Total | 0.786605 | -0.02278 | 0.999113 |
| Tannerella | Weight.lbs | 0.786783 | 0.023702 | 0.999113 |
| Tepidibacter | EDEQ4.Eating | 0.787015 | 0.02916 | 0.999113 |
| Solobacterium | Mini.IPIP.Conscientiousness | 0.787358 | -0.025 | 0.999113 |
| Lactobacillus | Mini.IPIP.Extraversion | 0.788167 | -0.00985 | 0.999113 |
| Dorea | EDEQ4.Eating | 0.788198 | -0.0312 | 0.999113 |
| Phascolarctobacterium | EDEQ4.Eating | 0.788198 | 0.027242 | 0.999113 |
| Paralactobacillus | Mini.IPIP.Conscientiousness | 0.789788 | 0.037021 | 0.999113 |
| Lachnospiracea_incertae_sedis | Mini.IPIP.Agreeableness | 0.790091 | -0.04297 | 0.999113 |
| Prevotella | BDI.II.score | 0.790101 | 0.039138 | 0.999113 |
| Syntrophococcus | EDEQ4.Restraint | 0.790843 | -0.03125 | 0.999113 |
| Faecalibacterium | EDEQ4.Shape | 0.7912 | 0.042283 | 0.999113 |
| Actinomyces | Qualtrics.age | 0.791313 | 0.019469 | 0.999113 |
| Hydrotalea | Mini.IPIP.Imagination | 0.791939 | -0.0311 | 0.999113 |
| Anaerofilum | PSS.10.score | 0.792408 | 0.026778 | 0.999113 |
| Peptoniphilus | EDEQ4.Shape | 0.792562 | -0.02698 | 0.999113 |
| shannonDiversity (genus) | BDI.II.score | 0.792954 | 0.030377 | 0.999113 |
| Ethanoligenens | Mini.IPIP.Imagination | 0.793382 | -0.02524 | 0.999113 |
| Anaerovorax | BDI.II.score | 0.793458 | -0.0281 | 0.999113 |
| Dorea | EDEQ4.Restraint | 0.793639 | -0.03946 | 0.999113 |
| Desulfovibrio | Height.inches | 0.793774 | -0.03803 | 0.999113 |
| Bilophila | Qualtrics.age | 0.793979 | -0.03684 | 0.999113 |
| Anaerococcus | BMI | 0.793983 | -0.03111 | 0.999113 |
| Victivallis | EDEQ4.Eating | 0.79473 | 0.028835 | 0.999113 |
| Syntrophococcus | EDEQ4.Eating | 0.794894 | 0.028666 | 0.999113 |
| Anaerofustis | EDEQ4.Eating | 0.795482 | 0.033037 | 0.999113 |
| Dialister | Mini.IPIP.Agreeableness | 0.795791 | 0.025959 | 0.999113 |
| Paludibacter | BMI | 0.795821 | -0.03342 | 0.999113 |
| Sutterella | PSS.10.score | 0.796114 | -0.03014 | 0.999113 |
| Gemmiger | Mini.IPIP.Imagination | 0.796248 | -0.02851 | 0.999113 |
| Parvimonas | Mini.IPIP.Extraversion | 0.796314 | 0.02581 | 0.999113 |
| Lachnospira | EDEQ4.Restraint | 0.796972 | 0.024566 | 0.999113 |
| Butyricimonas | Mini.IPIP.Imagination | 0.798452 | 0.026563 | 0.999113 |
| Hespellia | Weight.lbs | 0.799364 | 0.028609 | 0.999113 |
| Anaerofustis | Mini.IPIP.Agreeableness | 0.79937 | -0.03707 | 0.999113 |
| Bacteroides | Weight.lbs | 0.799801 | 0.016969 | 0.999113 |
| Sutterella | Mini.IPIP.Agreeableness | 0.8006 | 0.028703 | 0.999113 |
| Marvinbryantia | EDEQ4.Eating | 0.800751 | 0.029999 | 0.999113 |
| Roseburia | EDEQ4.Eating | 0.800755 | 0.023163 | 0.999113 |
| Sutterella | Mini.IPIP.Neuroticism | 0.801198 | 0.027954 | 0.999113 |
| Moryella | EDEQ4.Restraint | 0.801841 | 0.025126 | 0.999113 |
| Solobacterium | EDEQ4.Restraint | 0.802286 | 0.030149 | 0.999113 |
| Actinobacillus | EDEQ4.Restraint | 0.803373 | 0.03027 | 0.999113 |
| Hydrotalea | Mini.IPIP.Conscientiousness | 0.80351 | 0.01937 | 0.999113 |
| Phascolarctobacterium | Height.inches | 0.804305 | -0.01923 | 0.999113 |
| Eggerthella | Mini.IPIP.Agreeableness | 0.80436 | -0.02958 | 0.999113 |
| Oscillibacter | BDI.II.score | 0.804375 | -0.02556 | 0.999113 |
| Robinsoniella | EDEQ4.Restraint | 0.8045 | 0.019621 | 0.999113 |
| Slackia | Mini.IPIP.Neuroticism | 0.804542 | 0.025752 | 0.999113 |
| Acetanaerobacterium | EDEQ4.Weight | 0.805381 | -0.03236 | 0.999113 |
| Asaccharobacter | EDEQ4.Total | 0.805796 | -0.02981 | 0.999113 |
| Barnesiella | BDI.II.score | 0.807217 | 0.023538 | 0.999113 |
| Abiotrophia | Qualtrics.age | 0.809622 | -0.02429 | 0.999113 |
| Peptoniphilus | BMI | 0.810408 | -0.01943 | 0.999113 |
| Bilophila | Mini.IPIP.Neuroticism | 0.810509 | -0.01924 | 0.999113 |
| Syntrophococcus | Mini.IPIP.Conscientiousness | 0.810747 | -0.03012 | 0.999113 |
| Mogibacterium | Qualtrics.age | 0.811164 | 0.030921 | 0.999113 |
| Streptococcus | BMI | 0.811526 | -0.03095 | 0.999113 |
| Megamonas | Height.inches | 0.812249 | -0.01265 | 0.999113 |
| Moryella | EDEQ4.Shape | 0.813284 | 0.021358 | 0.999113 |
| Sarcina | BMI | 0.81334 | 0.022242 | 0.999113 |
| Erysipelotrichaceae_incertae_sedis | Mini.IPIP.Imagination | 0.813376 | -0.0229 | 0.999113 |
| Bifidobacterium | Mini.IPIP.Neuroticism | 0.81352 | 0.029208 | 0.999113 |
| Pseudobutyrivibrio | EDEQ4.Weight | 0.813525 | 0.013136 | 0.999113 |
| Shuttleworthia | Mini.IPIP.Neuroticism | 0.813699 | 0.027686 | 0.999113 |
| Parasutterella | Mini.IPIP.Extraversion | 0.813827 | 0.017634 | 0.999113 |
| Parasporobacterium | Mini.IPIP.Agreeableness | 0.814005 | 0.02111 | 0.999113 |
| Subdoligranulum | EDEQ4.Weight | 0.814066 | 0.023444 | 0.999113 |
| Clostridium.III | PSS.10.score | 0.81416 | -0.02661 | 0.999113 |
| Adlercreutzia | EDEQ4.Restraint | 0.814223 | -0.02621 | 0.999113 |
| Moryella | Mini.IPIP.Extraversion | 0.81613 | 0.027316 | 0.999113 |
| Lachnoanaerobaculum | Height.inches | 0.816155 | 0.028513 | 0.999113 |
| Oscillibacter | Weight.lbs | 0.816886 | -0.03398 | 0.999113 |
| Eggerthella | EDEQ4.Weight | 0.816964 | 0.030985 | 0.999113 |
| Holdemania | EDEQ4.Total | 0.817253 | 0.014778 | 0.999113 |
| Tepidibacter | Qualtrics.age | 0.817774 | 0.027545 | 0.999113 |
| Moryella | Mini.IPIP.Imagination | 0.81856 | 0.020262 | 0.999113 |
| shannonDiversity (genus) | Mini.IPIP.Agreeableness | 0.818697 | -0.0292 | 0.999113 |
| Haemophilus | BAI.score | 0.818965 | -0.02672 | 0.999113 |
| Corynebacterium | Mini.IPIP.Neuroticism | 0.819365 | -0.02926 | 0.999113 |
| Leuconostoc | Mini.IPIP.Extraversion | 0.820531 | -0.03452 | 0.999113 |
| Methanobrevibacter | Mini.IPIP.Imagination | 0.821484 | -0.01995 | 0.999113 |
| Anaerovorax | BMI | 0.82161 | -0.02325 | 0.999113 |
| Akkermansia | BMI | 0.822926 | 0.023442 | 0.999113 |
| Anaerorhabdus | BDI.II.score | 0.823287 | 0.033356 | 0.999113 |
| Streptococcus | EDEQ4.Restraint | 0.823443 | 0.029021 | 0.999113 |
| Weissella | Weight.lbs | 0.823508 | 0.023914 | 0.999113 |
| Anaerococcus | Mini.IPIP.Imagination | 0.823741 | -0.02884 | 0.999113 |
| Hydrotalea | Mini.IPIP.Neuroticism | 0.824183 | 0.036735 | 0.999113 |
| Alistipes | BAI.score | 0.82485 | 0.031062 | 0.999113 |
| Bifidobacterium | BAI.score | 0.82485 | -0.01493 | 0.999113 |
| Haemophilus | Weight.lbs | 0.825319 | 0.029083 | 0.999113 |
| Hallella | Qualtrics.age | 0.825397 | 0.027086 | 0.999113 |
| Anaerofustis | BDI.II.score | 0.825474 | -0.01925 | 0.999113 |
| Bacteroides | Qualtrics.age | 0.825478 | 0.027222 | 0.999113 |
| Collinsella | PSS.10.score | 0.825532 | 0.025571 | 0.999113 |
| Varibaculum | Mini.IPIP.Extraversion | 0.825979 | 0.017673 | 0.999113 |
| Coprococcus | EDEQ4.Eating | 0.826022 | -0.02861 | 0.999113 |
| Saccharibacteria_genera_incertae_sedis | EDEQ4.Total | 0.826223 | 0.021895 | 0.999113 |
| Corynebacterium | BDI.II.score | 0.8276 | -0.02738 | 0.999113 |
| Oribacterium | BMI | 0.828454 | -0.01858 | 0.999113 |
| Flavonifractor | BAI.score | 0.830598 | 0.027846 | 0.999113 |
| Subdoligranulum | BAI.score | 0.830598 | 0.026481 | 0.999113 |
| Paludibacter | Mini.IPIP.Extraversion | 0.830631 | -0.02546 | 0.999113 |
| Finegoldia | Mini.IPIP.Conscientiousness | 0.830706 | 0.028386 | 0.999113 |
| Escherichia.Shigella | Qualtrics.age | 0.830971 | 0.028437 | 0.999113 |
| Flavonifractor | EDEQ4.Shape | 0.831114 | 0.016382 | 0.999113 |
| Gordonibacter | BMI | 0.831469 | 0.004218 | 0.999113 |
| Weissella | EDEQ4.Weight | 0.832129 | 0.019864 | 0.999113 |
| Bacteroides | EDEQ4.Eating | 0.832368 | -0.02544 | 0.999113 |
| Barnesiella | Mini.IPIP.Agreeableness | 0.833076 | 0.023216 | 0.999113 |
| Akkermansia | BDI.II.score | 0.833106 | 0.042002 | 0.999113 |
| Catenibacterium | BDI.II.score | 0.833106 | 0.027346 | 0.999113 |
| Fusobacterium | EDEQ4.Shape | 0.833125 | 0.025728 | 0.999113 |
| Sutterella | Qualtrics.age | 0.833308 | -0.0294 | 0.999113 |
| Asaccharobacter | Mini.IPIP.Imagination | 0.8334 | 0.023778 | 0.999113 |
| Parasutterella | EDEQ4.Total | 0.834347 | 0.009547 | 0.999113 |
| Enterobacter | Qualtrics.age | 0.834623 | 0.021934 | 0.999113 |
| Shuttleworthia | BDI.II.score | 0.834802 | 0.020358 | 0.999113 |
| Barnesiella | Mini.IPIP.Conscientiousness | 0.836276 | 0.030801 | 0.999113 |
| Hydrogenoanaerobacterium | EDEQ4.Weight | 0.836308 | 0.020719 | 0.999113 |
| Collinsella | Mini.IPIP.Extraversion | 0.836795 | 0.013024 | 0.999113 |
| Erysipelotrichaceae_incertae_sedis | Qualtrics.age | 0.836943 | 0.027149 | 0.999113 |
| Dorea | EDEQ4.Weight | 0.837323 | -0.02554 | 0.999113 |
| Gallibacterium | EDEQ4.Restraint | 0.838073 | 0.027134 | 0.999113 |
| Gemmiger | EDEQ4.Eating | 0.838725 | -0.02605 | 0.999113 |
| Hydrogenoanaerobacterium | EDEQ4.Eating | 0.840942 | -0.02272 | 0.999113 |
| Odoribacter | Mini.IPIP.Agreeableness | 0.84173 | -0.0175 | 0.999113 |
| Phascolarctobacterium | Mini.IPIP.Agreeableness | 0.841758 | 0.023415 | 0.999113 |
| Metascardovia | Mini.IPIP.Agreeableness | 0.841821 | 0.019962 | 0.999113 |
| Weissella | Qualtrics.age | 0.842039 | 0.022045 | 0.999113 |
| Porphyromonas | EDEQ4.Shape | 0.842758 | -0.0239 | 0.999113 |
| Shuttleworthia | EDEQ4.Shape | 0.842847 | -0.02716 | 0.999113 |
| Hespellia | PSS.10.score | 0.845274 | 0.031337 | 0.999113 |
| Alistipes | BMI | 0.845836 | 0.019339 | 0.999113 |
| Pyrolobus | Weight.lbs | 0.846107 | -0.01996 | 0.999113 |
| Defluviitalea | Weight.lbs | 0.846776 | 0.018225 | 0.999113 |
| Flavonifractor | EDEQ4.Restraint | 0.847473 | 0.030291 | 0.999113 |
| Anaerococcus | PSS.10.score | 0.849167 | 0.016001 | 0.999113 |
| Tepidibacter | Mini.IPIP.Extraversion | 0.850018 | -0.01815 | 0.999113 |
| Bacteroides | Height.inches | 0.8504 | 0.011096 | 0.999113 |
| Lachnobacterium | Mini.IPIP.Neuroticism | 0.850498 | -0.01751 | 0.999113 |
| Anaerovorax | Mini.IPIP.Neuroticism | 0.850794 | -0.0201 | 0.999113 |
| Asaccharobacter | EDEQ4.Shape | 0.851179 | -0.0219 | 0.999113 |
| Oribacterium | EDEQ4.Total | 0.851451 | -0.01284 | 0.999113 |
| Phascolarctobacterium | BMI | 0.851584 | 0.011689 | 0.999113 |
| Hydrotalea | EDEQ4.Eating | 0.851604 | 0.0254 | 0.999113 |
| Candidatus.Carsonella | BDI.II.score | 0.853033 | 0.028572 | 0.999113 |
| Clostridium.III | EDEQ4.Eating | 0.853036 | -0.01662 | 0.999113 |
| Enterorhabdus | Weight.lbs | 0.853147 | 0.01822 | 0.999113 |
| Oscillibacter | Mini.IPIP.Agreeableness | 0.853342 | 0.019293 | 0.999113 |
| Pseudoflavonifractor | Mini.IPIP.Conscientiousness | 0.853622 | -0.02353 | 0.999113 |
| Butyricicoccus | Mini.IPIP.Neuroticism | 0.853788 | -0.02905 | 0.999113 |
| Clostridium.XVIII | Mini.IPIP.Neuroticism | 0.853788 | -0.02425 | 0.999113 |
| Sporobacterium | PSS.10.score | 0.853843 | 0.017123 | 0.999113 |
| Oribacterium | Weight.lbs | 0.854035 | 0.025452 | 0.999113 |
| Abiotrophia | BAI.score | 0.855371 | -0.02194 | 0.999113 |
| Anaerococcus | EDEQ4.Total | 0.855602 | -0.02203 | 0.999113 |
| Streptophyta | BDI.II.score | 0.855696 | 0.017052 | 0.999113 |
| Prevotella | Mini.IPIP.Neuroticism | 0.856677 | 0.016152 | 0.999113 |
| Gardnerella | EDEQ4.Shape | 0.856971 | 0.013827 | 0.999113 |
| Anaerofustis | Height.inches | 0.857575 | 0.017615 | 0.999113 |
| Anaerococcus | BDI.II.score | 0.85764 | 0.015808 | 0.999113 |
| Dialister | EDEQ4.Eating | 0.857858 | -0.03085 | 0.999113 |
| Desulfovibrio | Weight.lbs | 0.858208 | 0.021525 | 0.999113 |
| Murdochiella | Mini.IPIP.Conscientiousness | 0.858495 | -0.02432 | 0.999113 |
| Alistipes | EDEQ4.Shape | 0.8599 | 0.01682 | 0.999113 |
| Odoribacter | BMI | 0.860196 | -0.00757 | 0.999113 |
| Flavonifractor | EDEQ4.Total | 0.860249 | -0.02957 | 0.999113 |
| Sporobacterium | Mini.IPIP.Neuroticism | 0.86031 | 0.020383 | 0.999113 |
| Syntrophococcus | BAI.score | 0.86046 | 0.020786 | 0.999113 |
| Leuconostoc | Mini.IPIP.Agreeableness | 0.860552 | -0.01861 | 0.999113 |
| Ruminococcus | EDEQ4.Weight | 0.860722 | -0.01666 | 0.999113 |
| Gemella | PSS.10.score | 0.861212 | 0.014654 | 0.999113 |
| Ruminococcus2 | Height.inches | 0.862011 | -0.00653 | 0.999113 |
| Sporobacterium | EDEQ4.Weight | 0.86207 | -0.02254 | 0.999113 |
| Lachnoanaerobaculum | EDEQ4.Eating | 0.86241 | 0.016186 | 0.999113 |
| Tepidibacter | EDEQ4.Weight | 0.862561 | -0.01713 | 0.999113 |
| Solobacterium | EDEQ4.Shape | 0.862597 | -0.01461 | 0.999113 |
| Sarcina | EDEQ4.Eating | 0.862637 | -0.01714 | 0.999113 |
| Fusobacterium | Height.inches | 0.862694 | -0.01326 | 0.999113 |
| Odoribacter | Mini.IPIP.Extraversion | 0.862714 | 0.013186 | 0.999113 |
| Haemophilus | BMI | 0.862991 | 0.020521 | 0.999113 |
| Megamonas | EDEQ4.Shape | 0.863078 | -0.01369 | 0.999113 |
| Megamonas | EDEQ4.Weight | 0.864025 | -0.02452 | 0.999113 |
| Clostridium.sensu.stricto | EDEQ4.Eating | 0.864219 | 0.024694 | 0.999113 |
| Gallibacterium | Height.inches | 0.864755 | 0.016056 | 0.999113 |
| Bifidobacterium | Mini.IPIP.Agreeableness | 0.864957 | -0.0193 | 0.999113 |
| Holdemania | BAI.score | 0.865265 | 0.018555 | 0.999113 |
| Escherichia.Shigella | EDEQ4.Restraint | 0.865407 | -0.01293 | 0.999113 |
| Murdochiella | Height.inches | 0.865583 | -0.02021 | 0.999113 |
| Phascolarctobacterium | EDEQ4.Restraint | 0.86559 | 0.02331 | 0.999113 |
| Gordonibacter | EDEQ4.Shape | 0.865656 | -0.02213 | 0.999113 |
| Clostridium.XlVb | Qualtrics.age | 0.865754 | 0.029825 | 0.999113 |
| Sarcina | EDEQ4.Restraint | 0.866767 | -0.02312 | 0.999113 |
| Atopobium | PSS.10.score | 0.867723 | -0.0191 | 0.999113 |
| Anaerorhabdus | Mini.IPIP.Agreeableness | 0.868495 | 0.01748 | 0.999113 |
| Gemella | Mini.IPIP.Extraversion | 0.869832 | -0.01964 | 0.999113 |
| Porphyromonas | EDEQ4.Total | 0.870543 | 0.016349 | 0.999113 |
| Enterorhabdus | PSS.10.score | 0.871385 | 0.021246 | 0.999113 |
| Clostridium.III | EDEQ4.Weight | 0.8714 | -0.02103 | 0.999113 |
| Bifidobacterium | Qualtrics.age | 0.871539 | 0.006812 | 0.999113 |
| Eggerthia | Mini.IPIP.Neuroticism | 0.872324 | -0.01223 | 0.999113 |
| Defluviitalea | EDEQ4.Eating | 0.872521 | 0.014103 | 0.999113 |
| Odoribacter | Mini.IPIP.Imagination | 0.873952 | -0.0152 | 0.999113 |
| Shuttleworthia | EDEQ4.Eating | 0.874429 | -0.0186 | 0.999113 |
| Solobacterium | EDEQ4.Total | 0.874502 | 0.003592 | 0.999113 |
| Lachnoanaerobaculum | Mini.IPIP.Neuroticism | 0.875269 | 0.013062 | 0.999113 |
| Campylobacter | EDEQ4.Shape | 0.875749 | -0.02047 | 0.999113 |
| Clostridium.III | EDEQ4.Shape | 0.876419 | -0.01948 | 0.999113 |
| Solobacterium | EDEQ4.Eating | 0.876912 | 0.016591 | 0.999113 |
| Anaerotruncus | Mini.IPIP.Neuroticism | 0.876956 | -0.01623 | 0.999113 |
| Butyricicoccus | PSS.10.score | 0.877369 | -0.00662 | 0.999113 |
| Campylobacter | EDEQ4.Weight | 0.877394 | 0.020042 | 0.999113 |
| Methanobrevibacter | Mini.IPIP.Neuroticism | 0.877558 | -0.01383 | 0.999113 |
| Sporobacterium | Qualtrics.age | 0.877956 | -0.01909 | 0.999113 |
| Desulfovibrio | EDEQ4.Weight | 0.878763 | 0.015015 | 0.999113 |
| Acetitomaculum | Mini.IPIP.Imagination | 0.879321 | 0.023003 | 0.999113 |
| Gemmiger | Height.inches | 0.879482 | -0.02267 | 0.999113 |
| Clostridium.XlVb | BDI.II.score | 0.879526 | 0.028366 | 0.999113 |
| Holdemania | Mini.IPIP.Extraversion | 0.880117 | -0.02578 | 0.999113 |
| Turicibacter | Weight.lbs | 0.880205 | 0.015564 | 0.999113 |
| Escherichia.Shigella | BMI | 0.880271 | -0.01633 | 0.999113 |
| Roseburia | Mini.IPIP.Imagination | 0.882701 | 0.019783 | 0.999113 |
| Clostridium.XlVb | EDEQ4.Shape | 0.883065 | 0.005884 | 0.999113 |
| Veillonella | Weight.lbs | 0.883116 | 0.018778 | 0.999113 |
| Paludibacter | Height.inches | 0.883622 | 0.015817 | 0.999113 |
| Megasphaera | Height.inches | 0.884559 | -0.01904 | 0.999113 |
| Peptoniphilus | EDEQ4.Weight | 0.884778 | 0.018605 | 0.999113 |
| Saccharibacteria_genera_incertae_sedis | EDEQ4.Shape | 0.885349 | -0.01825 | 0.999113 |
| Candidatus.Carsonella | Mini.IPIP.Neuroticism | 0.885452 | 0.018621 | 0.999113 |
| Marvinbryantia | BAI.score | 0.885614 | -0.01958 | 0.999113 |
| Lachnobacterium | Weight.lbs | 0.885711 | 0.016289 | 0.999113 |
| Lactobacillus | Weight.lbs | 0.885951 | -0.00207 | 0.999113 |
| Slackia | Mini.IPIP.Imagination | 0.886147 | 0.021853 | 0.999113 |
| shannonDiversity (genus) | BMI | 0.886231 | -0.02078 | 0.999113 |
| Mogibacterium | EDEQ4.Total | 0.886232 | 0.016886 | 0.999113 |
| Megamonas | Qualtrics.age | 0.886774 | -0.01977 | 0.999113 |
| Syntrophococcus | PSS.10.score | 0.887418 | -0.01265 | 0.999113 |
| Slackia | EDEQ4.Weight | 0.887893 | 0.012138 | 0.999113 |
| Hallella | PSS.10.score | 0.888115 | -0.01716 | 0.999113 |
| Coprococcus | Mini.IPIP.Agreeableness | 0.888272 | 0.009385 | 0.999113 |
| Solobacterium | PSS.10.score | 0.888812 | 0.015407 | 0.999113 |
| Clostridium.III | Weight.lbs | 0.889053 | 0.02315 | 0.999113 |
| Anaerostipes | EDEQ4.Restraint | 0.889855 | 0.020379 | 0.999113 |
| Finegoldia | Mini.IPIP.Agreeableness | 0.889978 | -0.01218 | 0.999113 |
| Clostridium.XVIII | EDEQ4.Weight | 0.890143 | 0.017474 | 0.999113 |
| Tannerella | EDEQ4.Shape | 0.890499 | 0.01764 | 0.999113 |
| Oribacterium | Mini.IPIP.Imagination | 0.891325 | 0.005375 | 0.999113 |
| Marvinbryantia | PSS.10.score | 0.89187 | 0.020934 | 0.999113 |
| Bifidobacterium | BMI | 0.892029 | -0.00844 | 0.999113 |
| Robinsoniella | Mini.IPIP.Extraversion | 0.894073 | 0.019826 | 0.999113 |
| Asaccharobacter | BAI.score | 0.894317 | 0.010491 | 0.999113 |
| Anaerosporobacter | Mini.IPIP.Conscientiousness | 0.894319 | -0.00917 | 0.999113 |
| Bacteroides | Mini.IPIP.Imagination | 0.894358 | -0.02068 | 0.999113 |
| Papillibacter | EDEQ4.Total | 0.894939 | 0.013712 | 0.999113 |
| Paludibacter | EDEQ4.Total | 0.895279 | -0.01607 | 0.999113 |
| Mogibacterium | EDEQ4.Restraint | 0.89592 | 0.008701 | 0.999113 |
| Butyricicoccus | EDEQ4.Restraint | 0.895938 | -0.01535 | 0.999113 |
| Turicibacter | EDEQ4.Weight | 0.896035 | 0.013053 | 0.999113 |
| Collinsella | EDEQ4.Weight | 0.896046 | -0.01192 | 0.999113 |
| Erysipelotrichaceae_incertae_sedis | EDEQ4.Eating | 0.896364 | 0.013833 | 0.999113 |
| Fusobacterium | Mini.IPIP.Agreeableness | 0.896829 | -0.01178 | 0.999113 |
| Lachnobacterium | EDEQ4.Restraint | 0.898715 | -0.01805 | 0.999113 |
| Metascardovia | Mini.IPIP.Conscientiousness | 0.89941 | -0.00179 | 0.999113 |
| Anaerorhabdus | Weight.lbs | 0.899462 | -0.01599 | 0.999113 |
| Lactococcus | BDI.II.score | 0.899973 | -0.01005 | 0.999113 |
| Acidaminococcus | PSS.10.score | 0.900144 | 0.005627 | 0.999113 |
| Ruminococcus2 | Mini.IPIP.Extraversion | 0.900469 | 0.012905 | 0.999113 |
| Bacteroides | PSS.10.score | 0.900592 | -0.01867 | 0.999113 |
| Veillonella | BMI | 0.900734 | 0.016312 | 0.999113 |
| Erysipelotrichaceae_incertae_sedis | EDEQ4.Total | 0.900756 | 0.01258 | 0.999113 |
| Clostridium.III | Mini.IPIP.Neuroticism | 0.901403 | 0.005688 | 0.999113 |
| Solobacterium | BAI.score | 0.902984 | -0.012 | 0.999113 |
| shannonDiversity (genus) | Mini.IPIP.Conscientiousness | 0.903083 | 0.016077 | 0.999113 |
| Victivallis | EDEQ4.Weight | 0.903185 | 0.010214 | 0.999113 |
| Anaerosporobacter | EDEQ4.Shape | 0.903415 | 0.013796 | 0.999113 |
| Gemella | EDEQ4.Weight | 0.903741 | -0.01382 | 0.999113 |
| Paralactobacillus | Qualtrics.age | 0.904221 | 0.019435 | 0.999113 |
| Megamonas | EDEQ4.Total | 0.90474 | -0.02622 | 0.999113 |
| Clostridium.XI | Mini.IPIP.Agreeableness | 0.905824 | 0.020743 | 0.999113 |
| Butyricicoccus | Mini.IPIP.Imagination | 0.906039 | 0.002355 | 0.999113 |
| Erysipelotrichaceae_incertae_sedis | BAI.score | 0.906042 | 0.019966 | 0.999113 |
| Streptococcus | BAI.score | 0.906042 | 0.024337 | 0.999113 |
| Sporobacter | PSS.10.score | 0.906412 | -0.01365 | 0.999113 |
| Prevotella | BMI | 0.906545 | 0.001878 | 0.999113 |
| Gordonibacter | EDEQ4.Total | 0.906548 | -0.01816 | 0.999113 |
| Dorea | EDEQ4.Total | 0.906566 | -0.02485 | 0.999113 |
| Eggerthia | BDI.II.score | 0.90749 | -0.01835 | 0.999113 |
| Slackia | Mini.IPIP.Extraversion | 0.907826 | 0.006496 | 0.999113 |
| Lachnospira | Mini.IPIP.Conscientiousness | 0.907889 | -0.00581 | 0.999113 |
| Akkermansia | EDEQ4.Eating | 0.909259 | -0.01248 | 0.999113 |
| Anaerotruncus | Weight.lbs | 0.90927 | -0.01325 | 0.999113 |
| Fusobacterium | Mini.IPIP.Neuroticism | 0.910786 | 0.011515 | 0.999113 |
| Fusobacterium | EDEQ4.Total | 0.91119 | 0.025022 | 0.999113 |
| Solobacterium | BDI.II.score | 0.911568 | 0.00577 | 0.999113 |
| Ruminococcus | Mini.IPIP.Agreeableness | 0.911685 | 0.005353 | 0.999113 |
| Papillibacter | Mini.IPIP.Conscientiousness | 0.911846 | -0.01139 | 0.999113 |
| Shuttleworthia | Mini.IPIP.Extraversion | 0.911862 | 0.022098 | 0.999113 |
| Clostridium.sensu.stricto | EDEQ4.Weight | 0.913766 | -0.0206 | 0.999113 |
| Natronincola | Mini.IPIP.Neuroticism | 0.914649 | 0.012925 | 0.999113 |
| Olsenella | EDEQ4.Restraint | 0.914851 | -0.01667 | 0.999113 |
| Coprococcus | Weight.lbs | 0.915116 | -0.00956 | 0.999113 |
| Turicibacter | PSS.10.score | 0.915142 | 0.009486 | 0.999113 |
| Tepidibacter | EDEQ4.Total | 0.915743 | -0.01091 | 0.999113 |
| Paraprevotella | Mini.IPIP.Imagination | 0.916453 | 0.005284 | 0.999113 |
| Enterobacter | BAI.score | 0.916574 | -0.00394 | 0.999113 |
| Enterobacter | Mini.IPIP.Extraversion | 0.916792 | 0.011862 | 0.999113 |
| Sutterella | Mini.IPIP.Conscientiousness | 0.917329 | -0.00702 | 0.999113 |
| Bilophila | Height.inches | 0.91746 | -0.00839 | 0.999113 |
| Bilophila | Mini.IPIP.Agreeableness | 0.917484 | 0.015374 | 0.999113 |
| Ruminococcus2 | Mini.IPIP.Agreeableness | 0.917551 | -0.0042 | 0.999113 |
| Asaccharobacter | Weight.lbs | 0.917997 | -0.02196 | 0.999113 |
| Actinomyces | PSS.10.score | 0.918066 | 0.016989 | 0.999113 |
| Parvimonas | Qualtrics.age | 0.918441 | 0.003635 | 0.999113 |
| Moryella | BAI.score | 0.919126 | 0.00732 | 0.999113 |
| Lactovum | EDEQ4.Weight | 0.919417 | -0.00909 | 0.999113 |
| Anaerotruncus | EDEQ4.Weight | 0.9197 | -0.00338 | 0.999113 |
| Erysipelotrichaceae_incertae_sedis | EDEQ4.Weight | 0.919715 | 0.016695 | 0.999113 |
| Coprococcus | EDEQ4.Weight | 0.919715 | -0.0127 | 0.999113 |
| Anaerofustis | Mini.IPIP.Neuroticism | 0.91986 | 0.002679 | 0.999113 |
| Robinsoniella | Height.inches | 0.92003 | -0.01156 | 0.999113 |
| Hydrogenoanaerobacterium | Mini.IPIP.Imagination | 0.920164 | -0.02662 | 0.999113 |
| Erysipelotrichaceae_incertae_sedis | Height.inches | 0.920462 | -0.01618 | 0.999113 |
| Eggerthella | EDEQ4.Shape | 0.920914 | 0.011928 | 0.999113 |
| Lachnospiracea_incertae_sedis | Weight.lbs | 0.92095 | 0.014047 | 0.999113 |
| Acetitomaculum | Weight.lbs | 0.921659 | 0.011262 | 0.999113 |
| Bifidobacterium | EDEQ4.Eating | 0.922177 | 0.000737 | 0.999113 |
| Oscillibacter | EDEQ4.Eating | 0.922177 | -0.01404 | 0.999113 |
| Sporobacter | EDEQ4.Eating | 0.922177 | 0.008926 | 0.999113 |
| Anaerofustis | EDEQ4.Total | 0.923186 | -0.01321 | 0.999113 |
| Anaerorhabdus | EDEQ4.Restraint | 0.923411 | -0.00929 | 0.999113 |
| Barnesiella | Mini.IPIP.Neuroticism | 0.923648 | -0.00585 | 0.999113 |
| Actinomyces | EDEQ4.Total | 0.924026 | -0.00565 | 0.999113 |
| Oxalobacter | BDI.II.score | 0.924663 | 0.008722 | 0.999113 |
| Dialister | EDEQ4.Weight | 0.925644 | -0.01506 | 0.999113 |
| Flavonifractor | EDEQ4.Weight | 0.925644 | -0.01645 | 0.999113 |
| Oribacterium | EDEQ4.Restraint | 0.926362 | -0.00943 | 0.999113 |
| Dialister | EDEQ4.Restraint | 0.92644 | 0.011698 | 0.999113 |
| Clostridium.III | Mini.IPIP.Conscientiousness | 0.926682 | -0.0076 | 0.999113 |
| Metascardovia | EDEQ4.Shape | 0.927 | 0.003042 | 0.999113 |
| Peptoniphilus | Mini.IPIP.Conscientiousness | 0.92791 | -0.01404 | 0.999113 |
| Pseudobutyrivibrio | EDEQ4.Eating | 0.9283 | -0.00907 | 0.999113 |
| Ruminococcus | EDEQ4.Eating | 0.928644 | 0.009855 | 0.999113 |
| Victivallis | BDI.II.score | 0.928886 | -0.01141 | 0.999113 |
| Actinomyces | Mini.IPIP.Neuroticism | 0.929513 | 0.009248 | 0.999113 |
| Sporobacter | Mini.IPIP.Neuroticism | 0.929513 | 0.011088 | 0.999113 |
| Robinsoniella | EDEQ4.Weight | 0.931207 | 0.003858 | 0.999113 |
| Fusobacterium | EDEQ4.Eating | 0.931791 | -0.00644 | 0.999113 |
| Actinomyces | Mini.IPIP.Conscientiousness | 0.932366 | -0.01106 | 0.999113 |
| Saccharibacteria_genera_incertae_sedis | Height.inches | 0.933097 | 0.002922 | 0.999113 |
| Solobacterium | EDEQ4.Weight | 0.934455 | 0.003462 | 0.999113 |
| Solobacterium | Mini.IPIP.Agreeableness | 0.935084 | 0.003761 | 0.999113 |
| Lactonifactor | Mini.IPIP.Imagination | 0.935322 | -0.00884 | 0.999113 |
| Lactococcus | Mini.IPIP.Neuroticism | 0.935372 | -0.00879 | 0.999113 |
| Gemmiger | EDEQ4.Shape | 0.935524 | -0.00494 | 0.999113 |
| Lachnospiracea_incertae_sedis | EDEQ4.Shape | 0.935524 | -0.00908 | 0.999113 |
| Veillonella | EDEQ4.Restraint | 0.935612 | -0.01048 | 0.999113 |
| Peptoniphilus | Mini.IPIP.Extraversion | 0.937093 | 0.006976 | 0.999113 |
| Shuttleworthia | Qualtrics.age | 0.937558 | -0.00541 | 0.999113 |
| Lachnobacterium | Mini.IPIP.Agreeableness | 0.937948 | 0.003277 | 0.999113 |
| Papillibacter | Mini.IPIP.Agreeableness | 0.938108 | 0.001212 | 0.999113 |
| Parasutterella | BAI.score | 0.938241 | -0.01411 | 0.999113 |
| Desulfovibrio | Mini.IPIP.Conscientiousness | 0.938275 | -0.00119 | 0.999113 |
| Desulfovibrio | Mini.IPIP.Neuroticism | 0.938343 | -0.0088 | 0.999113 |
| Tannerella | PSS.10.score | 0.939502 | -0.00722 | 0.999113 |
| Parasporobacterium | Mini.IPIP.Neuroticism | 0.939688 | -0.00464 | 0.999113 |
| Varibaculum | Mini.IPIP.Neuroticism | 0.940225 | 0.006522 | 0.999113 |
| Anaerofustis | Mini.IPIP.Conscientiousness | 0.940525 | 0.008194 | 0.999113 |
| Granulicatella | Mini.IPIP.Neuroticism | 0.941007 | 0.009834 | 0.999113 |
| Peptostreptococcus | BAI.score | 0.941539 | -0.00633 | 0.999113 |
| Anaerofilum | Mini.IPIP.Imagination | 0.942002 | 0.007514 | 0.999113 |
| Hallella | Mini.IPIP.Neuroticism | 0.942226 | -0.0059 | 0.999113 |
| Actinobacillus | EDEQ4.Total | 0.942228 | -0.00537 | 0.999113 |
| Paraprevotella | EDEQ4.Total | 0.943564 | -0.00512 | 0.999113 |
| Varibaculum | Weight.lbs | 0.9441 | -0.01063 | 0.999113 |
| Olsenella | Height.inches | 0.94426 | -0.01258 | 0.999113 |
| Anaerorhabdus | Mini.IPIP.Conscientiousness | 0.944848 | 0.005829 | 0.999113 |
| Anaerofilum | Mini.IPIP.Neuroticism | 0.945092 | 0.006739 | 0.999113 |
| Hallella | BAI.score | 0.945221 | -0.00705 | 0.999113 |
| Corynebacterium | Qualtrics.age | 0.946427 | -0.01648 | 0.999113 |
| Anaerostipes | Mini.IPIP.Agreeableness | 0.946941 | -0.00116 | 0.999113 |
| Anaerotruncus | Mini.IPIP.Conscientiousness | 0.947035 | -0.0048 | 0.999113 |
| Collinsella | Mini.IPIP.Neuroticism | 0.947105 | 0.001721 | 0.999113 |
| Candidatus.Carsonella | Weight.lbs | 0.947135 | -0.00224 | 0.999113 |
| Parabacteroides | PSS.10.score | 0.947274 | 0.015538 | 0.999113 |
| Clostridium.sensu.stricto | EDEQ4.Restraint | 0.947843 | -0.00397 | 0.999113 |
| Natronincola | EDEQ4.Eating | 0.947937 | 0.006671 | 0.999113 |
| Flavonifractor | EDEQ4.Eating | 0.948072 | 0.004513 | 0.999113 |
| Coprobacillus | Height.inches | 0.949387 | 0.006416 | 0.999113 |
| Holdemania | Mini.IPIP.Agreeableness | 0.949884 | -0.00467 | 0.999113 |
| Barnesiella | BAI.score | 0.949996 | -0.00392 | 0.999113 |
| Eggerthella | Mini.IPIP.Imagination | 0.95 | 0.003683 | 0.999113 |
| Natronincola | Qualtrics.age | 0.950048 | -0.01365 | 0.999113 |
| Sporobacterium | EDEQ4.Total | 0.95111 | 0.007726 | 0.999113 |
| Lachnobacterium | EDEQ4.Total | 0.953074 | 0.013134 | 0.999113 |
| Ruminococcus2 | PSS.10.score | 0.953125 | -0.00469 | 0.999113 |
| Parvimonas | EDEQ4.Weight | 0.953929 | -0.00617 | 0.999113 |
| Actinomyces | EDEQ4.Eating | 0.954556 | -0.00092 | 0.999113 |
| Hydrotalea | BAI.score | 0.954923 | 0.005212 | 0.999113 |
| Actinobacillus | EDEQ4.Shape | 0.954944 | 0.004222 | 0.999113 |
| Sporobacter | Mini.IPIP.Conscientiousness | 0.95586 | -0.00032 | 0.999113 |
| Anaerosporobacter | Mini.IPIP.Imagination | 0.955875 | -0.006 | 0.999113 |
| Enterorhabdus | BAI.score | 0.956795 | -0.0032 | 0.999113 |
| Enterorhabdus | Mini.IPIP.Neuroticism | 0.956828 | -0.00189 | 0.999113 |
| Megamonas | Weight.lbs | 0.958159 | -0.01489 | 0.999113 |
| Adlercreutzia | Mini.IPIP.Imagination | 0.958757 | -0.00133 | 0.999113 |
| Catenibacterium | Mini.IPIP.Neuroticism | 0.958847 | -0.00256 | 0.999113 |
| Eubacterium | EDEQ4.Shape | 0.958941 | 0.004824 | 0.999113 |
| Rothia | EDEQ4.Eating | 0.96099 | -0.00842 | 0.999113 |
| Alistipes | EDEQ4.Eating | 0.961042 | 0.001777 | 0.999113 |
| Subdoligranulum | EDEQ4.Eating | 0.961042 | 0.009461 | 0.999113 |
| Finegoldia | EDEQ4.Shape | 0.961053 | -0.0034 | 0.999113 |
| Hydrotalea | PSS.10.score | 0.96108 | 0.007579 | 0.999113 |
| Bacteroides | Mini.IPIP.Conscientiousness | 0.961741 | 0.004042 | 0.999113 |
| Adlercreutzia | Weight.lbs | 0.961837 | -0.01454 | 0.999113 |
| Lactonifactor | Weight.lbs | 0.961891 | -0.0081 | 0.999113 |
| Prevotella | PSS.10.score | 0.961906 | 0.014607 | 0.999113 |
| Pseudobutyrivibrio | BDI.II.score | 0.962734 | 0.005929 | 0.999113 |
| Barnesiella | EDEQ4.Restraint | 0.963177 | -0.01083 | 0.999113 |
| Lachnoanaerobaculum | Mini.IPIP.Imagination | 0.9642 | -0.00802 | 0.999113 |
| Gordonibacter | EDEQ4.Weight | 0.964263 | 0.004265 | 0.999113 |
| Finegoldia | Mini.IPIP.Imagination | 0.964486 | 0.007213 | 0.999113 |
| Clostridium.IV | Mini.IPIP.Imagination | 0.964694 | 0.002172 | 0.999113 |
| Subdoligranulum | Mini.IPIP.Imagination | 0.964694 | 0.00427 | 0.999113 |
| Bifidobacterium | EDEQ4.Shape | 0.964804 | -0.02206 | 0.999113 |
| Oscillibacter | PSS.10.score | 0.964835 | -0.00108 | 0.999113 |
| Turicibacter | EDEQ4.Total | 0.964889 | -0.01272 | 0.999113 |
| Bifidobacterium | EDEQ4.Total | 0.964893 | -0.00242 | 0.999113 |
| Sporobacterium | BMI | 0.965062 | -0.00266 | 0.999113 |
| Lactovum | Mini.IPIP.Imagination | 0.965819 | 0.00532 | 0.999113 |
| Slackia | BAI.score | 0.966047 | 0.007771 | 0.999113 |
| Hallella | Mini.IPIP.Agreeableness | 0.966431 | 0.001159 | 0.999113 |
| Hydrotalea | Qualtrics.age | 0.967054 | -0.00836 | 0.999113 |
| Clostridium.XlVa | EDEQ4.Weight | 0.967245 | -0.00071 | 0.999113 |
| Coprobacillus | Mini.IPIP.Neuroticism | 0.967347 | -0.0107 | 0.999113 |
| Parabacteroides | BDI.II.score | 0.967563 | -0.00589 | 0.999113 |
| Hespellia | BAI.score | 0.967564 | 0.006092 | 0.999113 |
| Butyricimonas | Mini.IPIP.Extraversion | 0.967618 | -0.00111 | 0.999113 |
| Lachnospira | EDEQ4.Weight | 0.96888 | -0.00402 | 0.999113 |
| Acetanaerobacterium | EDEQ4.Restraint | 0.969313 | -0.00088 | 0.999113 |
| Lachnospira | Qualtrics.age | 0.969356 | 0.002561 | 0.999113 |
| Eubacterium | Mini.IPIP.Imagination | 0.970574 | 0.006222 | 0.999113 |
| Prevotella | BAI.score | 0.970576 | 0.013955 | 0.999113 |
| Oribacterium | EDEQ4.Shape | 0.970635 | 0.004783 | 0.999113 |
| Clostridium.XVIII | Mini.IPIP.Extraversion | 0.970657 | -0.00602 | 0.999113 |
| Pyrolobus | EDEQ4.Restraint | 0.971834 | 0.004187 | 0.999113 |
| Coprobacillus | EDEQ4.Restraint | 0.972117 | 0.005995 | 0.999113 |
| Acidaminococcus | Mini.IPIP.Extraversion | 0.972668 | -0.00057 | 0.999113 |
| Megamonas | BAI.score | 0.973001 | -0.01329 | 0.999113 |
| Anaerofilum | EDEQ4.Eating | 0.973045 | -0.00091 | 0.999113 |
| Streptophyta | EDEQ4.Shape | 0.973495 | 0.023082 | 0.999113 |
| Candidatus.Carsonella | EDEQ4.Total | 0.973607 | -0.01208 | 0.999113 |
| Finegoldia | Mini.IPIP.Extraversion | 0.975201 | 0.003338 | 0.999113 |
| Pseudobutyrivibrio | Mini.IPIP.Neuroticism | 0.975224 | -0.00732 | 0.999113 |
| Anaerococcus | EDEQ4.Eating | 0.975505 | 0.000555 | 0.999113 |
| Mogibacterium | EDEQ4.Weight | 0.976171 | 0.008126 | 0.999113 |
| Butyricicoccus | BAI.score | 0.976459 | -0.0034 | 0.999113 |
| Dialister | Mini.IPIP.Extraversion | 0.976523 | 0.013828 | 0.999113 |
| Flavonifractor | Qualtrics.age | 0.976545 | -0.00126 | 0.999113 |
| Streptococcus | PSS.10.score | 0.976552 | -0.00844 | 0.999113 |
| Tepidibacter | Mini.IPIP.Conscientiousness | 0.977858 | -0.00154 | 0.999113 |
| Finegoldia | BMI | 0.978796 | -0.00356 | 0.999113 |
| Gemella | Mini.IPIP.Conscientiousness | 0.979137 | 0.000584 | 0.999113 |
| Butyricimonas | Mini.IPIP.Conscientiousness | 0.979326 | 0.001816 | 0.999113 |
| Mogibacterium | BAI.score | 0.979397 | -0.00328 | 0.999113 |
| Bilophila | EDEQ4.Shape | 0.979448 | -0.00726 | 0.999113 |
| Oxalobacter | Mini.IPIP.Conscientiousness | 0.980356 | 0.004083 | 0.999113 |
| Paludibacter | Weight.lbs | 0.98186 | -0.00288 | 0.999113 |
| Hydrotalea | BDI.II.score | 0.981921 | 0.005448 | 0.999113 |
| Peptoniphilus | BAI.score | 0.981961 | -0.00145 | 0.999113 |
| Alistipes | Mini.IPIP.Extraversion | 0.982391 | 0.008883 | 0.999113 |
| Catenibacterium | EDEQ4.Shape | 0.982398 | -0.00645 | 0.999113 |
| Gardnerella | Weight.lbs | 0.982401 | -0.0001 | 0.999113 |
| Catenibacterium | EDEQ4.Total | 0.982442 | -0.01651 | 0.999113 |
| Subdoligranulum | EDEQ4.Total | 0.982442 | 0.007038 | 0.999113 |
| Oxalobacter | EDEQ4.Weight | 0.983441 | -0.00134 | 0.999113 |
| Porphyromonas | EDEQ4.Restraint | 0.983774 | -0.00271 | 0.999113 |
| Slackia | Mini.IPIP.Agreeableness | 0.984528 | 0.001608 | 0.999113 |
| Acetitomaculum | Qualtrics.age | 0.984912 | -0.00955 | 0.999113 |
| Megamonas | BDI.II.score | 0.984965 | 0.003088 | 0.999113 |
| Lachnobacterium | EDEQ4.Weight | 0.985068 | -0.00353 | 0.999113 |
| Defluviitalea | Mini.IPIP.Agreeableness | 0.98509 | 0.000102 | 0.999113 |
| Anaerosporobacter | EDEQ4.Restraint | 0.987723 | -0.00176 | 0.999113 |
| Corynebacterium | Mini.IPIP.Imagination | 0.988043 | 0.001721 | 0.999113 |
| Odoribacter | EDEQ4.Weight | 0.988084 | -0.00779 | 0.999113 |
| Adlercreutzia | BAI.score | 0.988212 | -0.00673 | 0.999113 |
| Sutterella | Weight.lbs | 0.988215 | 0.013609 | 0.999113 |
| Erysipelotrichaceae_incertae_sedis | PSS.10.score | 0.988275 | -3.65E-05 | 0.999113 |
| Desulfovibrio | PSS.10.score | 0.990285 | -0.0043 | 0.999113 |
| Sarcina | BAI.score | 0.990371 | -0.00557 | 0.999113 |
| Anaerococcus | BAI.score | 0.99048 | -0.00205 | 0.999113 |
| Parvimonas | BAI.score | 0.99089 | 0.00591 | 0.999113 |
| Paraprevotella | Mini.IPIP.Extraversion | 0.991057 | 0.010559 | 0.999113 |
| Lactonifactor | EDEQ4.Weight | 0.991065 | -0.00863 | 0.999113 |
| Granulicatella | Mini.IPIP.Extraversion | 0.99116 | -0.00232 | 0.999113 |
| Catenibacterium | Mini.IPIP.Conscientiousness | 0.991168 | -0.00388 | 0.999113 |
| Ruminococcus | Mini.IPIP.Conscientiousness | 0.991168 | 0.004097 | 0.999113 |
| Odoribacter | PSS.10.score | 0.991204 | -0.00028 | 0.999113 |
| Clostridium.IV | BMI | 0.991219 | 0.008853 | 0.999113 |
| Clostridium.sensu.stricto | BAI.score | 0.994112 | -0.00705 | 0.999402 |
| Clostridium.XI | Mini.IPIP.Imagination | 0.994114 | -0.00449 | 0.999402 |
| Oscillibacter | Mini.IPIP.Imagination | 0.994114 | 0.00941 | 0.999402 |
| Phascolarctobacterium | BAI.score | 0.994114 | 0.006606 | 0.999402 |
| Bacteroides | EDEQ4.Shape | 0.994132 | -0.00799 | 0.999402 |
| Alistipes | PSS.10.score | 0.994137 | -0.01195 | 0.999402 |
| Campylobacter | EDEQ4.Total | 0.996543 | 0.003021 | 0.999694 |
| Lachnospira | EDEQ4.Shape | 0.996933 | -0.00792 | 0.999694 |
| Parvimonas | BMI | 0.996979 | 0.003573 | 0.999694 |
| Gemella | Mini.IPIP.Agreeableness | 0.997014 | 0.001738 | 0.999694 |
| Corynebacterium | BMI | 0.997027 | -0.00676 | 0.999694 |
| Solobacterium | Mini.IPIP.Extraversion | 0.997061 | -0.00999 | 0.999694 |
| Actinobacillus | Height.inches | 1 | -0.00023 | 1 |
| Clostridium.XlVa | Qualtrics.age | 1 | -0.00517 | 1 |
| Gallibacterium | EDEQ4.Weight | 1 | 0.000842 | 1 |
| Megasphaera | BAI.score | 1 | 0.003205 | 1 |
| Pyrolobus | BDI.II.score | 1 | 0.003642 | 1 |
| Shuttleworthia | EDEQ4.Total | 1 | -0.0039 | 1 |

We considered associations between 17 different clinical and psychiatric measures from our human cohort (column B) and 227 bacterial taxa (11 phyla, 18 classes, 21 orders, 44 families, and 133 genera) (column A) present in at least 25% of our samples, as well as the Shannon diversity index. We evaluated 3,944 hypotheses [17 measures * (227 taxa + 5 Shannon diversity metrics)] using the non-parametric Kendall’s tau-b test for association (column D), and there were no associations that met established significance thresholds (column C), even after FDR correction (column E).”
